# Functional characterization of the cytochrome P450 monooxygenase CYP71AU87 indicates a role in marrubiin biosynthesis in the medicinal plant *Marrubium vulgare*

**DOI:** 10.1186/s12870-019-1702-5

**Published:** 2019-03-25

**Authors:** Prema S. Karunanithi, Puja Dhanota, J. Bennett Addison, Shen Tong, Oliver Fiehn, Philipp Zerbe

**Affiliations:** 1 Department of Plant Biology, University of California Davis, 1 Shields Avenue, Davis, CA USA; 2 Department of Chemistry and Biochemistry, San Diego State University, San Diego, CA 92182 USA; 3 West Coast Metabolomics Center, University of California-Davis, 1 Shields Avenue, Davis, CA USA; 4 Biochemistry Department, King Abdulaziz University, Jeddah, Saudi Arabia

**Keywords:** Diterpene synthase, Cytochrome P450 monooxygenase, Diterpenoid biosynthesis, Marrubiin, Plant natural products, *Marrubium vulgare*

## Abstract

**Background:**

Horehound (*Marrubium vulgare*) is a medicinal plant whose signature bioactive compounds, marrubiin and related furanoid diterpenoid lactones, have potential applications for the treatment of cardiovascular diseases and type II diabetes. Lack of scalable plant cultivation and the complex metabolite profile of *M. vulgare* limit access to marrubiin via extraction from plant biomass. Knowledge of the marrubiin-biosynthetic enzymes can enable the development of metabolic engineering platforms for marrubiin production. We previously identified two diterpene synthases, *Mv*CPS1 and *Mv*ELS, that act sequentially to form 9,13-epoxy-labd-14-ene. Conversion of 9,13-epoxy-labd-14-ene by cytochrome P450 monooxygenase (P450) enzymes can be hypothesized to facilitate key functional modification reactions in the formation of marrubiin and related compounds.

**Results:**

Mining a *M. vulgare* leaf transcriptome database identified 95 full-length P450 candidates. Cloning and functional analysis of select P450 candidates showing high transcript abundance revealed a member of the CYP71 family, CYP71AU87, that catalyzed the hydroxylation of 9,13-epoxy-labd-14-ene to yield two isomeric products, 9,13-epoxy labd-14-ene-18-ol and 9,13-epoxy labd-14-ene-19-ol, as verified by GC-MS and NMR analysis. Additional transient *Nicotiana benthamiana* co-expression assays of CYP71AU87 with different diterpene synthase pairs suggested that CYP71AU87 is specific to the sequential *Mv*CPS1 and *Mv*ELS product 9,13-epoxy-labd-14-ene. Although the P450 products were not detectable *in planta*, high levels of *CYP71AU87* gene expression in marrubiin-accumulating tissues supported a role in the formation of marrubiin and related diterpenoids in *M. vulgare*.

**Conclusions:**

In a sequential reaction with the diterpene synthase pair *Mv*CPS1 and *Mv*ELS, CYP71AU87 forms the isomeric products 9,13-epoxy labd-14-ene-18/19-ol as probable intermediates in marrubiin biosynthesis. Although its metabolic relevance *in planta* will necessitate further genetic studies, identification of the CYP71AU87 catalytic activity expands our knowledge of the functional landscape of plant P450 enzymes involved in specialized diterpenoid metabolism and can provide a resource for the formulation of marrubiin and related bioactive natural products.

**Electronic supplementary material:**

The online version of this article (10.1186/s12870-019-1702-5) contains supplementary material, which is available to authorized users.

## Background

Medicinal plants and their natural products are a rich, yet underutilized, source for modern therapeutics that stems from a wealth of knowledge of their use as traditional medicines [1, 2]. Among the myriad natural products formed in plants, diterpenoids are a diverse group of more than 12,000 metabolites [3] that have proven valuable for drug discovery. Examples include the chemotherapeutic agent Taxol® naturally produced in yew trees (*Taxus spp*.) [4, 5], the psychoactive clerodane diterpenoid salvinorin A [6, 7], ingenol mebutate from species of *Euphorbia* for the treatment of actinic keratosis [8, 9], and the cAMP-regulator forskolin from *Coleus forskohlii* [10–12].

This study focuses on the medicinal plant white horehound (*Marrubium vulgare*), a member of the mint family (Lamiaceae), that has been traditionally used to alleviate dermatological and respiratory ailments. *M. vulgare* extracts and metabolites have been shown to have potential for treating type II diabetes and cardiovascular diseases [13, 14]. The anti-diabetic efficacy of *M. vulgare* has been attributed to marrubiin, a furanoid diterpene lactone that represents the signature metabolite in *M. vulgare* [15–17]. In vitro and in vivo studies demonstrated that marrubiin stimulates insulin secretion under hyperglycemic conditions with a potentially higher efficacy as compared to established anti-diabetic drugs such as metformin [13, 14]. Marrubiin accumulates in *M. vulgare* leaf glandular trichomes and flowers at levels of up to 4 mg per gram fresh weight [17]. In addition, phytochemical analyses of *M. vulgare* and closely related *Marrubium* species have revealed a suite of structurally similar labdane diterpenoids that feature common furan ring and 19,6-lactone functional groups [15, 16]. These functional modifications have attracted increasing interest in recent years [7, 16, 18–20], since similar furan and lactone groups have been implicated with natural product bioactivity, such as the contribution of the furan ring in the κ-opioid receptor agonist activity of salvinorin A or the efficacy of sesquiterpene lactones such as the anti-malaria drug artemisinin [21, 22].

**Fig. 1:**
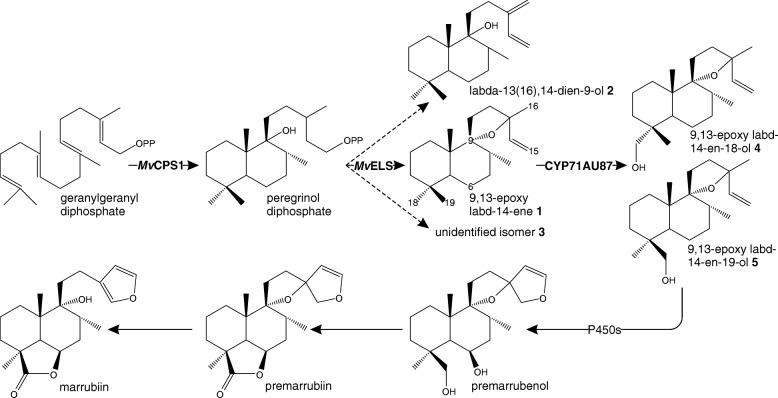
Proposed marrubiin-biosynthetic pathway. *Mv*CPS1 and *Mv*ELS form a diterpene synthase pair that converts the central precursor geranylgeranyl diphosphate (GGPP) into 9,13-epoxy labd-14-ene **1** (and possibly additional closely related products including labda-13(16),14-dien-9-ol **2**) via the prenyl diphosphate intermediate peregrinol diphosphate. Cytochrome P450 monooxygenases are hypothesized to catalyze position-specific functional modifications to yield premarrubiin possibly via the CYP71AU87 product 9,13-epoxy labd-14-en-19-ol **5** and other pathway intermediates such as premarrubenol previously identified in *M. vulgare* tissues. Subsequent lactone formation would form premarrubiin, from which marrubiin will derive through enzymatic or spontaneous ring opening to yield the free hydroxyl group at C-9. Dashed lines represent enzyme products identified in co-expression assays of *Mv*CPS1 and *Mv*ELS in this study

Toward elucidating the marrubiin-biosynthetic pathway in *M. vulgare*, we previously reported on the identification of gene candidates for terpenoid biosynthesis in *M. vulgare* using a genomics-based gene discovery approach [16, 23]. A functional pair of diterpene synthases (diTPSs), designated PEREGRINOL DIPHOSPHATE SYNTHASE (*Mv*CPS1) and 9,13-EPOXY-LABD-14-ENE SYNTHASE (*Mv*ELS), were identified that catalyze the first committed reactions in marrubiin biosynthesis [16]. *Mv*CPS1 transforms the central diterpenoid precursor geranylgeranyl diphosphate (GGPP) into peregrinol diphosphate, a bicyclic prenyl diphosphate intermediate that features a hydroxy group as C-9 characteristically present in marrubiin and related metabolites (Fig. 1). In a sequential reaction facilitated by *Mv*ELS, the diphosphate moiety is ionized and the resulting carbocation undergoes rearrangement to form 9,13-epoxy-14-labd-ene [16]. Downstream of this intermediate, several oxidative reactions catalyzed by members of the large family of cytochrome P450 monooxygenases (P450s) presumably facilitate the functional decoration of the diterpenoid scaffold to yield marrubiin and structurally similar bioactive diterpenoids (Fig. 1). Specifically, hydroxylation or carboxylation at C-6 and C-19 would have the potential to form the characteristic γ-lactone ring structure, and hydroxylation at C-16 and/or C-15 would facilitate formation of the furan ring.

In related Lamiaceae species, members of the CYP71 and CYP76 families of the P450 superfamily have been shown to catalyze position-specific oxygenation reactions in diterpenoid metabolism [24–28]. For example, CYP76AH1 and CYP76AH4 from *Salvia miltiorrhiza* and rosemary (*Rosmarinus officinalis*) catalyze oxygenation at C-11 and/or C-12 of abietatriene en route to ferruginol and downstream tanshinone diterpenoids [29, 30]. Similarly, CYP76AH24, CYP71BE52 and CYP76AK6/8, were shown to facilitate hydroxylation and carboxylation at C-12, C-2 or C-20, respectively, of related labdane scaffolds [26, 31]. Although plant P450s directly involved in the formation of diterpenoid lactones have not yet been reported, in rice (*Oryza sativa*), CYP99A3 and CYP76M8 were demonstrated to catalyze C-19 carboxylation and C-6 hydroxylation respectively as a prerequisite for lactone ring formation in bioactive momilactone diterpenoids [32, 33]. Additionally, P450s involved in furan ring formation have been reported, including CYP71A32 from peppermint (*Mentha x piperita*) involved in the biosynthesis of the monoterpene menthofuran [34], and CYP76BK1 from *Vitex agnus-castus* hydroxylates peregrinol at C-16 potentially en route to furan ring closure in the biosynthesis of diterpenoids with structural similarity to marrubiin [18].

In this study, we combined the interrogation of an established leaf transcriptome inventory with phylogenetic and gene expression analyses to identify the CYP71 family member, *Mv*1270 (CYP71AU87), as a probable candidate for a function in marrubiin biosynthesis. Co-expression of CYP71AU87 with *Mv*CPS1 and *Mv*ELS in *Nicotiana benthamiana* and yeast (*Saccharomyces cerevisiae*) resulted in the oxygenation of the *Mv*ELS product, 9,13-epoxy-labd-14-ene, at position C-18 or C-19 as verified by GC-MS and NMR analysis. Although these isomeric diterpene alcohols could not be identified *in planta* using GC-MS and LC-MS analysis, high expression levels of *CYP71AU87* transcript in marrubiin-accumulating tissues of *M. vulgare* supports a possible role in marrubiin biosynthesis. Thus, the discovery of CYP71AU87 provides new resources toward elucidating and metabolic pathway engineering of the production of marrubiin and related natural products.

## Results

### Transcriptomics-enabled identification of P450 genes with possible roles in marrubiin biosynthesis

**Fig. 2:**
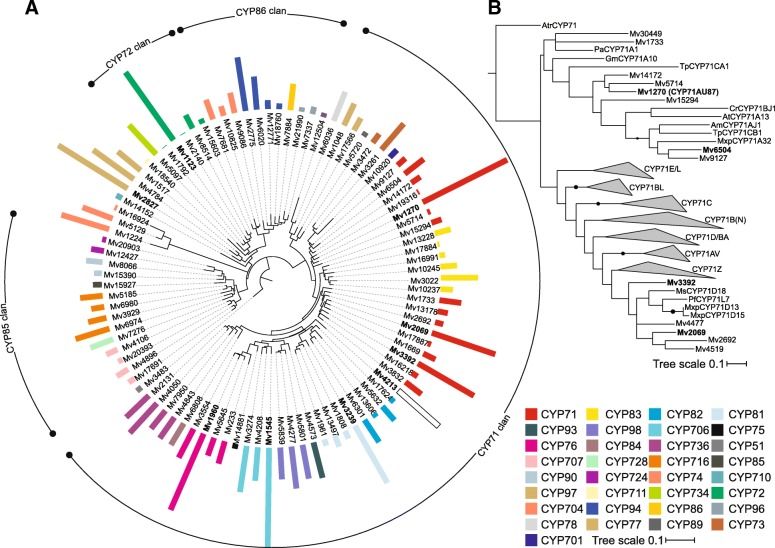
Identification of P450 candidates in the *Marrubium vulgare* leaf transcriptome. **a** Maximum-likelihood phylogenetic tree of P450 candidates (Additional file 2: Table S1) identified in the *M. vulgare* leaf transcriptome [23]. Bars illustrate transcript abundance of full-length P450 candidates as based on calculated FPKM (Fragments Per Kilobase of transcript per Million mapped reads) values, and color-coded to represent different CYP families. **b** Maximum-likelihood phylogenetic tree of select *M. vulgare* CYP71 family candidates as compared to functionally characterized CYP71 enzymes. A CYP71 family member of the ancestral dicot *Amborella trichopoda* was used to root the tree. Black dots highlight bootstrap support of ≥75% (1000 repetitions). Mv, *Marrubium vulgare*; Tp, *Tanacetum parthenium*; Gm, *Glycine max*; Pa, *Persea americana*; At, *Arabidopsis thaliana*; Cr, *Catharanthus roseus*; Am, *Ammi majus*; Mxp, *Mentha x piperita*, Atr, *Amborella trichopoda*

Previous studies demonstrated that marrubiin and related diterpenoid metabolites accumulate predominantly in leaves and leaf trichomes of *M. vulgare* [16, 17]. To identify P450 candidates with possible functions in marrubiin biosynthesis, we mined an established *M. vulgare* leaf transcriptome [23] against a P450-specific protein database (Additional file 1: Data file S1). Applying a minimal sequence length of 960 bp and an *E*-value cut-off of ≤1*E*^− 50^, a total of 95 candidate genes with significant matches to known P450 enzymes were identified (Additional file 2: Table S1). Phylogenetic analysis placed these P450 candidates into 33 P450 families with members of the CYP71 family being the most represented (Fig. 2). To further triage high-probability P450 candidates, gene expression levels were assessed on the basis of FPKM (Fragments Per Kilobase of transcript per Million mapped reads) values obtained by mapping the raw reads against the assembled *M. vulgare* leaf transcriptome. Highest transcript abundance was observed for members of the CYP71 (*Mv*1270, *Mv*2069, *Mv*3392, *Mv*4213), CYP72 (*Mv*1123), CYP76 (*Mv*1960), CYP81 (*Mv*3239), CYP706 (*Mv*1545), and CYP97 (*Mv*2827) families (Fig. 2). Since members of the CYP71 and CYP76 families have been demonstrated to function in diterpenoid metabolism in Lamiaceae and other plant species [26, 27, 30, 31, 35], *Mv*1270, *Mv*3392 and *Mv*4213 were pursued for functional studies. Despite extensive efforts, *Mv*2069 and the CYP76 candidate *Mv*1960 could not be cloned successfully, preventing further analysis. Phylogenetic analysis placed *Mv*1270, albeit distantly, into a branch that also contained known terpenoid-metabolic enzymes of the CYP71 family, such as the menthofuran synthase CYP71A32 from *Mentha x piperita* [34] and *Tanacetum parthenium* CYP71CA1 and CYP71CB1 involved in costunolide biosynthesis [36] (Fig. 2). Another lower abundant P450 assigned to the CYP71 family, *Mv*6504, was also located on this branch and showed a close phylogenetic relationship to CYP71A32 and was selected for functional analysis, due to a possibly similar activity in furan ring formation. *Mv*3392 was placed into a different branch distantly related to members of the CYP71D subfamily with roles in monoterpenoid biosynthesis in species of mint. In addition, *Mv*1545 was chosen as a candidate enzyme, since it represented the most abundant transcript of all identified P450s and was assigned to the CYP706 family with known activities in sesquiterpenoid metabolism [37, 38]. Similarly, *Mv*4213 was selected for further study, due to its high transcript abundance and only distant relationship to other *M. vulgare* P450 candidates (Fig. 2). The remaining abundant P450 transcripts assigned to the CYP72, CYP97 and CYP81 families were not further investigated in this study, since members of these families more commonly function in brassinosteroid, carotenoid or phenylpropanoid metabolism as demonstrated in other plant species [27, 39].

### Clarification of reaction products derived from the combined activity of MvCPS1 and MvELS

**Fig. 3:**
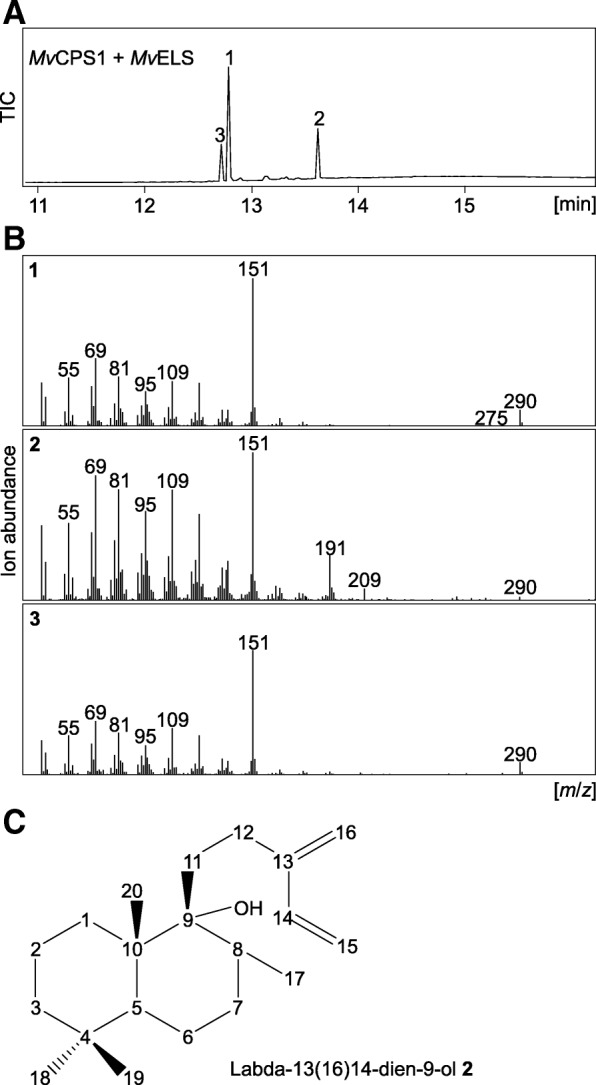
Analysis of reaction products of the pairwise activity of *Mv*CPS1 and *Mv*ELS. **a** Total ion chromatogram of reactions products derived from co-expression assays of *Mv*CPS1 and *Mv*ELS in *N. benthamiana*. Compound **1**, 9,13-epoxy labd-14-ene; compound **2** [16], labda-13(16),14-dien-9-ol (as verified by NMR analysis); compound **3**, unidentified diterpenoid closely related to 9,13-epoxy labd-14-ene. **b** Mass spectra of compounds **1**, **2**, and **3**. **c** Structure of labda-13(16),14-dien-9-ol as verified by NMR analysis (see Additional file 3: Figure S1 for details)

Our prior work on the in vitro pairwise reaction *Mv*ELS showed three reaction products (compounds **1–3**) [16]. The major product (compound **1**) was identified as 9,13-epoxy labd-14-ene, whereas low abundance of the remaining products prevented structural analysis [16]. Compound **3** likely represents an isomer of 9,13-epoxy labd-14-ene as based on a near identical retention time and fragmentation pattern. Conversely, the mass spectrum of compound **2** showed additional abundant ions of *m*/*z* 191 and 209, indicative of a more distinct structure (Fig. 3). To clarify the product profile of *Mv*ELS, we performed large-scale co-expression assays of *Mv*CPS1 and *Mv*ELS with a GGPP synthase from *Abies grandis* in an *E. coli* platform engineered for diterpenoid production [40]. Purification of compound **3** remained unsuccessful, due to its co-elution with compound **1**. In contrast, purification of compound **2** using an optimized protocol combining silica chromatography and semi-preparative HPLC analysis enabled 1D ^1^H-NMR and ^13^C-NMR, as well as 2D NMR experiments: COSY, HSQC, HMBC and H2BC (Additional file 3: Figure S1). This approach identified compound **2** as labda-13(16),14-dien-9-ol, which features a free hydroxyl group at C-9 (1C, 77.24 ppm) as compared to the 9,13-spiroether (1C, 93.8 ppm) observed in 9,13-epoxy labd-14-ene (Fig. 3 and Additional file 3: Figure S1) [16]. This was evidenced by the presence of two double bonds between C-13 (1C, 147.54 ppm) and C-16 (1C, 115.43 ppm) as well as between C-14 (1C, 138.88 ppm) and C-15 (1C, 113.35 ppm) in compound **2**, whereas compound **1** showed one double bond between C-14 (1C, 147.2 ppm) and C-15 (1C, 110.7 ppm), but not between C-13 (1C, 84.6 ppm) and C-16 (1C, 23.5 ppm) (Additional file 3: Figure S1) [16].

### Biochemical characterization of CYP71AU87 as a 9,13-epoxy labd-14-ene hydroxylase

**Fig. 4:**
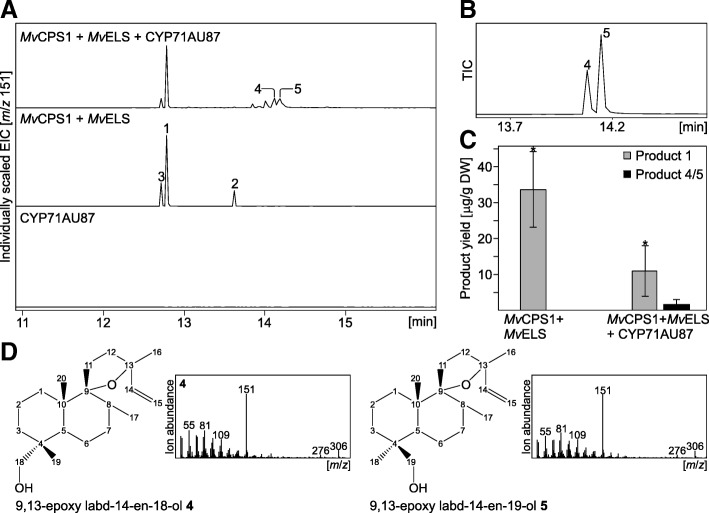
Functional characterization of CYP71AU87. **a** Extracted ion chromatograms (*m*/*z* 151) of enzyme products resulting from *Agrobacterium*-mediated transient co-expression of *Mv*CPS1, *Mv*ELS, and CYP71AU87 in *N. benthamiana*. Compound **1**, 9,13-epoxy labd-14-ene; compound **2** [16], labda-13(16),14-dien-9-ol; compound **3**, unidentified diterpenoid closely related to 9,13-epoxy labd-14-ene; compound **4**, 9,13-epoxy labd-14-ene-18-ol (as verified by NMR analysis); compound **5**, 9,13-epoxy labd-14-ene-19-ol (as verified by NMR analysis). **b** GC-MS total ion chromatogram (TIC) of the purified CYP71AU87 products **4** and **5** obtained via metabolite extraction from ~ 50 *N. benthamiana* leaves transfected with *Mv*CPS1, *Mv*ELS and CYP71AU87. **c** GC-MS-based quantification of 9,13-epoxy labd-14-ene **1** and 9,13-epoxy labd-14-ene-18/19-ol **4/5** (combined amounts) extracted from single-leaves of *N. benthamiana* co-transformed with *Mv*CPS1, *Mv*ELS and CYP71AU87. Co-expression assays performed in triplicate with error bars denoting one standard deviation and * denoting a *P*-value < 0.05 using Tukey’s test. **d** GC-MS mass spectra and structures of 9,13-epoxy labd-14-ene-18-ol and 9,13-epoxy labd-14-ene-19-ol as based on NMR analyses (see Additional file 5: Figure S3 for details on NMR analyses)

Next, we probed the activity of the selected P450 candidates in converting the reaction products of *Mv*ELS. The full-length sequences of *Mv*1270, *Mv*3392, *Mv*4213, *Mv*1545, and *Mv*6504 were amplified from cDNA prepared from *M. vulgare* leaf RNA, and cloned into the pLIFE expression vector for use in established *Agrobacterium*-mediated transient co-expression assays in *Nicotiana benthamiana* [16, 23]. P450 activity was then tested via co-expression of each P450 candidate with *Mv*CPS1 and *Mv*ELS.As compared to compounds **1**–**3** produced by the sequential activity of *Mv*CPS1 and *Mv*ELS alone, no additional diterpenoid products were detected when co-expressing *Mv*1545, *Mv*4213, *Mv*6504 or *Mv*3392 (Additional file 4: Figure S2). Co-expression of the CYP71 family member *Mv*1270 (hereafter designated CYP71AU87) with *Mv*CPS1 and *Mv*ELS resulted in the formation of two products (compounds **4** and **5**) with retention times of 14.12 min and 14.19 min, respectively, indicating two closely related structures of higher polarity than the diTPS products (Fig. 4). Both products showed near identical fragmentation patterns that featured the dominant *m/z* 151 mass ion characteristic for the premarrubiin scaffold, as well as an additional mass ion of *m/z* 306 indicative of the presence of an additional oxygen atom (Fig. 4). Although only low product quantities were observed, CYP71AU87-catalyzed formation of compounds **4** and **5** was accompanied by a near complete turnover of labda-13(16),14-dien-9-ol **2** and a 3-fold decrease in the abundance of 9,13-epoxy labd-14-ene **1** as compared to the activity of *Mv*CPS1 and *Mv*ELS only (Fig. 4). This reduction in substrate abundance was largely consistent with the formation of **4** and **5** up to 4 μg g^− 1^ DW in transfected *N. benthamiana* leaves (Fig. 4).

To determine the structure of the CYP71AU87 products, ~ 1 mg of compounds **4** and **5** were extracted from 150 g of agroinfiltrated *N. benthamiana* leaf tissue, followed by purification using silica chromatography and semi-preparative HPLC analysis (Fig. 4). Although a separation of both compounds could not be achieved, the mixture of products **4** and **5** was successfully isolated with > 90% purity. Combining a suite of 1D and 2D NMR analyses identified the products as 9,13-epoxy labd-14-ene-18-ol and 9,13-epoxy labd-14-ene-19-ol (Fig. 4 and Additional file 5: Figure S3). Importantly, chemical shifts of C-9 (13C, 92.8 ppm) and C-13 (13C, 83.7) showed the presence of the 9,13-epoxy group rather than a free C-9 hydroxyl function in both CYP71AU87 products (Additional file 5: Figure S3). In addition, the NMR analysis demonstrated that hydroxylation of the CYP71AU87 occurred at C-18 or C-19, whereas presence of the methyl groups at C-20, C-17 and C-16 could be verified by means of HSQC and HMBC correlations (Additional file 5: Figure S3). Additional HMBC correlation analysis between C-18 and C-19 supported the identity of compounds **4** and **5** as an isomeric pair featuring hydroxyl groups at C-18 or C-19 (Additional file 5: Figure S3). As a definite assignment of which structure represents compounds **4** and **5** was not possible on the basis of these analyses, 9,13-epoxy labd-14-ene-18-ol and 9,13-epoxy labd-14-ene-19-ol were tentatively assigned as compounds **4** and **5**, respectively. Additional transient *N. benthamiana* co-expression assays of *Mv*CPS1, *Mv*ELS and CYP71AU87 with *Mv*3392, *Mv*4213, *Mv*1545 or *Mv*6504 did not result in any new reaction products that would indicate the ability of these P450 candidates in utilizing compounds **4** and **5** rather than the *Mv*ELS products as substrates (Additional file 4: Figure S2).

To further validate the activity of CYP71AU87 in *N. benthamiana* co-expression assays, we next performed co-expression of *Mv*CPS1, *Mv*ELS, a *M. vulgare* cytochrome P450 reductase (*Mv*CPR) and CYP71AU87 in yeast (*Saccharomyces cerevisiae*). For this purpose, the constructs pESC-HIS:*Mv*CPS/*Sc*BTS1, pESC-Trp:*Mv*ELS:/*Mv*CPR and pESC-Ura:CYP71AU87 were generated to enable the co-expression of *Mv*CPS1, *Mv*ELS, *Mv*CPR, CYP71AU87 and the endogenous yeast GGPP synthase BTS1 in the yeast strain AM94 that is engineered for enhanced diterpenoid production [41]. GC-MS analysis of the resulting enzyme products confirmed the production of compounds **4** and **5**, although with relatively low product abundance (Additional file 6: Figure S4). Notably, yeast expression of *Mv*CPS1 alone or in combination with *Mv*ELS resulted in the formation of two additional compounds, **6** and **7**, which represent the *Mv*CPS1 product peregrinol **6** (i.e. dephosphorylated peregrinol diphosphate) [16] and likely a degradation product thereof **7**, indicating a lower catalytic activity of *Mv*CPS1, *Mv*ELS and/or CYP71AU87 as compared to co-expression assays in *N. benthamiana*. This observation is consistent with the presence of unconverted GGPP **8** substrate in the metabolite extract.

### CYP71AU87 exhibits substrate specificity for 9,13-epoxy labd-14-ene and labda-13(16),14-dien-9-ol

**Fig. 5:**
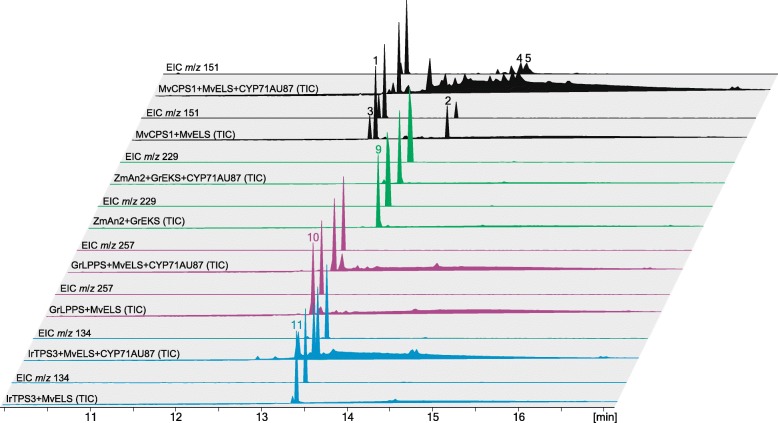
Substrate-specificity of CYP71AU87. Shown are total ion chromatograms (TIC) and extracted ion chromatograms (EIC, individual mass ions) of reaction products resulting from transient *N. benthamiana* co-expression assays of CYP71AU87 with different class II and class diTPSs that produce distinct diterpenoid scaffolds. Tested products included the gibberellin precursor *ent*-kaurene **9** formed by the maize (*Zea mays*) *ent*-CPP synthase *Zm*AN2 and the *ent*-kaurene synthase *Gr*EKS from *Grindelia robusta* [45], manoyl oxide **10** produced by the LPP synthase *Gr*LPPS from *G. robusta* [23] and *Mv*ELS, and miltiradiene **11** formed by the (+)-CPP synthase *Ir*TPS3 from *Isodon rubescens* [44] and *Mv*ELS. Co-expression of *Mv*CPS1, *Mv*ELS and CYP71AU87 was used as a positive control. Mass spectra and structures of the formed diterpenoids are given in Additional file 7: Figure S5

Our earlier studies demonstrated that *Mv*ELS is a multi-functional diTPS that not only produces 9,13-epoxy labd-14-ene as a major product, but also accepts the class II diTPS products (+)-copalyl diphosphate (CPP) and labda-13-en-8-ol diphosphate (LPP) to form miltiradiene and manoyl oxide, respectively [16]. While no LPP synthase has thus far been identified in this species, *M. vulgare* does contain a functional (+)-CPP synthase (*Mv*CPS3) [16]. Therefore, we probed the possible substrate promiscuity of CYP71AU87 for different labdane diterpenoid intermediates. This was achieved by taking advantage of the natural modular nature of labdane diterpenoid biosynthesis, where diTPSs and P450s from different species can be combined to form active pathways toward different products [10, 42, 43]. Transient *N. benthamiana* co-expression assays were performed by combining CYP71AU87 and *Mv*ELS with different class II diTPSs, including a LPP synthase from *Grindelia robusta* (*Gr*LPPS) [23] to produce manoyl oxide (compound **10**), and a (+)-CPP synthase from *Isodon rubescens* (*Ir*TPS3) [44] to form miltiradiene (compound **11**) (Fig. 5). In addition, transient *N. benthamiana* co-expression assays with maize (*Zea mays*) *ent*-CPP synthase *Zm*AN2 [45] and the *G. robusta ent*-kaurene synthase *Gr*EKS [23] were used to test the ability of CYP71AU87 to convert the gibberellin precursor *ent*-kaurene (compound **9**). Co-expression of CYP71AU87 with *Mv*CPS1 and *Mv*ELS served as a positive control. Co-expression of CYP71AU87 and the diTPS combinations *Mv*ELS/*Gr*LPPS, *Mv*ELS/*Ir*TPS3 or *Zm*AN2/*Gr*EKS resulted in the expected biosynthesis of manoyl oxide, miltiradiene and *ent*-kaurene, respectively (Fig. 5 and Additional file 7: Figure S5). However, no additional products indicative of a CYP71AU87-catalyzed conversion of these diterpenoids were detected.

### Analysis of CYP71AU87 products in planta

**Fig. 6:**
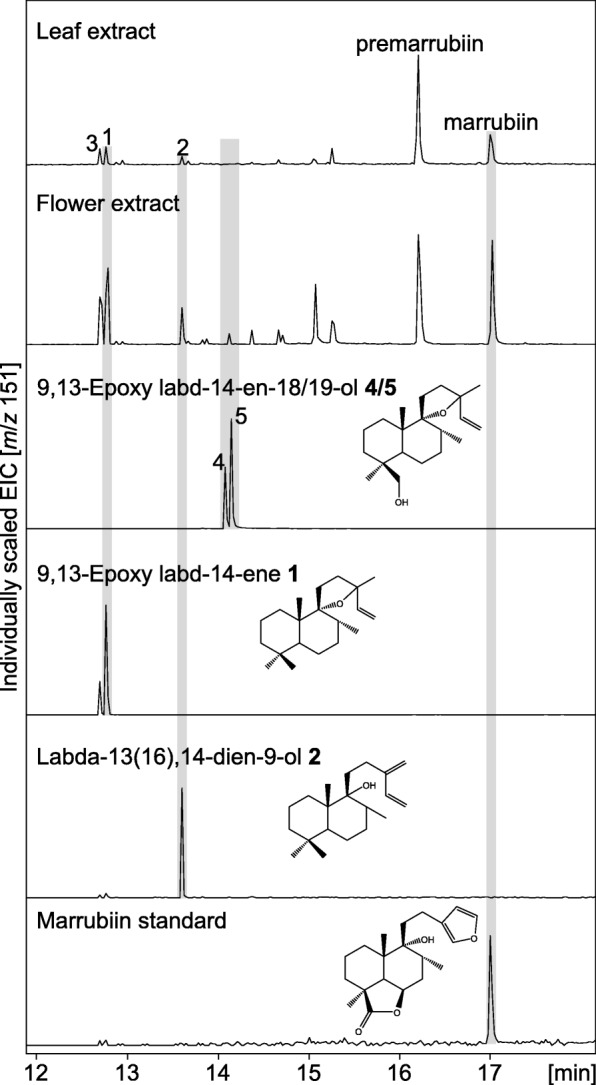
Analysis of *Mv*ELS and CYP71AU87 products *in planta*. Extracted ion chromatograms (*m*/*z* 151) resulting from GC-MS analysis of metabolite extracts from *M. vulgare* leaf and flower tissues as compared to purified standards of 9,13-epoxy labd-14-ene **1**, labda-13(16),14-dien-9-ol **2**, 9,13-epoxy labd-14-ene-18-ol **4**, and 9,13-epoxy labd-14-ene-19-ol **5**, and marrubiin. Diterpenoids were extracted from 12-week-old plants using hexane and detected using electron ionization (EI) GC-MS analysis

To investigate if the CYP71AU87 products 9,13-epoxy labd-14-ene-18-ol **4** and 9,13-epoxy labd-14-ene-19-ol **5** are present *in planta*, metabolite extracts prepared from *M. vulgare* leaves and flowers were analyzed via GC-MS, since these tissues had been shown to be most abundant in premarrubiin, marrubiin and related diterpenoids [16]. This approach identified marrubiin, premarrubiin, 9,13-epoxy labd-14-ene **1** and labda-13(16),14-dien-9-ol **2** as well as the unidentified *Mv*ELS product **3** in both tissues, whereas neither of the P450 products were detectable (Fig. 6). To verify the results obtained by GC-MS analysis, metabolite extracts were further analyzed using UPLC-Q Exactive MS analysis (Additional file 8: Figure S6). While the purified CYP71AU87 products could be detected as ammonium adducts with a parental ion of *m*/*z* 324.2893 (monoisotopic mass [M]^+^ *m*/*z* 306.2559) using this approach, neither 9,13-epoxy labd-14-ene-18-ol nor 9,13-epoxy labd-14-ene-19-ol were detectable, thus confirming the results derived from GC-MS analysis (Additional file 8: Figure S6).

### CYP71AU87 transcript is most abundant in *M. vulgare* leaves and flowers

**Fig. 7:**
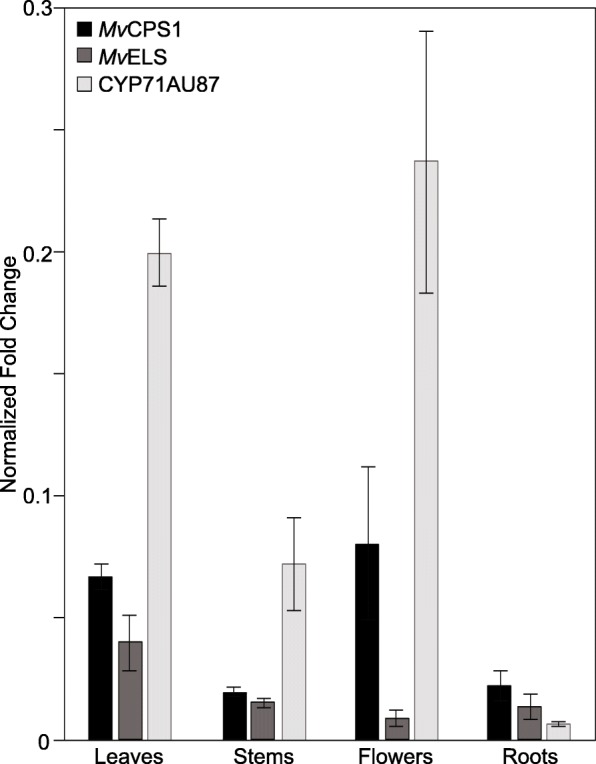
Relative Transcript abundance of *CYP71AU87* in *M. vulgare* tissues. Transcript abundance was measured by qPCR using gene-specific oligonucleotides (Additional file 9: Table S2) and normalization to the Elongation Factor 1α (*EF1α*) from *M. vulgare* as a reference. Error bars represent standard errors based on triplicate measurements of three biological replicates. Gene expression was calculated on the basis of efficiency-corrected oligonucleotides, and the reaction specificity was verified by dissociation curves and sequence verification of representative products

To further investigate a possible role of *CYP71AU87* in the biosynthesis of marrubiin or related diterpenoids, we next carried out primer efficiency-corrected quantitative PCR (qPCR) analysis of *CYP71AU87*, *MvCPS1* and *MvELS* in leaves, stems, flowers and roots of 12-week-old *M. vulgare* plants. Consistent with the predominant abundance of marrubiin [16, 17], transcript abundance of *MvELS* was highest in leaves and also present in stems, flowers and roots, albeit at overall low levels (Fig. 7). Similarly, *MvCPS1* gene expression was low in stems and roots, and highest in leaves and flowers. Transcript abundance of *CYP71AU87* also was highest in leaves and flowers as the primary marrubiin-accumulating tissues, but relatively lower in stems and detectable at only trace levels in roots. Notably, gene expression of *CYP71AU87* was 3.7- and 5.3-fold higher in leaves and flowers, respectively, as compared to *MvCPS1* and *MvELS*.

## Discussion

Marrubiin and related diterpenoids of *M. vulgare* are of potential medicinal value for their anti-diabetic and vasorelaxant properties [13, 15]. Further drug development relies on the availability of viable means for producing these metabolites. Isolation of bioactive compounds, including marrubiin, from plant biomass is often insufficient for meeting this need, due to the typically complex chemical profile of the natural producers, limited plant cultivation, and necessary protection of endemic wild species. Although multi-step synthetic routes for marrubiin and select derivatives thereof have been reported, the inherent stereochemical complexity of terpenoid natural products also hampers chemical synthesis approaches [46, 47]. With rapid advances in gene and enzyme discovery, reconstruction of pathways utilizing genes from the same or even different species has become an alternative strategy for plant natural product manufacture as exemplified by the microbial manufacture of the anti-malaria drug artemisinin [48]. Toward realizing such metabolic engineering approaches for marrubiin and related furanoterpenoid lactones, knowledge of the biosynthesis of the characteristic furan and lactone groups is of particular interest, since these functional modifications likely contribute to the therapeutic activities, as has been shown for the psychotropic compound salvinorin A, the anti-inflammatory diterpenoid andrographolide, and the anti-malarial agent artemisinin [49–52].

The presented study illustrates the utility of combining genomics-enabled gene discovery and multi-gene co-expression analyses to identify previously hidden functions among the diverse P450 superfamily. Characterization of CYP71AU87 adds an additional catalyst to the group of terpenoid-metabolic members of the CYP71 family. In a sequential reaction with the diTPS pair, *Mv*CPS1 and *Mv*ELS, CYP71AU87 forms the isomeric products 9,13-epoxy labd-14-ene-18-ol and 9,13-epoxy labd-14-ene-19-ol, as verified by GC-MS and NMR analysis (Fig. 4). Oxidation at C-18 and C-19 distinguishes CYP71AU87 from other members of the CYP71 family that function in labdane diterpenoid metabolism, including CYP71BE52 from *R. officinalis* and *Salvia pomifera* that catalyze the hydroxylation of ferruginol at C-2 [26, 31], rice CYP71Z6 and CYP71Z7 that facilitate C-2 oxygenation of *ent*-isokaurene and *ent-*cassadiene in oryzalide and phytocassane biosynthesis [53], and CYP71Z16/18 that controls the sequential oxygenation of dolabradiene at C-16 and C-3 to form dolabralexins in maize [54]. However, rice CYP99A2/A3, members of the CYP99 sub-family that belongs to the larger CYP71 family, have been shown to catalyze the sequential oxygenation at C-19 of *syn-*pimaradiene [55, 56]. In addition, oxygenation at C-18 and C-19 of labdane diterpenes has been shown for other P450 families, such as members of the CYP701A family relevant for the oxidation of *ent*-kaurene in gibberellin biosynthesis [35] and the gymnosperm-specific CYP720 family of diterpene resin acids metabolism [57, 58]. However, unlike the CYP71AU87-catalyzed hydroxylation, these enzymes facilitate a step-wise oxidation of their respective substrates to form the corresponding acids. Thus, P450-catalyzed oxidation of the labdane scaffold at C-18 or C-19 likely evolved independently several times with further species-specific diversification of catalytic specificity.

Co-expression assays in *N. benthamiana* and yeast showed that, while CYP71AU87 is specific to converting the C-9-oxygenated *Mv*ELS products within the scope of substrates tested here (Fig. 5), the enzyme shows promiscuity toward hydroxylating the C-18 or C-19 position (Fig. 4). As these products were not detected *in planta* (Fig. 6), it is not discernable if formation of this isomeric product pair is the native function of CYP71AU87. Marrubiin and all described structurally related diterpenoids are functionalized at C-19, supporting a regio-specific biosynthetic reaction at this position [15]. Notably, a similar catalytic pattern has been described for CYP720B1 from *Pinus taeda* that forms C-18- and C-19-hydroxylated isomers of miltiradiene when co-expressed in *S. cerevisiae* [59], whereas the close homolog CYP720B4 from Sitka spruce (*Picea sitchensis*) yields the regio-selective product oxygenated at C-18 [57]. C-19-oxidized diterpene acids, such as communic acids and isocupressic acid in species of the genus *Juniperus* and other coniferous trees [60, 61], have also been observed, but the underlying metabolic enzymes are unknown. Against this background, it remains to be clarified whether CYP71AU87 forms both, the C-18- and C-19-hydroxylated products, in *M. vulgare* or if more efficient metabolite channeling *in planta* may result in the formation of marrubiin from only the C-19 isomer.

In a previous study, biochemical characterization of *Mv*ELS illustrated three products, two of which represented 9,13-epoxy labd-14-ene **1** and an unidentified but closely related isomer thereof **3** as based on a near identical mass spectrum [16]. Here, NMR analysis identified the third product as labda-13(16),14-dien-9-ol **2**, which features a free hydroxy group at C-9 rather than the 9,13-spiroether function present in 9,13-epoxy labd-14-ene (Fig. 1). In the course of this study labda-13(16),14-dien-9-ol was also reported as a product of the diTPS pair *Vac*TPS1 and *Vac*TPS6 from *Vitex agnus-castus* [18], a Lamiaceae species related to the genus *Marrubium* that produces related C-9-oxygenated diterpenoid lactones. Earlier studies suggested that the characteristic free C-9 hydroxyl group in marrubiin is formed non-enzymatically upon metabolite extraction from plant material [17, 62]. This hypothesis is consistent with the observation that the CYP71AU87 products exclusively occurred in the 9,13-spiroether form (Fig. 4). However, if the opening of the 9,13 epoxide and possible ring re-formation occurs spontaneously or enzymatically remains to be verified, given the presence of both, labda-13(16),14-dien-9-ol and 9,13-epoxy labd-14-ene, in extracts from *M. vulgare* (Fig. 6) and similar metabolite profiles observed in related species [19].

Although direct evidence that C-18/19 hydroxylation of 9,13-epoxy labd-14-ene is a native function of CYP71AU87 in *M. vulgare* would require further genetic studies, co-occurrence of the *MvCPS1*, *MvELS* and *CYP71AU87* transcripts in tissues where premarrubiin and the *Mv*ELS products are also present (Fig. 6) suggest that CYP71AU87 would readily encounter 9,13-epoxy labd-14-ene as a substrate. Substrate specificity for the *Mv*ELS products further support a role of CYP71AU87 in the biosynthesis of marrubiin or related diterpenoids (Fig. 5). Absence of the CYP71AU87 products *in planta* could be attributed to a rapid conversion of these functionalized diterpenoids in *M. vulgare*, as has similarly been proposed for other P450s for which enzyme products were not detectable in plant tissues, such as *Thapsia garganica* CYP76AE2 with a probable function in thapsigargin biosynthesis [63] and tomato CYP71BN1 that catalyzes the formation of the diterpenoid lycosantalonol [64].

CYP71AU87-catalyzed formation of especially 9,13-epoxy labd-14-ene-19-ol then represents a possible reaction step in the biosynthesis of the 19,6-lactone ring structure characteristic of premarrubiin and other diterpenoids present in the genus *Marrubium* (Fig. 1). Presence of marrubenol and premarrubenol, bioactive diterpenoids featuring hydroxyl groups at both C-19 and C-6b, in several species of *Marrubium* supports a sequential hydroxylation reaction toward lactone formation [15, 65, 66] (Fig. 1). Conceptually similar pathways have been described for the biosynthesis of costunolide sesquiterpenoids via sequential oxidation of C-12 and hydroxylation at C-6 of germacrene A facilitated by a pair of P450 enzymes [20, 67]. Alternatively, dehydrogenases or reductases could function in further oxidizing the C-19 hydroxyl group in 9,13-epoxy labd-14-ene-19-ol and, combined with P450-catalyzed oxygenation at C-6b, result in lactone ring formation as similarly proposed for the rice diterpenoid momilactone A and the sesquiterpenoid artemisinin [33, 68, 69]. Formation of premarrubiin and related structures further requires formation of a furan ring via additional P450-enabled oxidation C-15 and/or C-16 and subsequent ring closure (Fig. 1). Although the sequence of lactone and furan ring formation in marrubiin biosynthesis is unresolved, a P450, CYP76BK1 from *V. agnus-castus*, that catalyzes the C-16-hydroxylation of peregrinol (a dephosphorylated derivative of peregrinol diphosphate) toward the predicted vitexilactone precursor labd-13*Z*-ene-9,15,16-triol has indeed recently been identified [18].

## Conclusions

The functional characterization of CYP71AU87 exemplifies the utility of combining gene-specific query of transcriptome data with multi-enzyme co-expression assays to identify natural product pathway genes in non-model plant systems. CYP71AU87 adds an additional catalyst to the catalog of diterpenoid-biosynthetic P450s and provides enzyme resources for producing marrubiin and related bioactive diterpenoid lactones.

## Methods

### Plant material

Seeds of *Marrubium vulgare* were purchased from Baker Creek Heirloom Seeds. Seeds of *M. vulgare* as well as *Nicotiana benthamiana* were germinated in Conviron TCR120 growth chambers (www.conviron.com) under a photoperiod of 16 h, 60% relative humidity, 100 μmol m^− 2^ s^− 1^ light intensity, and a day/night temperature cycle of 21/18 °C. After two weeks, *M. vulgare* plants were maintained in university greenhouses for an additional 12 weeks unless otherwise stated.

### Phylogenetic and sequence analysis

Protein sequence alignments were generated using clustalW2 and curated with Gblocks [70]. Maximum likelihood phylogenetic analyses were performed using PhyML-aBayes version 3.0.1 beta with four rate substitution categories, LG substitution model, BIONJ starting tree and 1000 bootstrap repetitions [71]. Phylogenies were visualized using Interactive Tree of Life (iTOL) v4.3 [72].

### Mapping of transcriptome data to candidate P450 genes

Leaf-specific transcriptome resources for *M. vulgare* were described earlier [23]. P450 candidates were identified by querying this transcriptome inventory against a custom P450 protein database (Additional file 1: Data file S1). Relative transcript abundance of P450 candidates was assessed by mapping adapter-trimmed Illumina reads back to the assembled P450 transcripts using BWA version 0.5.9-r16. Reads were mapped as paired with maximum insert size of 350 bp with a threshold of one allowed mismatch at the alignment step.

### Gene cloning

Total leaf RNA of 12-week old *M. vulgare* plants was converted to cDNA using the SuperScript III First-Strand Synthesis System (ThermoFisher). The *CYP71AU87* gene then was amplified using Phusion-HF polymerase (New England Biolabs) and gene-specific oligonucleotides (Additional file 9: Table S2) and ligated into the pJET vector (Clontech) for sequence verification. The resulting amplicon was inserted into the pLIFE and pESC:Ura vectors for expression in *N. benthamiana* and *S. cerevisiae*, respectively. The *Mv*CPR gene was cloned in the same manner and ultimately inserted into the pESC:Trp vector for expression in *S. cerevisiae*. *Mv*CPS1 was cloned into the first multiple cloning site of the pESC:His vector containing the yeast GGPP synthase BTS1. *Mv*ELS was cloned into the first multiple cloning site of a pESC:Trp vector already containing *Mv*CPR in the second multiple cloning site.

### GC-MS analysis of terpenoid metabolites

Extracts were prepared from ~ 150 mg fresh tissue from leaves, stems, flowers and roots of 12-week old *M. vulgare* plants. Tissues were ground to a fine powder in liquid N_2_ and terpenoids extracted with 1.5 ml hexane for 16 h under vigorous shaking at room temperature. Extracts were freed from residual water by addition of anhydrous Na_2_SO_4_, dried under N_2_ stream, and re-dissolved in hexane for further analysis. GC-MS analysis was performed on an Agilent 7890B GC interfaced with a 5977 Extractor XL MS Detector at 70 eV and 1.2 ml min^− 1^ He flow, using a HP5-ms column (30 m, 250 μm i.d., 0.25 μm film) and the following GC parameters: 50 °C for 1 min, 20 °C min^− 1^ to 300 °C, hold 3 min with pulsed splitless injection at 250 °C and 50 °C oven temperature. MS data from 90 to 600 mass-to-charge ratio (*m*/*z*) were collected after a 4 min solvent delay. Identification of marrubiin was achieved by comparison to the authentic standard (www.chromadex.com).

### LC-MS analysis of terpenoid metabolites

For LC-MS analysis, dried samples were re-dissolved in 300 μl acetonitrile:water (80:20; *v*/v). All measurements were carried out on a Q Exactive HF mass spectrometer coupled with a Vanquish LC system (Thermo Scientific). A sample volume of 5 μl were separated on a Waters Acquity UPLC CSH C_18_ column (100 × 2.1 mm; 1.7 μm) coupled to an Acquity UPLC CSH C_18_ VanGuard precolumn (5 × 2.1 mm; 1.7 μm). The column was maintained at 65 °C at a flow rate of 0.6 ml min^− 1^. The mobile phases consisted of A: acetonitrile:water (60:40, v/v) with ammonium formate (10 mM) and formic acid (0.1%) and B: 2-propanol:acetonitrile (90:10, v/v) with ammonium formate (10 mM) and formic acid (0.1%). The 15 min separation was conducted under the following gradient: 0 min 15% B; 0–2 min 30% B; 2–2.5 min 48% B;2.5–11 min 82% B; 11–11.5 min 99% B; 11.5–12 min 99% B; 12–12.1 min 15% B; 12.1–15 min 15% B. Orbitrap MS instrument was operated in electrospray ionization (ESI) in positive mode with the following parameters: mass range 60–900 *m*/*z*; spray voltage 3.6 kV, sheath gas (nitrogen) flow rate 60 units; auxiliary gas (nitrogen) flow rate 25 units, capillary temperature 320 °C, full scan MS1 mass resolving power 120,000, data-dependent MSMS (dd-MSMS) 4 scans per cycle, dd-MSMS mass resolving power 30,000. Thermo Xcalibur 4.0.27.19 was used for data acquisition and analysis [73].

### Transient expression in *Nicotiana benthamiana*

Full-length cDNA clones of *Mv*CPS1, *Mv*ELS and *CYP71AU87* in the pLIFE expression vector were individually transformed into *A. tumefaciens* strain GV3101. Bacterial cultures were grown at 28 °C in Luria-Bertani (LB) medium supplemented with 50 mg l^− 1^ kanamycin, pelleted and resuspended to a final OD_600_ of 1.0 in 10 mM MES buffer with 10 mM MgCl_2_. Following incubation for two hours at 22 °C and gentle shaking, cell suspensions were mixed and syringe-infiltrated into the underside of the leaves of 5-week-old *N. benthamiana* plants. For all assays, target genes were further co-expressed with the plant viral protein p19 to suppress RNA silencing. [23] Transfected plants were maintained for four days before metabolites were extracted with 1.5 ml hexane from a single transformed leaf and analyzed via GC/LC-MS as described above. Quantification of metabolites via GC-MS was performed using an external standard curve using the structurally related diterpenoid sclareol as a standard. Statistically significant differences between metabolite levels across tobacco co-expression assays was calculated based on Welch Two Sample *t*-test (*P*-value < 0.05). Quantification of metabolites via LC-MS in tobacco co-expression assays was calculated using sclareol as internal standard at a concentration of 1 μg ml^− 1^ using the Thermo Xcalibur 4.0.27.19 software.

### Enzyme co-expression in engineered yeast

The generated constructs pESC-HIS:*Mv*CPS/*Sc*BTS1, pESC-Trp:*Mv*ELS:/*Mv*CPR and pESC-Ura:*CYP71AU87* were co-transformed *Saccharomyces cerevisiae* strain AM94 that was specifically engineered for diterpenoid production [59, 74]. Cells were grown in 50 ml of the corresponding selective dropout medium (−His, −Trp, −Leu, −Ura, 2% dextrose) at 30 °C to an OD_600_ of ∼0.6, followed by transfer of cells into 50 ml of corresponding selective dropout media with 2% galactose for induction. After 48 h, metabolites were extracted by vortexing the cell pellets with glass beads in 5 ml of diethyl ether, air-dried and re-suspended in 1 ml hexane for GC-MS analysis.

### Nuclear magnetic resonance (NMR) analysis of the *CYP71AU87* product

Approximately 1 mg of the *CYP71AU87* product was extracted from ~ 50 *N. benthamiana* leaves transfected with *Mv*CPS1, *Mv*ELS and *CYP71AU87* using 400 ml hexane. Extracts were air-dried and purified through iterative chromatography on silica matrix (230–400 Mesh, Grade 60) using an ethyl acetate-hexane gradient as mobile phase (100% hexane, 10% ethyl acetate, and 20% ethyl acetate; *v*/v). Product-containing fractions were pooled, dried under N_2_ stream, and resuspended in 100% acetonitrile. Further purification was achieved by reverse-phase HPLC on an Agilent 1100 series HPLC equipped with an Agilent Zorbax Eclipse Plus C_8_ column, G1315B diode array detector (DAD) and G1364C-1260 FC-AS fraction collector. The mobile phases consisted of a mixture of A: water and B: acetonitrile. Product separation was conducted using the following gradient: 0 min 50% B; 0–7 min 50% B; 7–10 min 75% B; 10–20 min 90% B; 20–40 min 100% B. Purified products were pooled and resuspended in chloroform-D spiked with 0.03% Tetramethylsilane (TMS) as internal standard (Sigma). For structural identification of the *CYP71AU87* products 1D (^1^H, ^13^C, NOE) and 2D (COSY, HSQC, HMBC, selective HSQC, selective HMBC) spectra were collected on Brucker Avance 800 MHz spectrometer equipped with a 5 mm CPTCI cryoprobe.

### Quantitative PCR (qPCR)

Total RNA was isolated as previously described using approximately 100 mg tissue [75]. RNA integrity and concentration was measured using Bioanalyzer 2100 RNA Nano chip assays (Agilent) following the manufacturer’s protocol. Equal RNA amounts were used for cDNA synthesis with the Superscript III reverse transcriptase (ThermoFisher) and oligo(dT) primers. The subsequent qPCR reaction was performed on a Bio-Rad CFX96 Real-time system using the SsoFast kit (www.bio-rad.com) and target-specific oligonucleotides (Additional file 9: Table S2). Relative transcript abundance was calculated using efficiency corrected ΔCT using the amplification efficiency values (E-values) generated from primer efficiency calculations for each gene pair. Relative gene expression values were calculated based on *M. vulgare* Elongation Factor 1α as reference gene and triplicate measurements with three biological replicates. Target specificity was confirmed by sequence verification of representative amplicons.

## Additional files

Additional file 1: **Data file 1.** P450 database used in this study. (DOCX 164 kb)

Additional file 2: **Table S1.** List of transcript sequences of P450 candidate genes identified in the established *Marrubium vulgare* leaf transcriptome. (XLSX 79 kb)

Additional file 3: **Figure S1.** NMR analysis of labda-13(16),14-dien-9-ol (compound 2) formed by the coupled reaction of *Mv*CPS1 and *Mv*ELS. (PDF 910 kb)

Additional file 4: **Figure S2.** GC-MS analysis of reaction products derived from *Nicotiana benthamiana* co-expression assays of *Mv*CPS1 and *Mv*ELS the P450 candidates *Mv*1270, *Mv*1545, *Mv*4213, *Mv*6504, and *Mv*3392, respectively. Shown are extracted ion chromatograms (*m*/*z* 151) of the diterpenoid products 9,13-epoxy labd-14-ene 1, labda-13(16),14-dien-9-ol 2, unidentified diterpene 3, 9,13-epoxy labd-14-ene-18-ol 4, and 9,13-epoxy labd-14-ene-19-ol 5. The suppressor of RNA silencing p19 was included in all co-expression assays. (A) Co-expression of *Mv*CPS1, *Mv*ELS and *Mv*CYP71AU87, (B) Co-expression of *Mv*CPS1 and *Mv*ELS, (C-F) Co-expression of *Mv*CPS1 and *Mv*ELS with the P450 *Mv*1545, *Mv*4213, *Mv*6504, and *Mv*3392, respectively), (G-J) Co-expression of *Mv*CPS1 and *Mv*ELS with *Mv*CYP71AU87 and the individual P450 *Mv*1545, *Mv*4213, *Mv*6504, and *Mv*3392, respectively. (K) Expression of *Mv*CYP71AU87 only. (PDF 264 kb)

Additional file 5: **Figure S3.** NMR analysis of 9,13-epoxy-labd-14-ene-19-ol (compounds 4/5) formed by the coupled reaction of *Mv*CPS1, *Mv*ELS and CYP71AU87. (PDF 1415 kb)

Additional file 6: **Figure S4.** Co-expression of *Mv*CPS1, *Mv*ELS and CYP71AU87 in yeast (*Saccharomyces cerevisiae*). Shown are extracted ion chromatograms (*m*/*z* 151) and mass spectra of the reaction products resulting from co-expression of *Mv*CPS1, *Mv*ELS, CYP71AU87, *Mv*CPR and the endogenous yeast GGPP synthase BTS1 in the yeast strain AM94. 9,13-epoxy labd-14-ene 1, labda-13(16),14-dien-9-ol 2, 9,13-epoxy labd-14-ene-18-ol 4, 9,13-epoxy labd-14-ene-19-ol 5, peregrinol (i.e. dephosphorylated peregrinol diphosphate) 6, unidentified compounds 7, geranylgeraniol (i.e. dephosphorylated GGPP) 8. (PDF 451 kb)

Additional file 7: **Figure S5.** Mass spectra and chemical structures for diterpenoids observed in *N. benthamiana* co-expression assays to test CYP71AU87 substrate promiscuity (see Fig. 6). Transient *N. benthamiana* co-expression assays were performed by combining *Mv*CYP71AU87 and *Mv*ELS with different class II diTPSs, including the maize (*Zea mays*) *ent*-CPP synthase *Zm*AN2 to produce the gibberellin precursor *ent*-kaurene (compound 9), a LPP synthase from *Grindelia robusta* (*Gr*LPPS) to produce manoyl oxide (compound 10), and a (+)-CPP synthase from *Isodon rubescens* (*Ir*TPS3) to form miltiradiene (compound 11). (PDF 322 kb)

Additional file 8: **Figure S6.** LC-MS analysis of CYP71AU87 products in *M. vulgare* leaves and flowers. (A) Leaf and flower tissue from 12-week-old *M. vulgare* plants was extracted with 20% (*v*/v) ethyl acetate in hexane, and then dried and re-suspended in MeOH. Extracts were analyzed by LC-MS on a Q Exactive HF mass spectrometer coupled with a Vanquish LC system (Thermo Scientific). A volume of 5 μl was separated on a Waters Acquity UPLC CSH C_18_ column (100 × 2.1 mm; 1.7 μm) coupled to an Acquity UPLC CSH C_18_ VanGuard precolumn (5 × 2.1 mm; 1.7 μm) at 65 °C and a flow rate of 0.6 ml min^− 1^. The mobile phases consisted of A: acetonitrile:water (60:40, v/v) with ammonium formate (10 mM) and formic acid (0.1%) and B: 2-propanol:acetonitrile (90:10, v/v) with ammonium formate (10 mM) and formic acid (0.1%). The 15 min separation was conducted under the following gradient: 0 min 15% B; 0–2 min 30% B; 2–2.5 min 48% B;2.5–11 min 82% B; 11–11.5 min 99% B; 11.5–12 min 99% B; 12–12.1 min 15% B; 12.1–15 min 15% B. Orbitrap MS instrument was operated in electrospray ionization (ESI) in positive mode with the following parameters: mass range 60–900 *m*/*z*; spray voltage 3.6 kV, sheath gas (nitrogen) flow rate 60 units; auxiliary gas (nitrogen) flow rate 25 units, capillary temperature 320 °C, full scan MS1 mass resolving power 120,000, data-dependent MSMS (dd-MSMS) 4 scans per cycle, dd-MSMS mass resolving power 30,000. Thermo Xcalibur 4.0.27.19 was used for data acquisition and analysis. (B) Structures for CYP71AU87 products. (C) CYP71AU87 products were detectable in ESI(+) MS mode, annotated with accurate mass (< 5 ppm) with a retention time of 2.21 min and a parental mass ion of 324.2893 consistent with the addition of an ammonium ion (monoisotopic mass of the CYP71AU87 products 4/5 = 306.2559). (PDF 205 kb)

Additional file 9: **Table S2.** Oligonucleotides used in this study. (PDF 111 kb)

## Abbreviations

CPP: Copalyl diphosphate
DiTPS: Diterpene synthase
GC-MS: Gas chromatography mass spectroscopy
GGPP: Geranylgeranyl diphosphate
LC-MS: Liquid chromatography mass spectroscopy
NMR: Nuclear magnetic resonance
P450: Cytochrome P450 monooxygenase
