# Supplementary material for: Functional characterization of the cytochrome P450 monooxygenase CYP71AU87 indicates a role in marrubiin biosynthesis in the medicinal plant Marrubium vulgare
Source: BMC Plant Biol. 2019 Mar 25;19:114. doi: 10.1186/s12870-019-1702-5 (PMC6434833; doi:10.1186/s12870-019-1702-5)
Supplement: Supplementary file 1 — Data file 1. P450 database used in this study. (DOCX 164 kb) [file 12870_2019_1702_MOESM1_ESM.docx]

| **Additional File 1:** Data file 1. P450 database used in this study. | |  |
| --- | --- | --- |
| **Protein ID** | **Sequence** | |
| CYP51G | MDLPPELAVLADKVLSLSPVVLVALGSAVLILALAVGRVLFNLLPSKRPPVWEGLPFIGGLLKFTGGPWKLLENGYAKFGECFTVPVAHRRVTFLIGPEVSPHFFKAGDDEMSQSEVYDFNIPTFGRGVVFDVEQKVRTEQFRMFTEALTKNRLKSYVPHFNKEAEEYFAKWGETGVVDFKDEFSKLITLTAARTLLGREVREQLFDEVADLLHGLDEGMVPLSVFFPYAPIPVHFKRDRCRKDLAAIFAKIIRARRESGRREEDVLQQFIDARYQNVNGGRALTEEEITGLLIAVLFAGQHTSSITTSWTGIFMAANKEHYNKAAEEQQDIIRKFGNELSFETLSEMEVLHRNITEALRMHPPLLLVMRYAKKPFSVTTSTGKSYVIPKGDVVAASPNFSHMLPQCFNNPKAYDPDRFAPPREEQNKPYAFIGFGAGRHACIGQNFAYLQIKSIWSVLLRNFEFELLDPVPEADYESMVIGPKPCRVRYTRRKL | |
| CYP51G1 | MELDSENKLLKTGLVIVATLVIAKLIFSFFTSDSKKKRLPPTLKAWPPLVGSLIKFLKGPIIMLREEYPKLGSVFTVNLVHKKITFLIGPEVSAHFFKASESDLSQQEVYQFNVPTFGPGVVFDVDYSVRQEQFRFFTEALRVNKLKGYVDMMVTEAEDYFSKWGESGEVDIKVELERLIILTASRCLLGREVRDQLFDDVSALFHDLDNGMLPISVLFPYLPIPAHRRRDRAREKLSEIFAKIIGSRKRSGKTENDMLQCFIESKYKDGRQTTESEVTGLLIAALFAGQHTSSITSTWTGAYLMRYKEYFSAALDEQKNLIAKHGDKIDHDILSEMDVLYRCIKEALRLHPPLIMLMRASHSDFSVTARDGKTYDIPKGHIVATSPAFANRLPHIFKDPDTYDPERFSPGREEDKAAGAFSYIAFGGGRHGCLGEPFAYLQIKAIWSHLLRNFELELVSPFPEIDWNAMVVGVKGNVMVRYKRRQLS | |
| CYP55B1 | MAPQHDFPFSRPKGVEPPAEYKELRSKCPVAPGRLFDGSKIWLISRHKELKEVLQDGRFSKVRTLPGFPELSPGGKAAAQSGNAATFVDMDPPEHTKYRGMVWPYLTPEAVEQLRPSIQAKADKLVDAMIARGGPLDLNEAFSMPLPFRVIYDFIGIPEADFAYLSANVAVRSSGSSNAKDAAAAADDLVKYMDNLVAEKERNPTGKDLISELVTKQLRPGHMTREQLVQTAFLMLVAGNATVATQINLGVISLLQHPDQLAAMKADPARLVPAATEEICRFHTGSSYALRRLAVADVQVDGQLVKKGEGIIALNQSANRDESVFPDPDRFDIHRQSNPQQVGFGYGTHVCVAEWLARAEIQVAIGTLFRRLPNLRLAVPESQIQYSDPARDVGLAALPVTW | |
| CYP701A3 | MAFFSMISILLGFVISSFIFIFFFKKLLSFSRKNMSEVSTLPSVPVVPGFPVIGNLLQLKEKKPHKTFTRWSEIYGPIYSIKMGSSSLIVLNSTETAKEAMVTRFSSISTRKLSNALTVLTCDKSMVATSDYDDFHKLVKRCLLNGLLGANAQKRKRHYRDALIENVSSKLHAHARDHPQEPVNFRAIFEHELFGVALKQAFGKDVESIYVKELGVTLSKDEIFKVLVHDMMEGAIDVDWRDFFPYLKWIPNKSFEARIQQKHKRRLAVMNALIQDRLKQNGSESDDDCYLNFLMSEAKTLTKEQIAILVWETIIETADTTLVTTEWAIYELAKHPSVQDRLCKEIQNVCGGEKFKEEQLSQVPYLNGVFHETLRKYSPAPLVPIRYAHEDTQIGGYHVPAGSEIAINIYGCNMDKKRWERPEDWWPERFLDDGKYETSDLHKTMAFGAGKRVCAGALQASLMAGIAIGRLVQEFEWKLRDGEEENVDTYGLTSQKLYPLMAIINPRRS | |
| CYP701B1 | MLNESTSGHSSDTCVQTSLGCRDGKRRLNEMLETKVIAHHVSHSPCAAIPGGLPVLGNLLQLTEKKPHRTFTAWSKEHGPIFTIKVGSVPQAVVNNSEIAKEVLVTKFASISKRQMPMALRVLTRDKTMVAMSDYGEEHRMLKKLVMTNLLGPTQVHDHRVQQNPPCLKMCHVYASHSKGTPEEKIVCSPAFYRRISPSGMEFGCAEQKPIVEVLELGTCVSTWDMFDALVVAPLSAVINVDWRDFFPALRWIPNRSVEDLVRTVDFKRNSIMKGLIRAQRMRLANLKEPPRCYADIALTEATHLTEKQLEMSLWEPIIESADTTLVTSEWAMYEIAKNPDCQDRLYREIVSVAGTERMVTEDDLPNMPYLGAIIKETLRKYTPVPLIPSRFVEEDITLGGYDIPKGYQILVNLFAIANDPAVWSNPEKWDPERMLANKKVDMGFRDFSLMPFGAGKRMCAGITQAMFIIPMNVAALVQHCEWRLSPQEISNINNKIEDVVYLTTHKLSPLSCEATPRISHRLP | |
| CYP701C1v2 | VAEVPGLPFVGNLLQMTVERPHRKLTSWSNEYGPIYTIRTGQKSQVIVSSPELAREAVVAKYSSISNRDLGSNLTILTRNRKIVAMSDYGDRYRMLKRMVVNNLLGQTSQKALHVQRENYLRIALDGLFDELGRFPGSTGQVNARDCIANFLFRLGMHQASVFGRDIESVRVPELGAEVTRWEIYRVLVQDVMKAAVQIDWRDFFPSLKWIPNRKFEDGIYKVERKRSAVTKALMEQHRQLSRSQQRDKCYCDVLLDNESHYSEDELLLAAWEPIIESSDTTLVTSEWALYELASGPKLQEKLYNEIKRVVGDERMVSEDDLPNLPFLNAVIKETLRKYSPVPILPPRYIHEQVELGGYTIPAGYQLIVNIFGIHHDPKRWSNPETWDPSRFLGVEGGSFDMGLTDMRLMPFGGGKRICAGMAQVFYVVPMIIATLVQHFEWTLPQGDMDKRNVVEDTVYLTTQKLEPLQACAKPRVPRRLPSKTLNAVPSNNKVPEHKH | |
| CYP702A1 | MVEVYELLTVMVSLIVVKLFHWIYQSKNPKPNEKLPPGSMGFPIIGETFEFMKPHDAFQFPTFIKERIIRYGPIFRTSLFGAKVIISTDIELNMEIAKTNHAPGLTKSIAQLFGENNLFFQSKESHKHVRNLTFQLLGSQGLKLSVMQDIDLLTRTHMEEGARRGCLDVKEISSKILIECLAKKVTGDMEPEAAKELALCWRCFPSGWFRFPLNLPGTGVYKMMKARKRMLHLLKETILKKRASGEELGEFFKIIFEGAETMSVDNAIEYIYTLFLLANETTPRILAATIKLISDNPKVMKELHREHEGIVRGKTEKETSITWEEYKSMTFTQMVINESLRITSTAPTVFRIFDHEFQVGSYKIPAGWIFMGYPNNHFNPKTYDDPLVFNPWRWEGKDLGAIVSRTYIPFGAGSRQCVGAEFAKLQMAIFIHHLSRDRWSMKIGTTILRNFVLMFPNGCEVQFLKDTEVDNSSGSNPDCCNG | |
| CYP703A2 | MILVLASLFAVLILNVLLWRWLKASACKAQRLPPGPPRLPILGNLLQLGPLPHRDLASLCDKYGPLVYLRLGNVDAITTNDPDTIREILLRQDDVFSSRPKTLAAVHLAYGCGDVALAPMGPHWKRMRRICMEHLLTTKRLESFTTQRAEEARYLIRDVFKRSETGKPINLKEVLGAFSMNNVTRMLLGKQFFGPGSLVSPKEAQEFLHITHKLFWLLGVIYLGDYLPFWRWVDPSGCEKEMRDVEKRVDEFHTKIIDEHRRAKLEDEDKNGDMDFVDVLLSLPGENGKAHMEDVEIKALIQDMIAAATDTSAVTNEWAMAEAIKQPRVMRKIQEELDNVVGSNRMVDESDLVHLNYLRCVVRETFRMHPAGPFLIPHESVRATTINGYYIPAKTRVFINTHGLGRNTKIWDDVEDFRPERHWPVEGSGRVEISHGPDFKILPFSAGKRKCPGAPLGVTMVLMALARLFHCFEWSSPGNIDTVEVYGMTMPKAKPLRAIAKPRLAAHLYT | |
| CYP703B1 | MNILSPELLVPLITEWIQGGRLIFATCSVLVALLSSVFLVAHFRTPMNLPPGPKAMPLLGNLLQMGSHPHRTMTAMHKKYGHILYIRLGCIPTVVVDSPQLIAEITKEQDNVFSSRPHMTFTDIVAYDAHDFAMAPYGPHWRYVRRICVHELLTPKRLEITMKERIEESRCMIMAVAEAAQKGEIVDMRDVFAGVSMTVMCRMLLGRREFAATGKKAKDFKHLIHELFRLMGALNLRDFVPALGWLDLQGFERDMYKLRDEFDEVFDAVIQEHRDLASGKLPGGKPNDFISVLLDLPGENGAPHLDDKTIKAITPDMMAGATDTSAVTNEWAMAEIIRNTEIQRKLQEEIDSVVGLERNVQESDINKLPYLMCVVKETFRLHPAGPFAIPRETMADTKLSGYRIPKGTRVLINIFSLGRSSETWKDPLKFQPERWANENLSAIHDMGFRILPFGYGRRQCPGYNLGTTMVLLTLARLLHGFKWSFPPGVTAENIDMEELYGCTTPLRTRLRTIATPRLAPHLYSQ | |
| CYP703C1v2 | MQLGPLPHRTMAGWCQKYGPLVYCRLGSTPTITASSPQMIRELLWTQDETFASRPRTTAGKLMAYHDQDVGLAPYDAHWKLMRRICVENLLTTRRMEGFQKSRAEEARDLVETMLKEAKAGNTINIREHLGGFTMNVITRMLIGKRYFGTESTGEKEATDIRELLHEAVALIGVFFIPDHVPLLKWMDPKSYRHQFKKIGKRMDDYYSYIIEEHRKRQEVVDGPKDFVDVLLGENSELNDVEIKALIQDMVVGGTDTASFTMEWFMLEMIRNPKVMRKVQKEIDSVLEKKATNKHLVEESDLASLDYLKAAVKETFRMHPVGGFLIPHESIRDTNVAGYHVPKGSLILINTHGLGRNSAVWDNVDEFRPERFLRTDDKVHLRDSEYRVIPFGSGKRACPGAQLGQSMLLLGLGRLFHGFDWYPPPGMSTGDIDVMEAYGLTTPPRTPLRAVAKARLDESFYCLH | |
| CYP703D1v1 | MDSSVFQAIVAAFFLFLSLIHFLFFRPIRPGAKSNVDPGVKGRDRRQRLHLPPGPKPWPVIGNLLQIGPFPHKSMMEFTRRHGPLVYLKLGVVPTIVTDSPAIIRDILIKQDHIFASRPENIACQYFTYNGRDIAFAPYGQHWRAMRKICTLELLSPRKIASFRDGRCQELDLMVESVFQDLGREEGSSPTTHKINLRDKFASLSCNILTRMLLGKRHFGPGAAGPEDAAEHKQMIYEGFALVNAFNVADYLPFLRAFDLQGHERKMRRIMQRADEVYDEIIEEHRQKLAKNSGGSCQEQQGASFVDVLLSVPGANGEKQLSTTTIKAIMIDMLSAGTDTSSVISEWAMAELARHPREMAKVREELDAAVGVDRPVDESDVVNLNYLKAVVKEIFRLHPVGAFLIPHFSTEDTRIGGYDIPKNTRILVNTYSLGRSRSVWGDDVHLFRPDRFLASPGDLSSQIVELMDSECRVVPFGAGRRSCPGASLGSCMVVMGLARLIQRFDWSAPPGEEIDVSERVGFTVLDKPLELVAKPRECVNF | |
| CYP703E1 | MVEIPQLLSSSTPELYLKLAVAGSLLVLLLLLLNLPSSRGARRKSSSSSSSSGSSSSSSPPLPPGPRGWPIIGNLLDVGTVPHEGMMKLTRAYGPLVYLRLGAIPHVVSDDPAIIKEFLKIQDHIFASRPGNVILAELLTYGGKDIGFAPYGAHWRNMRKICTLELFSAKSVDSFQRLRRMEMIHTLGLILDAAVDRRAVDLRDAFNGLTSNMMTRMLLGKRYFGPGDPGPEVGAELKAMIAEGILMMNGFNISDYLPFLRFLDLQGQERRMKQIMRHIDGLATALLLELAPRIGKKPESFVDILVNLRGENGEPHLPEDVMKAVMVDMMAAGTDTPGVSCEWAMAELLRDPALLARVREEVDRVVCVDRLVDESDLAHFRLLRAVLKESFRLHPVGAILIPHLAMEDAVVAGYGIPKDTRVLINVFALNRNAQVWERPHEFDPERHLRGLGEGAVVEFGDPECRLIPFGSGRRMCPAASLGLTMVLLALANLVHAFDWEVPANLSMERAPGKMVKAQALTALARPRLPRHLYSQQI | |
| CYP704A1 | MAIIVVTTIFILLSFALYLTIRIFTGKSRNDKRYTPVHATIFDLFFHSHKLYDYETEIARTKPTFRFLSPGQSEIFTADPRNVEHILKTRFHNYSKGPVGTVNLADLLGHGIFAVDGEKWKQQRKLVSFEFSTRVLRNFSYSVFRTSASKLVGFIAEFALSGKSFDFQDMLMKCTLDSIFKVGFGVELGCLDGFSKEGEEFMKAFDEGNGATSSRVTDPFWKLKCFLNIGSESRLKKSIAIIDKFVYSLITTKRKELSKEQNTSVREDILSKFLLESEKDPENMNDKYLRDIILNVMVAGKDTTAASLSWFLYMLCKNPLVQEKIVQEIRDVTSSHEKTTDVNGFIESVTEEALAQMQYLHAALSETMRLYPPVPEHMRCAENDDVLPDGHRVSKGDNIYYISYAMGRMTYIWGQDAEEFKPERWLKDGVFQPESQFKFISFHAGPRICIGKDFAYRQMKIVSMALLHFFRFKMADENSKVSYKKMLTLHVDGGLHLCAIPRTST | |
| CYP704B1 | MSLCLVIACMVTSWIFLHRWGQRNKSGPKTWPLVGAAIEQLTNFDRMHDWLVEYLYNSRTVVVPMPFTTYTYIADPINVEYVLKTNFSNYPKGETYHSYMEVLLGDGIFNSDGELWRKQRKTASFEFASKNLRDFSTVVFKEYSLKLFTILSQASFKEQQVDMQELLMRMTLDSICKVGFGVEIGTLAPELPENHFAKAFDTANIIVTLRFIDPLWKMKKFLNIGSEALLGKSIKVVNDFTYSVIRRRKAELLEAQISPTNNNNNNNNKVKHDILSRFIEISDDPDSKETEKSLRDIVLNFVIAGRDTTATTLTWAIYMIMMNENVAEKLYSELQELEKESAEATNTSLHQYDTEDFNSFNEKVTEFAGLLNYDSLGKLHYLHAVITETLRLYPAVPQDPKGVLEDDMLPNGTKVKAGGMVTYVPYSMGRMEYNWGSDAALFKPERWLKDGVFQNASPFKFTAFQAGPRICLGKDSAYLQMKMAMAILCRFYKFHLVPNHPVKYRMMTILSMAHGLKVTVSRRS | |
| CYP704C1 | MDVNILTMFVTVSALALACSLWIASYLRNWRKKGVYPPVVGTMLNHAINFERLHDYHTDQAQRYKTFRVVYPTCSYVFTTDPVNVEHILKTNFANYDKGTFNYDIMKDLLGDGIFNVDGDKWRQQRKLASSEFASKVLKDFSSGVFCNNAAKLANILAQAAKLNLSVEMQDLFMRSSLDSICKVVFGIDINSLSSSKAESGPEASFAKAFDVANAMVFHRHMVGSFWKVQRFFNVGSEAILRDNIKMVDDFLYKVIHFRRQEMFSAEKENVRPDILSRYIIISDKETDGKVSDKYLRDVILNFMVAARDTTAIALSWFIYMLCKHQHVQEKLLEEIISSTSVHEDQYSTECNDIASFAQSLTDEALGKMHYLHASLSETLRLYPALPVDGKYVVNEDTLPDGFKVKKGDSVNFLPYAMGRMSYLWGDDAKEFKPERWIQDGIFHPKSPFKFPAFQAGPRTCLGKDFAYLQMKIVAAVLVRFFKFEAVKTKEVRYRTMLTLHMNEDGLNVQVTPRLNSD | |
| CYP704D1 | MDFAQGWGSESIAGMMKTIVTAFSGILSLLLAYLLWAAADNWVLHRERKGPVQWPILGVTLEALKNYQTLNDWVVYYFLRDGLTFSCKMMHLDLTFTADPVNVKHILKTNFANYDKRKFFHENFEIFLGDGIFNVDGEIWRTQRKTASFEFASRKLRDFSTVVFRDYSVKLASILARAATAQQSMDMQDLFMRFTLDSIFKVTFDYDVGTLQPGLPNIPFAQAFEITNEITSSRLINPIWKLNRALKIGSERVLLQSAKDVDEFIYGVIEAKKAEMANSKSDLFSRFMRLEEDDSDIQFTDKNFRDTLLNFIIAGRDTTAVSLSWFVYRMAQNPEMTARLQQELRDFDTVRNWKQQPEGDEGLRRRVLGFAELLTFDNLVKLQYLHACILETLRLHPAVPQDPKHAINDDILSDGTQIKKGSLIYYTPYAMGRMPALWGPDAMEFNPQRWFVDGVVQTEQPFKFTAFQAGPRICLGKDSAMLQLRMVLALLYRFFTFQIVEGTDIRYRQMATLLLANGLPAKIIKQKN | |
| CYP704E1 | MLSGGPLETFSVWFAMNGESSFPVKTCLSLTWVTAESFLVAAIAWSFAAWIWWHWREQRKLPGPFAWPLIGCLPELSANWDRLHDWVLEQFSDDRRTIYVQFGYPDVAVFTVDPANVEHLLKTNFSNYPKGESNCNLMRELFGVGIFTTDGELWKEQRRMASYEFSSASLRDFSTDVFREYALKLVFILSRFASTGADFDLQEMCMRMTLGTTCKIGFGVVLDCLSPSLPKIQFAQCFDDANFISYHRFVDPLWHVKRALNIGRERKLKHCVKVLNTFTYNVIEKRRQEMASFNTKVWSWAAQSDLLSRLTDLCNRGGEISHYVDTALRDMILNFIVAGRDTTAGTLTWFFYMMSSHPEIADKIFDELSTVVAVAGKHIVEFSKLLTYEKLGKLHYLHAALSETLRLYPAVPLDSKQAAEDDVLPDGTVVKKGSMVGYVPYSMGRMKCLWGDYAAEFKPERWIQEGEFVPQSLFKFTAFQAGPRTCLGKDSAYLQMKMTAALVMRFFTIRVVPGHSMQYRTMLTLNMKHGLRAVVSRR | |
| CYP704F1 | MGDEGATLFGAFKSGNVLPAGVGQQEVWIMAAVSLVVVTASMWLWLLSLRRRPPGPMIWPWLGSMLEIAPQFDTMNDWYLNYFSADVKTFSFGMPGFPSCTKFVATVDPVIVEHILTNVYKYGKGDQLRDRLGDFLGRGIFLADGEDWRRHRKIASTEFSTRKLRGHSASVFRGEGVKLANCLKVAMAADQPVEIQDLFLRMTLDSICKVAFGVEIGSLSPDLPDVQFAKDFDNAQAHISKRVVRPMFKILRALDIGEEHHFRIATNSVHSFAMDVIAKRRKEIAAAHDAGEEYHRDDLLSKFMANLTQDENSYDDKELRDVIISFMLAGRDTTAVTLSWFTYEMCCHPEIADKIYEEGVAVIGKHTVVESAVEHLTHEALGQMHYLHAALSESLRLHPAVPRDGKCVLEEDVLPNGIKVKKGDFVQYVPYSMGRMPFLWGPDALEFKPERWLKDGVYQSVSPYIHSAFQAGPRICLGKDSAYLQLKVTAALITHFFKFHLVPGQEIAYTTTLVMPIKKGLKVTLSPRQ | |
| CYP704G1 | MDVLYTILTLIAFSLLAIFLAICFILMTIFKGKSIGDPKYAPVKGTVFNHLFYFNKLYDYQAQMAKIHPTYRLLAPNQSDQLYTIDVRNIEHVLKTNFDKYSRGKYSQDVMTDLFGEGIFAVDGDKWRQQRKVASYEFSTRVLRDFSCSVFRKNAAKLVRVISVFYHEGLVFDMQDLQMRCALDSIFKVGFGTELNCLEGSSKEGTEFMKAFDESNALIYWRYVDPIWNLKRFLNIGGEAKLKHNVKLIDDFVNGVINTKKEQLALQQDSNVKEDILSRFLMESKKGQTTITDKYLRDIILNFMIAGKDTTANTLSWFFYMLCKNPIVEDKIVQEIRDVTCFHESELSNIDEFATNLTDSILDKMHYLHAALTETLRLYPVVPVDGRTADAPDILPDGHKLQKGDAVNYMAYAMGRMSSIWGEDAEEFRPERWITDGIFQPESPFKFVAFHAGPRMCLGKDFAYRQMKIVAMCVLNFFKFKLANGTQNVTYKVMFTLHLDKGLPLHAIPRS | |
| CYP704H1v1 | MASKLFSFLPSSSSPWLWKWSSSSQSFSSGAWTWIITWALILAWWIFLHRFRQRGLRGPKSWPLVGCLFEQIANFDRLHHWLLDYHHKTWTFSAPVLGVNNTFTAHPANVEYILKTNFVNYPKGELLRQRFRDMMGYGIFNVDGEMWMHQRKVATVEFASSKLRDYSTFAFRDLTLKLAGILADRSGTGQALDLQDLFLRLTLDSICKIGFGVEIGCLRPDLPLIPFAHAFDYGNTLIIRRYIDMFWKIKRSFNAGSERELKRCIRVMDDFLYRVIERRRQELKQSKDVGRPDILSRFLSLDEEEAYTDKMLRDVVINFVIAGRDTTALTLSWLFSELAKRPEVVEKILAEVDRVFGVDEELEGKDSMSKKEQVLAKVANFSRKLDYQGLNRLHYLQATLTEALRLYPAVPLETKTVVADDVLPDGFSVKGGQFVSYSSWAMGRLEEIWGPDVLEFKPDRWLRSDNIFQPQSPFKLTAFQARREFGKAGPRICLGKDSAYLQMKITTILLLRFFKFELLDEKPVNYRMMVVLYMANGLLSRVSFR | |
| CYP704J1v1 | MNSTDEVRNISGVFEGLSYPNPLVFITGIAAAVFLAVLLSAEKRDHKGPKRWPLVGSYFQVVKNFPVLHDWFLSYFSSECRTIAVDWGTFYNILTVDPANVEHILKTNFANYPKGRVSHARNYDFMGDGIFNSDGEMWKRHRKLASYEFSSKKVNEYSGQVFRKAAVRMMEVLENIASKKTSFDFQDISMRMTLDSICEVAFGVELNTLSPSLPAVPFAASFDRVNELIVRRLIGPVWKILRALNLGSERELKNQIQVLDSFTFQVIENRRQEIEACEKSGKEYERQDLLSRFMTSTGASDAYHDRELRDAILNFIIAGRDTTAITLSWFIYCICNNPRVAKEIRLELDRTFGSESNTLTFSAFAQLLSTENLRTLHYLHACISETLRLYPPVPRDGKYAANDDVLPDGTKVKRGDSVAYVQYSMGRMEFLWGPDALEFKPERWIKNSEYQPQSPFVYTAFQAGPRICLGKDAAYLQAKITAAMLMRFFNFELVKDHVVHYRLLMVLAMVNGIKVNVSTL | |
| CYP705A1 | MDAIVVDSQNCFIIILLCSFSLISYFVFFKKPKVNFDLLPSPPSLPIIGHLHLLLSTLIHKSLQKLSSKYGPLLHLRIFNIPFILVSSDSLAYEIFRDHDVNVSSRGVGAIDESLAYGSSGFIQAPYGDYWKFMKKLIATKLLGPQPLVRSQDFRSEELERFYKRLFDKAMKKESVMIHKEASRFVNNSLYKMCTGRSFSVENNEVERIMELTADLGALSQKFFVSKMFRKLLEKLGISLFKTEIMVVSRRFSELVERILIEYEEKMDGHQGTQFMDALLAAYRDENTEYKITRSHIKSLLTEFFIGAADASSIAIQWAMADIINNREILEKLREEIDSVVGKTRLVQETDLPNLPYLQAVVKEGLRLHPPTPLVVREFQEGCEIGGFFVPKNTTLIVNSYAMMRDPDSWQDPDEFKPERFLASLSREEDKKEKILNFLPFGSGRRMCPGSNLGYIFVGTAIGMMVQCFDWEINGDKINMEEATGGFLITMAHPLTCTPIPLPRTQNSLISHL | |
| CYP706A1 | METASSNFSLSQILNIEEPYSSVMLGVAALLAVVCYFWIQGKSKSKNGPPLPPGPWPLPIVGNLPFLNSDVLHTQFQALTLKHGPLMKIHLGSKLAIVVSSPDMAREVLKTHDITFANHDLPEVGKINTYGGEDILWSPYGTHWRRLRKLCVMKMFTTPTLEASYSTRREETRQTIVHMSEMAREGSPVNLGEQIFLSIFNVVTRMMWGATVEGDERTSLGNELKTLISDISDIEGIQNYSDFFPLFSRFDFQGLVKQMKGHVKKLDLLFDRVMESHVKMVGKKSEEEEDFLQYLLRVKDDDEKAPLSMTHVKSLLMDMVLGGVDTSVNASEFAMAEIVSRPEVLNKIRLELDQVVGKDNIVEESHLPKLPYLQAVMKETLRLHPTLPLLVPHRNSETSVVAGYTVPKDSKIFINVWAIHRDPKNWDEPNEFKPERFLENSLDFNGGDFKYLPFGSGRRICAAINMAERLVLFNIASLLHSFDWKAPQGQKFEVEEKFGLVLKLKSPLVAIPVPRLSDPKLYTA | |
| CYP706B1 | MLQIAFSSYSWLLTASNQKDGMLFPVALSFLVAILGISLWHVWTIRKPKKDIAPLPPGPRGLPIVGYLPYLGTDNLHLVFTDLAAAYGPIYKLWLGNKLCVVISSAPLAKEVVRDNDITFSERDPPVCAKIITFGLNDIVFDSYSSPDWRMKRKVLVREMLSHSSIKACYGLRREQVLKGVQNVAQSAGKPIDFGETAFLTSINAMMSMLWGGKQGGERKGADVWGQFRDLITELMVILGKPNVSDIFPVLARFDIQGLEKEMTKIVNSFDKLFNSMIEERENFSNKLSKEDGNTETKDFLQLLLDLKQKNDSGISITMNQVKALLMDIVVGGTDTTSTMMEWTMAELIANPEAMKKVKQEIDDVVGSDGAVDETHLPKLRYLDAAVKETFRLHPPMPLLVPRCPGDSSNVGGYSVPKGTRVFLNIWCIQRDPQLWENPLEFKPERFLTDHEKLDYLGNDSRYMPFGSGRRMCAGVSLGEKMLYSSLAAMIHAYDWNLADGEENDLIGLFGIIMKKKKPLILVPTPRPSNLQHYMK | |
| CYP706C9 | MDAPGASTTTLLLYGALLLVGFLFIAVARRSNAGLPPGPTGLPLLGSLPSLDPQLHVYFARLAARYGPIFSIRLGSKLGVVVTSPELAREVLREQDLVFSGRDVPDAARSISYGGGQNIVWNPVGPTWRLLRRVCVREMLGPAGLDNVQGLRAREFGATLAHLHAQARAAAPVDVGAQMFLTVMNVITGTLWGGNVGSEGERVALGREFRHLVAEITDMLGAPNVSDFFPALARFDLQGIRKKSDALRERFNQMFARIIEQRVHAERAGGEPPAPDFLEYMLQLEKEGGDGKASFTMTNVKALLMDMVVGGTETTSNTVEWAMAELMQKPELLAKVRQELDAVVGRDAVVEESHLPQLPYLHAVVKETLRLHPALPLMVPHCPSADATVGGYRVPAGCRVFVNVWAIMRDPAVWKDPRDFVPERFLDGAGEGRKWDFTGSEMEYLPFGSGRRICAGVAMAQRMTAYSLAMLLQAFDWELPAGARLELDEKFAIVMKKATPLVAVPTPRLSKPELYYSA | |
| CYP706D1 | MSSSTICGPWSWFCKGDQDNEDILLPIILLAVSVTILGTCLFQWGFKKQRETADKLPPGPRGLPIVGYLPFLGPNLHQLFMELAQTYGPIYKLSIGRKLCVIISSPALVKEVVRDQDITFANRNPTIAAKTFSYGGKDIAFQPYGPEWRMLRKILLREMQSNANLDAFYSLRRNKVKESVNETYRKIGKPVNIGELAFSTVISMISGMFWGGTLEVDTEIDIGSEFRAAASELIEILGKPNVSDFFPVLARFDIQGIERKMKKATQRIEKIYDFVMDEWIEKGSARVESEAKNDQRKDFMHFLLGFKEQDSRRSISREQIKALLMDIVVGGTDTTSTTVEWAMAEMMLHPEVMKNAQKELTDAVGTDEIVEERHIDKLQFLHAVVKETLRLHPVAPLLLPRSPSNTCCVGGYTIPRNAKVFLNVWAIHRDPKFWDNPSEFQPERFLSDVSRLDYLGNNMQYLPFGSGRRICAGLPLGERMLMYCLATFLHMFKWELPNGERADTSEKFGVVLEKSTPLIAIPTPRLSNLNLYA | |
| CYP706E1 | MILVTDFKDKIYYTMINYWSWWWEVDNESDNVARTILTILVPILVLLWYKWTVSYTKKPRSRLPPGPYGLPVIGYLPFLSSNLHERFTEMSHKYGPIFSLYLGSKLNVVVNSIDLAKVVARDLDQTFANRNPPVTAITVTYGLLDIAWSNNNTHWRNTRKLLVSQVLSNANLDACQGFRTDEVRKTVGHVYAKIGEIVDINEISFETELNVVTNMLWGRNESGSLLEGFREVELKMLELLGAPNISDFIPMLSWFDLQGRKREMQKQHERLDRILDNVIKARMEGVLHDDGKKDFLQIMLELKDQKDGPTSLNMVQIKALLFDILTASTDTTSTMVEWVMAEILHNPDVMRRVQEELTIVIGMNNIVEESHLQKLVYLDAVVKETFRVHPPLPLLIQRCPNESFTVGGYTIPKGSIVYINVMAIHHDPKNWINPLEFKPERFLNGKWDYNGYNLKYLPFGSGRRICPGIPLGEKMLMYILASLLHSFEWSLPKEEELELSEEFGFVTKKRRPLIAIPSQRLPEASLYS | |
| CYP706F2 | MMLEGWSLWMEAEHTTIMVSILVATVTIFVGYKWLMKIAMRGKPPLPPGPRGLPLLGNLPFVEPDLHIYFSKLSKKFGPIFKIQMGTKIYVVINSASLARKVLKEDDEIFANRDPPAAAIAETYGGGNILWRPNGPEWRNLRKVLIREMMSKTYFDASYGLRRREVREMVKEIYAKVGSPIKVRNHMFLISLNVVMGMLWGAPLDEDKKHNVGLELVPFIEEAVDLLGKPNISDFFPILAPLDLQGIVSKMLKIRLRFDNIFESVIAIKKSTKTSNQSKDFLQILLELMQQEDEKMLFSMTNIKALLLDIISATTETSSTTVEWVMTELLKNSDIMKKVHEELERVVGNEKIVEECDINQLHYLQSVVKETMRLHPVGPLLIPHSPSVSTTIAGYTIPKGSSVFINVWSIMRDHETWKNPLKFQPERFFEHPEIGDYRGNNFNYLPFGSGRRICAGINLAEKMVMNVLATLLHSFDWKVENATNLNSSEKFGIVVRKLDPLMAIPTIRLPTLEQYY | |
| CYP706G1 | MISYHLNKATETICSLWSWWWAGSNKKLDVVAGAVLTVSVAVLAILWYLRTSRKGTAPWPPGPRGLPVVGYLPFLGSNLHHSFAELAHLYGPIFKLWLGNKLCVVLSSPSLAKQVVRDQDIIFANRDPPVAAFAYTYGGLDIAWSPYGSYWRNLRKVFVREMLSNTSLEACYPLQRSEVRKAITNAYSKIGTPMDIGQLSFQTVVNVTLSSLWGNTPEAHNDGKIGAEFREAASEITELLGKPNISDFFPTLAGFDIQGVERQMKRAFLSAEQVIDSIIDRKMKKSTAKEEGASDNGEKKDFLQFLLDLKVQEDTETPITMTQIKALLMDILVGGTDTTATMVEWVMAEMIRNPVIMTRAQEELTNVVGMGSIVEESHLPKLQYMDAVIKESLRLHPALPLLVPKCPSQDCTVGGYTIAKGTKVFLNVWAIHRDPQIWDSPSEFKPERFLSEPGRWDYTGNNFQYLPFGSGRRICAGIPLAERMIIYLLASLLHSFNWQLPEGEDLDLSEKFGIVLKKRTPLVAIPTKRLSSSDLYL | |
| CYP706H1 | MSSMVHRLLAMASSFWSQWCNIGYGGAGLFVSFAVIAVGAISWHILRKESLALPPGPRGMPVLGNLPFLHPDLHSCFAKMAQKYGPVMRLWLGNKLTVVLSSPSLAKEVLRDNDAIFADRDTPIAMLTMTYGGSGLIWARCDQNWRMLRKVWVGEMLSKVSLDRLYALRHREVWDSMRRIYANAGTSVNVNEHTFSAMINVITSMLWGRTLEGEERRHADKEFRQVVWEMFDLLTKPDVSDLFPVLAPLDIQGKNSKMKKLGSRLDRIFDFIINHHEVKGLSMEGIENKDREHKDLLQIFLQNEEGSKGILDKTQLKALFLDMVAAGTDTASSAVEWAMAELMNKPEKMERAQKELEQVVGMNNMVEETHLPKLPFLNAVVKEVLRLHPPGPFLVPRRTREPCVLRGYTIPSGTQVLVNAWAIHRDPEFWDSPSEFQPERFLSGSLKCDYSGNDFRYLPFGSGRRICAGVPLAERIVPHILASMLHLFDWRLPDGVNGVDLTEKFGLVLRKATPFVAIPKPRLSNLDLYT | |
| CYP706J1 | MIAVLLRNLVNLVSEGWSWWWDGLTENTIFRVVAALSTAAIAISWYAWLIKKASRGLPPLPPGPRGLPLLGNLLFIEPDLHRYFSKLSQLYGPIFKLQLGSKTCIVISSSSVAKEILKDHDVIFANRDVPISALALTYGGQDIAWSHYSPEWRKLRKVFVQEMMSSASLDACSALRRREVQEMVRDVYGKVGTPINMGDQMFLTVLNVVTSMLWGGTLHGEDRSRIGMEFRRVIVEMVGLMGKPNISDLFPALAWFDLQGIESRVKKLVLWFDRIFESLIAQRTQLDGADGGGKNKSKESKDFLQFMLELMHQGDDKTSVSITQLKALFMDIVVAATDTSSTTVEWAMAELLQHPQTMQKAQEELEKVVGNENIVEESHLFQLPYLGAVIKETLRLHPPLPLLVPHSPSTSCIISGYTIPKGSRILFNAWAMQRNPEVWEHPLEFIPERFLEDAASADYKGNNFNFMPFGSGRRICAGLPLAEKMLLYVLASLLHSFDWKLPDGRTSVDLEERFGIVLKKSEPLLAIPTARLSNGHYDPSERIF | |
| CYP706K1 | MLLSSFCVTWPYLWCWDSTSLHPILTILVTLISVLCLLRWFKNSSYEATLPSPLPPGPLGLPLLGYLPFLGTNPHLKFHKLAQVYGPIYKLMLGTKTFIVVSSPSLVKEIVRDQDTVFANRDPPISVLVALYGGTDIASLPLGPRWRKARKIFVSEMLSNTNISSSFSHRKIEVKKSIRDVYEKKIGCPISISELAFLTATNAIMSMIWGETLQGEEGAAIGAKFRAFVSELMVLVGKPNVSDLYPALAWLDLQGIETRTRKVSQWIDKFFDSAIEKRMNGTGEGENKSKKKDLLQYLLELTKSDSDSASMTMNEIKAILIDIVVGGTETTSTTLEWVVARLLQHPEAMKRVHEELDEAIGLDNCIELESQLSKLQHLEAVIKETLRLHPPLPFLIPRCPSQTSTVGGYTIPKGAQVMLNVWTIHRDPDIWEDALEFRPERFLSDAGKLDYWGGNKFEYLPFGSGRRICAGLPLAEKMMMFMLASFLHSFEWRLPSGTELEFSGKFGVVVKKMKPLVVIPKPRLSKPELYQ | |
| CYP707A1 | MDISALFLTLFAGSLFLYFLRCLISQRRFGSSKLPLPPGTMGWPYVGETFQLYSQDPNVFFQSKQKRYGSVFKTHVLGCPCVMISSPEAAKFVLVTKSHLFKPTFPASKERMLGKQAIFFHQGDYHAKLRKLVLRAFMPESIRNMVPDIESIAQDSLRSWEGTMINTYQEMKTYTFNVALLSIFGKDEVLYREDLKRCYYILEKGYNSMPVNLPGTLFHKSMKARKELSQILARILSERRQNGSSHNDLLGSFMGDKEELTDEQIADNIIGVIFAARDTTASVMSWILKYLAENPNVLEAVTEEQMAIRKDKEEGESLTWGDTKKMPLTSRVIQETLRVASILSFTFREAVEDVEYEGYLIPKGWKVLPLFRNIHHSADIFSNPGKFDPSRFEVAPKPNTFMPFGNGTHSCPGNELAKLEMSIMIHHLTTKYRCVCVYYLLITFSFTHFVLFFQIYV | |
| CYP707B2v2 | MWDALSQWGILISLARAWRREKNAKLPRGNWWLPWLGESLDFFWRSPDDFYKTRFSRYGSIFLSHIFGSTTIVTSTPEEAKFILGTRHKLFRAKYPTSIDRVLNHPFWEGDFHCRVRKIVQAPMMPEVLKSQISKFDSLATWTLNTWSHGDHVITHAETRKFSFHVALYLVCSLEPSAESMKMLDDYECVAKGAICFPLNVPGTGFHLALKKSKVILEALDNIIARRRMERSVHNDILSSLLNSSDENGIKLTTDQVKNVLITLLFAGHETTGVLLVWIVKYLTENPQVLHLVKEEQEIVRQSMADDKQPLTWANVRNMPYTLKVVQETLRLANVAPFSPREILEDVEYNGILFPKGWRVQVYYRHFHLNPEYYKEPLKFDPSRFEVPPKPMVYTPFGNGIRLCPGSELVKLEVLIFIHRLVTNYSWHAVGADKGIQYWPTPRPKGGYKIKVHSHAHSSFSQEQL | |
| CYP707C1v1 | MVKQSRVAVEVSSTSGAKTPPGPAWRIPLVGETLSFLRDPHRFYLTRIARYGEIFSTSLFGDKCIIVTTPEASKWLLQSAQKFFKPAYPESANSLIDPTRSFGSEQLHNYVRRIVGSSLYPESLQSHIPAIEALACSVLDSWTKQKSVNVYSEMAKYTFEVAMKILCGMEPGKQMDALFQNMQDFEKAFLTLNINLPFTTYRRGLKARDSMFKAVEEMIQQRRKKKRDWSGREQQQRLDMLDSMICVETKDEKFANAVTDIHVRGIIMTILFAGHETSAAQLVWAIKNLHDNPELLHGVKEEHEAIRRKREPGSPLTWSQVMKEMPLTLRVINETMRTSYVGLFLPREALDDLEYDGYYFPKGWKVYASPSMVHLNPKLYTEPYKFDPTRFQDGGPKPNTFIPFGNGQRLCLGGELAKVEMLVLIHHLVTTYSWKIKEDHGGIRWWPVPIPKGGLVIQVEREVERGLEKETRQGQTGELGSGEFQH | |
| CYP708A1 | MNNLISLDIMKEMWGVALSFVIALVVVKISLWLYRWANPNCSGKLPPGSMGFPVIGETVEFFKPYSFNEIHPFVKKRMFKHGGSLFRTNILGSKTIVSTDPEVNFEILKQENRCFIMSYPEALVRIFGKDNLFFKQGKDFHRYMRHIALQLLGPECLKQRFIQQIDIATSEHLKSVSFQGVVDVKDTSGRLILEQMILMIISNIKPETKSKLIESFRDFSFDLVRSPFDPSFWNALYNGLMVRSARSNVMKMLKRMFKERREEATSDDSKYGDFMETMIYEVEKEGDTINEERSVELILSLLIASYETTSTMTALTVKFIAENPKVLMELKREHETILQNRADKESGVTWKEYRSMMNFTHMVINESLRLGSLSPAMFRKAVNDVEIKGYTIPAGWIVLVVPSLLHYDPQIYEQPCEFNPWRWEGKELLSGSKTFMAFGGGARLCAGAEFARLQMAIFLHHLVTTYDFSLIDKSYIIRAPLLRFSKPIRITISENPLSSSHQNANLF | |
| CYP709B1 | MGLVIFLALIVLILIIGLRIFKAFMILVWHPFVLTRRLKNQGISGPNYRIFYGNLSEIKKMKRESHLSILDPSSNDIFPRILPHYQKWMSQYGETFLYWNGTEPRICISDPELAKTMLSNKLGFFVKSKARPEAVKLVGSKGLVFIEGADWVRHRRILNPAFSIDRLKIMTTVMVDCTLKMLEEWRKESTKEETEHPKIKKEMNEEFQRLTADIIATSAFGSSYVEGIEVFRSQMELKRCYTTSLNQVSIPGTQYLPTPSNIRVWKLERKMDNSIKRIISSRLQSKSDYGDDLLGILLKAYNTEGKERKMSIEEIIHECRTFFFGGHETTSNLLAWTTMLLSLHQDWQEKLREEIFKECGKEKTPDSETFSKLKLMNMVIMESLRLYGPVSALAREASVNIKLGDLEIPKGTTVVIPLLKMHSDKTLWGSDADKFNPMRFANGVSRAANHPNALLAFSVGPRACIGQNFVMIEAKTVLTMILQRFRFISLCDEYKHTPVDNVTIQPQYGLPVMLQPLED | |
| CYP709C1 | MGLVWMVAAAVAAVLASWAFDALVYLVWRPRAITRQLRAQGVGGPGYRFFAGNLAEIKQLRADSAGAALDIGDHDFVPRVQPHFRKWIPIHGRTFLYWFGAKPTLCIADVNVVKQVLSDRGGLYPKSIGNPHIARLLGKGLVLTDGDDWKRHRKVVHPAFNMDKLKMMTVTMSDCAGSMMSEWKAKMDKGGSVEIDLSSQFEELTADVISHTAFGSSYEQGKKVFLAQRELQFLAFSTVFNVQIPSFRYLPTEKNLKIWKLDKEVRTMLMNIIKGRLATKDTMGYGNDLLGLMLEACAPEDGQNPLLSMDEIIDECKTFFFAGHDTSSHLLTWTMFLLSTHPEWQEKLREEVLRECGNGIPTGDMLNKLQLVNMFLLETLRLYAPVSAIQRKAGSDLEVGGIKVTEGTFLTIPIATIHRDKEVWGEDANKFKPMRFENGVTRAGKHPNALLSFSSGPRSCIGQNFAMIEAKAVIAVILQRFSFSLSPKYVHAPMDVITLRPKFGLPMILKSLEM | |
| CYP709D1 | MLKSTIELYIFTTAIAKKSLHSQTKHKSKMDVPSVVIPILVVLVSRLLTSALVHLLWKPYAITKLFRGQGITGPKYRLFVGSLPEIKRMKAAAAADEVAAGAHSHDFIPIVLPQHSKWATDHGKTFLYWLGAVPAVSLGRVEQVKQVLLERTGSFTKNYMNANLEALLGKGLILANGEDWERHRKVVHPAFNHDKLKFMSVVMAESVESMVQRWQSQIQQAGNNQVELDLSRELSELTSDVITRSAFGSSHEEGKEVYQAQKELQELAFSSSLDVPALVFLRGNTRAHQLVKKSRTMLMEIIEGRLAKVEAAEAGYGSDLLGLMLEARALEREGNGLVLTTQEIIDECKTFFFAGQDTTSNHLVWTMFLLSSNAQWQDKLREEVLTVCGDAIPTPDMANRLKLVNMVLLESLRLYSPVVIIRRIAGSDIDLGNLKIPKGTVLSIPIAKIHRDRDVWGPDADEFNPARFKNGVSRAASYPNALLSFSQGPRGCIGQTFAMLESQIAIAMILQRFEFRLSPSYVHAPMEAITLRPRFGLPVVLRNLQG | |
| CYP709E1 | MATLLLLAVAAAAAAWVWWGRYAWRARAVARRLAAQGVRGPRRGGLLRGCNDEVRRRKAEAEADGVAMDVGDHDYLRRVVPHFVAWKELYGTPFLYWFGPQPRICVSDYNLVKQILSKKYGHFVKNDAHPAILSMIGKGLVLVEGADWVRHRRVLTPAFTMDKLKVMTKTMASCAECLIQGWLDHASNSKSIEIEVEFSKQFQDLTADVICRTAFGSNSEKGKEVFHAQKQLQAIAIATILNLQLPGFKYLPTKRNRCKWKLENKLRNTLMQIIQSRITSEGNGYGDDLLGVMLNACFSTEQGEKRDELILCVDEIIDECKTFFFAGHETTSHLLTWTMFLLSVYPEWQDRLREEVLRECRKENPNADMLSKLKEMTMVLLETLRLYPPVIFMFRKPITDMQLGRLHLPRGTAIVIPIPILHRDKEVWGDDADEFNPLRFANGVTRAAKIPHAHLGFSIGPRSCIGQNFAMLEAKLVMAMILQKFSFALSPKYVHAPADLITLQPKFGLPILLKALDA | |
| CYP709F1 | MMVYTYVGYFAAGLAVQVLVAKILKLCWIVLWRPYALIKSFEKQGIKGPSYSILHGTLPEMKTLLKAANEVILDTNCHDIAQRVQPHYNRWSAEYGEVFLFWRGVQPAIRIADPKLAKQILSDKSGAYAQPQFDHRLLSFAGNGVGQLNGPDWVRHRSILTPAFTKDKLKLMTKRMASCTIDMIDDWKNRARIADHQNITIEMSEEFKKLTCDVITHTAFGSNYVEGGEVFKAQDELIHHCVATMADLYIPGSRFLPTPSNRQMWKMENNVNNSLRRLIQGRLESAQARGNLDGCYGDDVLGLLVEASKTTNKSLKLTMDEIIDECKQFFFSGHETTAKLLTWTIFLLSLHQEWQERLREEVLTECGMGIPDADMVSKLKLLNMVLLETLRLYCPVLETLRETSRATKLGDFLIPKGVFITIQLVQLHRSKEYWGEDANDFNPLRFKNGVSQAAKHPNAFLGFGMGPRTCLGQNFAMLEVKLVLSLLLQRFSFFLSPEYKHAPANYLTMEAQYGVPTIVKPLLSK | |
| CYP709G1 | MDCVIRSSVEAFVAILVVLFGSIVVKLFRDLIWRPYAFHKAYAGQGIRGTPYRILAGSVPEYTELLREAHAQPMQNISHDIVPRITPEYHKWCQIYGEPFFYWYGIHSRLYISEPELIKEVLSNKFGHYDKPTPRPILLALLGRGLVFADGLRWVKHRRIVSPVFNVDKLKPMVKKMAACTSSMLENWQEMMAQADSHGKEIDVHHDFRALTADIISHTAFGSSCNEGKEVFELQRQLQEMAAKAEQSVFIPGSQYIPTRKNSHAWKIDRRVKEILNSIIQSRLEPRTTTRAHVGYGSDLLGIMMTANQKELGGSQRNLSMTIDEIMNECKTFFFAGHDTTSNLLTWAVFLLSINPEWQEILRKEVISVCGTDIPDADMLSKMKSMTMVLNETLRLYPPASKIIRKAYKAIKLGQFSLPKGAVLSFSILAMHHNEKFWGLDANLFKPERFAAGVSKAAIHPNAFFPFSLGPRNCVGQNFAMLEAKTVLAMILQRLSFSLSPAYKHAPIAVLTLQPQYGMQIIFKSIEVQT | |
| CYP709H1 | MVVAVQLAALLALLLALWRLVWRPHAVARSFARQGIRGPPYTFLAGSLPEAKRLLMAGRRGTAPLDAGCHDIMPVLLPQFLRWVADYGRTLLFWIGPIPAVLSTDLQLIKQVLTDRTGLYQKDFMIPVLKFLFGNGVILINGDDWKRHRKVVLPAFNHETIKSMSAVTAEVTEQMMQQWRGQIHGSEEESAEIDMIHAFNDLTSKVNGRVAFGTSHREVEEVIVLMREMQKLATAATLDAPILWYLPTRRNLHVRRLNKQLRSKIMSIMQARLAADGADRRGGRGGAVSGGGDLLGLLLEAWTPQPQQHGNGGETLTTDEVIDECKTFFAAGQETTATLLVWAMFLLAVHPEWQDKVREEVVREFCTGDDGEVPHADVLAKLKLLYMVLLETSRLYPPIVYIQRRAAWDAVLGGIKVPQGTVISIPIAMLHRDKQVWGPDADEFNPMRFEHGLTKAAKDPKALLSFSLGPRVCTGQSFGIVEVQVVMAMILRRFSFSLSPKYVHKPKYLLSLTPKLGMPLIVRNVDG | |
| CYP71 | MGALWPWIQACLAIVTLIEILWLLLRNRSTKETARLNLPPGPLGLPIIGNLHQLGSLPHRSFASLSWKHGPLMFLQLGRVPTLVISSAKMAKEVMKTQDLAFASRPFLVAANRLCYGSTDMAFAPYGDYWRQVRKICVLQLLSIKKVQSFKLVREEEVASMVRAISGSFGGGPVNLSEAFYTLANNLICRVALGKSYYSEGQQRKYDFRKIVGEFAELLGAFCARDFFPSMGWVDIVTGIRARLNNNFKDLDGFLDEVITEHQGKEVPDPTTKQQSDFVDILLQLQKDPSLDVPLSMDNIKAIILDMFSGGTETSATTLEWLMSELIRNPIVMRTVQEELQRVVGGKGKVFVEEEDLHELQYLQSTIKETLRLYPPAPLLVPRELREKTTINGHDIPANTRVYINAWAIGRQHDSWERVDEFFPERFMNNDIDFKGHDFEFIPFGAGRRGCPGYHFALQIVELAAANLLHCFNWKLPSGCQGLDMAESSGLTMHRKSPLMLVATPRCT | |
| CYP710A1 | MVFSVSIFASLAPYLISAFLLFLLVEQLSYLFKKRNIPGPFFVPPIIGNAVALVRDPTSFWDKQSSTANISGLSANYLIGKFIVYIRDTELSHQIFSNVRPDAFHLIGHPFGKKLFGDHNLIYMFGEDHKSVRRQLAPNFTPKALSTYSALQQLVILRHLRQWEGSTSGGSRPVSLRQLVRELNLETSQTVFVGPYLDKEAKNRFRTDYNLFNLGSMALPIDLPGFAFGEARRAVKRLGETLGICAGKSKARMAAGEEPACLIDFWMQAIVAENPQPPHSGDEEIGGLLFDFLFAAQDASTSSLLWAVTLLDSEPEVLNRVREEVAKIWSPESNALITVDQLAEMKYTRSVAREVIRYRPPATMVPHVAAIDFPLTETYTIPKGTIVFPSVFDSSFQGFTEPDRFDPDRFSETRQEDQVFKRNFLAFGWGPHQCVGQRYALNHLVLFIAMFSSLLDFKRLRSDGCDEIVYCPTISPKDGCTVFLSRRVAKYPNFS | |
| CYP710B1 | MNATGLLNDGLASLGMSGFGDNLASGPALVAAGGALALGYALWEQMKFRWYRSDKNGNMLPGPASVTPIIGGIVEMVKDPYGFWERQRLYSFPGMSWNSIVGIFTVFVTDPALSRYVFSHNSSDSLLLALHPNAEWILGKTNIAFMSGPEHKALRKSFLALFTRKALGLYVLKQDDVIRKHFNEWMQTAGPREIRPFIRDLNAYTSQEVFVGPYLDDPTEREKFSDAYRAMTDGFLAFPLLLPGTGVWKGRQGRQFIVKTLTRAAARSKVRMAAGQEPECLLDFWTKQILSDIKDAADAGQEAPFYADDKKIAETVMDFLFASQDASTASLVWTITLMAEHPEVLARVRDEQYRLRPNPEEKVTGDMLNEMHYTRQVVKEILRFRPAAPMVPMRAKAPFKLTETYTAPKGALIVPSLVAACKQGYSNPDSFDPDRFSPERAEDIKYASNFLVFGHGPHYCVGKEYAMNHLTVFLALLATSLDFPRIRSKVSDDIIYLPTLYPGDSIFDLSWSAKK | |
| CYP711 | MKTQHQWWEVLDPFLTQHEALIAFLTFAAVVIVIYLYRPSWSVCNVPGPTAMPLVGHLPLMAKYGPDVFSVLAKQYGPIFRFQMGRQPLIIIAEAELCREVGIKKFKDLPNRSIPSPISASPLHKKGLFFTRDKRWSKMRNTILSLYQPSHLTSLIPTMHSFITSATHNLDSKPRDIVFSNLFLKLTTDIIGQAAFGVDFGLSGKKPIKDVEVTDFINQHVYSTTQLKMDLSGSLSIILGLLIPILQEPFRQVLKRIPGTMDWRVEKTNARLSGQLNEIVSKRAKEAETDSKDFLSLILKARESDPFAKNIFTSDYISAVTYEHLLAGSATTAFTLSSVLYLVSGHLDVEKRLLQEIDGFGNRDLIPTAHDLQHKFPYLDQVIKEAMRFYMVSPLVARETAKEVEIGGYLLPKGTWVWLALGVLAKDPKNFPEPEKFKPERFDPNGEEEKHRHPYAFIPFGIGPRACVGQRFALQEIKLTLLHLYRNYIFRHSLEMEIPLQLDYGIILSFKNGVKLRTIKRF | |
| CYP712A1 | MDLKLNTKLIILITSLAFPFMLYALFKWFLKEQGSLAATKLPQSPPALPFIGHLHLIGKVLPVSFQSLAHKYGPLMEIRLGASKCVVVSSSSVAREIFKEQELNFSSRPEFGSAEYFKYRGSRFVLAQYGDYWRFMKKLCMTKLLAVPQLEKFADIREEEKLKLVDSVAKCCREGLPCDLSSQFIKYTNNVICRMAMSTRCSGTDNEAEEIRELVKKSLELAGKISVGDVLGPLKVMDFSGNGKKLVAVMEKYDLLVERIMKEREAKAKKKDGTRKDILDILLETYRDPTAEMKITRNDMKSFLLDVFMAGTDTSAAAMQWAMGQLINHPQAFNKLREEINNVVGSKRLVKESDVPNLPYLRAVLRETLRLHPSAPLIIRECAEDCQVNGCLVKSKTRVLVNVYAIMRDSELWADADRFIPERFLESSEEKIGEHQMQFKGQNFRYLPFGSGRRGCPGASLAMNVMHIGVGSLVQRFDWKSVDGQKVDLSQGSGFSAEMARPLVCNPVDHFNTF | |
| CYP712B1 | MFTLNLDMNYTSCSYLFFTLVTIFLLHRLFSSSSRRGLPPGPRGLPVLGHMHLLRSSLPRSLQALAHTYGPLMTIRIGSLRVLVVSDSDTAKLILKTHDPDFASKFVFGPRQFNVYKGSEFFNAPYGSYWRFMKKLCMTKLFAGYQLDRFVDIREEETLALLSTLVERSRNGEACDLGLEFTALTTKILSKMVMGKRCRQNSNIPKEIRKIVSDIMACATRFGFMELFGPLRDLDLFGNGKKLRSSIWRYDELVEKILKEYENDKSNEEEEKDKDIVDILLDTYNDPKAELRLTMNQIKFFILELFMASLDTTSAALQWTMTELINHPDIFAKIRDEIKSVVGTTNRLIKESDLQKLPYLQAAIKETLRLHPVGPLLRRESNTDMKINGYDVKSGTKIFINAYGIMRDPTTYKDPDKFMPERFLVVEQDTERKMGYYQQYMLELKGQDVNYLAFGSGRRGCLGASHASLVLSLTIGSLVQCFNWTVKGDEDKIKIKLPTGFSASGTAGGSSLMCSPELCFDPFGYQTK | |
| CYP712C1 | MAPSDHSFAYYCCLCITWSTIIIIVVHLFIKTCTSFCNKTRHPPSPLGLPIIGHLHLLSSDLPNSLKTLASRYGPLMKIRFGSTPIYVVSDAKTAKEILKIHDVDFASKYTLGFGLSKFDIYDGYTFFNAPYGTYWRFMKKLCMTKLFRGPQLDRFVHIREQETLKLLKSLVDKSREGKPCDLGEELSVFSSNIICRMVIGNICVEDPNLPIEIRKLVGDIMENAAKFSFNEVFGPLNRFDLLGKGKRLVSATRKYDKLLEQLMKKYEDNFDKLINSGDEEQKDVMIILMEAYKDTNAELKLTRTHIKKFFLEIFFAGVETTATAMQSAITELINNPKAFMKLREEIHSVFGSNYRLLKESDVPKLPFLQAVVKETLRLNPIATLRARQCDVDTRINGYDIKAGTRILINAYAIMRDSDSWEKPDDFFPERFLADSMDTNFDHHPTMDFKGDHDFHFLPFGSGRRACIAASHGLIVTHATIGALVQCFDWEVKDDAKIDNEMATGYSGSRVLPLACYPITRFDPTNA | |
| CYP712D1 | MGTFTDLQYYTIFFILSFISTLLLRSFLNRITTPTTRLRLPPSPPALPIIGHLRLHFLSSSIYKSFHSLSTQYGPLLYLHFGASRCLLVSSAAMAAEIFKTNDLAFASRPRLAFADKLPYGTSSFITAEYGDYWRFMKKLCVTELLGVKQLERSRVVRREELDCFLKKLVESGENGEAVDVRAEVMKLTNHSTCRVILSARCSEDNDEAERLIEMVTEWVELAVKMSFGDVFGPLKRLGFWIYGRKAVELTLRYDEILEKMLKEHEERGKREDKDLMDVLLEVYQDDKAGMKLTRTHIKAFILDLFMAGTNTSAESMQWTIAELINHPDVFKKVREEIDLAVGRTRLVEESDIPNLPYLQAVVKETLRLHPPAPVATRECRKNCKIGGFNIPEKTAVAINLYAIMRDPEIWDDPTEFRPERFLVPSKEQVDLDQTKGQNFNFVPFGGGRRGCPGTLLAFAMMNTTVAAIVQCFDWKLGGDGDGGKVDMQSGPGLTLSMLHPLKCHPIVHFNPFEG | |
| CYP712E1 | MIAIQYVLAIFVLWVITVFLQFIFKRPGKKPAGYCPPPSPPTLPLIGHLHLLTPVAYKGFHALNNKYGPLLYLRLATYPAVLVSSAPLATEIFKALDVHFTSRIKSPFEDNLLFGSSTSFFNAPYGDYWKFMKKICTTELLGTRQMKKLKNVRREEVVRFLSKMLEIGQKHEVANVSAEVLTLANNSTCRMIMSARCSGEDNQAEKCRGLVSESFDLAAKLALFSVFGPLKRIGTWYLRKKIADVPRRYDELFENVLVEHEEKAKREGPHMENKDLMDILLEVYHDKNAEIRITRKQMKTFFLDLFTGGTNTTSDAILWILAELVNHPAAFKKLREEIDSAVGTERLVDEEDIPNLPYFQACVKEAMRLNPPVPLFDRICGENCKLGGYDIPKGITMIMNAYSIMRDPKIWENPNDFIPERFLTEQDNAEGQNLQVYVPFGGGRRMCPGTNMTSSLINCSVTAMVQCFDWKVLGGDGPDGSKVNMDSKSGVVKSMDKPFVAIPVLRRNLFSA | |
| CYP714A1 | MENFMVEMAKTISWIVVIGVLGLGIRVYGKVMAEQWRMRRKLTMQGVKGPPPSLFRGNVPEMQKIQSQIMSNSKHYSGDNIIAHDYTSSLFPYLDHWRKQYGRVYTYSTGVKQHLYMNHPELVKELNQANTLNLGKVSYVTKRLKSILGRGVITSNGPHWAHQRRIIAPEFFLDKVKGMVGLVVESAMPMLSKWEEMMKREGEMVCDIIVDEDLRAASADVISRACFGSSFSKGKEIFSKLRCLQKAITHNNILFSLNGFTDVVFGTKKHGNGKIDELERHIESLIWETVKERERECVGDHKKDLMQLILEGARSSCDGNLEDKTQSYKSFVVDNCKSIYFAGHETSAVAVSWCLMLLALNPSWQTRIRDEVFLHCKNGIPDADSISNLKTVTMVIQETLRLYPPAAFVSREALEDTKLGNLVVPKGVCIWTLIPTLHRDPEIWGADANEFNPERFSEGVSKACKHPQSFVPFGLGTRLCLGKNFGMMELKVLVSLIVSRFSFTLSPTYQHSPVFRMLVEPQHGVVIRVLRQ | |
| CYP714B1 | MVVVVAAAMAAASLCCGVAAYLYYVLWLAPERLRAHLRRQGIGGPTPSFPYGNLADMRSHAAAAAGGKATGEGRQEGDIVHDYRQAVFPFYENWRKQYGPVFTYSVGNMVFLHVSRPDIVRELSLCVSLDLGKSSYMKATHQPLFGEGILKSNGNAWAHQRKLIAPEFFPDKVKGMVDLMVDSAQVLVSSWEDRIDRSGGNALDLMIDDDIRAYSADVISRTCFGSSYVKGKQIFDMIRELQKTVSTKKQNLLAEMTGLSFLFPKASGRAAWRLNGRVRALILDLVGENGEEDGGNLLSAMLRSARGGGGGGGEVAAAAEDFVVDNCKNIYFAGYESTAVTAAWCLMLLALHPEWQDRVRDEVQAACCGGGGRSPDFPALQKMKNLTMVIQETLRLYPAGAVVSRQALRELSLGGVRVPRGVNIYVPVSTLHLDAELWGGGAGAAEFDPARFADARPPLHAYLPFGAGARTCLGQTFAMAELKVLLSLVLCRFEVALSPEYVHSPAHKLIVEAEHGVRLVLKKVRSKCDWAGFD | |
| CYP714C1 | MEKLLALIVVLVILLSLALFYLCNILWLRAVKIRKKLRRQGIRGPKPTFLYGNTKEIKRIRQELKFSQKQGTNNFISTLFPHFLLCRETYGMHMLGPVFLYSTGALEILQVSHPDMVKDIGRWTPSELGKPNYLKKSRKALFGGGLFTENGDEWAYQRKIIAPEFFMDKIKMIQLIEDATVTVLEAWEDMIDDVGGCREIVVDDYLRNLSADVIARACFGSSFTEGEEIFCKLRQLQKAIAQQDSFVGLSALWKYLPTKSNQEIQMLDEQVRLLILDVAKEQHHYQDSHNSLLNAIIDGAQDGRSAAEAEDFIVGNCKTIYFGGHESTVVTAIWCLMLLATHSEWQERARSEAMEVCRGRSTLDVDALRRLKIVTMVIQETLRLYPPASVMMQEALTDVKLGNIEVPRGTIVQVPRLMLHLDKEAWGAHADEFRPDRFANGVAAACRAAHMYVPFGHGPRTCIGQNLAMAELKVVLARLLTKFAFSPSPRYRHSPAFRLTIEPGFGLPLMVTKLP | |
| CYP714D1 | MESFFVFFTAAALPVVVAAAVIAGLCITAAWLARPRRVAEVFRRQGIDGPPPSSFLAGNLPEMKARVAAAASAAAPTADGEETASAGGGGGGRDFEKDGFDDYCTRIFPYFHKWRKAYGETYLYWLRRRPALYVTDPELIGEIGRCVSLDMGKPKYLQKGQEPLFGGGVLKANGACWARQRKVIAPEFYMARVRAMVQLMVDAAQPLIASWESRIDAAGGAAAAEVVVDGDLRSFSFDVISRACFGSDYSRGREIFLRLRELSGLMSETSVIFSIPSLRHLPTGKNRRIWRLTGEIRSLIMELVRERRCAARAAREHGGKAAPPSPPERDFLGSIIENSGGQPRPDDFVVDNCKNIYFAGHETSAVTATWCLMLLAAHPEWQDRARAEVLEVCGGDGAAAPAAPDFDMVSRMRTVGMVVQETLRLFPPSSFVVRETFRDMQLGRLLAPKGTYLFVPVSTMHHDVAAWGPTARLFDPSRFRDGVAAACKHPQASFMPFGLGARTCLGQNLALVEVKTLVAVVLARFEFTLSPEYRHSPAFRLIIEPEFGLRLRIRRAGGQDATSQVDTSTAPVHSSHN | |
| CYP714E1 | MSIVVEVMVALVAVLVALIHFLHVLVLRLRSLRAKLHRQGIHGPSPDFYFGNIKEMKTLLLQQQTQVKQIKQEHEDEDVCASISHSWTSTVFPHIHKWRKQYGPTFLYSTGSIQWLLVTDVEMVKEILLNTSFNLGKPSYLSRDMGPLLGQGIVSSSGLIWSHQRKIIAPELYLDKVKAMVDRVIYSTNILIRSWESRIERDGVVSEIKVDEDLRSLSADIIARVSFGSNYVEGKEIFTKLRDLIKLLSKIYVGIPGFRYLPNKSNRQIWRLEKEINSNISKLVKQRQEEGHEQDLLQMILEGAKNCEGSDGFFSNSISQDRFIIDNCKTIFFAGHDTTAITSSWCLMLLAKYQDWQDRARAEVLEVCGNGNPDASILRTMKTLTMVIQETLRLYPPAVFITRTSFQDINLKGIKVPKGINMQIPIAILQQDIDIWGPDAHEFNPERFANGVLGACKIPQAYMPFGIGSRVCPGQHLSMIELKVFLSLILSKFRVSLSSSYCHSPAFRLLIEPGHGVVLNMTRI | |
| CYP714E4 | MELVLLISLAVVITFLGLLELLYSGLVLKPERLRSVLRKQGIRGPSPSLLLGNISEIRKSQSTTVKASTNEPPVFHNCAATLFPFFEQWRKQYGPVFVFSLGNTQILYVSRADVVREISTCTSLEFGKPSYQQKELGSLLGQGILTSNGKVWAHQRKIIAPELYGDKVKGMMSLIIESTTVLLNSWKSRIDKEGGVAEIKIDEGMRSFSGDVISRACFGSNYSEGAEIFSRLRDLQEAMSKKSLSTGIPGMRYIPTKNNREAWALEKYVRNLILEIVKERKETAHEKDLLQMVLESAKTSNVGQDAMDRFIVDNCKNIYLAGYETTAVSATWCLMLLAANQEWQDRVRAEVLEVCGSGCLPDADMLRKMKQLNMVIHESLRLYPPVAVVSREAFKEMKFGGITVPKGVNVWTMVLTLHTDPEVWGPDAYRFNPDRFAKGITGACKLPHLYMPFGVGPRMCLGQNLAIAELKILIALILSQFSLSLSPKYIHSPALRLVIEPERGVDLLIKTL | |
| CYP714F1 | MEGFPISYNFTCLVLLLTWSLIVYLYYSFWWRPELRLRKQGIRGPPPNFLLGNIPEIKQATVQNRSESTPSMESDSFSGFPSFKQWCKKYGNTYMFKLGALHFLYATNPFMVKEIKLFRSLDLGKPAYLQKDRGVLLGKGVITSNGPAWSHQRKILSPQLYVDKXXDTLNIIVESGITVIKSWERILMESKDGLDADIMVDSHMRSFTSCIISKLMFGHDHCRGMNVTARCHTLFKAMGTPTTIGIPFLRYLPTKANRNAWRLAKEIHSMILDIAKNRCGSSTTKDILQVILEGSENGGPGPSSAHEFIVDNCKDMLLAASEGTAISAMWGLMLLASNPEWQARARSEVKQVCGGHLPNFNMLGKMKLKMVILEVLRLYPPVALVSRRALQDVKLCNMQVPKGVNIWIWAPALHRDPDLWGPDADKFNPERFIDGVSGACKSSHAYIPFGVGARLCPGNKLGMIQLKVVLLAMILSSFNLSISPNYRHSPTLGLLLEPEHGVNLVIQKI | |
| CYP714G5 | MEAFTVKVFISLALAGVLGLFFRLYNALVVKPEKLRSILRSQGISGPPPSFLLGNIREIKKSRSTAVKDSSTDTHNCAAALFPFFEQWRNKYGQVFMFSLGNTQILHANQPDIVREITTCSSLDFGKPSYLQREFGPLLGQGILTSNGAVWAHNRKILAPELYMEKVKGMIGLITESVDTLLNSWRSKIEAEGGIADIRIDEHMKSFSGDVISRACFGGSYTKGEEIFLKLKALQEAMSKKAFSFIPGMRYIPIKRNRDAWALQKDVRNLILKVVSERKELAAHEKDLLQMVLEGAKKSELSQEATDNFIVDNCKNIYLAGYETTAVSAVWCLMLLAANPDWQARVREEVVEICKGRTPDADMIRKMKQMTMVIYESLRLYPPVPVMSREALADMKFGGIHVPKGVNVWNLVVTLHTDPENWGPDALKFNPERFKNGITGACKLPHLYMPFGVGPRVCLGQNLAMVELKILISLILSNFSFSLSPNYKHSAALRLLIEPENGVNLLVKKL | |
| CYP714H1 | MEAEGQWQISEREICWSVVFIATCSIIILLYVKLWYRPQRIRSVLQKQGINGPKPSFPFGNISEMQQLPNQLAPVSLEALDEWAYSIFPYFHTWRQLYGPMFMYSTGTNEHLYVETPELVKWIGMHKSLHLGRPSYLTKTLKPLLGNGIIRSNGLHWAFQRNLLAPEFFHSKIKDWVDIMEESTMAINKKWENHITESEGGIAELVIDGDMKALTADVISKACFGSTYAQGNLIFAKLASMQTALAKPNHIFGFLNLRFLPTKENKEIWKLQKEVEAMILKMIKEREAENQKSSTHGNQTQKDLLQIILEGATSATSTESSGKGIFGPGYNIYQSIVDICKNMYFAGSESTALAITWTLFLFALHPEWQQLVRSEIMETYGNMLPHSFRDMDRLRNLKALTMVIQESLRLYGPAVTTARGVLAEMKLGEHVLPKGINMWLYIPALHRDPDNWGPDAREFKPERFAGGVSAACKYPQAYIPFGLGSRICLGQNFALLEIKEALCLLLSNFSFAVSPNYHHCPQYRMLLTPKYGMRLLVSKVHKTRT | |
| CYP714J1 | MEMNTVQVVLVAVVVVLIHVFNVLLLRSRSLRAKLHRQGIHGPSPHFYFGNIPEMKTLLLQVQSAPITQVKDKDDHDSLSHKWPFTLFPHIQKWISQYGPIYLFSSGTIQWLMVSDIEMVKEIIMYTSLNLGKPSYLSKDMGPLLGQGILTSSGPIWAHQRKIIAPELYLDKVKAMVNLIVDSTNVTLRSWEARLESEGAVSEIKIDDDLRSLSADIIARTCFGSNYIEGKEIFSKLRDLQKLLSKIHVGIPGFRYLPNKSNRQMWRLEKEINSKISKLIKQRQEETHEQDLLQMILEGAKNCEGSDGLLSDSISCDVFMIDNCKNIFFAGHETTAITASWCLMLLAAHQDWQDRARAEVLEVCGKGAPDASMLRSLKTLTMVIQETLRLYSPAAFVVRTALQGVNLKGILIPKGMNIQIPISVLQQDPQLWGPDAHKFNPERFSNGVFGACKVSQAYMPFGIGARVCVGQHLAMTELKVILSLILLKFHFSLSLSYCHSPAFRLVIEPGQGVVLKMTRI | |
| CYP715A1 | MEFFEMSRVWYIFFKVFVVVICLMFLKLFLRCWIWPVRAQKKLRGNGFVGPAPSFPFGNLNDMKKLKMASVVVDNSKSSTIINHDIHSIALPHFARWQQEYGKVFVYWLGIEPFVYVADPEFLSVMSKGVLGKSWGKPNVFKKDREPMFGTGLVMVEGDDWTRHRHIITPAFAPLNLKVMTNMMVESVSNMLDRWGIQINSGNPEFDMESEIIGTAGEIIAKTSFGVTGENGTQVLKNLRAVQFALFNSNRYVGVPFSNILSYKQTVKAKGLGHEIDGLLLSFINKRKISLAEGDDQGHDLLGMLLKADQKGNFTAKELVDECKTFFFAGHETTALALTWTFMLLAIHPEWQDTIREEIREVIGDSKIEYNKLAGLKKMSWVMNEVLRLYPPAPNAQRQARNDIEVNGRVIPNGTNIWIDVVAMHHDVELWGDDVNEFKPERFDGNLHGGCKNKMGYMPFGFGGRMCIGRNLTTMEYKIVLSLVLSRFEISVSPGYRHSPTYMLSLRPGYGLPLIIRPL | |
| CYP715B1 | MELVVQALAAAAALLAVFFLSTLYLSPAATARRLRNAGFRGPTPSFPLGNLREIASSLASNNDTDESNTKGGDIHAAVFPYFARWRRAFGKVFVYWLGTEPFLYVADPEFLKAATAGALGKLWGKPDVFRRDRMPMFGRGLVMAEGDEWARHRHIIAPAFSATNLNDMIGVMEETTAKMLGEWGDMVASGRSCVDVEKGVVRNAAEIIARASFGISADDDDATGARVFHKLQAMQAILFRSTRLVGVPLAGLLHIRATYEAWKLGREIDALLLDIIESRRRREGGGGGKKKKKTTSNDLLSLLLAGSEASAGAERKLTTRELVDECKTFFFGGHETTALALSWTLLMLAAHPEWQAAVREEVVEVAGRSGPLDAAALGKLTKMGCVLSEVLRLYPPSPNVQRQALQDVVVVAGDGEKKVVIPKGTNMWIDVVAMHRDGELWGEEASEFRPERFMREGVQGGCRHRMGYVPFGFGGRICVGRNLTAMELRVVLAMVLRRFAVEVAPEYRHAPRIMLSLRPSHGIQLRLTPLC | |
| CYP716A1 | MYLTIIFLFISSIIFPLLFFLGKHLSNFRYPNLPPGKIGFPLIGETLSFLSAGRQGHPEKFVTDRVRHFSSGIFKTHLFGSPFAVVTGASGNKFLFTNENKLVISWWPDSVNKIFPSSTQTSSKEEAIKTRMLLMPSMKPEALRRYVGVMDEIAQKHFETEWANQDQLIVFPLTKKFTFSIACRLFLSMDDLERVRKLEEPFTTVMTGVFSIPIDLPGTRFNRAIKASRLLSKEVSTIIRQRKEELKAGKVSVEQDILSHMLMNIGETKDEDLADKIIALLIGGHDTTSIVCTFVVNYLAEFPHIYQRVLEEQKEILNNKDVNEKLTWEDIEKMRYSWNVACEVMRIVPPLAGTFREAIDHFSFKGFYIPKGWKLYWSATATHKNPEYFPEPEKFEPSRFEGSGPKPYTYVPFGGGSRICPGREYARLEILIFMHNLVKRFKWEKVFPKENKLVADPAPIPAKGLPI | |
| CYP716B1 | MVWKEAVSVLQKAQELKEPPLMFTVFLASFIGLAFFFYLISNHRTKAWRGIPPGTFGWPLIGETLEFLGCQRKGNPRDFFDSRTQKYGNVFTTSLVGHPTVVFCSPEGNRFLFSNENKLVVNSWPSSVGNLFRSSLITTVGDDAKRLRRILMTFLRPEALREFVGRVDSMTKRHLAEHWIGKDEVMALPLLKRYTFSLACDLFASINTKDDLDRLWLHFMVFVKGVMQIPIDLPGTRYNKTKHAANAIRQQLGSIINERKIALEAGNASPEQDLLSFLLSNVDEQGESLTDNEIQDNILLLLYAGHDTSSSTLTVLLKFLAENPHCYEEVLREQLNIAGSKEEGQLLEWEDLQRMKYSWRVAQEALRLFPAVQGSFRKAIKEFIYDGFTIPKGWKLHWTVNSTHQKSEYFSNPEKFDPSRFEGEGPPPYTFVPFGGGPRMCPGNEFARMEILIFLHNIVKNFNWNLVNPLEKVIVDPMPAPVNGLPIKLVPHD | |
| CYP716C1 | MEVICLLLSMVLLVLALAISIFAFKHSSHSAKNLPPGSLGWPIFGETLDFLFGKPEKFVSDRMKRYSSDIFKTKILGEETAVICGPGGHKFLFSNEQKLFTAFRPHAMQKIFRSYQAAAPAQAQIPREAESKILRSPGFLKPEALVGYLGKMDSITQLHMQTYWEGKDEVKAFALAKTLTLSLACRFFLGSDDPERIARLVSNFDDMTLGMHSIPLNVYGTTFYRANKAAAAIRKELRIIIDEKRADMSKGAQVQDILCHMILATDPSGKHMAEAEIADKIMGLLVAGYSTVATAMTFFMKNVGERPDIYAKILAEQIEVAADKKAGELLDWNDIQKMKYSWNVMYEVMRLTPPLQGTFREALTDFTYAGYTIPKGWKIYWTVSTTNKDPEYFPDPEKFDPSRYDDEKVFPPFTFVPFGGGPRMCPGKEYARLAILTFVHNVVKRFRWEVAFPAEKIVGDMMPTPEKGLPIRLRSRQAERLASL | |
| CYP716D1 | MFSSLDHDSIMILVAIPCLLLLYFAIKTLKERLFPNPHLPPGSLGWPLVGETLQFLPINLPPEIFVNYRMKKYDSPVFKTSLFGETVAVFVGPAGNKFLFSNENKLVNVWWPTSVKKLMKLSLANVVGDEAKRLRKILMTSVDRDALKSYIDRMDLVAQNHIRTRWEGKQQVKVHPTANLYTFELSCRLFASIDDPIHISKLAHHFDIFLKGVIHFPIYIPGTTFYRASKSGDALKEEIRLVARQRRAALDKKMESHRKDLLSHLLVTADESGKLLSESEIVDNMLMLLFVSHETTTSAMTCVIKYLAEMPEVYEMVLREQLDIAKSKEAGELLKWEDIQKMKYSWRVVSEVLRMIPPISGTFRQAIVDFTYAGYTIPKGWKLYWSPNTTTKDPAHFPNAEDFDPSRYEGAGPAPYTHVPFGGGPRMCLGYEYVRPKILVFLHNIVKRFKWDLLIPDEKVPYNPLPAPSHGLPIRIRPHQSSA | |
| CYP716E1 | MPLIILLVVAAKYMDMDLRLLFSYLLPPAVLCISLYLVVSAYRRKSSNAKFPPGKTGWPIIGETWDFVRAGRSGTPEKFVNDRMSKYSTDVFHTSLLGDNLAMFCGVSGNKFLFSSENKYVTTWWPRPIQRILSFPEEIVTSSKDDSTILRRFLPEILKPEALKHYIPVMDSMAKDHLEADWSPYKQVRVLPLSKKYTFALACRLFMNIKDPAHVSRLENHFNLVTNGLVSVPINFPGTTYYRAVKGGKIIREELLAIMKQRKGELASENYEERAEATDLLTLMLLASDDNGQPLNERDIAYKVLGLLVAGHDTTSSAITMVMYYLAEYPHIYQGVLEEQKEIAMSKAPGELLNWDDVQKMKYSWSVACEVLRVSPPVSGTFREVIADFSFAGFTIPKGWKAYWSVYSTHKNPKYFPDPEKFDPSRFEGKGPAPYTFVPFGGGPFMCAGKEYARLEILVFMHNLVNRVKWEKVIPNEKIMYTSFAMPVKGLPVLLQPLRN | |
| CYP716F1 | MAHQQHGFLEGHAESTPAWAAVAAVVAMLVGWLFWRLFSVSPESQGKLPVPPGSFKWPLLGETLDYLDCARRNRVADFFNARVAKYGETFKTHILFNPTVSVTAPDGNKFLFANENKLVQNHWPPSVSRLLGEHSMATKVGEEHRRARRVYTNFFKPEGLQSFVPRIDELARSHNSKYWEGKEFILGGPTVRDFTFAVAADLFLSLKHDDPMFRPFELAACDYLAGILQVPINLPGTAYRKGILGRESQLRVIDMSLKQRRQEMKEGRVPPQQDLMSVLLNTLNEDGTPMSDDQIKDNMLLFVFAGHDTSSSALAGLLKYLSLNPECLKKVLEEQMEIRKEKGGEDIPLSWDDTRKMKYTWRTIQETLRLQPSVQAAFRTVIEEFEYDGYTIPKGWTIFWSVGRSHRNPKFFPDPEKFDPSRFEGTGPAPFTFVPFGGGPHICPGNEFARTEILVYIHYLVLNYEWEMVDPTEDVCIDPMPLFTKQLQLRVRKRFPSL | |
| CYP716G1 | FLQSLLLILIPLIAFFCFFLKTKQIGTKNMPPGAFGWPLVGETYQLLFKNIENFIQERAEKHSSEIFKTNLFGEPTVVMFGPAANKFLSINESKLVKVWYMKSQCKLFNLPDQNQNQTQVGVASPPVKVLGLLKHEGIIRYMGNNNNIESIIQKHFITHWEGKTELKVYPLVKSFSISLAFQFFLGTDETHYVDKFATKFENLFSGIYSVPMDFPGSTYHRAIKGASEIRKEIQYMIKDKIEGLSKGKVMDGLLAHIVDAEKSGKYVPKIEISNTIMGLMNASYISIATTLAFMIKHIGLSPHIYQRIISEHADIKRSSKESGTSQLDWDSIQKLKYTWAVALESMRLYSPAPGAFREAKTDFTYEGFTIPKGWKIFWAFIGTNKNPKYFDKPESFDPSRFEGNNVLAPYTYIPFGSGPRSCPGKDYTRLAILTFIHNLVTKFKWEVMLPDEEVSGAMIPIPTEGIPIRLH | |
| CYP716H1 | DLFLLSFATILTIIIYVFFKYFFAKPKEKIPPGTFGWPIIGETIQFFISLYYGMVHEFVQERTKKYNSHVFKTSLLGQKVVIFSGPAANKFIFTQGNKLIIGWRPKSVQKLFASTSFVPIEHDTKRAHNVISYLLNSQNVERLISTMDNMSHLHLKNHWKGKNEVIVYDLVKLFTFSLSIRAFIGIKESDKILNLYEKFKIFTYGLLAVDINLPGTTFYKAMKAGNELRKQMKVIIKQRRAELSENPNLSKVDVLTQMINEQDEDGKYMTEVEIEDKVFGFIIGSYDTTATTITLTMKYLQQMPEFFNEIIQEQNEISMQMMPRKELCWNDIQKMKKTWSFVNEVLRNTPVVQGIFREVIEDFTYEDFYIPKGWKIYLSFGATQKNGEYFPNPTKFDPSRFEGNGQVPYTSVPFGGGHRMCPGKEFARILILVFLHHLLKNFRWEPKVPSEKILYPFFLLAIPTDGYPITLSAV | |
| CYP716J1 | MMIVVFFLVSTALLILTRSLLQFLRNSSSNSSSSSKGRVPPGSLGVPVIGDSLNFVKALKRNDPWRFYGEKRAKYGTVFKMSLLGSPVVILPAPAGHKLLFGSEEKLMVNSWPVGFKRLLGPGSLTSLTGEDFKRMKKVFMSFLKPEALQRYVPRVSQLSLKHLEDHWEAYAGEEFAIYPAVKSFIFSVACSSFMSLETEEEQLELEEPFAIWTKGLLQLPVNIPGTLFHKALKRREVIHDLLGRLISKRRQEFLQGRASESSDMLSVMLSYRNEDGKPACTDAEIKDNLLLLLFAAHDTSSSTLTLSLKFLAENPYWRNQVLQENLAISQEKSGQDGYSLEWDDLRGMKVSWRVLQETLRLQPPALSGYREVIQDFEFGDYLIPKGWKACWTVVSHRLPEFFPDPEKFDPSRFEGDGPTPYTYVPFGGGPRMCPGNEYAKMVMLVLLHHLVLRFDWQLADPDEGVTMDPMPMPQNGLNVKLHKRT | |
| CYP716K1v1 | MILLLGACFLLLITTIFAIRFSPSSNLPPGNLGWPIVGETLQFLALFRKNKAYSFFHERMAKHGGVFKTSLLGSPTIVMPGPDGNKFLFSQENKLVVGCWPPSTASLLGPCSLAVQTGQEHRRLREVFMTFLSSQALGRYLPKLCLLAQSFLQSKWNEEAVVTVAPLVQSFVFSAACNLFLSMDKESDQELLLVPFYKFVKGMMSLPVHFPGTRYYEALKSREAILRLLDPVISARKKELLANPTDDRDMLSVLLTTCDEDGKLISENEIEDNVLLMLFAGHETVFRALTITMKMLTDNPHWKEELYQEHLEIRASKSKPDYVLEWNDLRKMKLTWCSVQESMRLYPPSPGATRKATQEFEYAGYRIPEGYKLMWSVNTSRMKDEFFPEPQKFDPLRFQGNGPAPYVFTPFGGGPRTCPGNEFAKMEMLVFLHYLLLSHDWKPVITNEGIIVETAPLPAHGLPVKLSKR | |
| CYP716L1v1 | MALLAMVALLFFLVAPLFWIFNLIVASRKETPAQALQIPPGNLGWPLIGETFRYAVQSGSTFYDERVAKYGAVFKTSLFGSKTVVLPAPEGNRLILMNENKLVSVSYPKSVSVLLGENSLIALRGDEHRRSKALLMTFLRPEMLQKFVGRVCKVVHDHLQKFWSGGDEVIRVYNLMKMFTFALACDLLMGLDIGDEEMEFLARDFDTLVRGLFQLPIDLPGTQFCRAKAARKKLDQCFDRHIREKRRELAGSFRARSHEQDMLEVLLTTRDENGEFSTDLAIKDNIVSLLFAGHDTSSVALTWTLKFLADSPSCMDKVVQENLAVRSSRSSSELSWEDLRKLKYTWQVVQESMRMRPPVGGGFREALVDLEFDGYLNWTTATSYRKPEFFVEPNKFDPSRFDGGNGIAPYTFLPFGAGARMCPGSEFAKMEILVFLHYCVLQFDWKLLEPNEQVIIDPMPRPVHGMPVRISKRN | |
| CYP716M1v1 | MDLPPGSLGLPLLGETLQFIRYTKSNRPWEFIEQREAKYGKIFKTSLFGSPMVMVSPPQGNKLVFSNHNLLVETAWPSPMKTLVGSNAINFMSGEEAKSFRDVLMTFLSAQAVQSQVVPTSNMIQDHLHKHWKHGETVLAYSLIKQALFSVTCCAFLSVSDEEEQLELLEPLAKIIKGLISLPLDLPWTNFHHAKKGRVELYKMFDKYIARRRIELENGSSSQQDLLSLLLSTKLDNGKLMNDDQIKDNILSLLFAGHDTSSSSLAMTLKCLAQNPACYQELRREHLDILSAKQPGEELNQNDLRKMKYTWMVIQETLRVMPTGFGILRKALKDIEMDGFTIPKGWQLLISGYRSYRKPEFFAEPFKFEPSRFAEGTGPVPYTYIPFGGGPRICPGIQLAKMQVMVFLHHLVTRYEWTLVEPDEPVSYTPVAMPTKGLPIKLK | |
| CYP716N1 | MSQALFFVLLFVISVIWFTFTNRNRKRKHASLPPGNMGLPFIGETLPFLRSLVSNQPWEFFRVREAKYGKVYKTRLFGMPVVVVSPPVGTRFMFADTNHTLITKSWPVPVIKLFPESAFVRPDASGSRQLITSFLGPECMKRYVTSTSVIVQKHLDSWPTGELVRVYPLIKRCLFSIVCNMYLGLTDEKEVMELMEPFEKVIHGIISIPVNLPGTAFHRAKLGQKEICNILEKHIAKRRINSQLSPARDQDLLSMLLSTRSKEGTAMTDNEITHNILGLLVSGHELSASSISMTIKSLVENQTVYKEMKRVHCEIGSFKRPREPLEPLDLKQMKYSWRVVQESLRLRPTAPAVARKTLTDVELEGYTIPKGWQMFSAVYNSHTTPEFFPDPLKFDPSRFERAGPNPYTYFPFGGGPRICGGIEQVKMHSLVILHHITTRFDWTLMEPDEPIKISPVAVPAHGLPLELRLV | |
| CYP716P7 | MPWITVVASFLIPVLGTLWIWRYFSRERYDPPLPPGSMGLPLVGETLHILYAMKTSTLWEFYGAREKKYGPIYKTHIFGRPTIVVSPPLGFKLLFSNHGKLVESSWPQPMKTLLGDKCLFFMEGQKAKSFRHILLAFLGPDAIRRYVERASVIIQEHIDKFWMAGSEVKAYPLVKKALFSLVFSLFLSISDEEEERELLAPFQGFLQGLLELPIDLPGTMFRRAKVGRAKIFKKLDEYIAKRKIELETGKAWPQQDFLSVLLTTKGEDGEPMTKEEIKQNILMLVMSAHDTTVSSLVSSMKYIGENPWCYDRLREEHVSIALAKSQKEPLTHSDLQKMDYTWKIVQEAMRLAPPAAGNLRRATTEFTMDGFTVPKDWQLNWTVFRSHKKKEFFEEPEMFNPDRFDRPLLPNTYVPFGGGPRICPGYELAKMQDRIFLHYLVTRFKWTLLDPNEAIHMTPLALPVNGLGIKLVSNPVKI | |
| CYP718 | MVLEPNFVLSWVFLCIAATISSTLFFFRKKHHRFITKKIQKKKKLLPGEMGLPWIGETMDFYKAQKSNRVFEDFVNPRIIKHGNIFKTRIMGSPTIVVNGAEANRLILSNEFSLVVSSWPSSSVQLMGMNCIMAKQGEKHRVLRGIVANSLSYIGLESLIPKLCDTVKFHHETEWRGKEEISLYRSAKVLTFTVVFECLYGIKVEIGMLEVFERVLEGVFALPVEFPCSKFARAKKARLEIETFLVGKVREKRREMEKEGAEKPNTTLFSRLVEELIKGVITEEEVVDNMVLLVFAAHDTTSYAMSMTFKMLAQHPTCRDTLLQEHAQIKANKGEGEYLTVEDVKKMKYSWQVVRETMRLSPPIFGSFRKAVADIDYGGYTIPKGWKVILWTTYGTHYNPEIFQDPMSFDPTRFDKPIQAYTYLPFGGGPRLCAGHQLAKISILVFMHFVVTGFDWSLVYPDETISMDPLPFPSLGMPIKISPKVS | |
| CYP719A1 | MEMNPLLVCATVAIVFATTTIIRILFSSSSLPQMKWPSGPRKLPIIGNLHQLGDDVLHVALAKLAKVHGSVMTIWIGSWRPVIVISDIEKAWEVLVNKSADYGARDMPEITKIASASWHTISTSDAGSFWQNVRKGLQSGAMGPLNVAAQNQYQERDMKRLIKAMSDEAANNNGIVKPLDHIKKNTVRLLTRLIFGQAFDDNKFIESMHYEIEDIIRISGYARLAEAFYYAKYLPSHKKAEREAFLVKCRVEELVRPLLSSKPPTNSYLYFLLSQNFEEEVIIFCIFELYLLGVDSTSSTTTWALAYLIREQGAQEKLYQDIRMTLGDVDLVKIEDVNKLKYLQGVVKETMRMKPIAPLAIPHKTAKETTLMGTKVAKGTRIMVNLYALHHNQNIWPDPYKFMPERFLEGETGTAYNKAMEQSFLPFSAGMRICAGMDLGKLQFAFALANLVNAFKWSCVEEGKLPDMGEELSFVLLMKTPLEARIAGRNV | |
| CYP719B1 | MAPINIEGNDFWMIACTVIIVFALVKFMFSKISFYQSANTTEWPAGPKTLPIIGNLHQLGGGVPLQVALANLAKVYGGAFTIWIGSWVPMIVISDIDNAREVLVNKSADYSARDVPDILKIITANGKNIADCDSGPFWHNLKKGLQSCINPSNVMSLSRLQEKDMQNLIKSMQERASQHNGIIKPLDHAKEASMRLLSRVIFGHDFSNEDLVIGVKDALDEMVRISGLASLADAFKIAKYLPSQRKNIRDMYATRDRVYNLIQPHIVPNLPANSFLYFLTSQDYSDEIIYSMVLEIFGLGVDSTAATAVWALSFLVGEQEIQEKLYREINNRTGGQRPVKVVDLKELPYLQAVMKETLRMKPIAPLAVPHVAAKDTTFKGRRIVKGTKVMVNLYAIHHDPNVFPAPYKFMPERFLKDVNSDGRFGDINTMESSLIPFGAGMRICGGVELAKQMVAFALASMVNEFKWDCVSEGKLPDLSEAISFILYMKNPLEAKITPRTKPFRQ | |
| CYP71A1 | MAILVSLLFLAIALTFFLLKLNEKREKKPNLPPSPPNLPIIGNLHQLGNLPHRSLRSLANELGPLILLHLGHIPTLIVSTAEIAEEILKTHDLIFASRPSTTAARRIFYDCTDVAFSPYGEYWRQVRKICVLELLSIKRVNSYRSIREEEVGLMMERISQSCSTGEAVNLSELLLLLSSGTITRVAFGKKYEGEEERKNKFADLATELTTLMGAFFVGDYFPSFAWVDVLTGMDARLKRNHGELDAFVDHVIDDHLLSRKANGSDGVEQKDLVDVLLHLQKDSSLGVHLNRNNLKAVILDMFSGGTDTTAVTLEWAMAELIKHPDVMEKAQQEVRRVVGKKAKVEEEDLHQLHYLKLIIKETLRLHPVAPLLVPRESTRDVVIRGYHIPAKTRVFINAWAIGRDPKSWENAEEFLPERFVNNSVDFKGQDFQLIPFGAGRRGCPGIAFGISSVEISLANLLYWFNWELPGDLTKEDLDMSEAVGITVHMKFPLQLVAKRHLS | |
| CYP71A10 | MALLSSVLKQLPHELSSTHYLTVFFCIFLILLQLIRRNKYNLPPSPPKIPIIGNLHQLGTLPHRSFHALSHKYGPLMMLQLGQIPTLVVSSADVAREIIKTHDVVFSNRRQPTAAKIFGYGCKDVAFVYYREEWRQKIKTCKVELMSLKKVRLFHSIRQEVVTELVEAIGEACGSERPCVNLTEMLMAASNDIVSRCVLGRKCDDACGGSGSSSFAALGRKIMRLLSAFSVGDFFPSLGWVDYLTGLIPEMKTTFLAVDAFLDEVIAEHESSNKKNDDFLGILLQLQECGRLDFQLDRDNLKAILVDMIIGGSDTTSTTLEWTFAEFLRNPNTMKKAQEEVRRVVGINSKAVLDENCVNQMNYLKCVVKETLRLHPPLPLLIARETSSSVKLRGYDIPAKTMVFINAWAIQRDPELWDDPEEFIPERFETSQVDLNGQDFQLIPFGIGRRGCPAMSFGLASTEYVLANLLYWFNWNMSESGRILMHNIDMSETNGLTVSKKVPLHLEPEPYKT | |
| CYP71A12 | MEMILMVSLCLTTLITLFLLKQFLKRTANKVNLPPSPWRLPLIGNLHQLSLHPHRSLHSLSLRYGPLMLLHFGRVPILVVSSGEAAQEVLKTHDLKFANRPRSKAVHGLMNGGRDVVFGPYGEYWRQMKSVCILNLLTNKMVASFEKIREEELNEMIKKLEKASSSSSSENLSELFVTLPSDVTSRIALGRKHSEDETARDLKKRVRQIMELLGEFPIGDYVPALAWIDRINGFNARIKEVSQGFSDLMDKVVQEHLEAGNHKEDFVDILLSIESEKSIGFQAQRDDIKFMILDMFIGGTSTSSTLLEWIMTELIRNPNVMKKLQDEIRSTIRPHGSYIKEKDVENMKYLKAVIKEVFRVHPPLPLILPRLLSEDVKVKGYNIAAGTEVIINAWAIQRDPAIWGPDAEEFKPERHLDSTLDYHGKDLNFIPFGSGRRICPGINLALGLVEVTVANLVGRFDWRAEAGPNGDQPDLTEAFGLDVCRKFPLIAFPSSVI | |
| CYP71A13 | MEMILSISLCLTTLITLLLLRRFLKRTATKVNLPPSPWRLPVIGNLHQLSLHPHRSLRSLSLRYGPLMLLHFGRVPILVVSSGEAAQEVLKTHDHKFANRPRSKAVHGLMNGGRDVVFAPYGEYWRQMKSVCILNLLTNKMVESFEKVREDEVNAMIEKLEKASSSSSSENLSELFITLPSDVTSRVALGRKHSEDETARDLKKRVRQIMELLGEFPIGEYVPILAWIDGIRGFNNKIKEVSRGFSDLMDKVVQEHLEASNDKADFVDILLSIEKDKNSGFQVQRNDIKFMILDMFIGGTSTTSTLLEWTMTELIRSPKSMKKLQDEIRSTIRPHGSYIKEKEVENMKYLKAVIKEVLRLHPSLPMILPRLLSEDVKVKGYNIAAGTEVIINAWAIQRDTAIWGPDAEEFKPERHLDSGLDYHGKNLNYIPFGSGRRICPGINLALGLAEVTVANLVGRFDWRVEAGPNGDQPDLTEAIGIDVCRKFPLIAFPSSVV | |
| CYP71A32 | MAALLVFFSVSLILLAVLFHKRKSSLSSRKRPPPSPLRLPVIGHFHLIGALSHRSFTSLSKRYGEVMLLHFGSAPVLVASSAAAAREIMKNQDVIFASRPRLSIFDRLMYSGKGVAFAPYGEHWRNARSMCMLQLLSAKRVQSFGGIREEETSAMIEKIRRSKPTTVVNLSEMFMALTNGVIHRAVLGRKGDGGDDFNRILIKVIKLLGSFNVGDYVPWLSWINRINGVDAEVEKVGTKLDGSMEGILRKYRRKKVGDDETNFVDTLLQFQRESKDTDPVEDDVIKALIFDMVSAGTDTTFAALEWTMAELIKNPRTLKTLQNEVREVSRNKGGITEDDVDKMPYLKAVSKEILRLHPPFAILLPRELTQDANMLGYDIPRGTVVLVNNWAISRDPSLWENPEEFRPERFLETSIDYKGLHFEMLPFGSGRRGCPGSTFAMALYELALSKLVNEFDFRLGNGDRAEDLDMTEAPGFVVHKKSPLLVLATPRQS | |
| CYP71AA2 | MAGIMDSTTASYYTTLLCGALLLAAVVFKLKTAAAFSRHNAGVNLPPGPWALPVIGSIHCLLGSLPHHAMRELSRRYGPVMLLRLGHVRTLVLSSPEAAREVMKTHDVAFANRAVTPTASILTYGARDIVFAPFGKHLRELRKLCALELLSPRRVRSFRHVREEEAARLARSVAAAASASSAVNVSELVKIMTNDVTMRAIIGDRCPQREEYLEALDKTMDLLAGFNLVDLFPGSPLARVLGGRSLRTTKRVHEKLHQITEAIIQGHGIKDTVGDEHHECEDILDVLLRFQRDGGLGITLTKEIVSAVLFDLFAGGSETTSTTILWAMSELMRSPHVMEQAKYEIRQVLQGKAMVSEADIEGRLHYLQLVIKETLRLHPPVPIVIPRLCSKPNSKIMGYDIPQGTSVLVNVSAIGRDEKIWKNVNEFRPERFKDDIVDFSGTDFRFIPGGSGRRMCPGLTFGVSNIEIALVTLLYHFDWKLPSETDTHELDMRETYGLTTRRRSELLLKATPSY | |
| CYP71AB1 | MANLIYYSLLIILPFLFLIKFYKAMFSSRKQARRLPPCPWQLPIMGSIHHLIGDLPHRALRDLSRRYGPVMLLKFGQVPFIIVSSPEAAKDIMKTHDSIFATRPQSEIMKIITKRGQGLVFAPYDDQWRQLRKICIRELLCAKRVQSFCAIREEEAARLVKSISSDQAHLVNLSKKLADYATDAAIRIITGTRFENQEVRDKFQYYQDEGVHLAASFCPANLCPSLQLGNTLSRTAHKAEIYREGMFAFIGGIIDEHQERRAQDMSHKEDLIDVLLRIQQEGSLESPVSMETIKFLIFDILAGGSETVTTVLQWAMAELMRNPTVMSKVQDEVREVFKWKEMVSNDDINKLTYLQFVIKETLRLHTPGPLFMRECQEQCQVMGYDMPKGTKFLLNLWSISRDPKYWDDPETFKPERFEDDARDFKGNDFEFISFGAGRRMCPGMLFGLANIELALANLLFYFDWSLPDGVLPSELDMTENFGVTVRKKEDLLLHASLYAQLSC | |
| CYP71AC1 | MDLMKSNPLQGSPWSLLNLLVLIIVAAMICGELCRRRRRRRGDENGGATRLPPGPWRLPFVGSLHHLAVMRPRGVVVHRALAELARRHDAPVMYLRLGELPVVVASSPEAAREVLKTHDAAFATRAMSVTVRESIGDKVGILFSPYGKKWRQLRGICTLELLSVKRVRSFRPIREEQVARLVDAIAAAAASSTAEAAAVNISRQITGPMTDLALRAIMGECFRWREEFLETLAEALKKTTGLGVADMFPSSRLLRAVGSTVRDVKLLNAKLFELVECAIEQHREQIRAAHDNGGDDDDAHGHGDKECFLNTLMRIQKEGDDLDDLTMATVKAVILDMFAGGSESTSTTLEWALSELVRNPHVMQKAQAEIRHALQGRTRVTEDDLINLKYPKNIIKETLRLHPVAPLLVPKECQESCKILGYDVPKGTIMFVNAWAIGRDPRYWNDAEVFMPERFEKVAVDFRGTNFEFKPFGAGRRMCPGITFANATIEMALTALLYHFDWHLPPGVTPDGLDMEEEFGMSVSRKRDLYLRPTLHMGLETI | |
| CYP71AD1 | MEIELSPVLLLLPFLLLGFLYLTGGVLRSGGNARRRLAPAPRGLPVIGNLHQVGALPHRALRALAAATGAPHLLRLRLGHVTALVASSPAAAAAVMREHDHVFATRPYFRTAEILTYGFKDLVFAPYGEHWRHARRLCSEHVLSAARSHRYGPMREQEVALLVNAIRTEAAAAAVDVSKALYAFTNAVICRAVSGRLSREDEGRSELFRELIEENATLLGGFCVGDYFPALAWADAFLSGFAARACRNLRRWDELLEEVIAEHEARLRGGDDGGGEEHREEDFVDVLLALQEESQRHDGSFKLTRDIIKSLLQDMFAAGTDTSFITLEWAMSELVKNPAAMRKLQDEVRRGGGATTAATPYLKAVVKETLRLHPPVPLLVPRECARDTDDDATVLGYHVAGGTRVFVNAWAIHRDAGAWSSPEEFRPERFLPGGGEAEAMDLRGGHFQLVPFGAGRRVCPGMQFALATVELALASLVRLFDWEIPPPGELDMSDDPGFTVRRRIPLRLVAKPVGSEDDK | |
| CYP71AE1 | MASLATVPNLPLLLLLHYALATFTASRARKNNKDRLPPSPLALLVIGHLLHLMGSLPRTSPSAASPHGTGPTCSSGLAPCRCSLRRRRVPAAEAILRTHDHVFASRPRTVLLANIVFYRSRDVRFAPYGDHWRQARKLVTTHLLSAKKVRSLRLAREEEVSLVMTKISKAATASAVVDIGQILRSFTNDMICRTVSGKCPRDDRKRIFQELANETSLLLGGFDIEEYFPVLARVGLVGKMMCLKAERLKKRWDELLEELINDHENDDHSCNLISDQNDEDFVDILLSVRQEYGFTREHVKAILDVFFGGIDTSALVLEFTIAELMQRPRMLKKLQDEVRACIPKGQKIVSEVDINNMAYLRAVIKEGIRLHPVAPVLAPHISMDDCNIDGYMIPSGTRVLVNVWAIGRDPRFWEDAEEFVPERFIDSMSSAAANVNFTENDYQYLPFGYGRRMXPGMKFGIAVVEIMLANLMWKFDWTLPPGTEIDMSEVFGLSVHRKEKLLLVPNNMSSC | |
| CYP71AF1 | MEQYLFLATLLILSLAFVKLRPRNNGENPPPGPWQLPVIGSLHHLAGALPHRALRDLATRHGELMLLRLGELPVVVASSPAAAREVMRTHDAAFATRPQTATLRALTRDGLGVAFAPQGEHWRCLRKLCVTELLGARRVRCLRRAREAEAAALVASLSTTTPEPVNVSSLVARYVTDAVVRAVVGDRISDRDAFLERLEEGVKVAAGFTLADVFPSSRLARALSGTARRAEAHSREMTRLMDGVIEEHRQRRAATGWRDEEDEDLLDVLLRIQKDGGLQIPLDMGTIRAIIIDLFSAGSETTGTTLQWAMAELMRNPAALRKAQAEVRGVLAGHSHVTEDALPDLHYLHLVIKETLRLHVAVPLLLPRECQEPRLRVLGYDVPERAMVLVNAWAICRDTAVWGPDAEEFRPERFDGGAVDFKGTDFEFVPFGAGRRMCPGVAFAVAIMELGLASLLFHFDWELAGGTAAGELDMAEGLGITARRKSDLWLHATVSVPVPNTETS | |
| CYP71AG1 | MTLSAPLSYNTIILVFVVFIISYVSLLVGGGGKKSVANAAANRLPPPSPRGLPVIGNLHQLGSLPHRSLRSLAAAHGPVMLIRLGQVPAVVVSSASAAREVLQAQDHVFAGRPSLTIPRRLLYGCTDIAFAPHGAYWRGARKMSVRHLLSPPRVRAYRAVREQEVDALVRRVLEQACGAGGGVVRLSELLNDFAKDVAGRIVLGLRAAGDDGWRGKVDALLEESNVLLGAFHVGDYIPWLSWVSHVDGTDARVTRAFEKMDRILEEMVDAAATRGREMPLSDSGEEASGGDDAFIHVLLSLQQQQRQQQEEEPTAEWRLSRDNVKALLEDLFGAGTEATIIVLEWAMAELLRNKGVMEKLQREVRQAQARARRSSSSDIIVGEQDLAGTGMEYLRAVIKETMRLHTPGPLLLPHKSMEATRISHGHGYDVPSDTMVIVNAWAIGRDPEAWESPADEFRPERFVGSGVDFRGHHFQLIPFGAGRRMCPGINLAMSVVELALANLVARFDWALPGAELELDMEETTGCTARKKAPLCAVATLLP | |
| CYP71AH1 | MKFLLVVASLFLFVFLILSATKRKSKAKKLPPGPRKLPVIGNLLQIGKLPHRSLQKLSNEYGDFIFLQLGSVPTVVVFSAGIAREIFRTQDLVFSGRPALYAGKRFSYNCCNVSFAPYGNYWREARKILVLELLSTKRVQSFEAIRDEEVSSLVQIICSSLSSPVNISTLALSLANNVVCRVAFGKGSDEGGNDYGERKFHEILFETQELLGEFNVADYFPGMAWINKINGLDERLEKNFRELDKFYDKIIEDHLNSSSWMKQRDDEDVIDVLLRIQKDPNQEIPLKDDHIKGLLADIFIAGTDTSSTTIEWAMSELIKNPRVLRKAQEEVREVAKGKQKVQESDLCKLEYLKLVIKETLRLHPPAPLLVPRVTTASCKIMEYEIPADTRVLINSTAIGTDPKYWENPLTFLPERFLDKEIDYRGKNFELLPFGAGRRGCPGINFSIPLVELALANLLFHYNWSLPEGMLPKDVDMEEALGITMHKKSPLCLVASHYNLL | |
| CYP71AJ1 | MKMLEQNPQYLYFFSLFLVTIFLYKWLTLKKTPLKNLPPSPPQYPIIGNLHQIGPDPQASLRDLAQKYGPLMFLKFGTVPVLVVSSADAAREALKTHDLVFADRPYSSVANKIFYNGKDMVFARYTEYWRQVKSICVTQLLSNKRVNSFHYVREEEVDLLVQNLENSHSKVANLTELLIEVTGNVVCRVSVGSGDKVDSYKILILEIMDMLGYSRSIEDFFPLLGWVDWLTGLRGKVAEAAKGVDTFLEGVLKEHLSTTGSKYNDFVSILLEIQEADAGSSMDNECIKSLIWDMLGAGTETISTALEWTLAALIKNPDAMFKLQNEVREIGKGKSKISEADLVKMNYLQAVMKESMRLYFTAPLLVPREARQDIKFMGYDISSGTQVLINAWAIARDPLLWDKPEEFRPERFLNSPIDYKGFHYEFLPFGAGRRGCPGIQFAMCINELVVANLVHKFNFELPDGKRLEDLDMTAASGITLRKKSPLLVVARPHV | |
| CYP71AJ1 | MKMLEQNPQYLYFFSLFLVTIFLYKWLTLKKTPLKNLPPSPPQYPIIGNLHQIGPDPQASLRDLAQKYGPLMFLKFGTVPVLVVSSADAAREALKTHDLVFADRPYSSVANKIFYNGKDMVFARYTEYWRQVKSICVTQLLSNKRVNSFHYVREEEVDLLVQNLENSHSKVANLTELLIEVTGNVVCRVSVGSGDKVDSYKILILEIMDMLGYSRSIEDFFPLLGWVDWLTGLRGKVAEAAKGVDTFLEGVLKEHLSTTGSKYNDFVSILLEIQEADAGSSMDNECIKSLIWDMLGAGTETISTALEWTLAALIKNPDAMFKLQNEVREIGKGKSKISEADLVKMNYLQAVMKESMRLYFTAPLLVPREARQDIKFMGYDISSGTQVLINAWAIARDPLLWDKPEEFRPERFLNSPIDYKGFHYEFLPFGAGRRGCPGIQFAMCINELVVANLVHKFNFELPDGKRLEDLDMTAASGITLRKKSPLLVVARPHV | |
| CYP71AK1 | MSSYVVVAAALLVFVVVVVAAIKNLGKGKLPPSPPSLPFVGHLHLVGELPHRSLDALHRRYGSDGGLMFLRLGRAGALVVSTAAAAADLYRGHDLAFASRPPSHSAERLFYGGRNMSFAPLGDAWRRTKKLAVAHLLSPRRARRAQRGAGAVQPRELRTPNKKGVITRVAAGGSGATAERFRKMMADTSELLAGFQWVDRLPEAAGWAARKLTGLNKKLDDMADESDRFLGEILAAHDDEKAEGEEEDFVDVLLRLRRQGAAAAGGLELAEDNVKAIIKDIMGAATDTSFVTLEWIMTELIRNTQVMSKLQNEIIQVTGSKPTVTEEDLTKLDYLKAVIKEVLRLHPPAPLLIPHHSTMPTTIQGYHIPAKTIAFINVWAIGRDPAAWDTPDEFRPERFMGSAVDFRGNDYKFIPFGAGRRLCPGIILALPGLEMVIASLLYHFDWELPDGMDVQDLDMAEAPGLTTPPMNPVWLIPRCRTI | |
| CYP71AL1 | MEITDFSTFLLAFLLLSYLLVTGRRLISKKSTGKLPPGPKKFPIVGNLPQLALAGTLPHRAMRDLAKTYGPLMHLRLGEVSQLVVSSPEMAKEVLKTLDPMFASRPDLILADIMLYDNAGLTFAKYGDYWRQLKKIFATELLSAKRVKSFRSLREEETLNTIRWISSNEGKPINMTNTLLNLVFGVLSRATFGKKSPEQDKLVYIVNKAAELATGGNISDLFPSIKFFRLISVVNYKLKSMFAESNRLLDMIMKEHKKGNGSGESKDLVDVLLGYQRENAEFSLTDENIKAVLLDIFIGGTDGSFTTLDWAMSELMRAPTVLKRAQEEVRQAFETDGYIDEEKFEDLKYVTSIIKETLRLHPPAPLLVPRSNDETAHILGYEVPAKSKILVNVWAINRDPRYWEDAESFKPERFLGSSVGYKGTDFHFLTFGAGRRMCPGMVYGYANIVHPLVKLLYYFDWNLPSGIKPEELDMTEEHGLSVKRKADLYLIPSVRNSISHL | |
| CYP71AM1 | MDEYFVDLPYPNLCLYGSCLVLAVVVARAIILSGSGKKPGGLPPGPWQLPVIGSLHHLLRGLPHHAIRDLSLRHGPLMLLRICERTAIVVSSAEAVAEMLKRHDAAFSERPSSPGIEELSRHGQGVIFAPYGDHWRLLRRILMTELLSPRRVEAFRHIREDEAARLVSSLSSLPQPVDMDERLEVFVADSSVRAILGDRLPDRAAFLKMVKAGQDPSSLFDLRDLFPSSWLVRMLPRSRKAERHLQEMFRLMDDILVSHSQRRVDDDSPDGGGGGAVDEEHDMVDVLLRIQKQGDMRVSLNHGVIRAALIDAVGAALDTTSTTLRWAMAELIANPRVMHKAQLEIRRVMAAGQQRRVHEATLRDLHYLKAVIKETLRLHPPAPFVPRVCLDDGIKIQGYHVPRGTIVVANVWAISRDPKYWEDPDMFIPERFHQGDPDHHRCFDFKGFDFEFTPFGAGRRMCPGMNFAHMNVEIALASLLYHFDWKLPDGATPEEIDMTELWGVTVARKAKLLLHPIPCIPAAASIDA | |
| CYP71AN1 | MTLLYFQQTWQEIRPKIGLNYLVFFLIFLSFILFLFKLTRSRKLNLPPSPPKLPVIGNIHHLGTLPHRSLQALSEKYGPLMLLHMGHVPTLIVSSAEAASEIMKTHDIVFANRPQTTAASIFFHGCVDVGFAPFGEYWRKVRKISVQELLGPKTVQSFHHVREEEAAGLIDKIRFACHSGTSVNISEMLISVSSDIVSRCVLGRKADKEGGNSKFGELTRTFMVQLTAFSFGDLFPYLGWMDTLTGLIPRLKATSRALDSFLDQVIEEHRSLESDGDRCAQTDFLQALLQLQKNGKLDVQLTRDNIIAVVLDMFVGGTDTSSTMMEWAIAELVRNQTIMRKAQEEVRRIVGKKSKVEANDIEEMGYLKCIIKETLRLHPAAPLLVPRETSASFELGGYYIPPKTRVLVNAFAIQRDPSFWDRPDEFLPERFENNPVDFKGQDFQFIPFGSGRRGCPGALFGVTAVEFMIANLLYWFDWRLPDGATQEELDMSEICGMTAYKKTPLLLVPSLYSP | |
| CYP71AP1 | MSLLQWLKECSKPTLFVVTIFLVVVLKFLMKDKLKKRKLNLPPSPAKLPIIGNLHQLGNMPHISLRGLAKKYGPIIFLQLGEIPTVVISSAGLAKEVLKTHDLVLSSRPQLFSAKHLLYGCTDIAFAPYGAYWRNIRKICILELLSAKRVRSYSYVREEEVARLIRRIAESYPGITNLSSMIALYTNDVLCRVALGRDFSGGGEYDRHGFQKMFDDFQALLGGFSLGDYFPSMEFVHSLTGMKSKLQYTFRRFDQFFDEVIAEHRSSKGKQEEKKDLVDVLLDIQKDGSSEIPLTMDNIKAVILDMFAGGTDTTFITLDWAMTELIMNPHVMEKAQAEVRSVVGDRRVVQESDLPRLNYMKAVIKEILRLHPAAPVLLPRESLEDVIIDGYNIPAKTRIYVNVWGMGRDPELWENPETFEPERFMGSGIDFKGQDFELIPFGAGRRICPAITFGIATVEIALAQLLHSFDWKLPPGLEAKDIDNTEAFGISMHRTVPLHVIAKPHFD | |
| CYP71AQ1 | MILHPYSLACLLFIFVTKWFFFNSARNKNLPPSPLKIPVVGNLLQLGLYPHRSLQSLAKRHGPLMLLHLGNAPTLVVSSADGAHEILRTHDVIFSNRPDSSIARRLLYDYKDLSLALYGEYWRQIRSICVAQLLSSKRVKLFHSIREEETALLVQNVELFSSRSLQVDLSELFSELTNDVVCRVSFGKKYREGGSGRKFKKLLEEFGAVLGVFNVRDFIPWLGWINYLTGLNVRVEWVFKEFDRFLDEVIEEFKANRVGVNEDKMNFVDVLLEIQKNSTDGASIGSDSIKAIILDMFAAGTDTTHTALEWTMTELLKHPEVMKKAQDEIRRITGSKISVTQDDVEKTLYLKAVIKESLRLHPPIPTLIPRESTKDVKVQGYDILAKTRVIINAWAIGRDPSSWENPDEFRPERFLESAIDFKGNDFQFIPFGAGRRGCPGTTFASSVIEITLASLLHKFNWALPGGAKPEDLDITEAPGLAIHRKFPLVVIATPHSF | |
| CYP71AR1 | MAELINTETLSLVLLAVFLILFYIWSSSTSTTRNSPPSPPKLPIIGNLHQLLGSPGTPPHRALQALSKLHGPLMLLHFGSFPVLVVSSAEAAREIMKTHDLAFASRPRTTAFEKLLYNYKDVAAAPYGDYWRQVKSICVLNLLSAKKVRSFRTLREEETRSMINNIKETSRRGEVVDVRKMVMGLTNDVVSRAALGKKYYNDGEFKELITEFTELAGSIHIGDYIPSLGWLSRLGGLDAKLVSLAKRYDAFLDTVLQEHIDRSSETTSNRNDKSVDDQNEDNKDFVDVLLDIQRENSLHFPLNRISIKAVVQDVFLAGTDTTSTLLEWAMAEILRHPRVMSKLQKELRSVKKGEEEILTEDDMVDMHYLKAVIKEALRLHPPFTLLLPKMSIQDVKIKGYDIKANTQVLVNAWQIGRDPESFSYKPEEFEPEGFLEVNSGLSYKGTDFEFIPFGAGRRICPGIQFATTVNEIGLANLLHKFDWKLPGGVRNEDLDMNESSGLTIHKKHPLKAVAIPYSSA | |
| CYP71AS1 | LLLIPLLLILKKLKAQNKQQLPPSPPKLPVIGNFHQLGELPHQSLWQLSKKYGPVMLLKLGRVPLVVISSAEAARDVLKVHDLDCCSRPPLIGSGKFTYNYSDIAFSPYSDYWRELRKISVLEVFSLRRVQSFGFIREEEVALLMNSISESSSSASPVDLSEKMFALTGSIVFRMAFGRRFRGSNFDNHSFQELVHAVESLLGGFAAAECFPYVGWIIDRLNGYHAKLERVFQELDTLFQQIIDDHLKPAETTKQEHVQQDIIDVMLKIERDQAESHESEAWLTKNHIKAVLLNIFLGGVDTSAITVIWAMAELCKNPRLMKKAQAEIRNHIGNKGRVTEADIDQLQYLKMVIKETLRLHPPAPLLIARDTLYRFKVNGYDIYPKTLIQVNAWAIGRDSKYWESPEEFIPERFIDKPVDVKGQDFEYLPFGSGRRICPGINLGLIMSELALANLLYCFDWKLPNGREEDCVNMNMEEATGVSLTLSKKTPLILVPVNYLQ | |
| CYP71AT1 | MILFLLFVALPIILIFVLPKAKKGAKNTQPPGPVGLPFIGNLHQFDSLTPHIYFWKLSKKYGKIFSLKLGSTPMVVVSSAKLAKEVLKTQDLVYCSRPSILGQQKLSYNGRDIVFAPYNDYWREMRKISVLHLFSLKKVQLYKPIREDEVSRMIKKISLHAASSQITNLSNLMISLISTIICRFAFGVRFDDEAHERKRFDYLLAETQAMMASFFVSDVFPFLGWIDKLTGLTDRLKKNLKELDEFYEELIEQHQNPNRPKSMEGDIVDLLLQLKKEKSIPIDLTLEDIKGLLMNVLVAGSDTSAAGIVWTMTALMKNPKAMKKVQEEIRKSIGNKGIVNEDDIQNMPYFKAVIKESFRLYPPVPLLVPRESMKKSTLEGYEIQAGTIVHVNSWAIARDPEIWENPEEFIPERFLNSDIDYKGQNYELIPFGAGRRGCPGMTLGVASMELALSNLLYAFDWELPHGMKKEDIDTNVRPGITMHKKNDLCLIPKSYF | |
| CYP71AU2 | MISLFAVFPFLIFLGFILLSFFQLSSKKSKKNLPPSPPKLPLIGNFHQLGQQPHRSLQKLTNEYGPMMMLQFGSVPVLIASSAEAASHIMKTQDLGFANKPKSIIPSKLFFGPKDVAFTPYGEYWRNARSVCMLQLLNNKRVQSFSKIREEETSLLLRKINHSIGNSQVVDLTDLFVSMTNDVLCRVALGRKYCDGEEGKKFKSLLLEFVELLGVFNIGDYMPWLAWVNRFNGLNAKVDKVAEEFSAFLEGVIEEHKEKIKTDEKEEGSADFVDILLQVQKENKSGFNVEMDSIKAIIMDMFSAGTDTTSTLLEWTMNELIRNPNALRKLRDEVRKVTQGKSDVTEDDLEHMPYLNAVMKESLRLHSPVPLLPREAIKDTKVLGYDVAAGTQVFVCPWAISRDPTIWENPEEFQPERFLDSCVDYKGLHFELIPFGAGRRGCPGITFAKVVNELALARMLFHFEFSLPNGAKAEDLDVDEALGITVRRKFPLLVVATPRI | |
| CYP71AV | MALSLTTSIALATILFFVYKFATRSKSTKNSLPEPWRLPIIGHMHHLIGTIPHRGVMDLARKYGSLMHLQLGEVSTIVVSSPKWAKEILTTYDITFANRPETLTGEIVAYHNTDIVLAPYGEYWRQLRKLCTLELLSVKKVKSFQSLREEECWNLVQEIKASGSGRPVNLSENIFKLIATILSRAAFGKGIKDQKEFTEIVKEILRQTGGFDVADIFPSKKFLHHLSGKRARLTSIHQKLDNLINNLVAEHTVKTSSKTNETLLDVLLRLKDSAEFPLTADNVKAIILDMFGAGTDTSSATIEWAISELIKCPRAMEKVQVELRKALNGKERIHEEDIQELSYLNLVIKETLRLHPPLPLVMPRECRQPVNLAGYDIPNKTKLIVNVFAINRDPEYWKDAETFIPERFENSSTTVMGAEYEYLPFGAGRRMCPGAALGLANVQLPLANILYHFNWKLPNGASYDQIDMTESFGATVQRKTELLLVPSF | |
| CYP71AV | MELSITTSIALATIVFFLYKLATRPKSTKKQLPEASRLPIIGHMHHLIGTMPHRGVMDLARKHGSLMHLQLGEVSTIVVSSPKWAKEILTTYDITFANRPETLTGEIIAYHNTDIVLAPYGEYWRQLRKLCTLELLSVKKVKSFQSIREEECWNLVKEVKESGSGKPINLSESIFTMIATILSRAAFGKGIKDQREFTEIVKEILRQTGGFDVADIFPSKKFLHHLSGKRARLTSIHKKLDNLINNIVAEHHVSTSSKANETLLDVLLRLKDSAEFPLTADNVKAIILDMFGAGTDTSSATVEWAISELIRCPRAMEKVQAELRQALNGKEKIQEEDIQDLAYLNLVIRETLRLHPPLPLVMPRECREPVNLAGYEIANKTKLIVNVFAINRDPEYWKDAEAFIPERFENNPNNIMGADYEYLPFGAGRRMCPGAALGLANVQLPLANILYHFNWKLPNGASHDQLDMTESFGATVQRKTELLLVPSF | |
| CYP71AV1 | MKSILKAMALSLTTSIALATILLFVYKFATRSKSTKKSLPEPWRLPIIGHMHHLIGTTPHRGVRDLARKYGSLMHLQLGEVPTIVVSSPKWAKEILTTYDITFANRPETLTGEIVLYHNTDVVLAPYGEYWRQLRKICTLELLSVKKVKSFQSLREEECWNLVQEIKASGSGRPVNLSENVFKLIATILSRAAFGKGIKDQKELTEIVKEILRQTGGFDVADIFPSKKFLHHLSGKRARLTSLRKKIDNLIDNLVAEHTVNTSSKTNETLLDVLLRLKDSAEFPLTSDNIKAIILDMFGAGTDTSSSTIEWAISELIKCPKAMEKVQAELRKALNGKEKIHEEDIQELSYLNMVIKETLRLHPPLPLVLPRECRQPVNLAGYNIPNKTKLIVNVFAINRDPEYWKDAEAFIPERFENSSATVMGAEYEYLPFGAGRRMCPGAALGLANVQLPLANILYHFNWKLPNGVSYDQIDMTESSGATMQRKTELLLVPSF | |
| CYP71AV1 | MKSILKAMALSLTTSIALATILLFVYKFATRSKSTKKSLPEPWRLPIIGHMHHLIGTTPHRGVRDLARKYGSLMHLQLGEVPTIVVSSPKWAKEILTTYDITFANRPETLTGEIVLYHNTDVVLAPYGEYWRQLRKICTLELLSVKKVKSFQSLREEECWNLVQEIKASGSGRPVNLSENVFKLIATILSRAAFGKGIKDQKELTEIVKEILRQTGGFDVADIFPSKKFLHHLSGKRARLTSLRKKIDNLIDNLVAEHTVNTSSKTNETLLDVLLRLKDSAEFPLTSDNIKAIILDMFGAGTDTSSSTIEWAISELIKCPKAMEKVQAELRKALNGKEKIHEEDIQELSYLNMVIKETLRLHPPLPLVLPRECRQPVNLAGYNIPNKTKLIVNVFAINRDPEYWKDAEAFIPERFENSSATVMGAEYEYLPFGAGRRMCPGAALGLANVQLPLANILYHFNWKLPNGVSYDQIDMTESSGATMQRKTELLLVPSF | |
| CYP71AV3 | MEVSLTTSIALATIVFFLYKLLTRPTSSKNRLPEPWRLPIIGHMHHLIGTMPHRGVMDLARKYGSLMHLQLGEVSAIVVSSPKWAKEILTTYDIPFANRPETLTGEIIAYHNTDIVLAPYGEYWRQLRKLCTLELLSVKKVKSFQSLREEECWNLVQEIKASGSGTPFNLSEGIFKVIATVLSRAAFGKGIKDQKQFTEIVKEILRETGGFDVADIFPSKKFLHHLSGKRGRLTSIHNKLDSLINNLVAEHTVSKSSKVNETLLDVLLRLKNSEEFPLTADNVKAIILDMFGAGTDTSSATVEWAISELIRCPRAMEKVQAELRQALNGKERIKEEEIQDLPYLNLVIRETLRLHPPLPLVMPRECRQAMNLAGYDVANKTKLIVNVFAINRDPEYWKDAESFNPERFENSNTTIMGADYEYLPFGAGRRMCPGSALGLANVQLPLANILYYFKWKLPNGASHDQLDMTESFGATVQRKTELMLVPSF | |
| CYP71AV4 | MELSLTTSIALATIVLILYKLATRPKSNKKRLPEASRLPIIGHMHHLIGTMPHRGVMELARKHGSLMHLQLGEVSTIVVSSPKWAKEILTTYDITFANRPETLTGEIIAYHNTDIVLAPYGEYWRQLRKLCTLELLSVKKVKSFQSIREEECWNLVKEVKESGSGKPISLSESIFKMIATILSRAAFGKGIKDQREFTEIVKEILRQTGGFDVADIFPSKKFLHHLSGKRARLTSIHKKLDTLINNIVAEHHVSTSSKANETLLDVLLRLKDSAEFPLTADNVKAIILDMFGAGTDTSSATVEWAISELIRCPRAMEKVQAELRQALNGKEQIHEEDIQDLPYLNLVIRETLRLHPPLPLVMPRECREPVNLAGYEIANKTKLIVNVFAINRDPEYWKDAEAFIPERFENNPNNIMGADYEYLPFGAGRRMCPGAALGLANVQLPLANILYHFNWKLPNGASHDQLDMTESFGATVQRKTELILVPSF | |
| CYP71AV5 | MELSFTTSIAVATIVFVLFKLATRPKSNKKLLPEPWRLPIIGHMHHLIGTMPHRGVMDLARKYGSLMHLQLGEVSTIVVSSPKWAKEILTTHDITFANRPETLTGEIIAYHNTDIVLAPYGEYWRQLRKLCTLELLSVKKVKSFQSLREEECWNLVQEVKESGSGRPVDLSENIFKMIATILSRAAFGKGIKDQKEFTEIVKEILRQTGGFDVADIFPSKKFLHHLSGKRARLTSIHKKLDNLINNIVAEHPGNNSSKSNETLLDVMLRLKDSVEFPLTADNVKAIILDMFGAGTDTSSATVEWAISELIRCPRAMEKVQAELRQALKGKDKVKEEDIQDLSYLDLVIKETLRLHPPLPLVMPRECRQPVNLAGYDIANKTKLIVNVFAINRDPEYWKDAESFIPERFENSPITVMGAEYEYLPFGAGRRMCPGAALGLANVQLPLANILYHFNWKLPNGASHDQLDMTESFGATVQRKTHLVLVPSF | |
| CYP71AV6 | MEVSLTTSIALATIVFFLYKLLTRPTSSKNRLPEPWRLPIIGHMHHLIGTMPHRGVMDLARKYGSLMHLQLGEVSAIVVSSPKWAKEILTTYDIPFANRPETLTGEIIAYHNTDIVLAPYGEYWRQLRKLCTLELLSVKKVKSFQSLREEECWNLVQEIKASGSGTPFNLSEGIFKVIATVLSRAAFGKGIKDQKQFTEIVKEILRETGGFDVADIFPSKKFLHHLSGKRGRLTSIHNKLDSLINNLVAEHTVSKSSKVNETLLDVLLRLKNSEEFPLTADNVKAIILDMFGAGTDTSSATVEWAISELIRCPRAMEKVQAELRQALNGKERIKEEEIQDLPYLNLVIRETLRLHPPLPLVMPRECRQAMNLAGYDVANKTKLIVNVFAINRDPEYWKDAESFNPERFENSNTTIMGADYEYLPFGAGRRMCPGSALGLANVQLPLANILYYFKWKLPNGASHDQLDMTESFGATVQRKTELMLVPSF | |
| CYP71AV7 | MELTLTTSLGLAVFVFILFKLLTGSKSTKNSLPEAWRLPIIGHMHHLVGTLPHRGVTDMARKYGSLMHLQLGEVSTIVVSSPRWAKEVLTTYDITFANRPETLTGEIVAYHNTDIVLSPYGEYWRQLRKLCTLELLSAKKVKSFQSLREEECWNLVKEVRSSGSGSPVDLSESIFKLIATILSRAAFGKGIKDQREFTEIVKEILRLTGGFDVADIFPSKKILHHLSGKRAKLTNIHNKLDSLINNIVSEHPGSRTSSSQESLLDVLLRLKDSAELPLTSDNVKAVILDMFGAGTDTSSATIEWAISELIRCPRAMEKVQTELRQALNGKERIQEEDIQELSYLKLVIKETLRLHPPLPLVMPRECREPCVLAGYEIPTKTKLIVNVFAINRDPEYWKDAETFMPERFENSPINIMGSEYEYLPFGAGRRMCPGAALGLANVELPLAHILYYFNWKLPNGARLDELDMSECFGATVQRKSELLLVPTAYKTANNSA | |
| CYP71AV8 | MEISIPTTLGLAVIIFIIFKLLTRTTSKKNLLPEPWRLPIIGHMHHLIGTMPHRGVMELARKHGSLMHLQLGEVSTIVVSSPRWAKEVLTTYDITFANRPETLTGEIVAYHNTDIVLAPYGEYWRQLRKLCTLELLSNKKVKSFQSLREEECWNLVKDIRSTGQGSPINLSENIFKMIATILSRAAFGKGIKDQMKFTELVKEILRLTGGFDVADIFPSKKLLHHLSGKRAKLTNIHNKLDNLINNIIAEHPGNRTSSSQETLLDVLLRLKESAEFPLTADNVKAVILDMFGAGTDTSSATIEWAISELIRCPRAMEKVQTELRQALNGKERIQEEDLQELNYLKLVIKETLRLHPPLPLVMPRECREPCVLGGYDIPSKTKLIVNVFAINRDPEYWKDAETFMPERFENSPITVMGSEYEYLPFGAGRRMCPGAALGLANVELPLAHILYYFNWKLPNGKTFEDLDMTESFGATVQRKTELLLVPTDFQTLTAST | |
| CYP71AX1 | YFLLVPLLAFIYFLHQCFFSPSNTQKRLLPPSPTKLPIIGNLHQLGSLPHRSLHKLSKKYGPVMLLHLGSKPVIIASSVDAARDIMKTHDLVWSNRPKSSMADGLFYGSKDVTFSPYGEYWRQIRSITVLHLLSNKRVQSYRRVREEEISNMIDKIRQKCDSVIDLRDVFSCLANNIISRVNIGRTYNEGECGIAVKSLIEELLILIGTFNIGDYTPWFKWVNKIKGVDSRVKKVAKDLDAFIESVIEERLIRNKKAECSAVEAKDFLGVLLEIQDGKETGFPLQRDSLKALLLDAFVAGTDSTYTVLEWTMTELLRHPRVMTKLEDEVRELGQGKTEITEDDLRNMHYLKAVIKESLRLHAPVPLLVARESMEEVKLLDYDIPAKTEVLINAWSIGRDPLLWDHPEEYMPERFLSSDIDVKGLNFELIPFGAGRRGCPGIPFAIMVNELALANLVYKFNFALPKGIKGEDLDMTECNGLAVRRKSPLLVVATPKSMV | |
| CYP71AY1 | ARVNFSLTSPIFLLLSSLFLIILLNKLMRGNKIQKGKKLPPGPKKIAIIGNLPSNGRFTSLIVFLNNLAEKYGPIMHLRIGQLSAVIISSAEKAKEILNTHGVRVADRPQTTVAKIMLYNSLGVTFAPYGDYLKQLRQIYAMELLSPKTVKSFWTIMDDELSTMITSIKSEVGQPMILHDKMMTYLYAMLCRATVGSVCNGRETLIMAAKETSALSASIRIEDLFPSVKILPVISGLKSKLTNLLKELDIVLEDIISAREKKLLSQPQQPLMLDEEDMLGVLLKYKNGKGNDTKFRVTNNDIKAIVFELILAGTLSSAAIVEWCMSELMKNPELLKKAQDEVRQVLKGKKTISGSDVGKLEYVKMVVKESVRLHPPAPLLFPRECREEFEIDGMTIPKKSWVIINYWAIGRDPKIWPNADKFEPERFSNNNIDFYGSNFELIPFGAGRRVCPGILFGTTNVELLLAAFLFHFDWELPGGMKPEELDMNELFGAGCIRENPLCLIPSISTVVEGN | |
| CYP71AZ1 | MQMDAVVILLILAFPIASVYVLFYHKKRVDGLSEPPGPPGLPFIGNFYQLYKAPCIHEYLCTLSKRYGSLMTLRMGSVPILVVSSPKMAKEVLKTQDLAYCSRPMMTGMQKLSYNGLDVAFSPYSEHWRQVRKFCTLELFTQKRAQIDFRHVHEQEVSRMIARLSETAAASKDVNAFECFSNLATSIISRVAFGKRHDEDGIGKERLQRMLSELDTMLSVYFVSDFFPMFGWIDSLTGMRARLDRTFKEMDMFYEELIDDHLKPDRPESLTEDIIDVMLKNKGCSSSSLTKDTMKAILLNVFNGGTGTSASLLVWAMTALMRNRGVMKKVQEEIRSVIGKKGNVDEDDIQNLPYLRAVVKETMRLYPTGALLIPRKTIESSIIGEDKDHMYMIKPKTLVYVSMWAIGRDPEIWKNPMKFVPERFLERHDINYQGQQFEYIPFGAGRRICPGIHLGLTTVELALANLLYTFNWEPPVGTRFEDINDETVNGITLQKKNALYIRPKTYMFS | |
| CYP71B2 | MTILLCFFLVSLLTIVSSIFLKQNKTSKFNLPPSPSSLPIIGNLHHLAGLPHRCFHKLSIKYGPLVFLRLGSVPVVVISSSEAAEAVLKTNDLECCSRPKTVGSGKLSYGFKDITFAPYGEYWREVRKLAVIELFSSKKVQSFRYIREEEVDFVVKKVSESALKQSPVDLSKTFFSLTASIICRVALGQNFNESGFVIDQDRIEELVTESAEALGTFTFSDFFPGGLGRFVDWLFQRHKKINKVFKELDAFYQHVIDDHLKPEGRKNQDIVTLILDMIDKQEDSDSFKLNMDNLKAIVMDVFLAGIDTSAVTMIWAMTELIRNPRVMKKAQGSIRTTLGLKKERITEEDLGKVEYLNHILKETFRLHPALPFVVPRETMSHIKIQGYDIPPKTQIQLNVWTIGRDPKRWNDPEEFNPERFANSSVDFRGQHFDLLPFGSGRRICPGMPMAIASVELALMNLLYYFDWSMPDGTKGEDIDMEEAGNISIVKKIPLQLVPVQRY | |
| CYP71B3 | MSILLYFFFLPVILSLIFMKKFKDSKRNLPPSPPKLPIIGNLHQLRGLFHRCLHDLSKKHGPVLLLRLGFIDMVVISSKEAAEEVLKVHDLECCTRPKTNASSKFSRDGKDIAFAPYGEVSRELRKLSLINFFSTQKVRSFRYIREEENDLMVKKLKESAKKKNTVDLSQTLFYLVGSIIFRATFGQRLDQNKHVNKEKIEELMFEVQKVGSLSSSDIFPAGVGWFMDFVSGRHKTLHKVFVEVDTLLNHVIDGHLKNPEDKTNQDRPDIIDSILETIYKQEQDESFKLTIDHLKGIIQNIYLAGVDTSAITMIWAMAELVKNPRVMKKAQEEIRTCIGIKQKERIEEEDVDKLQYLKLVIKETLRLHPPAPLLLPRETMADIKIQGYDIPRKTILLVNAWSIGRNPELWENPEEFNPERFIDCPMDYKGNSFEMLPFGSGRKICPGIAFGIATVELGLLNLLYYFDWRLAEEDKDIDMEEAGDATIVKKVPLELVPIIH | |
| CYP71B40v3 | MALYVVPLWLPLILLLALLLLFMKKMEVKRQSEQLLPPSPPKLPILGNLHQLGSLPHQSLWQLSKKYGPVMLIRLGRIPTVVISSAEAAREVLKVHDLAFCSRPLLAGTGRLTYNYLDIAFSPYSDHWRNMRKIVTLELFSLKRVQSFRFIREEEVSLLVNFISESSALAAPVDLTQKLYALVANITFRMAYGFNYRGTSFDRDKFHEVVHDTKAVAGSISADESIPYLGWIVDRLTGHRARTERVFHELDTFFQHLIDNHLKPGRIKEHDDMVDVLLRIEKEQTELGASQFTKDNIKAILLNLFMAGVDTSSLTVNWAMAELVRNPRVMKKVQDEVRKCVGNKGRVTESDIDQLEYLRMVIKETLRLHPPGPLLIPRETMSHCKVSGHNIYPKMLVQINVWAIGRDPRYWKDPEEFFPERFLDRSIDYKGQSFEYLPFGSGRRICPGMHMGSITMEIILANLLYCFDWVFPDGMKKEDINMEEKAGVSLTTSKKTPLILVPVNYLQ | |
| CYP71B41v2 | MALYVVPLWLPLILLLALLLLFMKKMEVKRQSEQLLPPSPPKLPILGNLHQLGSLPHQSLWQLSKKYGPVMLIRLGRIPTVVISSAEAAREVLKVHDVAFCSRPLLAGTGRLTYNYLDIAFSPYSDHWRNMRKILTLELFSLKRVQSFRFIREEEVSLLVNFISESSALAAPVDLTQKLYALVANITFRMAYGFNYRGTSFDRDKFHEVVHDTVAVVGSISADESIPYLGWIVDRLTGHRARTERVFHEVDTFFQHLIDNHLKPGRIKEHDDMVDVLLRIEKEQTELGASQFTKDNIKAILLNLFLGGVDTSSLTVNWAMAELVRNPRVMKKVQDEVRKCVGNKGRVTESDIDQLEYLRMVIKETLRLHPPAPLLIPRETMSHCKVSGHNIYPKMLVQINVWAIGRDPTYWKDPEEFFPERFLDSSIDYKGQSFEYLPFGSGRRICPGMHMGFITMEIILANLLFCFDWVFPDGMKKEDINMEEKAGVSLTTSKKTPLILVPVNYLQ | |
| CYP71BA1 | MEAISLFSPFFFITLFLGFFITLLIKRSSRSSVHKQQVLLASLPPSPPRLPLIGNIHQLVGGNPHRILLQLARTHGPLICLRLGQVDQVVASSVEAVEEIIKRHDLKFADRPRDLTFSRIFFYDGNAVVMTPYGGEWKQMRKIYAMELLNSRRVKSFAAIREDVARKLTGEIAHKAFAQTPVINLSEMVMSMINAIVIRVAFGDKCKQQAYFLHLVKEAMSYVSSFSVADMYPSLKFLDTLTGLKSKLEGVHGKLDKVFDEIIAQRQAALAAEQAEEDLIIDVLLKLKDEGNQEFPITYTSVKAIVMEIFLAGTETSSSVIDWVMSELIKNPKAMEKVQKEMREAMQGKTKLEESDIPKFSYLNLVIKETLRLHPPGPLLFPRECRETCEVMGYRVPAGARLLINAFALSRDEKYWGSDAESFKPERFEGISVDFKGSNFEFMPFGAGRRICPGMTFGISSVEVALAHLLFHFDWQLPQGMKIEDLDMMEVSGMSATRRSPLLVLAKLIIPLP | |
| CYP71BC1 | MTMKISENMLLLFSQSSANQWLLALGILSFPILYLFLLQRWKKKGIEGAARLPPSPPKLPIIGNLXQLGKLPHRSLSKLSQEFGPVLLLQLGRIPTLLISSADMAKEVLKTHDIDCCSRAPSQGPKRLSYNFLDMCFSPYSDYWRAMRKVFVLELLSAKRAHSLWHAWEVEVSHLISSLSEASPNPVDLHEKIFSLMDGILNMFAFGKNYGGKQFKNEKFQDVLVEAMKMLDSFSAEDFFPSVGWIIDALTGLRARHNKCFRNLDNYFQMVVDEHLDPTRPKPEHEDLVDVLLGLSKDENFAFHLTNDHIKAILLNTFIGGTDTGAVTMVWAMSELMANPRVMKKVQAEVRSCVGSKPKVDRDDLAKLKYLKMVVKETFRMHPAAPLLIPHRTRQHCQINANGCTYDIFPQTTILVNAFAIGRDPNSWKNPDEFYPERFEDSDIDFKGQHFELLPFGAGRRICPAIAMAVSTVEFTLANLLYCFDWEMPMGMKTQDMDMEEMGGITTHRKTPLCLVPIKYGCVE | |
| CYP71BE1 | MEFPSSFLFPFLLFLFILFKVSKKSKPQISIPKRPPGPWKLPLIGNLHQLVGSLPHHSLRDLAKKYGPLMHLQLGQVSMLVVSSPEIAKEVMKTHDINFAQRPHLLATRIATYDSTDVAFSPYGDYWRQLRKICVVELLSAKRVKSFQVIRKEEVSKLIRIINSSSRFPINLRDRISAFTYSVISRAALGKECKDHDPLTAALGEITKLASGFCLADLYPSVKWIPLVSGVRHKLEKVQQRIDGILQIVVDEHRERMKTTTGKLEEEKDLVDVLLKLQQDGDLELPLTDDNIKAVILDIFGGGGDTVSTAVEWTMAEMMKNPEVMKKAQAEVRRVFDGKGNVDEAGIDELKFLKAVISETLRLHPPFPLLLPRECREKCKINGYEVPVKTRVVINAWAIGRYPDCWXEAERFYPERFLDSSIDYKGADFGFIPFGSGRRICPGILFGIPVIELPLAQLLFHFDWKLPNGMRPEDLDMTEVHGLAVRKKHNLHLIPIPYSPLTVG | |
| CYP71BE5 | MELQFSFFPILCTFLLFIYLLKRLGKPSRTNHPAPKLPPGPWKLPIIGNMHQLVGSLPHRSLRSLAKKHGPLMHLQLGEVSAIVVSSREMAKEVMKTHDIIFSQRPCILAASIVSYDCTDIAFAPYGGYWRQIRKISVLELLSAKRVQSFRSVREEEVLNLVRSVSLQEGVLINLTKSIFSLTFSIISRTAFGKKCKDQEAFSVTLDKFADSAGGFTIADVFPSIKLLHVVSGMRRKLEKVHKKLDRILGNIINEHKARSAAKETCEAEVDDDLVDVLLKVQKQGDLEFPLTMDNIKAVLLDLFVAGTETSSTAVEWAMAEMLKNPRVMAKAQAEVRDIFSRKGNADETVVRELKFLKLVIKETLRLHPPVPLLIPRESRERCAINGYEIPVKTRVIINAWAIARDPKYWTDAESFNPERFLDSSIDYQGTNFEYIPFGAGRRMCPGILFGMANVELALAQLLYHFDWKLPNGARHEELDMTEGFRTSTKRKQDLYLIPITYRPLPVE | |
| CYP71BE52 | MEIHIPSLVLCISFFIFFKIVSKLKTKTSNRKHLPLPPGPWKLPLIGNLHNLVGALPHHTLRRLSRKFGPMMSLQLGELSAVIISSADAAKEIMKTHDLNFASRPQVAAADIIGYGSTNITFSPYGGHWRQLRKICTLELLSAKRVQSFRPLRERVFVDLCRRFADHGSSAVNFSEEFMSATYTLISRAVLGEEAEQHEGLLPNVKEMPELTAGFDISEVFPSVGLFKVMSRLRKRIVAVHKDTDRILDDVIHQHRAAKSEEHKDLLDVLLQLQEDGLELPLTDENIKSVLVDMLVAGSETSSTVIEWAMAEMLKNPRILEKAQEEVRRVFDKEGTVDESHIHELKYLKSVVKETLRVHPPAPLILPRICGETCEINGYEIPAETKIIVNAWAVNRDPKYWEDSDCFKPERFLDNLVDFRGNHFQYIPFGAGRRMCPGIGFGLANVELPLAMFMYHFDWELDGGMKPQDLDMEEKFGASAKKLKDLFLIPAIKRTLPTK | |
| CYP71BF1 | MWNSLWFTVLFIFLFRYFFLRCYSSSKKNSPPSPPKLPIVGDLHRLGSSPHRSLRALAQQYGPFMLLRFGSVPVLVISSAHAALDVMKTHDNIFSSRFKSSVFDKLVYNCKDVVLAPYGEYWRQMKSICVIHLLSSKKVQCFQKVREEETMILTKKIQESYCSPMNLSETFTVLANDILCRVAFGRKYGGDEENGKKLKELLTRFAQLTGTVDIGDYIPWLSWVSCVNGLNTKLEKLAKELDDLFEGIVEEHVNHLKNKSNTSDYGDVQDTDSKDFVDVLLWIQRENTIGFPIDRVTIKALILDMFIAGTDTTSTTLEWAMTELLRHPKVMKKLRNEVRTIAGDKSNIIEEDLGKMKYLKAVIKEVLRLHPPLPLLMPRESVKDANVRGYDITAGTQVLINAWAIGRDPDSWEEAEEFKPERFLNSCIDFKGHDFQLIPFGAGRRGCPGIHFATILIEMILANVLHKCDWKPMPYRGAKEHGIDLTESAGISVHRKFPLIAIPSPPRF | |
| CYP71BG1 | MEASILQLLLLLSLTSCTILFYKIRRWRRPPSPPSLPIIGHLHLLTDMPHHTFFHLSQKLGPIIHLQLGQIPTLIISSPRLAELILKTNDHIFCSRPQIIAAQYLSFGCSDITFSPYGPYWRQARKICVTELLSSKRVNSFQFIRNEEINRMIQLISSHFDSELSSELDLSQVFFALANDILCRVAFGKRFIDDRLKDKDLVSVLTETQALLAGFCLGDFFPDWEWVNWLSGMKKRLMNNLKDLGEVCDEIIDEHLMKKRDDDQNGDGSEDFVDVLLRVQKRDDLQVPITDDNLKALILDMFVAGTDTSAATLEWTMTELARHPSVMKKAQDEVREIAANKGKVEEFDLQHLHYMKAVIKETMRLHPPVPLLVPRESIEKCTLDDYEIPAKTRVLINTYAIGRDPEYWNNPLDYNPERFMEKDIDFRGQDFRFLPFGGGRRGCPGYALGLATIELSLARLLYHFDWKLPTGVEAQDVNLSEIFGLATRKRVALKLVPTINKLYLLSD | |
| CYP71BJ1 | MLSSLKDFFVLLLPFFIGIAFIYKLWNFTSKKNLPPSPRRLPIIGNLHQLSKFPQRSLRTLSEKYGPVMLLHFGSKPVLVISSAEAAKEVMKINDVSFADRPKWYAAGRVLYEFKDMTFSPYGEYWRQARSICVLQLLSNKRVQSFKGIREEEIRAMLEKINQASNNSSIINGDEIFSTLTNDIIGRSAFGRKFSEEESGSKLRKVLQDLPPLLGSFNVGDFIPWLSWVNYLNGFEKKLNQVSKDCDQYLEQVIDDTRKRDEENGANNNGGNHGNFVSVLLHLQKEDVKGFPSEKGFLKAIILDMIVGGTDTTHLLLHWVITELLKNKHVMTKLQKEVREIVGRKWEITDEDKEKMKYLHAVIKEALRLHPSLPLLVPRVAREDINLMGYRVAKGTEVIINAWAIARDPSYWDEAEEFKPERFLSNNFDFKGLNFEYIPFGSGRRSCPGSSFAIPIVEHTVAHLMHKFNIELPNGVSAEDFDPTDAVGLVSHDQNPLSFVATPVTIF | |
| CYP71BL | MEPFTIFSLVVASLVFFACWALVAPNTSKNLPPGPPKLPIIGNIHQLKSPTPHRVLKDLAKKYGPIMHLQLGQVSTVVVSTPRLAQEIMKTNDISFADRPTTTTSQIFFYKAQDIGWAPYGEYWRQMKKICTLELLSAKKVRSFSSIREEELTRIRKILEFKAGTPINYTEMTIEMVNNVICKATLGDCCKDQALLIELLYDVLKTLSAFNLASYYPRLQFLNVISGKKAKWLKMQKRLDDIMEDILKEHRAKGRAKNSDQEDLVDVLLRIKDTGGLDINVTDEHVKAVVLDMLTAGTDTSSTTLEWAMTELMRNPDMMKRAQEEVRSVVKGEHVTETDLQSLHYLKLIVKETMRLHAPTPLLVPRECRQDCNVDGYDIPAKTKVLVNAWACGVDPGSWENPDSFIPERFENSSINFMGADFQYIPFGAGRRICPGLTFGLSMVEYPLAHFLYHFDWKLPYGMKPHELDITEITTISTSLKHHLKIVPFPKSSLAK | |
| CYP71BL1 | MELFTIFSIVVSSLILFTFWSLKVPKNLPPGPPKLPIIGNIHLLDKIAPHRNLRNLARKYGPIMHLRLGQVSTVVISSPRLAHEIMKTQDLSFADRPTTTTSQIFFYKASNIAWARYGNYWRQMKKICTLELLSAKKSRSFFYIREEELTRTYKFLDFSSGTPITLRDTIQEMVNNVVSRATLGDVSEDRQFIIDSTYTMLKSFNSFNLFNYYPSLSFINVISGKQAQWLKMHKEVDVILEKILREHRSRPRGKNDHEDLVDVLIRIKETGDLDMAITDDNIKAIILEMLTAGTSSSSMTIEWAFTEMMRNPKIMKKAQTEVRSVVKGDRVTEADIQNLDYTKLVIKETLRLHGVPILVPRENQEDCVVNGYDIPAKTRLLVNAWACATDPDSWEDPDSFIPERFENNSIGYSGADFEFIPFGAGRRICPGMNFGMGTVEYVVANLLLHYDWKLPDGMKPHDIDMREITGISTLPIHPLKIVPISLSK | |
| CYP71BL2 | MEPLTIVSLAVASFLLFAFWALSPKTSKNLPPGPPKLPIIGNIHQLKSPTPHRVLRNLAKKYGPIMHLQLGQVSTVVVSTPRLAREIMKTNDISFADRPTTTTSQIFFYKAQDIGWAPYGEYWRQMKKICTLELLSAKKVRSFSSIREEELRRISKVLESKAGTPVNFTEMTVEMVNNVICKATLGDSCKDQATLIEVLYDVLKTLSAFNLASYYPGLQFLNVILGKKAKWLKMQKQLDDILEDVLKEHRSKGRNKSDQEDLVDVLLRVKDTGGLDFTVTDEHVKAVVLDMLTAGTDTSSATLEWAMTELMRNPHMMKRAQEEVRSVVKGDTITETDLQSLHYLKLIVKETLRLHAPTPLLVPRECRQACNVDGYDIPAKTKILVNAWACGTDPDSWKDAESFIPERFENCPINYMGADFEFIPFGAGRRICPGLTFGLSMVEYPLANFLYHFDWKLPNGLKPHELDITEITGISTSLKHQLKIVPILKS | |
| CYP71BL3 | MEPLTIVSLVVASLFLFAFWALSPKTSKNLPPGPPKLPIIGNIHQLKSPTPHRVLRNLARKYGPIMHLQLGQVSTVVVSTPRLAREIMKTNDISFADRPTTTTSQIFFYKAQDIGWAPYGEYWRQMKKICTLELLSAKKVRSFSSIREEELSRISKVLESQAGTPINFTEMTVEMVNNVICKATLGDSCKDQATLIEVLYDVLKTLSAFNLASYYPGLQFLNVILGKKAKWLKMQKQLDDILEDVLKEHRSKGSNKSDQEDLVDVLLRVKDTGGLDFTVTDEHVKAVVLDMLTAGTDTSSATLEWAMTELMRNPHMMKRAQDEVRSVVKGNTITETDLQSLHYLKLIVKETLRLHAPTPLLVPRECRQDCNVDGYDIPAKTKILVNAWACGTDPDSWKDPESFIPERFENCPINYMGADFEFIPFGAGRRICPGLTFGLSMVEYPLANFLYHFDWKLPNGLKPHELDITEITGISTSLKHQLKIVPMIPKSIAK | |
| CYP71BN1 | MEVSSFIFTIFLIFLPLLLAIVKNHKKSKKLPPGPWKLSIIGNLHQLEISRPYITLKELSKKYGPLMHLKLGERSTIVISSYKILKELMKTSDTILSHRPELLVSETVAYNGRDIAFAPYGDYWKQMRKICTSEILSTRRIHSNYPLMEEEISRLVKNIKESSSKGTLINVYECLNSLSCAIICRVTVGTTCNDSDSLISTIRKITPLVGLFNISDLFPSLKFLDRYITGSNQKLLKVHHELCDRLLEEIVHEHEESIRKNNVDEEDLLHLLLRLREKESHNFQVPITRDNVKAIILDMFIGGTDTTSILVEWAMAELLKNRNMMKKAQVEVREVLKGKKKVDHIDVQNLKYLKLIVKETLRLHPPGPLAIPRESIEEIAINGYVIPNKTIALMNLYAMGRDPEYWHDPEKFMPDRFNNYVDNDVKMIKGSSNVPMEFLAFGFGKRVCPGMLFATASSELTLARLLYHFDWTLPNGMNPQDLDMTEGFGAAATMKNNLYLVATPYD | |
| CYP71C103 | MEVPHADAALLVLLASILLLMARRRFGLGNAAARAREEALNKLPSPGWRLPVIGHLHQVGPLPHVSLRHLAAEHGRDGLMLVRLGAVPTLVVSTPAAAQAVLRTHDHVFASRPHSPVAHILFYGSADVVFAPYGHHWRQVKKISTTHLLTARKVHSYRHARQHEVNLVLAKVRDAMRAGVALDMSELLNAFVFDIVCHAVAGNSFRERGLNKHFRELVEANASLIGGFNLEDHFPALVKLEIFRKIVCAKARRVNNKWDDLLDRLIDEHATPPALDEDRDFIHVLLSVQQEYNLTRDHIKAQLLIMFEAGTDTSTIVLEYAMVRLMQNPRVMAMLQAEVRSTIPKGKDTVTQDDLHGLPYLKAVIKETLRLHMPGPLMVPHLSMDECIINGYTIPSGTRTFINTYAIQRDPSNWESPEEFMPERFMEGGSAAAMDYKGNDFQYFPFGSGRRICPGINFATATIQLMLTNLMYHFDWKLPPESEEEGINMTETFGLTVHRKEKLLLVPLVPQN | |
| CYP71C113 | MGEVPSDELLLVLVSAALLVTILVMVRRSGSAARVREELLRKLPSPGGWLPVIGHLHMVGSQPHVSLGDLAAKHSRDGLMLLRLGSVPTLIVTSSNAARAVLRTHDDVFASRPHNPATDIIFYGPSDIAFCPYGDHWRQVKKIAMTHLLTANKVRSYRQAREEEACLVVAKLRDAMAAGAALDLGELLSAFSTNIVGHAVCGKSFRQKGHEKLFRELVETNSLLIGGFNVGDYFPELLKLDIIRWMVCGRARRVNKMWDDLLESLIHEHESKPAPALGDDTDYIHDLLSIQKEYNLTREQVKAQLVIMFGAGTDTSYIVMEYAMARLMQNPDLMTKLQAEVRSSIPKGKHMVIEDDLNHLAYLKAVIKETLRLHMPAPLLVPHLAMADCVINGYTIPSGTRVIVNSRAIARDPSSWESAEEFLPERFMQGGSAAAMDYKGNGFLYLPFGTGRRICPGINFAIAAIEIMLANLVYHFDWKLPPGSAERGISMTESFGLTVHRKDKLLLVPLVPQDYET | |
| CYP71C12 | MAQMLDGLRHDEQASLHAPQEASTMPTMSCSDLLLAMMCPLILLLIIFRCYAYATRSGGMLSRVPSPPGRLPVIGHMHLISSLPHKSLRDLATKHGPDLMLLHLGAVPTLVVSSARTAQAILRTHDRVFASRPYNTIADILLYGATDVAFSPYGDYWRQIKKIVTMNLLTIKKVHSYGQTRQQEVRLVMAKIVEEAATHMAIDLTELLSCYSNNMVCHAVSGKFFREEGRNQLFKELIEINSSLLGGFNLEDYFPSLARLPVVRRLLCAKAYHVKRRWDQLLDQLIDDHASKRRSSMLDNNDEESDFIDVLLSIQQEYGLTKDNIKANLVVMFEAGTDTSYIELEYAMAELIQKPQLMAKLQAEVRGVVPKGQEIVTEEQLGRMPYLKAVIKETLRLHPAAPLLVPHVSMVDCNVEGYTIPSGTRVIVNAWAIARDPSYWENAEEFMPERFLSNTMAGYNGNNFNFLPFGTGRRICPGMNFAIAAIEVMLASLVYRFNWKLPIDQAANGGIDMTETFGITIHLKEKLLLVPHLP | |
| CYP71C14 | MAVMLVPIPLLLLHQHHNHEHEHPSPVAPQPTMASYYTLLLALLCPLLLLLIKLCRAKTRDDELFDKLPSPPGRLPVIGHLHLIGSLPYVSFRELAIKHGPDLMLLRLGTVPTLVVSSARAAQAILRTNDHVFASRTYSAVTDILFYGSSDVAFSPYGEYWRQVKKIATTHLLTNKKVRSYSRARQQEVRLVMARINEAAVARTTVDLSELLNWFTNDIVCHAVSGKFFREEGQNQMFWELIQANSLLLGGFNLEDYFPNLARVTTVRRLLCAKAHNVNKRWDQLLDKLIDDHATKRSSSVLDLDNEESDFIDVLLSIQHEYGLTRDNVKAILVIMFEGGTDTAYIELEYAMAELIRKPQLMAKLQAEVRSVVPRGQEIVTEEQLGRMPYLKAVIKEMLRLHLAGPLLVPYLSIAECDIEGYTIPSGTRVFVNAWALSRDPSFWENAEEFIPERFLNSIAPDYNGNNFHFLPFGSGRRICPGINFAIATIEIMLANLVYRFDWEIPADQAAKGGIDMTEAFGLTVHRRRSSSLFLGSHKIK | |
| CYP71C15 | MELNNTEPLTASRAQAAAVFLLLPVALLLLLLRFARATTMAGDRNSELLLSKLPSPPLRLPVIGHMHLVGSLPHVSLRDLAAKHGRDGLMLVHLGSVPTLVVSSPRAAEAVLRTHDLAFASRPRAMVPDIITYGATDSCYGPYGDHFRKVRKAVTVHLLNSHKVQAYRPAREEEVRLVIAKLRGAAAMAGAPVDMTELLHSFANDLICRAVSGKFFREEGRNKLFRELIDTNASLLGGFNLEDYFPSLARTKLLSKVICVRAMRVRRRWDQLLDKLIDDHATRLVRRHDHDQQQDSDFIDILLYHQEEYGFTRDNIKAILVDMFEAGTDTSYLVLESAMVELMRKPHLLAKLKDEVRRVIPKGQEVVNEDNIVDMVYLKAVIKETLRLHPPAPLYIPHLSREDCSISGYMIPTGIRVFVNAWALGRDAKFWDMPDEFLPERFMDSNIDFKGHDFHYLPFGSGRRMCPGIHSATVTLEIMLANLMYCFNWKLPAGVKEEDIDMTEVFGLTVHRKEKLFLVPQAA | |
| CYP71C16 | MELILQLEAKTAAQAVVTVFFFFLLPLALLFYFARAAISSRDSKTRELILSKLPSPPFKLPVIGHMHLIGPLPYVSLRDLAAKHGRDGLMLVRLGSVPTLVVSSPRAAEAVLRTHDLAFASRPRSMVTDIIMYGALDSCFAPYSDHFRSVKKVVTVHLLNSKRVQAYRHVREEEVRLVMARLRGAAAAAAAVDLSQTLQFFANDLICRAVSGKFLCEQGRNKVFRDLMEANSNLLGGFNLEAYFPGLARMPLISKLICARAIRIRRRWDQLLDMLIDDHVASARDRAKNDDDDFIHVLLSLQDEYGFTRDHIKAISIDMFEAGTDTSHLVLEYAMVELTRKPHILTKLQDEVRRITPKGQHMVTEDDIVGMVYLKAVIKETLRLHAPGGFTIPHLAREDCNVDGYMIPAGTRVLINLWALSRDANYWDKPDEFLPERFMDGSNKNTDFKGQDFQFLPFGSGRRMCPGIHSGKVTLEIMLANLVYCFNWKLPSGMKKEDIDMTDVFGLAIHRKEKLFLVPQIANY | |
| CYP71C17 | MVVQLMLFFHDKFMAPMAEEPLPFVLIMIIILLLLVLLHYYLSASTRRSSAASKSNDDVLPPSPPRLPVIGHMHLVGSNPHVSLRDLAEKHAADGFMLLQLGQVRNLVVSSPRAAEAVLRAHDHVFASRPRSAIADILAYGSSNISFSPYGDYWRKARKLVAAHLLSPKKVQSLRRGREEEVGIAVAKLHEAAAAGAAVDMRELLGSFTNDVLCRAVCGKSSFRREGRNKLFMELAAGNADQYAGFNLEDYFPSLAKVDLLRRVVSADTKKLKEKWDSVLGDIVSEHEKKSSLRRDDQVQMDDDRDDDQEEQESDFVDILLDRQQEYNLTRHNIHAILMDMFAAGTDTSYIALEFAMSELIRKPHLMTKLQDEVRKNTTTQMVSEDDLNNMPYLKAVVKETLRLHPPVPLLLPRLSMAQCNANGYTIPANTRVIINVWALGRDAKCWENSEEFMPERFMDSGDTIDNVDFKGTDFQFLPFGAGRRICPGMNFGMASVELMLSNLMYCFDWELPVGMDKDDVDMTDQFALTMARKEKLYLIPRSHVIKIT | |
| CYP71C19 | MEQAAGLVYQLFQHEMFPWTFSVLALFPFLLLVLHYLATNHRTPTTCKETKNHHPPPPSPPRLPIIGHLHLIGGLLHVSLRELAHRYGPDLMLLHLGQVPNLIVSSPRAAEAVLRTHDLVFASRPYSLIADILLYGPSDVGLSPYGEWRRRIITTHLLTNKKVRSYRVAREEEVHKVMAKVHELSTKGMAVDMTELFSTFSNDLICRLVSGKNFQGEGRNKLFRQLFKANSVLLAGFNLKDYYPGLARLKAVSMVMCAKARNTRKLWDELLDEIIDERMSKQQCEHDEGNDQDEMNFVNVLLLQEQGITREHLKAILVDMYQAGTETSSVVLVFAMAELMQKPHLMAKLQAELRTTIPKQGHELITERDLTDMTYLKAVIKETLRLHPPTPLLLPHLAMADCNIDGYTVRSGTRVIVNAWAIGRNSESWEAAEEFLPERFVDDGSAANVDFIGTDFQFLPFGAGRRICPGINFASASMEIILANLLYHFDWDVSAEAAIDKDGIDMAEAFGLSVQLKEKLLLVPVDYKDGMQDSAVILL | |
| CYP71C2 | MALGAAYHHYLQLAGDHGTATHALLLGVLIFLVIRLVSARRTGTTSANKRKQQQRLPLPPWPPGKLPIIGHLHLIGAETHISIRDLDAKHGRNGLLLLRIGAVPTLFVSSPSAADAVLRTQDHIFASRPPWMAAEIIRYGPSDVAFVPYGEYGRQGRKLLTTHMLSTKKVQSFRHGRQEEVRLVMDKIRAAATAAPPAAVDLSDLLSGYTNDVVSRAVLGASHRNQGRNRLFSELTEINVSLLAGFNLEDYFPPNMAMADVLLRLVSVKARRLNQRWNDVFDELIQEHVQSRPSGESEESEADFIHVLLSIQQEYGLTTDNLKAILVDMFEAGIETSYLTLEYGMAELINNRHVMEKLQTEVRTTMGSPDGKKLDMLAEEDLGSMPYLKATIKETLRLHPPAPFLLPHYSTADSEIDGYFVPAGTRVLVHAWALGRDRTTWEKPEEFMPERFVQEPGAVDVHMKGKDLRFIPFGSGRRICPGMNFGFATMEVMLANLMYHFDWEVPGSGAGVSMEESFGLTLRRKEKLLLVPRIAS | |
| CYP71C20 | MAQMLAAFLLDGLISHEHGHESLGAPPQAGTMAWYSLVLMTSLLFPLLVLLVMRCYVTRSGAKLLDKLPSVPGRLPVIGHLHLIGSLPHISLRDLATKHSPDMMLLHLGAVPTLVVSSSRVAQSILHTHDDIFASRPYSPIANILFYGATDVGFSPYNEYWRQIKKITTTHLLTMKKVRSYVSARQREVRIVMARITEAASKHVVVDLTEMLSCYSNNIVCHAVCGKFSLKEGWNQLLRELVKVNTSLLGGFNIEDYFPSFTRLAAVRRLLLSCAKAHNINKRWDQLLEKLIDDHTTKHIRSSSMLNHYDEEAGFIDVLLSIQHEYGLTKDNIKANLAAMLMAGMDTSFIELEYAMAELMQKPHVMGKLQAEVRRVMPKGQDIVTEEQLGCMPYLKAVIKETLRLHPPAPLLMPHLSISDCNINGYTIPSGTRVIVNVWALARDSNYWENADEFIPERFIVNTLGDYNGNNFHFLPFGSGRRICPGINFAIATIEIMLANLVYRFDWELPADQAAKGGIDMTETFGVAVHRKEKLLLIPHLHLR | |
| CYP71C6 | MALEAAFHYLQLAGIHGTSTPAVLLTILLLLIIRLAWVRTTTASTRFGKQQQLPPSPPGKLPIIGHLHLLGSQTHISIRDLDAKHGRNGLLLLRIGAVPTLFVSSPSAAEAVLRTHDQIFASRPPSMAADIIRYGPTDIAFAPYGEYWRQARKLLTTHMLSAKVVHSFRHGRQEEVRLVINKIHEAATRGTAVDMSELLSGYTNDVVCRAVLGESHRKEGRNRLFSELTEINVSLLGGFSLENYIPPNMVMADVLLRLVSVKARRLNKRWDELFNEIIEEHLHPSKPSSGDQQAVDFIDLLLSLKEEYGLTTDNIKAILVDMFEAGIETSYLTLEYGMAELMNNRHILKKLQEEVRSQGKKLDMITEEDLGSMAYLRATIKETLRLHPPAPFLLPHFSTADCKIDGYLIPSNTRVLVNAWALGRDPSSWERPEDFLPERFLQDQDGDVDTQMRGKDLRFLPFGFGRRICPGMNFGFATMEVMLANLMYHFDWDVPNMVGTGAGVDMAESFGLTLRRKEKLQLVPRIP | |
| CYP71C6 | MALEAAYHYLQRAVGHGTSTEALLLTVLLLLIIRVAWVRAFTTTTASTKCKQQLPPTPPGKLPIIGHLHLIGSHPHVSFRDLAAKHGRDGLMLVHVGAVPTVVVSTPQAAEAVLRTHDHVFASRPRNPVADIIRYNSTDIAFAPYGDYWRRARKVVNTHLLSVKMVYSKRHDREEEVRLVVAKICELAMAAPGKALDMTELLGGYASDFVCRAVLGESHRKHGRNELFRELTEISASLLGGFNLEDYFPRLANLDVFLRVVCSKAMGVSKRWDNLFNELIAEYEHGKEDNAEDFVHLLLSLKKEYGLSTDNVKAILVNMFEAAIETSFLVLEYSMAELINNRHVMAKVQKEVRESTPKGEKLDLIMEEDLSRMPYLKATIKEAMRIHPPAPFLLPHFSTNDCEVNGYTIPAGTRVIVNAWALARDPSHWERAEEFYPERFLQEGRDAEVDMYGKDIRFVPFGAGRRICAGATFAIATVEVMLANLIYHFDWELPSEMEAIGAKVDMTDQFGMTLRRTERLHLVPKIYK | |
| CYP71CA1 | MDTSTSFPSLFLPTLCTILISYIIIKYVLIWNRSSMAAFNLPPSPPKLPIIGNIHHVFSKNVNQTLWKLSKKYGPVMLIDTGAKSFLVVSSSQMAMEVLKTHQEILSTRPSNEGTKRLSYNFSDITFSPHGDHWRDMRKVFVNEFLGPKRAGWFNQVLRMEIKDVINNLSSNPLNTSINLNEMLLSLVYRVVCKFAFGKSYREEPFNGVTLKEMLDESMVVLAGSSADMFPTFGWILDKLYGWNDRLEKCFGNLDGFFEMIINEHLQSASETSEDEKDFVHSLVELSLKDPQFTKDYIKALLLNVLLGAIDTTFTTIVWAMSEIVKNTQVMQKLQTEIRSCIGRKEEVDATDLTNMAYLKMVIKETLRLHPPAPLLFPRECPSHCKIGGYDVFPGTCVVMNGWGIARDPNVWKEIPNEFYPERFENFNIDFLGNHCEMIPFGAGRRSCPGMKSATSTIEFTLVNLLYWFDWEVPSGMNNQDLDMEEDGFLVIQKKSPLFLIPIKHI | |
| CYP71CB1 | MFSSFETLILSFVSLFFMMIFIHSKWISSYSKMAKNLPPSPFGLPIIGNLHQLGMTPYNSLRTLAHKYGSLMLIHLGSVPVIVASSAEAAQEIMKTHDQIFSTRPKMNIASIVSFDAKIVAFSPYGEHWRQSKSVYLLNLLSTKRVQSFRHVREDETNLMLDVIENSCGSEIDLSNMIMSLTNDVVCRIAYGRKYYEDWFKELMKEVMDVLGVFSVGNYVPSLSWIDRLSGLEGRAYKAAKQLDAFLEGVVKQHETKSNESMRDQDVVDILLETQREQASAGTPFHRDTLKALMQEMFIAGTDTTSTAIEWEISEVIKHPRVMKKLQQELDEIAQGRQRITEEDLEDTQHPYLEAILKESMRLHIPVPLLLPREATHDVKVMGYDIAAGTQVLINAWMIARDPTIWEDADEFKPERFLDTNIDYKGLNFELLPFGAGRRGCPGIQFAMSVNKLALANLVYKFDFKLPNGLRLEQLDMTDSTGITVRRKYPLLVIPTARF | |
| CYP71D12 | LLFCFILSKTTKKFGQNSQYSNHDELPPGPPQIPILGNAHQLSGGHTHHILRDLAKKYGPLMHLKIGEVSTIVASSPQIAEEIFRTHDILFADRPSNLESFKIVSYDFSDMVVSPYGNYWRQLRKISMMELLSQKSVQSFRSIREEEVLNFIKSIGSKEGTRINLSKEISLLIYGITTRAAFGEKNKNTEEFIRLLDQLTKAVAEPNIADMFPSLKFLQLISTSKYKIEKIHKQFDVIVETILKGHKEKINKPLSQENGEKKEDLVDVLLNIQRRNDFEAPLGDKNIKAIIFNIFSAGTETSSTTVDWAMCEMIKNPTVMKKAQEEVRKVFNEEGNVDETKLHQLKYLQAVIKETLRLHPPVPLLLPRECREQCKIKGYTIPSKSRVIVNAWAIGRDPNYWIEPEKFNPDRFLESKVDFKGNSFEYLPFGGGRRICPGITFALANIELPLAQLLFHFDWQSNTEKLNMKESRGVTVRREDDLYLTPVNFSSSSPA | |
| CYP71D13 | MELQISSAIIILVVTYTISLLIIKQWRKPKPQENLPPGPPKLPLIGHLHLLWGKLPQHALASVAKQYGPVAHVQLGEVFSVVLSSREATKEAMKLVDPACADRFESIGTKIMWYDNDDIIFSPYSVHWRQMRKICVSELLSARNVRSFGFIRQDEVSRLLGHLRSSAAAGEAVDLTERIATLTCSIICRAAFGSVIRDHEELVELVKDALSMASGFELADMFPSSKLLNLLCWNKSKLWRMRRRVDAILEAIVEEHKLKKSGEFGGEDIIDVLFRMQKDSQIKVPITTNAIKAFIFDTFSAGTETSSTTTLWVMAELMRNPEVMAKAQAEVRAALKGKTDWDVDDVQELKYMKSVVKETMRMHPPIPLIPRSCREECEVNGYTIPNKARIMINVWSMGRNPLYWEKPETFWPERFDQVSRDFMGNDFEFIPFGAGRRICPGLNFGLANVEVPLAQLLYHFDWKLAEGMNPSDMDMSEAEGLTGIRKNNLLLVPTPYDPSS | |
| CYP71D15 | MELLQLWSALIILVVTYTISLLINQWRKPKPQGKFPPGPPKLPLIGHLHLLWGKLPQHALASVAKEYGPVAHVQLGEVFSVVLSSREATKEAMKLVDPACANRFESIGTRIMWYDNEDIIFSPYSEHWRQMRKICVSELLSSRNVRSFGFIRQDEVSRLLRHLRSSAGAAVDMTERIETLTCSIICRAAFGSVIRDNAELVGLVKDALSMASGFELADMFPSSKLLNLLCWNKSKLWRMRRRVDTILEAIVDEHKFKKSGEFGGEDIIDVLFRMQKDTQIKVPITTNSIKAFIFDTFSAGTETSSTTTLWVLAELMRNPAVMAKAQAEVRAALKEKTNWDVDDVQELKYMKSVVKETMRMHPPIPLIPRSCREECVVNGYTIPNKARIMINVWSMGRNPLYWEKPDTFWPERFDQVSKDFMGNDFEFVPFGAGRRICPGLNFGLANVEVPLAQLLYHFDWKLAEGMKPSDMDMSEAEGLTGILKNNLLLVPTPYDPSS | |
| CYP71D16 | MQFFNFFSLFLFVSFLFLFKKWKNSNSQTKRLPPGPWKLPILGSMLHMLGGLPHHVLRDLAKKYGPIMHLQLGEVSLVVISSPGMAKEVLKTHDLAFANRPLLVAAKIFSYNCMDIALSPYGNYWRQMRKICLLELLSAKNVKSFNSIRQDEVHRMIKFFRSSPGKPVNVTKRISLFTNSMTCRSAFGQEYKEQDEFVQLVKKVSNLIEGFDVADIFPSLKFLHVLTGMKAKVMNTHNELDAILENIINEHKKTSKSDGESGGEGIIGVLLRLMKEGGLQFPITNDNIKAIISDIFGGGTETSSTTINWAMVEMMKNPSVFSKAQAEVREILRGKETFGEIDVEEFKYLKMVIKETFRLHPPLPLLLPRECREEIDLNGYTIPLKTKVVVNAWAMGRDPKYWDDVESFKPERFEHNSMDYIGNNYEYLPFGSGRRICPGISFGLANVYFPLAQLLNHFDWKLPTGINPRNCDLTEAAGAACARKNDLHLIATAYQHCEE | |
| CYP71D16 | MQFFNFFSLFLFVSFLFLFKKWKNSNSQTKRLPPGPWKLPILGSMLHMLGGLPHHVLRDLAKKYGPIMHLQLGEVSLVVISSPGMAKEVLKTHDLAFANRPLLVAAKIFSYNCMDIALSPYGNYWRQMRKICLLELLSAKNVKSFNSIRQDEVHRMIKFFRSSPGKPVNVTKRISLFTNSMTCRSAFGQEYKEQDEFVQLVKKVSNLIEGFDVADIFPSLKFLHVLTGMKAKVMNTHNELDAILENIINEHKKTSKSDGESGGEGIIGVLLRLMKEGGLQFPITNDNIKAIISDIFGGGTETSSTTINWAMVEMMKNPSVFSKAQAEVREILRGKETFGEIDVEEFKYLKMVIKETFRLHPPLPLLLPRECREEIDLNGYTIPLKTKVVVNAWAMGRDPKYWDDVESFKPERFEHNSMDYIGNNYEYLPFGSGRRICPGISFGLANVYFPLAQLLNHFDWKLPTGINPRNCDLTEAAGAACARKNDLHLIATAYQHCEE | |
| CYP71D18 | MELDLLSAIIILVATYIVSLLINQWRKSKSQQNLPPSPPKLPVIGHLHFLWGGLPQHVFRSIAQKYGPVAHVQLGEVYSVVLSSAEAAKQAMKVLDPNFADRFDGIGSRTMWYDKDDIIFSPYNDHWRQMRRICVTELLSPKNVRSFGYIRQEEIERLIRLLGSSGGAPVDVTEEVSKMSCVVVCRAAFGSVLKDQGSLAELVKESLALASGFELADLYPSSWLLNLLSLNKYRLQRMRRRLDHILDGFLEEHREKKSGEFGGEDIVDVLFRMQKGSDIKIPITSNCIKGFIFDTFSAGAETSSTTISWALSELMRNPAKMAKVQAEVREALKGKTVVDLSEVQELKYLRSVLKETLRLHPPFPLIPRQSREECEVNGYTIPAKTRIFINVWAIGRDPQYWEDPDTFRPERFDEVSRDFMGNDFEFIPFGAGRRICPGLHFGLANVEIPLAQLLYHFDWKLPQGMTDADLDMTETPGLSGPKKKNVCLVPTLYKSP | |
| CYP71D20 | MQFFSLVSIFLFLSFLFLLRKWKNSNSQSKKLPPGPWKIPILGSMLHMIGGEPHHVLRDLAKKYGPLMHLQLGEISAVVVTSRDMAKEVLKTHDVVFASRPKIVAMDIICYNQSDIAFSPYGDHWRQMRKICVMELLNAKNVRSFSSIRRDEVVRLIDSIRSDSSSGELVNFTQRIIWFASSMTCRSAFGQVLKGQDIFAKKIREVIGLAEGFDVVDIFPTYKFLHVLSGMKRKLLNAHLKVDAIVEDVINEHKKNLAAGKSNGALGGEDLIDVLLRLMNDTSLQFPITNDNIKAVIVDMFAAGTETSSTTTVWAMAEMMKNPSVFTKAQAEVREAFRDKVSFDENDVEELKYLKLVIKETLRLHPPSPLLVPRECREDTDINGYTIPAKTKVMVNVWALGRDPKYWDDAESFKPERFEQCSVDFFGNNFEFLPFGGGRRICPGMSFGLANLYLPLAQLLYHFDWKLPTGIMPRDLDLTELSGITIARKGGLYLNATPYQPSRE | |
| CYP71D443 | MEFTYYFSLFLLFLLSCFVFVLIFSKTKQNLPPGPRKLPIIGHLHHLAGTAPPHHTLRHLADKHGPLMHLQLGECGYVIASSTEIATHFFKTHDALFASRPSILASEIGAYNNTDISFAPYGKFWRQLRRICSVELLSAKRVKSFQPVREEEATELCKWIAQREGSAINLGEKVQQMNYHIMGRAVLGKKTGEQAAFIALVKEGLDLMSGLDIVDLYPSYRILRLFSRLKRRIEKHHHAMDRIAHNIIEDRKRSDNGEHRDHDLLDVLLGLQDDKSLEIPLTTDNIKAVLGDLFGAGVETSSTTVEWAMAEMLKHPKVLKKAQDEVRMVFDAKNGVVDECYFDELKYLKLIVKESLRLHPPGPLLLPRVSSERCEINGYEIPAKTRLLVNVYAIARDPKCWEDGESFKPERFLEKSVDFMGSSIELIPFGAGRRICPGITFGVATVEIALAMLLYYFDWVLPEGMKAEDLDMTDWPGIAARKKDNLWAVPLVRRTLPA | |
| CYP71D445 | MELEFRSPSSPSEWAITSTITLLFLILLRKILKPKTPTPNLPPGPKKLPLIGNIHQLIGGIPHQKMRDLSQIHGPIMHLKLGELENVIISSKEAAEKILKTHDVLFAQRPQMIVAKSVTYDFHDITFSPYGDYWRQLRKITMIELLAAKRVLSFRAIREEETTKLVELIRGFQSGESINFTRMIDSTTYGITSRAACGKIWEGENLFISSLEKIMFEVGSGISFADAYPSVKLLKVFSGIRIRVDRLQKNIDKIFESIIEEHREERKGRKKGEDDLDLVDVLLNLQESGTLEIPLSDVTIKAVIMDMFVAGVDTSAATTEWLMSELIKNPEVMKKAQAEIREKFKGKASIDEADLQDLHYLKLVIKETFRLHPSVPLLVPRECRESCVIEGYDIPVKTKIMVNAWAMGRDTKYWGEDAEKFKPERFIDSPIDFKGHNFEYLPFGSGRRSCPGMAFGVANVEIAVAKLLYHFDWRLGDGMVPENLDMTEKIGGTTRRLSELYIIPTPYVPQNSA | |
| CYP71D55 | MQFFSLVSIFLFLSFLFLLRKWKNSNSQSKKLPPGPWKLPLLGSMLHMVGGLPHHVLRDLAKKYGPLMHLQLGEVSAVVVTSPDMAKEVLKTHDIAFASRPKLLAPEIVCYNRSDIAFCPYGDYWRQMRKICVLEVLSAKNVRSFSSIRRDEVLRLVNFVRSSTSEPVNFTERLFLFTSSMTCRSAFGKVFKEQETFIQLIKEVIGLAGGFDVADIFPSLKFLHVLTGMEGKIMKAHHKVDAIVEDVINEHKKNLAMGKTNGALGGEDLIDVLLRLMNDGGLQFPITNDNIKAIIFDMFAAGTETSSSTLVWAMVQMMRNPTILAKAQAEVREAFKGKETFDENDVEELKYLKLVIKETLRLHPPVPLLVPRECREETEINGYTIPVKTKVMVNVWALGRDPKYWDDADNFKPERFEQCSVDFIGNNFEYLPFGGGRRICPGISFGLANVYLPLAQLLYHFDWKLPTGMEPKDLDLTELVGVTAARKSDLMLVATPYQPSRE | |
| CYP71D9 | MDLQLLYFTSIFSIFIFMFMTHKIVTKKSNSTPSLPPGPWKLPIIGNMHNLVGSPLPHHRLRDLSAKYGSLMHLKLGEVSTIVVSSPEYAKEVMKTHDHIFASRPYVLAAEIMDYDFKGVAFTPYGDYWRQLRKIFALELLSSKRVQSFQPIREEVLTSFIKRMATIEGSQVNVTKEVISTVFTITARTALGSKSRHHQKLISVVTEAAKISGGFDLGDLYPSVKFLQHMSGLKPKLEKLHQQADQIMQNIINEHREAKSSATGDQGEEEVLLDVLLKKEFGLSDESIKAVIWDIFGGGSDTSSATITWAMAEMIKNPRTMEKVQTEVRRVFDKEGRPNGSGTENLKYLKSVVSETLRLHPPAPLLLPRECGQACEINGYHIPAKSRVIVNAWAIGRDPRLWTEAERFYPERFIERSIEYKSNSFEFIPFGAGRRMCPGLTFGLSNVEYVLAMLMYHFDWKLPKGTKNEDLGMTEIFGITVARKDDLYLIPKTVHN | |
| CYP71D97 | MMKMALQFVPIFMFMIILFMLLNLLKKLFQRSTKKLPPGPFKFPIVGNLLQVTGGLPHRRLYNLSKTHGPLMHLQLGEVSAVVISNPRVAKEVLKTHDLCFADRPTLLLGNIVLSNCRDIVLAKYGEHWRQFRKICTLELLSASKVRSFRTIREEEASDLIQSIQSTSGSPVNVSEKVSHLANSITCRSTIGKRCKYEHELIEATENIAYWGAGFFMADLFPSMLVFPVLSGMKPALKKIRRDLDHIFDYIINEHKEKLASRKDQGTKLDAEEEDLVDILLRINDTLQLEFPVTSNDIQGIVQDMFTAGTDTSSAVLEWAMSELMKKPSAMKKAQDELRNALRGKERICEADIQGLTYLKLVIKETLRLHPPVPLLLPRECRKECEIDGYTIPVGTKVMVNAWAIGRDPDYWVDADSFIPERFDGSSVNYNGANFEYIPFGAGRRMCAGITFGIASIELPLAQLLYHFDWTLPNGMKPEDLDMDETFGATTKRKNSLVLNVTSHISSLEE | |
| CYP71E1 | MATTATPQLLGGSVPQQWQTCLLVLLPVLLVSYYLLTSRSRNRSRSGKLGGAPRLPPGPAQLPILGNLHLLGPLPHKNLRELARRYGPVMQLRLGTVPTVVVSSAEAAREVLKVHDVDCCSRPASPGPKRLSYDLKNVGFAPYGEYWREMRKLFALELLSMRRVKAACYAREQEMDRLVADLDRAAASKASIVLNDHVFALTDGIIGTVAFGNIYASKQFAHKERFQHVLDDAMDMMASFSAEDFFPNAAGRLADRLSGFLARRERIFNELDVFFEKVIDQHMDPARPVPDNGGDLVDVLINLCKEHDGTLRFTRDHVKAIVLDTFIGAIDTSSVTILWAMSELMRKPQVLRKAQAEVRAAVGDDKPRVNSEDAAKIPYLKMVVKETLRLHPPATLLVPRETMRDTTICGYDVPANTRVFVNAWAIGRDPASWPAPDEFNPDRFVGSDVDYYGSHFELIPFGAGRRICPGLTMGETNVTFTLANLLYCYDWALPGAMKPEDVSMEETGALTFHRKTPLVVVPTKYKNRRAA | |
| CYP71E1 | MATTATPQLLGGSVPQQWQTCLLVLLPVLLVSYYLLTSRSRNRSRSGKLGGAPRLPPGPAQLPILGNLHLLGPLPHKNLRELARRYGPVMQLRLGTVPTVVVSSAEAAREVLKVHDVDCCSRPASPGPKRLSYDLKNVGFAPYGEYWREMRKLFALELLSMRRVKAACYAREQEMDRLVADLDRAAASKASIVLNDHVFALTDGIIGTVAFGNIYASKQFAHKERFQHVLDDAMDMMASFSAEDFFPNAAGRLADRLSGFLARRERIFNELDVFFEKVIDQHMDPARPVPDNGGDLVDVLINLCKEHDGTLRFTRDHVKAIVLDTFIGAIDTSSVTILWAMSELMRKPQVLRKAQAEVRAAVGDDKPRVNSEDAAKIPYLKMVVKETLRLHPPATLLVPRETMRDTTICGYDVPANTRVFVNAWAIGRDPASWPAPDEFNPDRFVGSDVDYYGSHFELIPFGAGRRICPGLTMGETNVTFTLANLLYCYDWALPGAMKPEDVSMEETGALTFHRKTPLVVVPTKYKNRRAA | |
| CYP71F1 | MEEWLLSLCFIALSTATVLAFWFLKLSGGKADPHKKQLPPGPWTLPVIGSLHHVISALPHRTMMQLSCRHGPLMLLRLGEVPAVVVSTADAAALVMKTHDLVFVDRPRSPTMDIASSGGKDIVFAPYGGHWRQMRKICVVQLLSSTQVSRMEGVRAEEVGSLLRDITAAASTGATINVSEKVMALTNDIVTRAVFGGKFARQCEFLREMDKAFKLVGGFCLADLFPSSRLVRWLSNGERDMKRCHGLIHHIIAEVVENRKAARASGVGRSIPGDEDMLDVLLTLQEDDSLEFPLTTETMGAVLHDVFAGATETTGNTLAWVISELMHNPHTMAKAQHEVRDVLGEGRSVITNSDLGELHYMPMILKEALRLHPPGPLIPRMAREDCTVMGYDIPKGTNVYINIFAISRDPRYWINPEEFMPERFENNNVNYKGTYFEFIPFGAGRRQCPGIQFSSSITEMALANLLYHFDWMLPDGANLASFDMSEKFGFAVSKKYDLKLRAIPHVWSNAMTLK | |
| CYP71G1v1 | MTVSITAAVQLFLLLLLLLPLLFVHHKTKPKTKCRSPPGPPPLPVIGNLHQLSLLLHQSLYRLSKIHGPIFKLSLGRVPVLVISSPSLAKQVLKTHDLACCSRASTVSFKEYTYDGCDVAGAPYGDSWRNLRKIFVLKLLSSKKLTSFRLVQEEEIEGMISSIRTRSDTNATVNITEFVVRLANNITFRVAFGYRSEGEYGEKSRFQRLLESGNDTVASFYVGDYFPGLGWLDKMTGKLGKMKRNARDLDEFYQEVIDAHMKDGRKEDGKEDIVDVLLRLREEGQLTMDHIKGALMNIFVGGTDTSAASIAWAMAELARKPKVMKKAQEEVRKAASKKGKVEENDLAQLQYIKCVVNETLRLHLPLPLLVPRETIQHCEINGYDVSAKTRVLVNAWAIGRDEDAWENPEEFNPDRFVGSSLDYKGQDFQFIPFGAGRRICPGIQFGVETVELALANLLYAFNWELPPGVERENIDMHEAPGLVTRRATDLRLVATNYEEAN | |
| CYP71J1 | MPLILVILLLLPILLLVIRREKSTSSKLPPCPPKLPLIGNLHQLGSLPHQSLHALSVKYGPLMLLKLGEIPTLIVSSSDMAREIMRTHDHIFASRPSLLTSDILLNGATDVVFAPYGEHWRQMRKLCVNHLLSAKMVQSFRLMREEEVSSMLTRISGLVNMSEVLNLFTSKILFNAISGKFFVEEEGRINVFCKLIRENIAILAQLSVSDFFPSLGWLDLVFGVGARARATAKKWDDVLDEVIEDHVKRSNETGDADDQEERADFVSVLMALQEDDNTGFTLNRNIIKAILQDMIAAGTETSFLVLDWGMTELVRNPGTMKKLKDEVRSVAGSETVVREEDISKMFYLKAVIKEILRLHPPVPLLIPRESMDHCNVQQYEVPSKTRVLINAWSMGRDPKVWEDPEEFRPERFLDSDIDFRGQCFEFVPFGAGRRICPGMHFAAANLELALANLMYRFDWELPDGMKSEDLDMGDSPGLTTRRRQNLHLVARPFQRVKR | |
| CYP71K1 | MAELPLYLLLLALLVAVPFLCLTRWSLRHGGGGGGRLPPSPWALPVIGHLHHVAGALPHRAMRDLARRHGPLMLLRLCELRVVVACTAEAAREVTKTHDLAFATRPITPTGKVLMADSVGVVFAPYGDGWRTLRRICTLELLSARRVRSFRAVREEEVGRLLRAVAAAAAVAALTTPGATAAVNLSERISAYVADSAVRAVIGSRFKNRAAFLRMLERRMKLLPAQCLPDLFPSSRAAMLVSRMPRRMKRERQEMMDFIDDIFQEHHESRAAAGAEEDLLDVLLRIQSQDKTNPALTNDNIKTVIIDMFVASSETAATSLQWTMSELMRNPRVMRKAQDEVRRALAIAGQDGVTEESLRDLPYLHLVIKESLRLHPPVTMLLPRECRETCRVMGFDVPEGVMVLVNAWAIGRDPAHWDSPEEFAPERFEGVGAADFKGTDFEYIPFGAGRRMCPGMAFGLANMELALAALLYHFDWELPGGMLPGELDMTEALGLTTRRCSDLLLVPALRVPLRDHER | |
| CYP71L1 | MAAASLVLELLRQQWQVTVAILLLPLASFLLTRRRSSNLNGCDESGGLRLPPCPWRLPMVGNLHQIGSLPHRDLARLARRHGPVMMVRLGMVPAVVLSTAAAAEEAFKTNDKDCSSRPLTVGPGKLTYGYKDVVFAPWSDYVREMRKLFIIEMLSARRVKAAYFARETQIERMVAKLEAVGPNPIRIDEHIFTTVDAIVSLFVFGELNAGEQFKGELVDLLNETTDLLTSFTAEDYFPNAAGRLIDRITGMHGRRETLFRKLDSMMEYLLAMYEDPGHKRKADADGSDLVQEVVDLMKRPPAKGMITFTRDHAKSILFDTFMAATDTSSISSYWVMTELIRHPRVLHKAQAEVRAAAGGAPQVRISDMPKLKYLRMVLSETFRMHPPATMLVPRETMRPIRLGGYDIPANTMLMVNAWAIGRDPASWKDPEVFYPERFEELDVDFNGGHYELLPFGAGRRICPGLAMGVANTEFILANLLYCFNWALPQGMRSEDVGVEEFGGLTFRKKKPLVLVPTRYYPDKEEK | |
| CYP71L1 | MAAASLVLELLRQQWQVTVAILLLPLASFLLTRRRSSNLNGCNESGGLRLPPCPWRLPMVGNLHQIGSLPHRDLARLARRHGPVMMVRLGMVPAVVLSTAAAAEEAFKTNDKDCSSRPLTVGPGKLTYGYKDVVFAPWSDYVREMRKLFIIEMLSARRVKAAYFARETQIERMVAKLEAVGPNPIRIDEHIFTTVDAIVSLFVFGELNAGEQFKGELVDLLNETTDLLTSFTAEDYFPNAAGRLIDRITGMHGRRETLFRKLDSMMEYLLAMYEDPGHKRKADADGSDLVQEVVDLMKRPPAKGMITFTRDHAKSILFDTFMAATDTSSLSSYWVMTELIRHPRVLHKAQAEVRAAAGGAPQVRISDMPKLKYLRMVLSETFRMHPPATMLVPRETMRPIRLGGYDIPANTMLMVNAWAIGRDPASWKDPEVFYPERFEELDVDFNGGHYELLPFGAGRRICPGLAMGVANTEFILANLLYCFNWALPQGMRSEDVGVEEFGGLTFRKKKPLVLVPTRYYPDKEEK | |
| CYP71L7 | LIKQWKTTENRGKLLPSPPKLPVIGHLHLMVGRLPQHVLTRAAQKYGPVMHLQLGEIFSVVVSPREATKQVMKGLDPACADRADSIGTKIMWYDNKDLIFSPYNAHWRQMRKICVSELLNARNEKSFGFIREDEMSRLVRFLRSSAGQAVNMTEKITATTSSIICRAAFGSVVRDDEVLIGLVKTASGMANGFELADLFPSSKLLNLLCLNKYRLWKMRRELDAILEGVVEEHKLKQSGEFGGEDIVDVLLRMQKNSQLQFPITTDTIKGFIFDTFAAGTETSSTTTVWAMAELMKNPRVMANVQAEVREGLKGKKSVDASDVQQLKYLKSVVKETLRLHPPFPLIPRKCREDIEVEGYSIPSNSRIVINVWSLGRDPLYWEEPEIFWPERFDHISTDYVGNNFEFIPFGGGRRICPGLNLGVANVEVPLAQLLYHFDWKLGEPGMSPVHMDMTVAKGLSGPRKTPLFLVPSIYIPTQPN | |
| CYP71M1v1 | MAPDLNPSPELKHVLLWSVPLLIIAPTVIFLYMQAVGKKKKNTIRLPPSPLRLPIIGHLHLMVHEPHRSLQRLARSLGPVVHLQLGGVAAIVVSSPEAAKEVLKTHDVHCCSRPSSPGAKLITYGNQDIAFSPYNASWRERRKLFVSELVSSKRVQSFAYALQAQVGELIQSLSLRSPPREPVNLNETLFTLIDGFIGTVAFGSMKGAKLMKYAKFQQVFSEAMVALSAFSAQDFFPASRMSRWFDKLVGLEARYQRIFLELDSYFEMVLSQHMDPGRVKTDKDDLVDVLISLWKGQGKVTKDHLKALLMDAFIGGTTTSSVTLLWAMSELIKNPTVMKKAQTEIRSLVGDKRRLVQVDDLSKLNYLKMIVKETLRLHPPAPLLVPRETMDHVKVLGYDIPTKTRIFVNVWAMGRDPACWDKPEEFYPERFDGVDTDFYGSHYELLPFGAGRRICPAIPMGATIVEFTLASLLHSFDWELPDGMTKEDVSMEGTGRQIFCRKTPLYLVPSFYTG | |
| CYP71N1 | MALPPLLLSPLPSLLVVLALLSSLLLAGRKARGGSATWKLPPGPPKLPVIGHLHLLGSSLLHRSLWELSKKHGPLMHLKFGRVPVVVVSSPEMAKEVLKTHDLECCSRPSLLSFSKFSYGLSDVAFIPYGERWRQLRKLCTVELLSTRKINSFRDIRKEEMERVTKLICSHVRASSMVNLSELLLSLSCNMTCRSAFGSGFDDGGDIQLHDMLREAQEELSGLFLSDYLPLLGWVDRLSGMRSRLERAFLKLDSIYQRRIDYHQDRLRQQGKEDGDVLDALLRMQKDEEGLTEDHIKGVLMDIFIAGTDTSSATVEWAMAELIRQPELMKRAQDEVRRCVGSKGEVEESDLHQLHFFKCVIKETMRLHPPAPLLLPRETMQHFKLNGYDILPKTWMYVNAWAIGRDPNSWGRPHVFDPERFMHDSTEASGQDFKLIPFGEGRRICPGKNLGMLMVELVLANLLYSFDWHLPPGMVKEDISMEEAPGVTVHREYALCLMATKYDATTA | |
| CYP71P1 | MSLALLVLSAAYVLVALRRSRSSSLKPRRLPPSPPGWPVIGHLHLMSGMPHHALAELARTMRAPLFRMRLGSVPAVVISKPDLARAALTTNDAALASRPHLLSGQFLSFGCSDVTFAPAGPYHRMARRVVVSELLSARRVATYGAVRVKELRRLLAHLTKNTSPAKPVDLSECFLNLANDVLCRVAFGRRFPHGEGDKLGAVLAEAQDLFAGFTIGDFFPELEPVASTVTGLRRRLKKCLADLREACDVIVDEHISGNRQRIPGDRDEDFVDVLLRVQKSPDLEVPLTDDNLKALVLDMFVAGTDTTFATLEWVMTELVRHPRILKKAQEEVRRVVGDSGRVEESHLGELHYMRAIIKETFRLHPAVPLLVPRESVAPCTLGGYDIPARTRVFINTFAMGRDPEIWDNPLEYSPERFESAGGGGEIDLKDPDYKLLPFGGGRRGCPGYTFALATVQVSLASLLYHFEWALPAGVRAEDVNLDETFGLATRKKEPLFVAVRKSDAYEFKGEELSEV | |
| CYP71Q1 | PPLLQLSAAVLFFLLPLLYLLFLRGSNGEVRGRQGNSASAPSLPGPCRQLPVLGNLLQIGSRPHRYFQAVSRRYGPVVQVQLGGVRTVVVHSPEAAEDVLRTNDVHCCSRPPSPGSYNYLDVAFAPYSDYWREMRKLFVVELTSVSRVRSFAYARAAEVARLVDTLAASPPGVPVDLSCALYQLLDGIIGTVAFGKGYGAAQWSTERAVFQDVLSELLLVLGSFSFEDFFPSSALARWADALAGVERRRRRIFRQVDGFLDSVIDKHLEPERLSAGVQEDMVDALVKMWREQQDRPSGVLTREHIKAILMNTFAGGIDTTAITAIWIMSEIMRNPRVMQKARAEVRNTVKNKPLVDEEDSQNLKYLEMIIKENFRLHPPGNLLVPRQTMQPCLIGGYNVPSGTRVFINIWAMGRGPMIWDNPEEFYPERFEDRNMDFRGSNFELVPFGSGRRICPGVAMAVTSLELVVANLLYCFDWKLPKGMKEEDIDMEEIGQISFISFRRKVELFIVPVKHEQYQLMGHIN | |
| CYP71R4L | MASFELDSTLVLLCLVFVVSCFAVVVRGSGTGRKYGVRAVPPGPLALPIIGNLHKLGGAHPHRSLQGLARRHGPLFLLHLGSVPTVVVSSASLAEALLRTQDHVFCSRPQPYTARGTLYGCRDIAFSPYGEKWRQIRRIAVVHLLSMKRVDSFRALRVEEVARFVQRIGAASGRERVDVSELIIDLTNTVISKAAFGNKLGGMEPATVRDMMKELTVLLGTIAVSDVFPRLGWLDWAMGLDARVKRTAARLDTVVERTLAEHEGNRVKDGEACDLLDDLLSIYKDGDQGFKLDRTDVKALILDMFIAGTDTIYKTIEWTMAELVRNPREMAKVQYEVRLHAAASAQGVVLEEELEKMSLLHAAIKEALRLHPPVPLLIPRESIEDTRLHGYDILAKTRVMVNTWAIGRDSESWENAEEFLPERFIGQAMEYNGKDTRFIPFGAGRRGCPGIAFGTRLVELTLANMMYHFDWKLPNGQDIESFELIESSGLSPGLKSALILAVKPL | |
| CYP71S1 | PRPRGLPLIGNLHQVGALPHRSLAALAARHATPLMLLHLGSVPTLVVSTADAARALFRDNDRALSGRPALYAATRLSYGQKNISFAPDGAYWRAARRACMSALLGAPRVRELRDAREREAAALIAAVAAAGASPVNLSDMVAATSSRIVRRVALGDGDGDESMDVKAVLDETQALLGGLWVADYVPWLRWVDTLSGMRRRLELRFHQLDALYERVIDDHLNNRKHASDEEDDLVDVLLRLHGDPAHRSTFGSRSHIKGILDMFIAGSDTSAVTVQWAMTELVRNPDVLAKAQHEVRRVVAAGDKVREADLPELHYLRLVIKETLRLHPAAPLLVPREMTEPFRTAHGVEIPARTRVVVNAMAIHTDPGVWGPDAERFVPERHRDDADGCAQQHDGFALVPFGIGRRRCPGVHFAAAAVELLLANLLFCFDWRAPPGREVDVEEENGLVVHKKNPLVLI | |
| CYP71T1 | MELSSSLAAVLHSPLFLLAALLLLPVFTLLSFSSAKKPGDGGGRRLPLPPSPRGVPFLGHLPLLGSLPHRKLRSMAEAHGPVMLLWFGRVPTVVASSAAAAQEAMRARDAAFASRARVSMAERLIYGRDMVFAPYGEFWRQARRVSVLHLLSPRRIASFRGVREQEVAALLDRVRRRCGVRGGGETVNLSDMLMSYANGVISRAAFGDGAYGLDGDEGGGKLRELFANFEALLGTATVGEFVPWLAWVDKLMGLDAKAARISAELDGLLERVIADHRERRRLSQPDGGDGDGDGDENVDHRDFVDVLLDVSEVEEGAGAGEVLLFDAVAIKAIILDMIAAATDTTFTTLEWAMAELINHPPVMRKLQCEIRAAVGVPGASGGAEVTEDHLGELRLLRAVVKETLRLHAPVPLLVPRETVEDTELLGYRVPARTRVIINVWAIGRPXAAWGDRAEEFVPERWLDGGGGGEAVEYAAQLGQDFRFVPFGAGRRGCPGAGFAAPSIELALTNLLYHFDWELPPHADGAAAATAARLDMGELFGLSMRMKTTLNLVAKPWSSDV | |
| CYP71T3 | MAVSLVVVVVVVIAIVVPLLYLVLLPAWKPARRDDGDGGMRRRLPPSPPWGLPLLGHLHLLGALPHRALRSLAAAHGPVLLLRLGRVPVVVVSSAAAAEEVMRTRDLEFASRPRVAMAERLLYGGRDVAFAPYGEYWRQTRRICVVHLLSARRVLSFRRVREEEAAALVARVRAAGGAVDLVEHLTAYSNTVVSRAVFGDESARGLYGDVDRGRVLRKLFDDFVELLGQEPMGELLPWLGWVDALNGMEVKVQRTFEALDGILEKVIDDHRRRRREVGRQMDDGGGGDHRDFVDVLLDVNETDMDAGVQLGTIEIKAIILDMFAAGTDTTTTVIEWAMAELITHPDAMRNAQDEIKAVVGITSHITEDHLDRLPYLKAVLKETLRLHPPLPLLVPHEPSSDTKILGYSIPACTRIVINAWTIGRDQATWGEHAEEFIPERFLESGLDYIGQDFVLVPFGAGRRGCPGVGFAVQAMEMALASLLYNFDWETRVVDRRSEFGTSSLDMSEMNGLSVRLKYGLPLIAISRFP | |
| CYP71U2 | MDELSIENHSPISMDELSFGSLCLVAMATLALALALMVVMGAHRRGGEKGATTGAKNLPPGPWNLPVTGSLHHLLGASPPPHRALLRLSRRHGPLMLVRLGEVPTVIVSGSDAAMEGWVLKAHDPAFADRARSTTVDAVSFGGKGIIFAPYGEHWRQARRVCLAELLSARQVRRLESIRQEEVSRLVGSIAGSSNAAAVDMTRALAALTNDVIARAVFGGKCARQEEYLRELGVLTALVAGFSMADLFPSSRVVRWLSRRTERRLRRSHAQMARIVGSIIEERKEKKASDDGVGAKDEDDDLLGVLLRLQEEDSLTSPLTAEVIGALVIDIFGAATDTTASTLEWVMVELMRNPRAMEKAQQEVRNTLGHEKGKLIGTDISELHYLRMVIKETLRLHPSSALILRQSQGNCRVMGYDIPQATPVLINTFAVARDAKYWDNAEEFKPERFENSGADIRTSTAHLGFVPFGAGCRQCPGALFATTTLELILANLLYHFDWALPDGVSPESLDMSEVMGITLHRSSSLHLHATLSRLGFVSHSGQ | |
| CYP71U5 | KAKLPPGPWNLPVIGSLHHLVVTKLPPHRALLRLSRRHGPLMLVWLGEVPSIVVSSPEAAKEVLKTNDLVFANRPCGPTMDIVSCGGKGILLAPYGDHWRQMRKVCVVEVLSARQVRRIESIQQAEVARLLESVSAATTGCAVVDVGKALAELSSNIIATAVFGGKFPQQEAFLREIDALSVLVGGFSMADLFPSSRLVRWLSSATHDVKRSHARVQRILEDIIQERKEKTSKNGASSAARDNEDLLDVLLRLQRDDTLSFPLTSEIIDCVIRDIIGAATETTSSTIEWAMAELVGNPEAMAKAKHEVRERCHGVVASADIGELQYLRMVIKETLRLHPAGMFHRASLEDCQVMGYHIPKGTAVMINGFAVGTDPAHWGEDAGEFRPERFHDMEMTEYMQMEFVPFGAGRRQCPGTLFATTIMELVLAHLLCHFDWEVPDGQTLDMGEDYGFIVHTRSSLRLQASSIVPSC | |
| CYP71V1 | MDDYFFLQSLLLCVAAVALLQLAKVAATMRRRPRTPPGPWRLPVIGSMHHLVNALPHRAMRDLAGVHGPLMMLRLGETPVVVASSRGAARAVLKTHDANFATRPRLLAGEIVGYGWADILFSPSGDYWRKLRQLCAAEILSPKRVLSFRHIREDEVTARVEEIRAAAAPSTPVNLSVLFHSTTNDIVARAAFGRKRKSAPEFMAAIKAGVGLSSGFKIPDLFPTWTTALAAVTGMKRSLRGIHKTVDAILQEIIDERRCVRGDKINNGGAADDQNADENLVDVLIALQEKGGFGKSVTTPWVIVTHMICTLDVQDMFAGGTGTSASALEWAMSELMRNPAVMKKLQGQIREAFHRKAVVTEADLQASNLRYLKLVIKEALRLHPPAPLLVPRESIDTCELDGYTIPAKSRVIVNVWAIGRDPKDAEEFKPEQFDDDAIDFMGGSYEFIPFGSGRRMCPGFNYGLASMELVLVAMLYHFDWSLLVGVKEVDMEEAPGLGVRRRSPLLLCATPFVPAAVSADY | |
| CYP71W1 | MELTTLLLLALISFFFLVKLIARYASPSGRESALRLPPGPSQLPLIGSLHHLLLSRYGDLPHRAMRELSLTYGPLMLLRLGAVPTLVVSSAEAAAEVMRAHDAAFAGRHLSATIDILSCGGKDIIFGPYTERWRELRKVCALELFNHRRVLSFRPVREDEVGRLLRSVSAASAEGGAACFNLSERICRMTNDSVVRAAFGARCDHRDEFLHELDKAVRLTGGINLADLYPSSRLVRRLSAATRDMARCQRNIYRIAESIIRDRDGAPPPERDEEDLLSVLLRLQRSGGLKFALTTEIISTVIFDIFSAGSETSSTTLDWTMSELMKNPRILRKAQSEVRETFKGQDKLTEDDVAKLSYLQLVIKETLRLHPPAPLLIPRECRETCQVMGYDVPKGTKVFVNVWKIGREGEYWGDGEIFRPERFENSTLDFRGADFEFIPFGAGRRMCPGIALGLANMELALASLLYHFDWELPDGIKSEELDMTEVFGITVRRKSKLWLHAIPRVPYYSTY | |
| CYP71X2 | MYDAVACVVAVVVVVVFAMLWVKLARSGDGGGGGSGGVRLPPGPWRLPVIGSLHHVVGDRLLHRSMARIARRLGDAPLVYLQLGEVPVVVASSPGAAREVTRTHDLAFADRALNPTARRLRPGGAGVALAPYGALWRQLRKICVVELLSARRVRSFRRVREEEAGRLVGALAAAAASPGEEAAVNFTERIAEAVSDAALRAMIGDRFERRDEFLQELTEQMKLLGGFSLDDLFPSSWLASAIGGRARRAEANSRKLYELMDCAIRQHQQQRAEAAVVDGGAGVEDDKNQDLIDVLLNIQKQGELETPLTMEQIKAVILDLFSGGSETSATTLQWAMSELIKNPMVMQKTQAELRDKLRRKPTVTEDDLSGLKYVKLIIKETLRLHPVVPLLVARECRESCKVMGYDVPKGTTVFVNAWAIGRDPKYWDDAEEFRPERFEHSTVDFKGIDLEFIPFGAGRRICPGMAFAEAIMELLLAALLYHFDWELPNGMAASELDMTEEMGITVRRKNDLHLRPHPPCVVRSNFRSFVERERERHFV | |
| CYP71Y1 | MEDATHGYVYVGLALVSLFVVLLARRRRSPPPAAHGDGGLRLPPGPWTLPIIGSLHHLVGQIPHRAMRDLARRHGPVMLLRIGEVPTLVVSSRDAAREVTKTHDTAFAMRPLSATLRVLTNGGRDLVFAPYGDYWRQVRKIAVTELLTARRVHSFRSIREEEVAALLRAVAVAAGTVEMRAALSALVSDITARTVFDNRCKDRGEFLVLLERTIEFAGGFNPADLWPSSRLAGRLSSVVRRAEECRNSVYKILDGIIQEHQERTSAGGEDLVDVLLRIQKEGGLQFPLAMDDIKSIIFDIFSAGSETSATTLAWAMAELIRNPTAMHKVMAEVRRAFAAAGAVSEDALGELRYLQLVIRETLRLHPPLPLLLPRECREPCRVLGYDVTRGTQVLVNAWAIGLDERYWPGGSPEEFRPERFEDGEATAAVDFRGTDFEFLPFGAGRRMCPGMAFGLANVELPLASLLFHFDWEVPGLADPAKLDMTEAFGITARRKADLHLRPCLLVSVPGV | |
| CYP71Z1 | MGASILLVVVVSKLMISFAAKPRLNLPPGPWTLPLIGSIHHVVSSRESVHSAMRRLARRHGAPLMQLWFGEVGTVVASSPEAAREVLRSHDLAFADRHLTAAAAAFSFGGRDVVLSPYGERWRQLRKLLTQELLTASRVRSFRRVREEEVARLMRDLSAAATAGAAVNLSEMVTRMVNDTVLRCSVGSRCEHSGEYLAALHAVVRLTSGLSVADLFPSSRLAAMVSAAPRAAIANRDKMVRIIEQIIRERKAQIEADDRAADSKSCACSLDDLLRLQKEGGSPIPITNEVIVVLLMDMFAGGTDTSSTTLIWAMAELIRSPRVMAKVQSEMRQIFDGKNTITEDDLVQLSYLKMVIKETLRLHCPLPLLAPRKCRETCKIMGYDVPKGTSAFVNVWAICRDSKYWEDAEEFKPERFENNDIEFKGSNFEFLPFGSGRRVCPGINLGLANMEFALANLLYHFDWKLPNGMLHKDLDMREAPGLLVYKHTSLNVCPVTHIASSCA | |
| CYP71Z18 | MEDKVLIAVGTVAVVAVLSKLKSAVTKPKLNLPPGPWTLPLIGSIHHIVSNPLPYRAMRELAHKHGPLMMLWLGEVPTLVVSSPEAAQAITKTHDVSFADRHINSTVDILTFNGMDMVFGSYGEQWRQLRKLSVLELLSAARVQSFQRIREEEVARFMRSLAASASAGATVDLSKMISSFINDTFVRESIGSRCKYQDEYLAALDTAIRVAAELSVGNIFPSSRVLQSLSTARRKAIASRDEMARILGQIIRETKESMDQGDKTSNESMISVLLRLQKDAGLPIELTDNVVMALMFDLFGAGSDTSSTTLTWCMTELVRYPATMAKAQAEVREAFKGKTTITEDDLSTANLRYLKLVVKEALRLHCPVPLLLPRKCREACQVMGYDIPKGTCVFVNVWAICRDPRYWEDAEEFKPERFENSNLDYKGTYYEYLPFGSGRRMCPGANLGVANLELALASLLYHFDWKLPSGQEPKDVDVWEAAGLVAKKNIGLVLHPVSHIAPVNA | |
| CYP71Z6 | MEDKLILDLCLSALFVVVLSKLVSSAMKPRLNLPPGPWTLPLIGSLHHLVMTKSPQTHRSLRALSEKHGPIMQLWMGEVPAVVVSSPAVAEEVLKHQDLRFADRHLTATTEEVFFGGRDVIFGPYSERWRHLRKICMQELLTAARVRSFQGVREREVARLVRELAADAGAGGDAGVNLNERISKLANDIVMVSSVGGRCSHRDEFLDALEVAKKQITWLSVADLFPSSKLARMVAVAPRKGLASRKRMELVIRRIIQERKDQLMDDSAAGAGEAAAGKDCFLDVLLRLQKEGGTPVPVTDEIIVVLLFDMISGASETSPTVLIWTLAELMRNPRIMAKAQAEVRQAVAGKTTITEDDIVGLSYLKMVIKETLRLHPPAPLLNPRKCRETSQVMGYDIPKGTSVFVNMWAICRDSRYWEDPEEYKPERFENNSVDYKGNNFEFLPFGSGRRICPGINLGVANLELPLASLLYHFDWKLPNGMAPKDLDMHETSGMVAAKLITLNICPITHIAPSSA | |
| CYP71Z7 | MEDNKLILALGLSVLFVLLSKLVSSAMKPRLNLPPGPWTLPLIGSLHHLVMKSPQIHRSLRALSEKHGPIMQLWMGEVPAVIVSSPAVAEEVLKHQDLRFADRHLTATIEEVSFGGRDVTFAPYSERWRHLRKICMQELLTAARVRSFQGVREREVARLVRELAADAGAGGDAGVNLNERISKLANDIVMVSSVGGRCSHRDEFLDALEVAKKQITWLSVADLFPSSKLARMVAVAPRKGLASRKRMELVIRRIIQERKDQLMDDSAAGAGEAAAGKDCFLDVLLRLQKEGGTPVPVTDEIIVVLLFDMFTGASETSPTVLIWILAELMRCPRVMAKAQAEVRQAAVGKTRITENDIVGLSYLKMVIKEALRLHSPAPLLNPRKCRETTQVMGYDIPKGTSVFVNMWAICRDPNYWEDPEEFKPERFENNCVDFKGNNFEFLPFGSGRRICPGINLGLANLELALASLLYHFDWKLPNGMLPKDLDMQETPGIVAAKLTTLNMCPVTQIAPSSAEDAS | |
| CYP720A1 | MAESAGESYRLLSVSSSTTFLAFIIIFLLAGIARRKRRAPHRLPPGSRGWPLIGDTFAWLNAVAGSHPSSFVEKQIKRYGRIFSCSLFGKWAVVSADPDFNRFIMQNEGKLFQSSYPKSFRDLVGKDGVITVHGDQQRRLHSIASSMMRHDQLKTHFLEVIPVVMLQTLSNFKDGEVVLLQDICRKVSIHLMVNQLLGVSSESEVDEMSQLFSDFVDGCLSVPIDLPGFTYNKAMKVSFKHLSQLLIQARKEIIRKINKTIEKRLQNKAASDTAGNGVLGRLLEEESLPNESMADFIINLLFAGNETTSKTMLFAVYFLTHCPKAMTQLQEEHDRLAGGMLTWQDYKTMDFTQCVIDETLRLGGIAIWLMREAKEDVSYQDYVIPKGCFVVPFLSAVHLDESYYKESLSFNPWRWQKRNWRTSPFYCPFGGGTRFCPGAELARLQIALFLHYLIACFRWTQLKEDRISFFPSARLVNGFKIQLNRRDSDPPNQ | |
| CYP720B1 | MADQISLLLVVFTAAVALLHLIYRWWNAQRGQKRTSNEKNQELHLPPGSTGWPLIGETYSYYRSMTSNRPRQFIDDREKRYDSDVFVSHLFGSQAVISSDPQFNKYVLQNEGRFFQAHYPKALKALIGDYGLLSVHGDLQRKLHGIAVNLLRFERLKFDFMEEIQNLVHSTLDRWVDKKEIALQNECHQMVLNLMAKQLLDLSPSKETNEICELFVDYTNAVIAIPIKIPGSTYAKGLKARELLIRKISNMIKERRDHPHIVHKDLLTKLLEEDSISDEIICDFILFLLFAGHETSSRAMTFAIKFLTTCPKALTQMKEEHDAILKAKGGHKKLEWDDYKSMKFTQCVINETLRLGNFGPGVFRETKEDTKVKDCLIPKGWVVFAFLTATHLDEKFHNEALTFNPWRWELDQDVSNNHLFSPFGGGARLCPGSHLARLELALFLHIFITRFRWEALADEHPSYFPLPYLAKGFPMRLYNRE | |
| CYP721A1 | MAVFFILVLVFFFLVFRFIYSNIWVPWRIQSHFKKQSVTGPSYRIFSGNSGEVSRLTAEAKSKPIPSGRNPHEFVHRVAPHYHEWSRVYGKTFLYWFGSKPVVATSDPRLIREALTTGGSFDRIGHNPLSKLLYAQGLPGLRGDQWAFHRRIAKQAFTMEKLKRWVPQMVTSTMMLMEKWEDMRNGGEEIELEVHKEMHNLSAEMLSRTAFGNSVEEGKGIFELQERMMRLFYLVRWSVYIPGFRFFPSKTNREIWRIEKQIRVSILKLIENNKTAVEKSGTLLQAFMSPYTNQNGQEEKLGIEEVTDECKTFYFAAKETTANLMTFVLVLLAMNQEWQNIAREEVICVLGQTGLPTLDILQDLKTLSMIINETLRLYPPAMTLNRDTLKRAKLGDLDIPAGTQLYLSVVAMHHDKETWGDDAEEFNPRRFEDPKKQSALLVPFGLGPRTCVGQNLAVNEAKTVLATILKYYSFRLSPSYAHAPVLFVTLQPQNGAHLLFTRISS | |
| CYP721B4 | MAALTSALLFAALLVAAQYVLRLLHSFLWVPFRLERRFRRQGIRWPPRSLVSGNAADYRDLLAAARSAPLSSFRHNGVVARATPQYAVWLARYGRPFVYWFGPRPRLVISDTELVKAVMTDSTGGFDKAASGGNNPLARQLIGEGLVGLSGETWARHRRVISPAFNMERVKAWIPEIAAAASPVLDKWEAEGGSRTEFEIDVHKAFHTLSADVISCVAFGSSYEEGKRIFQLQEEQMQLALLAMRTVYIPGFRFVPTKKNRKRQRLNQEIQCSLRKLIEINGTKCEDSKNLLGLMLSASKAGSEYKMGIEEIIHECKTFYFAGKETTANLLTWATLLLALHQEWQVKARDEVLKVCGKHEHPNAENLSDLKIVTMVLKETLRLYPPATFINRTATRDIKLGKLDIPAGTRLDFPIIHIHRDHEVWGMDAEEFNPSRFADGSSYHLGAYFPFGIGPTICVGQNLAMVEAKVALAMTLQRFAFTVSASYAHAPMLVFTLQPQFGAQVLVRKI | |
| CYP722A1 | MEHLCLCLVLCAAMLTLGKFLKIMFQDRKKSTAGVPPGSDGFPVIGETLQFMLSVNSGKGFYEFVRSRRRYGSCFRTSLFGETHVFLSTTESARLVLNNDSGMFTKRYIKSIGELVGDRSLLCAPQHHHKILRSRLINLFSKRSTALMVRHFDELVVDALGGWEHRGTVVLLTDLLQITFKAMCKMLVSLEKEEELGSMQRDVGFVCEAMLAFPLNLPWTRFHKVMQARGRVMEMLEKIIRERRNEINSHNNHHEDFLQQLLAVDNDTPQLTDAEIKDNILTMIISGQDTTASALTWMVKYLGENQKVLDILIEEQSQITKKASNKPFLELEDLSEMPYASKMVKESLRMASVVPWFPRLVLQDCEMEGYKIKKGWNINIDARSIHLDPTVYSEPHKFNPLRFEVNKPKANSFLAFGMGGRTCLGLALAKAMMLVFLHRFITTYRWEVVDEDPSIEKWTLFARLKSGYPIRVSRRL | |
| CYP722A1 | MEXDEIKRSMLNLLPNATLQLCCYTVVLIVSLMWLMRGIARVLRDSHWESTAKIPPGSRGLPLIGETLHFMAATSSSKGFYDFVHIRQLRTSIFGQTHVFVSSTESAKVVLNNEVGKFTKRYIKSIAELVGNESLLCASHQHHKLIRGRLINLFSTASISSFIKQFDQLIVTTLSGWEHKPTVVVLHEALELICKAMCKMLMSLESGDEVEMLQKDVAHVCEAMIAFPLRLPCTRFYKGLEARKRVMKMLEKKIEERRRGEAYHEDFLQHLLKDNGSACCDEVPPLTDAEIQDNILTMIIAGQDTTASAITWMVKYLDENQHVLHTLRAEQGRIAEKTSHTSSLTLDDLNEMPYASKVVKESLRMASIVAWLPRVALQDCEVQGFKIKKGWNINIDARSIHLDPTLYNNPTMFIPSRFDGEXKPNSFLAFGTGGRTCLGMNMAKAMMLVFLHRLITTYNWTVVNPDSSIEKWALFSRLKSGCPIHVSPIAKDAADA | |
| CYP722B1 | MESLAAGAWWVVVLLLLVLTIVASWYRSWWKTTEAGGPLLPPPAAGAGPWWVWVWQWRETAAFLASHGSGRGFYHFVQERYKLYKGEGEGEATCCFRTALMGRVHVFVSASHPAASQLLTAEPPHLPKRYARTAADLLGPHSILCSTSHAHHRHARRALATTLFATPSTAAFAAAFDRLVIRHWTTLLPPHNQNQVVVVLDAALHISYRAICEMLLGAGGGKLRPLQSDVFAVTQAMLALPLRWLPGTRFRRGLHARKRIMAALREEMAARNHHHHHHHHHHDLLSVLMQRRQLGHPDALTEDQILDNMLTLIIAGQVTTATAITWMVKYLSDNRLIQDKLRAEAFRLELKGDYSLTMQHLNAMDYAYKAVKESLRMATIVSWFPRVALKDCQVAGFHIKKDWIVNIDARSLHYDPDVFDNPTVFDPSRFDVQPQKRRLLVFGAGGRTCLGMNHAKIMMLIFLHRLLTNFRWEMADDDPSLEKWAMFPRLKNGCPILLTPIHNS | |
| CYP722C1 | MLNLLREEVLLVVQKYYYDLIMVALFTIGVTYLASKAWKRATTNNREEIPGRLGLPFIGETFSFLSATNSTRGCYDFVRLRRLWNGRWFKTRLFGKIHIFIPSPEGARTIFANDFVLFNKGYVKSMADAVGQKSLLCVPVESHKRIRGLLSEPFSMTSLSAFVTKFDKMLCGRLQKLEESGKSFKVLDLCMKMTFDAMCDMLMSITEDSLLRQIEEDCTAVSDAMLSIPIMIPRTRYYKGITARKRVMETFGEIIARRRRGEETPEDFLQSMLQRDSLPASEKLDDSEIMDNLLTLIIAGQTTTAAAMMWSVKFLHDNRETQDILREEQLSITKMKPEGASINHEDLNSMRYGLKVVKETLRMSNVLLWFPRVALEDCTIEGYDIKKGWHVNIDATHIHHDSDLYKDPLKFNPQRFDEMQKPYSFIPFGSGPRTCLGINMAKVTMLVFLHRLTGGYTWTLDDLDTCLEKKAHIPRLRNGCPITLKSLSKSMPEA | |
| CYP723A2 | MMLLLTLLLVLTLFLCLAVFRRTTSRARRAPVSLRQPTVEIHDGDVARRALLDHADAFVNRPAIGAEPRGRRSDNLTTVRYGPQWRVLWRNLTAGFLRPSRVGLLAPLQQKAVDALVADIAARGADGGEVGVRDVVHDALFPLAVRFCFGDDIDERHVRDLRRVMQELKLDVVVKGFSGSMLANLVHWWRLRRFIASGRRRAEIFLPLIAQRRRTQHRDADGGVFRPYVDSLLDLRVPVGHDADADAAGCEDNEGRNSGRALTDDEMVGLVAEFLSGGTETVVSCVEWTLAHLVIEPEIQDKLCRQVVAAADHHGGERGTTPAYLRAVILESLRMHPPVPLTMRDVRSPQAVEHLSLPDGGARVHFILGDIGRDGKAWTDPDEFRPERFMAGGEAEGVGPLPGPREVRMMPFGAGRRYCPGMGLGVAHACLLVAALVREFEWAPTAVAATGGVDLTEVNGFFKMMRTPLRARATPRGTSA | |
| CYP724A1 | MLVLSIFLSLGLFFLSILILYISISKKNETNDHHSSLTGSMGWPFIGETISFFKPHRSDSIGTFLQQRVSRYGKVFKSNICGGKAVVSCDQELNMFILQNEGKLFTSDYPKAMHDILGKYSLLLATGEIHRKLKNVIISFINLTKSKPDFLHCAENLSISILKSWKNCREVEFHKEVKIFTLSVMVNQLLSIKPEDPARLYVLQDFLSYMKGFISLPIPLPGTGYTNAIKVRSNRNIHQNAIIEDMNNAIREEDFLDSIISNEDLNYEEKVSIVLDILLGGFETSATTLSLVVYFLAKSPNLLHKLKEEHAAIRAKKGDGELLNWEDYQKMEFTQCVISEALRCGNIVKTVHRKATHDIKFNEYVIPKGWKVFPIFTAVHLDPSLHENPFEFNPMRWTDKAKMNKKTTAFGGGVRVCPGGELGKLQIAFFLHHLVLSYRWKIKSDEMPIAHPYVEFKRGMLLEIEPTKFLED | |
| CYP724B1 | MVGGELVLAALVILLALLLTLVLSHFLPLLLNPKAPKGSFGWPLLGETLRFLSPHASNTLGSFLEDHCSRYGRVFKSHLFCTPTIVSCDQELNHFILQNEERLFQCSYPRPIHGILGKSSMLVVLGEDHKRLRNLALALVTSTKLKPSYLGDIEKIALHIVGSWHGKSKDKGMVNVIAFCEEARKFAFSVIVKQVLGLSPEEPVTAMILEDFLAFMKGLISFPLYIPGTPYAKAARARISSTVKGIIEERRNAGSSNKGDFLDVLLSSNELSDEEKVSFVLDSLLGGYETTSLLISMVVYFLGQSAQDLELVKREHEGIRSKKEKDEFLSSEDYKKMEYTQHVINEALRCGNIVKFVHRKALKDVRYKEYLIPSGWKVLPVFSAVHLNPLLHGNAQQFQPCRWEGASQGTSKKFTPFGGGPRLCPGSELAKVEAAFFLHHLVLNYRWRIDGDDIPMAYPYVEFQRGLPIEIEPLCSES | |
| CYP725A1 | MDSFIFLRSIGTKFGQLESSPAILSLTLAPILAIILLLLFRYNHRSSVKLPPGKLGFPLIGETIQLLRTLRSETPQKFFDDRLKKFGPVYMTSLIGHPTVVLCGPAGNKLVLSNEDKLVEMEGPKSFMKLIGEDSIVAKRGEDHRILRTALARFLGAQALQNYLGRMSSEIGHHFNEKWKGKDEVKVLPLVRGLIFSIASTLFFDVNDGHQQKQLHHLLETILVGSLSVPLDFPGTRYRKGLQARLKLDEILSSLIKRRRRDLRSGIASDDQDLLSVLLTFRDEKGNSLTDQGILDNFSAMFHASYDTTVAPMALIFKLLYSNPEYHEKVFQEQLEIIGNKKEGEEISWKDLKSMKYTWQAVQESLRMYPPVFGIFRKAITDIHYDGYTIPKGWRVLCSPYTTHLREEYFPEPEEFRPSRFEDEGRHVTPYTYVPFGGGLRTCPGWEFSKIEILLFVHHFVKNFSSYIPVDPNEKVLSDPLPPLPANGFSIKLFPRS | |
| CYP726A1 | MEQKNLSFPSILISFLLVLILVVVMRLWKKQNPPPGPWKFPIIGNLPHLLLTSDLGHERFRALAQIYGPVMSLQIGQVSAVVISSAEAAKEVMKTQADAFAQRPIVLDAQIVFYNRKDVLFASYGDHWRQMKKIWILEFLSAKKVQSSRLIREEEMEDAITFLRSKAGSPVNITKIIYGIIISIMIRTSVGNCKQKERLLSVADAVNEAATSFGTADAFPTWKLLHYIIGAESKPRRLHQEIDDILEEILNEHKANKPFEADNLMDVLLNLQKNGNVPVPVTNESIKASVLQMFTAGSETTSKATEWVMAELMKNPTELRKAQEEVRQVFGEMGKVDESRFHDLKFFKLVVKETLRLHPPVVLIPRECRETTRIDGYEIHPNTRIVVNAWAIGRDPNTWSEPGKFNPERFKDCAIDYKGTTFELVPFGAGKRICPGITSAITNLEYVIINLLYHFNWELADGITPQTLDMTEAIGGALRKKIDLKLIPIPYQVSLGSNIS | |
| CYP726A1 | MEQKNLSFPSILISFLLVLILVVVMRLWKKQNPPPGPWKFPIIGNLPHLLLTSDLGHERFRALAQIYGPVMSLQIGQVSAVVISSAEAAKEVMKTQADAFAQRPIVLDAQIVFYNRKDVLFASYGDHWRQMKKIWILEFLSAKKVQSSRLIREEEMEDAITFLRSKAGSPVNITKIIYGIIISIMIRTSVGNCKQKERLLSVADAVNEAATSFGTADAFPTWKLLHYIIGAESKPRRLHQEIDDILEEILNEHKANKPFEADNLMDVLLNLQKNGNVPVPVTNESIKASVLQMFTAGSETTSKATEWVMAELMKNPTELRKAQEEVRQVFGEMGKVDESRFHDLKFFKLVVKETLRLHPPVVLIPRECRETTRIDGYEIHPNTRIVVNAWAIGRDPNTWSEPGKFNPERFKDCAIDYKGTTFELVPFGAGKRICPGITSAITNLEYVIINLLYHFNWELADGITPQTLDMTEAIGGALRKKIDLKLIPIPYQVSLGSNIS | |
| CYP726A14 | MEQQLLSFPALLSFLLLIFVVLRIWKQYTYKGKSTPPPGPWRLPLLGNFHQLVGALPHHRLTELAKIYGPVMGIQLGQISVVIISSVETAKEVLKTQGEQFADRTLVLAAKMVLYNRNDIVFGLYGDHWRQLRKLCTLELLSAKRVQSFKSVREEELSNFVKFLHSKAGMPVNLTHTLFALTNNIMARTSVGKKCKNQEALLSIIDGIIDASGGFTIADVFPSVPFLHNISNMKSRLEKLHQQADDILEDIINEHRATRNRDDLEEAENLLDVLLDLQENGNLEVPLTNDSIKGAILVGFFFLSSPFFFSTNYHVNSLISLHLIKFVLDMFGAGSDTSSKTAEWALSELMRHPEEMKKAQEEVRRIFGEDGRIDEARFQELKFLNLVIKETLRLHPPVALIPRECREKTKVNGYDIYPKTRTLINVWSMGRDPSVWTEAEKFYPERFLDGTIDYRGTNFELIPFGAGKRICPGMTLGIVNLELFLAHLLYHFDWKLVDGVAPDTLDMSEGFGGALKRKMDLNLVPIPFTTLP | |
| CYP726A17 | MEKQILSFPVLLSFVLFILMILRIWKKSNPPPGPWKLPLLGNIHQLAGGALPHHRLRDLAKTYGPVMSIQLGQISAVVISSVQGAKEVLKTQGEVFAERPLIIAAKIVLYNRKDIVFGSYGDHWRQMRKICTLELLSAKRVQSFRSVREEEVSEFVRFLQSKAGTPVNLTKTLFALTNSIMARTSIGKKCEKQETFSSVIDGVTEVSGGFTVADVFPSLGFLHVITGMKSRLERLHRVADQIFEDIIAEHKATRALSKNDDPKEAANLLDVLLDLQEHGNLQVPLTNDSIKAAILEMFGAGSDTSSKTTEWAMSELMRNPTEMRKAQEEVRRVFGETGKVDETRLHELKFLKLVVKETLRLHPAIALIPRECRERTKVDGYDIKPTARVLVNVWAIGRDPNVWSEPERFHPERFVNSSVDFKGTDFELLPFGAGKRICPGILVGITNLELVLAHLLYHFDWKFVDGVTSDSFDMREGFGGALHRKSDLILIPIPFTP | |
| CYP727A1 | MASPCEHHVPYTLLGALLSGGGPHAAACGGAAFLRDYAERGTNALLWAALLAVTWLLVLRLAALLRLWALGARLPGPPAFPADPGLAAGDITGYLSKLHGSYGPVVRLWLGPSQLLVSVKDSRVIKELLTKAEDKLPLTGKTYNLACGKLGLFISSFEKVKSRRESLKSFLDEKLSVGTGGSSFKIIQIVLDRVDSIMARDFLDCRYFSQHMAFNIVGSALFGDAFFDWSDASAYEELMMTVAKDACFWASYAVPPFWKPDYRRYRTLCARLKLLTQGIVAKSRNQNGVLSLIDLSSCQRSERMIKDPCRGFSLLDGVISSRCLNEAAEGPLSSEEEICGNIMGLMLHGISTCANLIGNILTRLALYPNLQCQLHSEIVSGHSESSELKIDDVLRMKFLLATVCESARLLPAGPLLQRCSLQQDVNLNSSITIPAGAILVIPLHLVQMEASTWGNDACQFNPNRFLKKEINFEEILAAAHKGSNGINLFTDECDKTESFLPFGSGSRACVGQKFAVLGIAMLIASLLRSYEVQPHPALSQEMESLVDSNSLHHIPNPKIILKKRSI | |
| CYP727B1 | MNSVLNSVNDFVSSKSTKEYAKKELNAILWVALITITVFSLEKVFKLFRLWSKASQIPGPPCNSFFGHGNLGSRENFIDLLSVSHDKYGSVFKLWLGPTQLLVSIKDPALIKEMLLKAEDKLPFIGKAFRLAFGRSSLFFCSYDQKRRESLALELNGKMLGRANVIPKNVVDCIMERVDAIMSKGSVDCKSVSQHMAFTILGTALFGDTFLAWSKATFYEELLMMIAKDASFWASYRVTPFWKRGFWRYQSLCTKLKCLTLDIVQQCGKNYGLFSHMDQNSHIGIEKVGIKAASGAPPSNGVEMQDKLFSPELDGHLNEREEPCGNIMGVMFHGCIATASLIGSILERLVTDVEMQDKIYSEIIKVKQGSVKEDQNVEKMLLLLATIYESARLLPAGPLLQRCSLKDDLIFKSGVVIPAGAVLVVPAQLLQMDDSSWGSDASKFNPYRFLSKAGKDSDLVQDTSFTEEAVDPIQCSFILNDPNDNAAFLPFGSGMRACVGQKFAIHGVATLFASLLQRYEVRLDPQLANNPKSTTGPQIVFVRRNS | |
| CYP728A1 | MDASAMLVALLTILATAAAVASSSLRRRKNQPPGSLGLPVVGHTLALLRALRSNAARAAAYGPVSTISLFGRPTAFLAGASCNKLLFSSDKLAAMSSASFLRMVGRRNIREVAGDDQRRVRAMMARFLRLDAVKNYVSAMDDEVRRHLRAEWGGRAAVAVMPSMKSLTFDVMCTVLFGLERRGDHAAVRRELSSEFQQLVRGIWAVPVNLPFTTFGKCLAASRRGRRAVARIVEERRRAMPRGGGGGGSAGDLVTHMLAEGMDEEEIIDNVVFLMVAAHDTTAVLLTFLLRHLDGNRAAYERVAAEQEAIATQRRRRGGSGSGSGSALTWDDLAGMRYTWAAAMETLRMVPPTFANMRKAVADVEVGGYVIPKGWQVITAATMTHLDPTIFPDPGRFEPARFEAAAAKSAPPPFSYVPFGGGARACPGNEFARAETLVAMHYIVTGFRWRLAAGCDGGFSRHPLPCPNQGLLLDIEPKE | |
| CYP728B1 | MALAVVVVALLVAFLTPLAVYLAGRSTRTKPPPRRNLPPGSLGLPLVGQSLSLLRAMRRNTAERWLQGRIDRYGPVSKLSLFGAPTVLLAGPAANKAVFLSEALAPKQPRSLAAIIGRRNMLELVGDDHRRVRGALAQFLRPEMLRRYVGRIDGEVRRHLAGRWAGRRTVAVLPLMKLLTLDVIATLLFGLARGAVRERLAAAFADMLEGLWAVPLDLPFTAFRRSLRASARARRLLAATVREKKANLEQGESSPSDDLISYLVSLRDGDGGGGRPLLTEEEIIDNSIVCLTAGHDTSAILLTFMVRHLADDPAILAAMHEEIARSKRDGEALTWEDVARMKLTWRVAQETLRMVPPVFGSFRRALEDVELDGGYVIPKGWQVFWAPCVTHMDPAIYHDPDKFDPSRFDAQAAASAPPPYSFVAFGGGPRICPGMELARVETLVTMHYLVRHFRWRLCCGGEENTFVRDPLPSPANGLPVELDHIAPLRCDEFNS | |
| CYP728C1 | MDSSMPFALLLALLIPTLLRFVIRRKYSSYNLPPGSLGFPLIGQSISLLRALRSNTDYQWYQDRIKKYGPVFKMSLFGSPTVLMAGPAANHFVFSNQDLIFTQTKAINTILGRSILTLSGEELKRVRSALQGYLRLEMVTKYMRKMDEEVRRHIDLNWVGHKTVKAAPLAKRLTFDIICSVVFGQGIGPIREALATDFETLVQALLSLPVNIPFTKFIKGLRASRRIRKVLRQIAREREAALQQGHSSSADDFFTYMLVLRSEGTHSLTVEDIVDNAIVLLTAGYGNSAVLITFLLRYLANDPDILGKITEEQEEIARRKGPNEPLTWDDVSRMKYTWKVALETLRTVPPIFGSFRTAVKDIEYHGYHIPKGWQVFTAQRITHLDGNFFNDPVKFDPTRFDNHTSIPPYCFVPFGGGPRMCPGNEFARTEILVTMHLVRQFRWKLCCEEEGYRKDPVPIPVLGLPIELETRSPPEYAHA | |
| CYP728D2 | MNPEILFALLLFLLPLYFLLTRRSSKRLPPGSLGLPIIGQTLSFLNAMRKNTAEKWLQNRTRKYGPVSKMNLFGTPTVFLQGQAANKFIYTCDGDTLSSQQPLSVKRICGERNIFELSGLEHRRVRGALVSFLKPEVLKQYVGMMDERIRKHFEMHWHGKQKVMAMPLMKTLTFNLMSSLIMGIEQGSKRDVPVKLFQQLMEGLISVPINLPFTRFNRSLQASEKIREIVMDLIREKRVALDHQNASPQQDLITSLLSLRNDHNSVALSDEEIVDNAIIIMIGGHDTSSILLAFLIRLLAKDPSVYAGVVQEQEEIAKNKASNELLTWDDLGRMKYTWRVAMESLRMNPPVFFSFRKVLKDFNYEGYLIPKGWQVMWAACMTHMDGSIFPNPSDFDPKHFERQSSIPPYSFMGFGGGPRICPGYEFARLETLITVHYLVNMFTWKLCCPEISFSRDPMPTFKDGLEIEIEPKILGEII | |
| CYP728E1 | MDLMILFEHPSFESKVSSPTIILVTLLALVAGFYYKLKASKLAGKKLPPGSLGFPLVGESISLVRAQKRDKIDEWMWKRIDKFGPIFKTSIFGTKTVVLTGQAGNRFLFSGGDGISYKQPKTIASILGKYSLFEISGSRHKLIRGAIVGFLKPERIQKIVGEINSLVQQQLSKELDGVDSVKIVPFMKRIAFNITCNIFFGIPDGKEKDTLFEEFSVAVKGCWAVPLDIPGTVFHRAMQARASLCKILSKIIDERKRQMEEGTVDVNENIIYSFLSLRDENDEPLIEEEILDMVLSLIMASHDSTTILLCLLVRLLSRDAEIYNKVLEEQREVIKVKGGSDGKITWNEIQMMKYSWRVAQEVMRFYPPIFGNFRQITKDIEFDGFHIPKGWQVLWVASGTHMDKSIFEDPEKFDPSRFDTSSKTFPPYTYVPFGAGLRICPGADFVRIESMLVIHHFITKYQWKEIIPDEPIIRDPMPYPAMGLPVKFYPRSGDLAIAGNDI | |
| CYP728F1 | MEAVFGLDKLSSTTVISLATLTTLVAVIWTYRFSLIQRKKLPPGKLGLPFIGESISFFRAHKHNNIGKWIEERTIKYGPVFKTSLMGENVVVMTGEASHRFIFSGRDNGIAAKLATSALAILGKNNIFDLYGSPHKLVRSAIMSFLNSECIQRYVSKMDSLVKEQVLQELNDKETVQVVLLMKKISFIATASLLFGLPEAKERDGLFKDFTIAVKGMWSIPLNLPGSTFRKAVQARGRIFKLFTNLIAERKRGLEDGSMGSHDDVILCLLSLRDENGKTLPDEEIINNLIALMMASHDTTSVLLSLIVRELAKNASVYDKVLEEQNEIAKVRSIASDGQLGWREIQKMRYTWNVAQELMRLTPPIIGNFRHAWRDTTFNGYDIPKGWQVFWLATSTHLDNKVFEDPVKFNPSRFDTNSKSSVPPYTYIPFGAGPRVCPGAEFARTEVLLIIHHLITNYKWTAMVEDEIVVRDPMPFPNKGLPVKIYPKHNI | |
| CYP728G1 | MEVLLSSLEESSFEWKLPLISTILVTLIALLAGSIKLKFSPPVDKKLPSGSLGFPFIGETISFLRAQRQDKTVEWIESRIAKYGPVFKTSLMGSKVVVLTGQAGNRFLFSGSDNGILSNQPMSVAKILGKHSIFELAGTRHKLVRGAIMNFLKPESIQRSVSRMDSVVQQQLFQELEGKDSVQMVGLMKKITFKVTCSLLFGLPDGKETEELLEDFTTALKGAWTVPWDLPGTVFRKALQARGRICKQLAQLVRERKAKIEEGRVDSHEDIISSLITLRQENGQPLSEEEIIDNLISVVIASHDTSTVLLGLLIRHLARDTEVCKKVLEEQKQVAKAKEGKGNGKLTWGEVQMMKYTWRVAQELMRMTPPVLGNFKCAWRDTTFGGFDIPKGWQVFWVAPGTHMDKKVFEEPEKFDPSRFENPSTSVPPYAYLAFGAGPRACPGADFSRVEVLLMIHNLITKYHWAEMIIDEPIVREPMPYPAMGLPVKLYQRSTT | |
| CYP728H1 | MVYGILFFVLFAFTLSLAFLLSKCLSKSQTKNVPKGSLGYPIIGETLSFLKAQRQDKGSVWLEERISKYGPIFKTSLMGFPTVFVIGQEGNKFVLGSPDDLLSSKKPLTLRKILGRQSLVELTGPRYRLVKGEMLKFLKPECLQNYVKEMDELVNATLLREFRENEIIRAVVFMKKLSYEIACNLLFDIKDEHTKEALFVDFTLAFKAIHSLPINLPGTTFWRGQRARARIVDRMIPILNKRREELSKGVLSSTNDMLSCLLALRDENHQPLDDDLITDNFIFLFVASHDTSATLMSLMIWKLSRDQEVYNKVLEEQMEIIKQREGTEERLTWAEIQKMKYTWRVAQELMRMIPPLFGSFRKALKDTNYKGYDIPKGWQVYWAAYGTHMNDDIFENPHKFDPSRFENPTKPIPPYSYLPFGAGLHYCIGNEFARIETLAIIHNFVKMYEWSQVNPEEAITRQPMPYPSMGLPIKIKPRSCSIS | |
| CYP729A1 | MSGATADWAWWLGLVAGAVPLLALAVWHCTDAFHSAAFAFRRRGTRARLPPGHMGLPFVGETLALIWYFNLARRPDAFIEAKRRRYCYGDGDDDGGIYRTHLFGSPAVLVCSPASNGFVFRSAPPGSFGVGWPVPELVGASSLVNVHGGRHARLRRFVLGAINRPGSLRTIARVAQPRVAAALRSWAAKGTITAATEMKNVTFENICKIFVSMEPSPLTEKIHGWFTGLVAGFRSLPLDMPGTALHHARKCRRKLNSVFREELERRKVKMVTGEGGDDDDDGDLMSGLMHVEDEQGRRLDDDEVVDNIVSLVIAGYESTASAIMWATYHLAKSPSALAKLREENLAIAKEKNGDGFITLEDVSKMKYTAKVVEETIRLANIAPMAHRVALRDVEYRGYTIPKGWKVIVWIRSLHVDPAHYDNPLSFNPDRWDKSAELGTYQVFGGGERICAGNMLARLQLTIMLHHLSCGYKWELLNPDAGIVYLPHPRPTDGAVMSFSEL | |
| CYP729A6 | MDAVSGWFVTVVICGLLWWWNVLWYVVPLSLRGKLPPGNMGLPFVGDMISFLWYFKFLRRPDDFINAKRRKYGDGAGMFRTHLFGTPSIIVYTPAVSKFIFRSEDKFMQEWPTIELMGRTSMVAVHGKAHARVRSFVMNAINKPEALRRLAALVQPRMISALESWAKMGKIKAQFETQKMTFENISKSFMSMEPGPFLLSMDKLYKGLLEGVRAYPIDFPGFAYHRSIQCRKKLEEIFWTEFDNRKKESYKLKPNNDLMDGLMQIEDAEGDKLSDTEVVDNIVSLVVAGYMSTSLVSMWAISLLAKYPNVLKKLREENMALEKGSPGDLITANDVSNLKYTNKVVDEVIRVANVAAFVFRKSVEEAEYKGYKIPKGWNVLVFIRYIHTNPEHFHDPMYFNPERWNEPLKPGTNQVFGGGQRLCPGNMLAKIQLALLLHHLSLGYKWELLNPNADTIYLSHPAPSDGVEVNFSKL | |
| CYP72A1v1 | MEMDMYTIRKAIAATIFALVVAWAWRVLDWAWFTPKRIEKRLRQQGFRGNPYRFLVGDVKESGKMHQEALSKPMEFNNDIVPRLMPHINHTINTYGGNSFTWMGRIPRIHVMEPELIKEVLTHSSKYQKNFDVHNPLVKFLLTGVGSFEGAKWSKHRRIISPAFTLEKLKSMLPAFAICYHDMLTKWEKIAEKQGSHEIDIFPTFDVLTSDVISKVAFGSTYEEGGKIFRLLKELMDLTIDCMRDVYIPGWSYLPTKRNKRMKEINKEITDMLRFIINKRMKALKAGEPGEDDLLGVLLESNIQEIQKQGNKKDGGMSINDVIEECKLFYFAGQETTGVLLTWTTILLSKHPEWQERAREEVLQAFGKNKPEFERLNHLKYVSMILYEVLRLYPPVIDLTKIVHEDTKLGPYTIPAGTQVMLPTVMLHREKSIWGEDATEFNPMRFADGVANATKNNVTYLPFSWGPRVCLGQNFALLQAKLGLAMILQRFTFDVAPSYVHAPFTILTVQPQFGSHVIYKKLES | |
| CYP72A5 | MLREVSPWALASVVASVSLLWLVVWTLEWAWWTPWRLERALRVQGLKGTRYRLFTGDLRETARANREARKKPLPLGSHDIAPRVQPMHHSTIKEYGKLSFTWFGPTPRVMIPDPELVKEVLSNKFGHFGKPRSNRIGRLLANGLVNHDGEKWAKHRRILNPAFHHEKIKGMMPVFSTCCIEMITRWDNSMPSEGSSEIDVWPEFQNLTGDVISRTAFGSNYQEGRRIFELQGELAERLIQSVQTIFIPGYWFLPTKNNRRMRAIDVEIRKILREIIGKREKDTKNRETNNDDLLGLLLESNTRQSNGNASLGLTTEDVIEECKLFYFAGMETTSVLLTWTLIVLSMHPEWQERAREEVLSHFGRTTPDYDSLSRLKTITMILHEVLRLYPPATFLTRRTYKEMELGGIKYPAGVDLLLPVIFIHHDPDIWGKDASEFNPERFANGISSATRHQAAFFPFGGGPRICIGQSFALLEAKMTLCTILQRFSFELSPSYTHAPYTVITLHPQHGAQIRLKKLSP | |
| CYP72A61 | MRGLGLNLTPITTFAIITVIATVLIWWFWNALNWVWLRPKRIERRLKEQGIQGNSYRPLIGDIRDMVKMIKEAKSKPMDPHSNDIAPRVLPYVVHTIAKYGKSSFMWLGPTPRVFILDPDKFKEMATKVYDFQKPDTSPLFKLLASGFANYDGDKWAKHRKIVSPAFNVEKMKLLVPIFCQSCDDLISKWESLLSSSNGSCELDVWPFVQNVSSDVLARAGFGSSYQEGKKIFELQREMIQLTMTLFKFAFIPGYRFLPTHTNRRMKAIDKEIRESLMVIINRRLKAIKAGEPTNNDLLGILLESNYKESEKSSGGGMSLREVVEEVKLFYLAGQEANAELLVWTLLLLSRHPDWQEKAREEVFQVFGNEKPDYERIGQLKIVSMILQESLRLYPPVVMFARYLRKDTKLGELTIPAGVELVVPVSMLHQDKEFWGDDAGEFNPERFSEGVSKATKGKLSYLPFGWGPRLCIGQNFGLLEAKVAVSMILQRFSLHFSPSYAHAPSFIITLQPERGAHLILRKL | |
| CYP72A7 | MSFSVVAALPVLVAVVVLWTWRIVKWVWIKPKMLESSLKRQGLTGTPYTPLVGDIKRNVDMMMEARSKPINVTDDITPRLLPLALKMLNSHGKTFFIWIGPLPTIVITNPEQIKEVFNKVNDFEKASTFPLIRLLAGGLASYKGDKWASHRRIINPAFHLEKIKNMIPAFYHCCSEVVCQWEKLFTDKESPLEVDVWPWLVNMTADVISHTAFGSSYKEGQRIFQLQGELAELIAQAFKKSYIPGSRFYPTKSNRRMKAIDREVDVILRGIVSKREKAREAGEPANDDLLGILLESNSEESQGNGMSVEDVMKECKLFYFAGQETTSVLLVWTMVLLSHHQDWQARAREEVMQVLGENNKPDMESLNNLKQMTMIFNEVLRLYPPVAQLKRVVNKEMKLGELTLPAGIQIYLPTILVQRDTELWGDDAADFKPERFRDGLSKATKNQVSFFPFGWGPRICIGQNFAMLEAKMAMALILQKFSFELSPSYVHAPQTVMTTRPQFGAHLILHKL | |
| CYP72C1 | MLEIITVRKVFLIGFLILILNWVWRAVNWVWLRPKRLEKYLKKQGFSGNSYRILMGDMRESNQMDQVAHSLPLPLDADFLPRMMPFLHHTVLKHGKKCFTWYGPYPNVIVMDPETLREIMSKHELFPKPKIGSHNHVFLSGLLNHEGPKWSKHRSILNPAFRIDNLKSILPAFNSSCKEMLEEWERLASAKGTMELDSWTHCHDLTRNMLARASFGDSYKDGIKIFEIQQEQIDLGLLAIRAVYIPGSKFLPTKFNRRLRETERDMRAMFKAMIETKEEEIKRGRGTDKNQRLLFSMLASNTKTIKEQGPDSGLSLDDLIDDCKAFYLAGQNVTSSLFVWTLVALSQHQDWQNKARDEISQAFGNNEPDFEGLSHLKVVTMILHEVLRLYSPAYFTCRITKQEVKLERFSLPEGVVVTIPMLLVHHDSDLWGDDVKEFKPERFANGVAGATKGRLSFLPFSSGPRTCIGQNFSMLQAKLFLAMVLQRFSVELSPSYTHAPFPAATTFPQHGAHLIIRKL | |
| CYP72D1 | MEDFIFRGFLSSSLLLSLYVVFRVAHTFWLKPKSQEKRLRKQGIRGTSYKILNGDMKEFARSSKEARSRPLALNQEIAPRVFPFFYKMVQIYGKVSLCWMGTRPSLLLADPELVRLVLTDTSGHIIKPPRNALVGLLQLGVSTLEGDKWAKRRRLMTPAFHVERLRGMIPAFSACCCDLVQRWKKLAGPQGSCELDVASEFNILASDVIARAAFGSSYEEGKRIFDLQKDQVTLVLEAFYSIYFPGLRFIPSKKNKKRYSIDKEIKAALRNIIHKKEQAMQNGDLGDADLLGLLLKGRDDADNDMKIEDVIEECKLFFFAGQETTANLLTWTLVVLSMHPDWQEKAREEVLQICGKRTPDTDSIKQLRIVSMILNEVLRLYPPVNLLYRHTLKETSIRGMSIPAGVDLLLPFLFLHYDPEYWGDNAEEFKPERFSEGVSKASKDEIAFYPFGWGPRFCLGQNFALTEAKMALTMILQNFWFELSPSYTHAPGNVITLQPQHGAPIILHQL | |
| CYP733A1 | MAVFGAAVLVALAVTCGLIWSRSRRLSKEMRDIPGTMGWPVVGETFSFISGFSSPAGILSFMRDRQKRFGKVFKTYVLGRMTVFMTGREAAKILLSGKDGVVSLNLFYTGKQVLGPTSLLTTNGDEHKKLRRLIGEPLSIDALKKHFDFINDLAVQTLDTWLDRRVLVLEEASSFTLKVIANMLISLEPEGEEQEKFRANFKIISSSFASLPLKIPGTAFHRGLKARNRMYAMLDSVIARRRDGGEVRNDFLQTLLRKHAKDDADKLTDAQLKDNILTLLVAGHDTTTAGLTWLIKFLGENPEALQKLREEHMEIKERLDGSSHLRWSDVNSMPYTNKVMNETLRRATILPWFSRKAAQDFSIDGYEIKKGTSVNLDVVSIHHDPSVFADPYKFDPNRFDGTLKPYSFLGFGSGPRMCPGMSLARLEICVFIHHLVCRYSWTPLEDDDSVQPTLVRMLRNKYPIVAAAI | |
| CYP734A2 | MEEDGGGGAGWGWATWRVAAVAAAAAVWVTMHVAARMADALWWRPRRLEAHSRGAGVRGPPVPVLLGSVREMVALMAEASSKPMSPPTSHNALPRVLAFYHYWRKIYGHRFLIWFGPTPRLTVAEPELIREIFLTRADAFDRYEAHPVVRQLEGDGLVSLHGDKWALHRRVLTDAFYPDNLNRLIPHVGKSVAALAAKWGAMAEAGGSGEVEVDVAEWFQAVTEEAITRATFGRSYDDGRVVFAMQGQLMAFASEAFRKVLVPGYRFLPTKKNRLSWRLDREIRRSLMRLIGRRSDEAEQGEKADDGSFRDLLGLMINAGAAAATRGNAGGEKNSPAAAIPVEDMLEECKTFFFAGKQTTTNLLTWATVLLAMHPDWQERARREVFDVCGAGELPSKEHLPKLKTLGMIMNETLRLYPPAVATIRRAKVDVQLSDGCMIPRDMELLVPIMAIHHDTRYWGPDASQFNPARFANGASKAAKHPLAFIPFGLGSRMCVGQNLARLEAKLTMAILLQRFEIRTSPNYVHAPTVLMLLYPQYGAPLIFRPLSSHPPDSTGP | |
| CYP735A2 | MVTLVLKYVLVIVMTLILRVLYDSICCYFLTPRRIKKFMERQGITGPKPRLLTGNIIDISKMLSHSASNDCSSIHHNIVPRLLPHYVSWSKQYGKRFIMWNGTEPRLCLTETEMIKELLTKHNPVTGKSWLQQQGTKGFIGRGLLMANGEAWHHQRHMAAPAFTRDRLKGYAKHMVECTKMMAERLRKEVGEEVEIGEEMRRLTADIISRTEFGSSCDKGKELFSLLTVLQRLCAQATRHLCFPGSRFLPSKYNREIKSLKTEVERLLMEIIDSRKDSVEIGRSSSYGDDLLGLLLNQMDSNKNNLNVQMIMDECKTFFFTGHETTSLLLTWTLMLLAHNPTWQDNVRDEVRQVCGQDGVPSVEQLSSLTSLNKVINESLRLYPPATLLPRMAFEDIKLGDLIIPKGLSIWIPVLAIHHSNELWGEDANEFNPERFTTRSFASSRHFMPFAAGPRNCIGQTFAMMEAKIILAMLVSKFSFAISENYRHAPIVVLTIKPKYGVQLVLKPLDL | |
| CYP735A3 | MAMAAAVLVAIALPVSLALLLVAKAVWVTVSCYYLTPARIRRVLASQGVRGPPPRPLVGNLRDVSALVAESTAADMASLSHDIVARLLPHYVLWSNTYGRRFVYWYGSEPRVCVTEAGMVRELLSSRHAHVTGKSWLQRQGAKHFIGRGLLMANGATWSHQRHVVAPAFMADRLKGRVGHMVECTRQTVRALRDAVARSGNEVEIGAHMARLAGDVIARTEFDTSYETGKRIFLLIEELQRLTARSSRYLWVPGSQYFPSKYRREIKRLNGELERLLKESIDRSREIADEGRTPSASPCGRGLLGMLLAEMEKKEAGGNGGGEVGYDAQMMIDECKTFFFAGHETSALLLTWAIMLLATHPAWQDKARAEVAAVCGGGAPSPDSLPKLAVLQMVINETLRLYPPATLLPRMAFEDIELGGGALRVPSGASVWIPVLAIHHDEGAWGRDAHEFRPDRFAPGRPRPPAGAFLPFAAGPRNCVGQAYAMVEAKVALAMLLSSFRFAISDEYRHAPVNVLTLRPRHGVPVRLLPLPPPRP | |
| CYP736A1 | MSPPEIAILILVFLTFLWSLLRLINVSSRQSRTLPPGPAALPIIGNLHMLGDLPHRSLQNLAKKYGPIMSMRLGSVPTIVVSSPKTAKLFLKTHDTIFASRPKLQASEYMAYGTKAMAFTEYGPYWRHIRKLCTLQLLCPSKIESFAPLRREEVGLLVQSLKVAAEAGEVVDFSEKVGELVEGITYRMVLGRKNDDMFDLKGIIEEALFLTGAFNISDYVPFLSPLDLQGLTKRMKRVSKTVDQLFEKIIQDHEQVSRSEQGNHHKDFVDVLLSSIHQTLKPNDEEVYMLERTNAKATLLDMIAGAFDTSATAIIWTLAELLRHPKVMKRLQEELQSVIGMDRMVEESDLPKLDYLSMVVKESFRLHPVAPLLVPHQSMEDITVDGYHTPKKSRIFINIWTIGRDPKSWDNAEEFYPERFMNRNVDLRGHDFQLIPFGSGRRGCPAMQLGLTTVRLALGNLLHCSNWELPSGMLPKDLDMTEKFGLSLSKAKHLLATPTCRLYNES | |
| CYP737A1 | SWPAATVAMLGTDSVTFSTGAYHRSLRRLLGPCFSPQAVEGYLPSIQAICERYCAEWAAETTAAAAAAAPAATGGDSSAVIEQLPKLQKGARMLTFEVMSHVVAGFHFSPQQLASLSDAFDVFVRGIFAPVALAIPGSNYAKASAARKVMVAALTQQLELLKGGSGGGGNGGGANGGGDGDSDLAINLLFAGHETTATSIVRLMLVLRSRPDVVSRLREEQAAAVRQHGAAISGSSIRDMPYLDAVVKETWRCHPVVPMVPRRAVRDFTLGGHDVPQGWGVVLGLVEPMRDLPAWSGLTPDSPLHPSHFNPDRWLSGRSSASGNSSNSASSSALQQQDGTATADGDDVASAAAAASVGGGGGAAGSGTLSSPMGMLPPQMLTFGGGGRYCLGANLAWAELKVFVAVLLRGYDFTSPLPELEVKLFPALTVAQGFPIEVRAR | |
| CYP738A1 | MRSSSRGAKIGRAYPTAHHIDGRASGGRPLHFGLHPCHRPCLRAKAAQSGLAELPLPEGSLGLPVVGETLELITNGDTFGTSRRERYGDVYKTNILGAPTVMVAAPMARRYACICFRFSCQVTSTLVGPDSLNLLTGPRHGAVKRALSDAFADRALRRHVPAIAELVQAVFDRVVLGGAGSRDRAAQLQAVMSALQAGFNTPPVQLPFTAYGKAVAARQEFGQLVSQSIQRSRQHTAASATVSVSPSSAPAFDCAMSDVVAAAAAAAATGTALPDSLLVDNAAAAFFGNASTGPSLAKALQHLATNAAGPNGGATGGVMAALRQEQDIVSRHGPAITAEALDEMSYGTAVARELLRITPAVPAVFRLALVDFELQGRRIPKGWRVWCHVGDSVTRYNKDQFQPERWLGSSGMAAGGCPMHAGGGGAARGAQPEYSLPFGSGVRTCLGRNLVMTELLVVLAVLARGYEWEAVNPAEQWGVVPSPAPKEGLRVRLHRRL | |
| CYP739A1 | MAVFGFRELFASMYIPGLSPVLSTITCLAGVLLFLAWQRHSRATSVPRLGPLLTIPLLGDVAWLAADPTRFVFGRFQRYGPTFILNLMGVPLYVLTQPADLRGPYRDQGAEPDVPFSSFRRLMEVAPGRPYDVQADKAAHGPWRRMFLSALGPAGLQALLPRAQAVMQAHLAQWEAAGTAAGGRSGGGCIPSLFRQVRLLSVDLAIEVIAEVPLPPGVERIAFREQLLCFLDGLFGLPLALPGSSVARALAAKEELVAALGPLVAADRQRMAKRWRAAGSSYAALVDTLTAASAAVGGSAAAEAAAGVQAAEPSAAAAARVTVRDAVISGFMALGRAAAVSVLHAVVAGADTTRFALFNTLALVAMSARVQEEIFAEQERVVAEHGPELSARVLGSAAITPYLDAVVREAMRLLPATPGNMRRLTADLRVGAGRGGPASELVIPKGSMVWRFVPLMHCLDPVLWDGDTSVDVPAHMDWRSNFEGAFRPERWLSEDTKPKYYYTFGSDNHLCVGQNLAYMEVKLLLAMLLRKYRLQLHTPDMLARASQMFPFVIPRRGTDRVLLEPR | |
| CYP73A5 | MDLLLLEKSLIAVFVAVILATVISKLRGKKLKLPPGPIPIPIFGNWLQVGDDLNHRNLVDYAKKFGDLFLLRMGQRNLVVVSSPDLTKEVLLTQGVEFGSRTRNVVFDIFTGKGQDMVFTVYGEHWRKMRRIMTVPFFTNKVVQQNREGWEFEAASVVEDVKKNPDSATKGIVLRKRLQLMMYNNMFRIMFDRRFESEDDPLFLRLKALNGERSRLAQSFEYNYGDFIPILRPFLRGYLKICQDVKDRRIALFKKYFVDERKQIASSKPTGSEGLKCAIDHILEAEQKGEINEDNVLYIVENINVAAIETTLWSIEWGIAELVNHPEIQSKLRNELDTVLGPGVQVTEPDLHKLPYLQAVVKETLRLRMAIPLLVPHMNLHDAKLAGYDIPAESKILVNAWWLANNPNSWKKPEEFRPERFFEEESHVEANGNDFRYVPFGVGRRSCPGIILALPILGITIGRMVQNFELLPPPGQSKVDTSEKGGQFSLHILNHSIIVMKPRNC | |
| CYP740A1 | MAPLLDAKQLELLGIGMQLAAVLLVLYYLLKWLAGKRGGVPGPAFYLPAIGETLSLFASPTRYMWKNWLEYGPFFRTHLLGYPLYVVGSPGLLKPVLGDDSAFEFFVPGKTFTMLISDIRHMQVPEQHAVFRRRLGQALNPGALSRHVMAPLRVVLERHLDAWEAAGRVQLAEACAAASLDVALEVLTGVPLPAAPETRAEVRRGTGGLFRTALAGLYGVPLPWLPGTAIHSALRAQRRLMALLGPELDREVAELAGKSRLPTGGTAWHETHLAHARTPRPGSAACPRGPTADAGSRRSHRHRHHQLLLRHRGAHAHAGGPGRCGPALPHRHAFLRTGTPLSLTKEQIFERALGVVIASDDTSKHLFFFELVAAAMLPGVWAKLEEEQKQAMRKYGDELSYSILNDMPYLDAVIKETIRVFPTAVGGFRRALKDVPVEGGQLIPAGSIVFYSTHLLNAADPALLPRSLAPEALEGPTGLPAHLDYECRLEEAFRPERWLSDETRPRQFAGFGGGQHLCLGMHLAHAEARMLLALVVRRFHLRLEQPQLLSRVTYFPGPVPRKGADGLVLMPRRLEP | |
| CYP741A1 | MDGFWKTLGLGALLSPVLYALYLASLIVIPYLKSLPLRRKLRHLPGPPVTGFFLLGNVPDLVRTPVHQCMARWAEQYGKIFKLELPTMTAVVLTDPEAVSQVLKVDRFEKLTTSYQNMEKLTAEQQPNILTEPLSAYYKAVRRAVTPAFSTANLRRFFPLLLDITQQVMTGLAAAGPSAALDLDRVAQRLTIDVIGRFAFDRDFGATADIAKTNEALQVVGELMTALQRMLNPLNRWFWWRKEARGLWASRRRYDALVRRALEDLRSSPPAQHTLLHHLMSLTDPDTGKPLSARRLRSETALFWIAGFETTAHAIGWTLMFIAGSPEVESRVAAELEGAGLLAVPGRPEPRQLAWGDLGGLKYLNAVIHESMRLMPPTSGGTVRVVPRDTQLAGHVLPKGTMLWIPFYAMQRSERVWGPDAAQFRPERWLAAAAGAGGPGARGFLPFSEGPRNCVGQSLALLELRTALALLCGSFRFRLADDMGGVEGAVSEARQHITLKPGDRGLLMHAIPRVPA | |
| CYP742A1 | MHTAPRRIHAARCRPLHASTGASTPGPAGAPDLPPLQRAPGPPGLPWLGQLPAYLATKFFPKKMLEWSEQYNGVYAMEIVGRKYLVVTEPSLIAGIVGRGSAGLPKSTGYAMWDSAISPHAGVQGLFTVAENTTTWRAVRRAYGPAIGPGSMSSGTSTSTSSSSTASINSTTGLTSHEMNHLAKCLTLDMLGLSAFGIDFRCLDDPAAAQLPSLIESAMHECGERARSVGRRLLPWLYEEEARAGAADMAAFHALVEDVWRQIRARGAPTEDDNSFGAQLLRLADPSLAPGGAALSDEQICAEIATVIIAGYETTANTLTWMLYGLHAHKDASEQLVAELRGAGLVPDTSSSSSPSSVDPTTASFASLAGAHEALGGLPVLDAYVRECLRLYSTAPNGLIKEVPKNGPPARVGPFAADPGVVVWIPFWSLHLSNLNWEQPHDFQLSRWLGKDPRTAGSLTASRCPVSGTLNALRAATSSSSSSSSSSSSSSSSSSSSSSGSDSDGEGGSSSGGRGSKAIRFMPFGDGSRNCVGQHLGMLQLKLSLAYLAARFDLVLDEARMGGSAAAALERQRVNLTLEVDGGMYLLGASVHSHARVYWYQLVSCEPKC | |
| CYP743A1 | MLRALSCLALLAAGAARLAAAAGATDSAVSRALAVLALLLALHVLADPLQRWRLRHIPGPPALPLLGSVPAMMRAGGPFFFRQCFAKYGPVFKVAMGRKWVVVVADAELMRQAGQRLRSHVIIEPNLNRGHLRRLDAEGLFQAHGEFWRLLRGAWQPAFSSAALSGYLPLMSACGLRLAQQLQAGGGARPAAGYVDVWRALGGMTLQVVGSTAYGRLAVACGDVFRFGSALHGSSYQRIGLLLPELVPALVPLAHSLPDPPFKRLQRARSTLLAACMELIRSWRQQHHATTLARGEINSERTHERETRTAGGTTATGVAAAAEAPAAMCGAAVPAAAAAVDGAAAPAGPEEADAAARGGGVGGGGGDGSGVGGSGVAAGSFLDLMLAARDKANGAALTDRMVAAQVQTFLLAGYETTANALAFAIYCVATHPEVESRLLAEVDAVLGRDRPPTESDLPRLPYTEAVLNEAMRLFPPAHATTRIVEAGAPLQLGGVSLPPRTPLILAIYSAHHDPAVWPRPEDFIPERFLPASPLHSEVAARVPGAHAPFGYGSRMCIGWKFAMQEAKLVLALLYQRLLFRLQPGQVPLPTATALTLAPRDGLWVRPVLRRAARAE | |
| CYP743B1 | MVASASWQLDLLGALSGAPSPQMAAAGLALLLASLLIYLLDPIQRWRLRKVPGPPARPLLGCLPQLRAQPMPLFLQSCAQTYGPVFKASAEVQGIAVIPHHVSRMQVALGRKWAVVLADAEMQRQVRGTGAERGGSTWRQLRAAWQPAFAPASLAGYLPLMTGCADQLARRLEAKATAAAGATASGATAGGGSSVDMWRELGGMTLQVVGSTAYGVDFHSINEEDQAGSGSGSGSAIATAGATAAAKGRGDDGYGKQLAAACGQIFRYTSSAHGSPYLRVAMLFPELRRLLVPLAHTLPDKRFAILMQARNRLSGAVFQLMDSWKQQHIAAAGSGAAGKGSSGKADASNGVGAAATSGRGGMAGVAPGSFLDLMLGHRQGGGSGSGGKKAEGEEGVEHAPLTDEQVAGQVQLFILAGYETTANALAFAVYCIATHPEVESRLLREVDDVLPGSDQLPGESDLPRLAYTEAVVNEALRLFPPAHLTSRVVPPGETLTVGGFNIPAGIPIFLPMYIAHRDPAVWPRADVFLPERFLHPRGAAQQHAHAPFGYGSRMCIGYKFAMQEAKVALATLYRRLTFTLEPGQQPLQVEASLTMAPRGGLRVTPVPRRKL | |
| CYP743C1 | MTFLQLLPGVPLVLLGVLALPVVITLVQEVITKRKYRHIPGPKPQPISGNLREFLTSPGGLLGCLEGWVKQYGDLLTFRLGSRQFVLVADPDAARPVFTARVFLTQIVFPHTARSLRGYQALMDREAVALAGRLRRQAAAGGGGGGGGGGGGGGGDKAGEIEVMSEMSRVTLAVVGTAAYGCNDFFRTMSPAARSSWSWAVALPCLLPAVRHLAAAAPDPVLALHIQSRQVLRQVSTKLITAWRDSHTAASANGSSTNSTSGSSSSTGVAPGSFLGLMLAARDRSRKEGGAAATAKDGMAPTLTDAQIEAQVQTFLLAGFETTANALTFAVYLLACHPEIQGHRIPAGSTLWLSIAHLHTRDGVWPEPQAFRPERFLSPDVPGSAPELAARHPHVHLPFGSGPRMCIGWRFAMQEAKTVLSRLVQAVDFTLAPGQAAPLDTVAGLTLAPRNGVWVRLSPRGGGGSGGGGGRGQEVATAAAKGAAVRSAAA | |
| CYP744A1 | MALSSAWALAGLFLAMFVFFGYSLRKRWQLRKIPGALGWPFLGSIPEFSIYGYEYVLGLSAKLGNAWLGVEPLIIICDPALIRKYAYKCVSKPPSMSEYGHVLTGFNYDVDQASAFVASGEVWRRGRRVFEASVINGVRLAAHLPAINRCANRFVAQLAQRVAAPAAAHSGKTLGEEGIDMFSIVGGYTMAVTGEVAYGHVPAVTRGVRPFWQVEHSTLYLPLGVMFPWARPLVRWLATHFPDRAQREHMAARTQIIANISRLLMERWATSKKAAAAAAGTGTGTGTAITADSKAGTASAPPAEAARADGAAAAGKGAEEAIKEVGGGISSSSFMAAMMEGRRGAPQEERLSDVEVIAQSFTFVMAGFETTALTLSLVTFMLATHPEAAARLTAEVDGLGPGELTHEVLAEKLPYTEAVIKETLRLHPPIPYFIREAREDLDLGNGMVAPKGSYLTMYMHAVHLNPDVWPHPERFLPQRFLPEGSAAFGPADPGAWAPFGIGARMCVGHKLAMMMAKTLLVRMYQRFRIELHPRQPLPLKMKTGLSRVPVDGVWVTLTER | |
| CYP744B1 | MELVSGLALAGVALFILGFIWAGFNPIERYLSPLRRFPGPAPLPFLGNLVSVATRDLTAYLADCRQAYGGIWLGNQPWVCVADPDLIRRVAYRVLSRPFSHTDSIHLLAGEQWEVDCNTLVFLKNGPTWRLARRAFESSIIHPQSLAGHLPAVWRCVRRYTPRLERHAATGEPLDLSSDLADLTLAVVGEAAYGVDFRTTDEQQDGGRPADPSAPGPALVAAVRECFDCLDVNKTTMYGPLKMIWPGLTPLWRWMAKHLPDAAQTRHMRVADVSRQLMAQWQAAKAKTAAAADTAGATAASGAGAEAGAGVGVGAGAQAKPGGGGAVQAFVEVGGGISSSSFMASLLEGRRGAAKEEERLTDLQIVAQCLTFLLAGFETTAATISFTAFCLATHPEAQARLLAEVDEHFARQAAAEQQQQGQQQREGDDALPELPYLDAVLKESMRLYPAGSALIRKSPQPLDLGRDGLVIPGNTFVCLATHAVMHDPAIWPEPEAFRPERFLPEGSSSLGPMVGGAAASAPAGGGADAAAAAWVPFGMGPRMCVGSKFATMVSKAVLLQIYRRFTFELHPKQVLPLRTRTALTHAPRDGIWVVVKAR | |
| CYP744C1 | MQLTWLGWAPVTRWRLRNIPGPFALPFLGHLPAISARDLVHFCHDVARQYGPVWVAARPWIVVSDPVAARKIAYRSLARPSTVASFTHALVGEPRQVDDESIFWNRGPAWKASRRAFETSVLRPDRLAAHMPAVRRCTERFLARLAPYADGSTAVDMKDEYGVIALAITGEVAYVSFWPSDEDAALLAAPTGGSGAATSSSSSSSSSSKSPSSALVRACHECMACFELPLATMYLPLQMLLPALRPLWLALAAALPDAAQRRHMEARQAVADVSRRLMREWQQQAAARANDSGGDGLLLKDQTPVVNGGSSSSGSGGISSSSFLAAMLKDQTGSNTACASSSGTDGGVISQGLSFILAGYDTTGTTLALTTFLLAHNPTTQEKLRAELVENRELLDSADGLAQLPYLDAVLKESQRLHPAVGHFWRDATSDIALPEMGGLVIPKGSFVSISIYNMHRDPAHWKEPERFIPERFLQATGGALGPTDPGAYVPFGSGPRMCVGYKMAIMVVKSVLAGLLLRYRVALHPRQPLPLRLKTGLTLEPADGVWVTLQPLLLPGAK | |
| CYP745A1 | MASSSSPLEELLAFAGVKDGTISSPRLALVVLGAALAAYALVFAVINVVDYIRIARGLSAIPSAPGGVPLLGHVIPMLTCVSQNKGAWDIMEDWMDAKGPIVKYNIAGTQGVAVRDPKAMKRIFQTGYKLYEKDLKLSYRPFLPILGTGLVTSDGALWQKQRMLMGPALRVDVLDDIIRIAKKAIDRLCEKLSHHAGKGDIVDIEEEFRLLTLQVIGEAVLSLGPEECDRVFPQLYLPVMNEANRRVLRPYRMYLPTPEWFRFSSRMGQLNGFLIDLFRRRWQARQAAAAAAQGEGSSSSKPKPADILDRIMEAIEESGAKWDAALETQLCYEVKTFLLAGHETSAAMLTWSTLELAAHSQAADKVVEEARAAFGPRGESEAGRRAVDEMIYTLAVLKEGLRKYSVVPVVTRVLAEDDPQGLLGYPLPRGTMVACHLQGTHRLYESPDEFRPDRFMPGGEYDQFDDADRAYMFLPFIQGPRNCLGQHLALLEARVVLGLLHARFSFKPAPSVHPDPASLFMRHPTVIPVGPIRGLKVLVEQRK | |
| CYP745B1 | IFQRHCVVVADPELVKRVMQTNLKNYKKDTEFSYEPFLEILGTGLVTSEGETWRAQRQRISSALRIEILDDIIAIATRALEKVRGKGEAVELAEEFRLLTLQVIAEAILSLTPEQSDEVMPNLYLPIMDECNRRSLEPWRKFLPTREWREHRKRVAALNKYIVDLIRVRWKKRVSGETNPNPDILDRVLASVEMEEYGSDVEEQMCFEIKTFLLAGHETSAAMLVWTIYELVKNEDKMTEAVAEANKVLGAVKPGNLPTRDELAHLDYCVSALKETLRLYSVVPVVTRRAVEDDVLGGCKIPKGTTVIISLQGIHHREDLWPNAMSFEPERFLNGKGDEIGNYAYLPFIQGPRNCLGQYLALLEARVVLATLIRRFKFKSASANNGKKHTKAIPIAPADGMWFTVE | |
| CYP746A1 | MLALAGGLQSMLQVSSPLVTHKITYGSLRLSSPPPPAFPAGPSGDQTLPLLTDPLRFLTDATATYGPVVGLLLGGERVALVTGRAEARAVLVEAAGEVYVKEGTAFFPGSSLAGNGLLVSDGPVWQRQRRLSNPAFRRAAVEAYGGAMVAATEDMMRRVWGPAGGTRDVYADFNELTLQVTLEALFGFSEDAAQIVAAVEKAFTFFTQRAATGFVIPEWLPTWDNLEFAAAVQQLDRVVYGMINRRRQELAAAFAGVPSDLLTSLLLARDEDGSGMSDQALRDELMTLLVAGQETSAILLGWASALLAAHPEVQAAAAAEVAAVCGGPEAGTPTPASVRHMPYLESVVLETLRLYSPAYMVGRCARRDAALGPYVLPAGTTVLVSPYVMHRDPEVWEEPEVFRPERWQELQRSNLGPNGAYLPFGGGPRNCIGTGFAMMEALLVLAALLQRYSLALPPAAGSSSGGAFPKPKPLLTLRPEAVVLRISPRRQ | |
| CYP746B1 | MGSISAANLELIATLASHCLQRTTESVKQGQAVLQQAVQPLPSCFPPGPNGDVALDFARDPLECLASLKSRYGSLVGFKLASRPIVLVSSPNFSREVFVTQSSTFIKAGTAFFPGSSLAGNGLLVSDGDIWKRQRRLSNPAFRRAAIQTYAEQAMVNITEKMVDKVWRTGGVRDVYADFNELTMEIVASALFGASEASEEMAQVGPAITQAFQFFTRRATSMFIVPEWVPTFDNIQYNNAVTDLNKVVFRLINERRRQLANSSAPPRKDLLTRLLHVNEDGSGMDNQSLRDELMTFLVAGQETSAILLTWALLMFALHPHTQELVFQEISEVLNGQLPRQTDVSKLRYLEAFIWETLRLMPPAYVVGRCACHPTELGGYKIPQGTTILVSPYLLHQDPAFWPRVSEFDPSRWMPGGDATEHMENDSFWPFGGGPRNCIGMGFAMMEVTLVLAVISSRFRVSLPVGEPIPSPRAMITLRPESEVKLRLTSRRQQRQRKSEAADEMKVIVCLN | |
| CYP747A1 | MKSALSAFVRDSGDQVAETGAPTATRPIPGPAPLSLEALKDVSVIFFEGLHVAQLKFSEKYGPVCRFANPASLNGATSWVFINSPENIQHVCATNVRNYSRRYLPDIYTYVTHGKGILGSQDEYNARHRRLCSGPFRNKWQLQRFSSVVVERSKRLVDIFSAAAAADPSGAFTTDVATQTQRLTLDVVGLVAFSHDFACVEQVQRRDLAGATAGDGRSGVLQDRVLWAVNTFGEVLAQVFITPLPLLKAMDRLGAPHLRQLGEAVSVMRAAMLDVIAATEDDGRGLSDEELWEDVHDIMGAGHETTATTTAALLYCISAHPHVRQRLEEELDAVLADGEAPTYESLERMPYLQACAKEVMRLYPAIPVFPREAARPDVLPTGHGVAAGDVVFMSSYALGRSEAVWGPDVLEFDPDRFSPEREARQHRFQWLPFGAGPRMCLGASFAQMSVALMAATLLQRFRFTPLAPCSPLIPVGYDITMNFGPSGGLRMRVAPRQRGQQQ | |
| CYP748A1 | MSSALDELRFYGTLAATLLGPRYDLGRVPGPPGHPLLGNITAVMRPDYHVQMLEWANTYGGIFKFSLGFQPVVVVSDPAVAVQVLGRAPGRAIPRKCVGYKFFDLATNASGAHSFFTTSDEGQWAAVRKAAAAAFSSANVKKAFPIALRHLLLLSLLHVFVEALFGVTPEDFPGRQVAADMNLVLEEANSRLKVPLSGLARAVTQPVVGWREGGTGHVSRGFGARNSRAWGSGEKEWTEENWEPRAVTDLWACLGRVRHPRTGELLGRQGLVPEIGALMMAGFDTSSHSVAWALFALAANPEAQQRVRQELDGRGLLRRPGTAAPPRLPVLDDLPQLPYLNACIDEAMRMYPVAATASVREVTEPTRVGDFVIPPGVIVWPMLYALHNSVHNWDQPDVFKPERWLQSNAGGSSGKGGGGGKRYMPFSDGMKSCLGQALGLMEVRTALVVLLGRYAFALDPGHGGEAAVRRSMIMSLTLKIRGGLRLVATPLG | |
| CYP749A1v1 | MANPLIYFSGCLFLSILIILIKFFNKVWWTPIRIQSLMKSQGIRGPSYRFLHGNTKEISTMIRKTRSSPQELLHHTLPMVHPHFYSWIKLYGMNFLQWYGPQAQLIITEPELVKQILSNKDRAYPKTKVSNEIKKLLGDGIVLSEGEKWVKLRKLANHAFHGESIKGMVPEMIASLEIMLERWRHHHSKEIDIFVEFKILTSEVISRTSFGSSYLEGQHVFDMLTRMTHIISENNYRVRIPGIGKFVKASYDIEFENLEAKIRKSFMNMMKRREKDAMLGELDGYGHDLFGLLLKAYHDSDETKKISLDDLIDQCKNFYLAGQETSASALTWIVFLLAVHSDWQDKARKEVLELFGLQIPSQDRIAKLKIMGMVINESLRLYTPNAILMRRVERETKLGKITVPANTEVYISTLAVHQNPEIWGEDALLFKPERFADGVVKATNNNIAAFMPFGLGPRNCAGMNFAITETKLALSMILQRYSFTLSPTYAHCPTEVLTMCPQHGVQVILQRYEHIALKV | |
| CYP749B1 | MMTAICFVFLVGLALVLARFLYKSWWYPVSLQLLMKSQGIKGPPYKAPNWNYAKGVLDMEVKSTSAPMEISHDIIPRLFPQVYSWINLYGKNFLHWIDTQPQLVVTDINLIKEILSDKEGSFDKVQLEGVLKKFLGGGIVFEEGKKWSKLRKVANHAFHGQNLKEKVPAMVASVEELLKTWKSYEGKEIEVFKEFKLLSLEIISKSVFGSDYLTGKTMYHMLDEIVLICYKIIADKFSKSSHELKVADHILQAFVDSLVGIMKQREDKVKAGQSNNFGSDFLGSLMESHHNTDQNKRISVVEIIEECKTFYFAGHETVRSVLSWSILLLAVHTDWQDTARKEVLEMLGQGNPNIESISRLKTVGMILNETLRLYPPLVFLHRKVKRNIKLGELRLPAGMEVYIASLAVHHNSEIWGEDTHLFKPERFAEGVAKATRDQLMAFLSFGFGLRKCVGFNFAQMEVKIALCMILQRYRFTVSPNYRHFPTLVMGLWPKHGIQIMLHPL | |
| CYP74A1 | MASISTPFPISLHPKTVRSKPLKFRVLTRPIKASGSETPDLTVATRTGSKDLPIRNIPGNYGLPIVGPIKDRWDYFYDQGAEEFFKSRIRKYNSTVYRVNMPPGAFIAENPQVVALLDGKSFPVLFDVDKVEKKDLFTGTYMPSTELTGGYRILSYLDPSEPKHEKLKNLLFFLLKSSRNRIFPEFQATYSELFDSLEKEAFPLRESGFRRFQRRNRLLFLGSSFLRDESRRYKLKADAPGLITKWVLFNLHPLLSIGLPRVIEEPLIHTFSLPPALVKSDYQRLYEFLRIRGEILVEADKLGISREEATHNLLFATCFNTWGGMKILFPNMVKRIGRAGHQVHNRLAEEIRSVIKSNGGELTMGAIEKMELTKSVVYECLRFEPPVTAQYGRAKKDLVIESHDAAFKVKAGEMLYGYQPLATRDPKIFDRADEFVPERFVGEEGEKLLRHVLWSNGPETETPTVGNKQCAGKDFVVLVARLFVIEIFRRYDSFDIEVGTSPLGSSVNFSSLRKASF | |
| CYP74B2 | MAATSPRPPPSTSLTSQQPPSPPSQLPLRTMPGSYGWPLVGPLSDRLDFQGPDKFFRTRAEKYKSTVFRTNIPPTFPFFGNVNPNIVAVLDVKSFSHLFDMDLVDKRDVLIGDFRPSLGFYGGVRVGVYLDTTEPKHAQIKGFAMETLKRSSKVWLQELRSNLNIFWGTIESEISKNGAASYIFPLQRCIFSFLCASLAGVDASVSPDIAENGWKTINTWLALQVIPTAKLGVVPQPLEEILLHTWPYPSLLIAGNYKKLYNFIDENAGDCLRLGQEEFGLTRDEAIQNLLFVLGFNAYGGFSVFLPSLIGRITGDNSGLQERIRTEVRRVCGSGSDLNFKTVNEMELVKSVVYETMRFSPPVPLQFARARKDFQISSHDAVFEVKKGELLCGYQPLVMRDANVFDEPEEFKPDRYVGETGSELLNYLYWSNGPQTGTPSASNKQCAAKDIVTLTASLLVADLFLRYDTITGDSGSIKAVVKAK | |
| CYP750A1 | MSFDKLLQALPPPLPLPAILIATFIFFFSCWILHQSQRNERLPPGPYPWPIIGNFHQVRLPLHRTLKNLAEKYGPILFLRFGSVPTVVVSSSEKAKHFLKTHDLIFASRPPTSVGKYFFYNFKDIAFSPYGDHWRKMRKICVLELLTSKRIESFKHVRQEELSAMIHSIWEESESGRIAVNVSKAISTSLANILWRILARKKFSDNDLGADGKGFADLVVEVSIAVGSLNIGDFIPYLDCLDLQGIKRALKKANARFDAFAEKMIDEHINASTIRNGEADAGCHVKDIIDVLLEMAKNDNTGAKVTREIIKAITYELFSAGMETSANVLEWAMSELLRHPHAMKKLQQEIESVVGQQGTVKESDLASIVYLHCVVKETLRLYPSLPLALPHESLEAVTVGGYYIPKKTMVIMNLWAIGRDPSVWGADASEFKPERFMQMEENGIDLSGGQSDFRMLPFGAGRRTCPGSAMAILTVEFTLAQLLHTFDWRVEGDPSELDMKEACATKMPRQTPLLAYPRLRLPRCP | |
| CYP751A1 | MAAWKRESVVSVFATNESAAIGVCDVTPVHETPLWRYVKHEQNTLAWVSLIVVTFLLSRRLCSVLRLLILGYRLPGPRARAFDGRSQCDDIVELLARLHQEHGPLVKVWTGPAQLLVSVKDVDILQHVFERAHDRVPVLRMALQLLYGRRSLFTSNYSKVSCRSLINGLVLRQAHISSIEVAEKMTQLGGLSKNGCHDLDCMTFSKLMAFAALGTSLYGDGYMIWPVAREFERVMMEVMEALPIWMRYSVPPLWNAKFVVFWKQCLRLRDLARELAAHGNQTSIQESEDRVEGLNILGKLLEEFVSVLFSRMGMGSSTVAEPGAAGMMSHGSLNTAGVLCNVLAQLARHPHIQTKVHNEISTISGFDKSLTETDVQKMIYLNATVLEAARLLPTVPFLQRCSDEHDIALLPGVVIPAGAILTAPIQLIQRDTVYWGDDAAIFNPDRFLKPRRIASGELASEQNQNIPHPCNFTKEPLQLNPAFLVFGAGSRSCIGSSLAVKQISILVTVILKRFEVMLYPTLFISKL | |
| CYP752A1 | MEKDDDVSFDVALPTIGLETTHSKFPQLVLSAIGLIVVCGGAYVLYNTHYLRVKIKLPPGPPPWNVFCTSMQSKKPCQALAKICNCEYGGIMTLSLGKFPTILITSTVIATQLHVLKRYKFKFGHPKNIPRPCEYLHPDNYQNLKCVLPYNWTQWHKLWQIYIDHLLPVAHNMSFQSINQLDIQIMLKNLENEMTKEGGMKPFGIGLRPHLRHASFKFIFNICFGRHVDAIAGGVGSHKDPLMMQLEALFIEVIRLGPAFIISDFVPTSLPFHSPIDIQRAAISGTMKKYKTFYHRANIKVPRSEPVDLLDHLVCLQKDEQLQDKEIVWLLSELILASTDHVSTILEWTFAHLMANPQVQAKLHQEIDIVCSKRTNISTTEFDNMPYLVAIVKESMRVSSPIMLTIPHSTTKELNIGGFQLPMNTQIVCHLGALGQDANIYENPSCFDPNRFIGIGVNLNNAFEKQKNIVHLMTQQFCPGRGLEILHVYIFLVKLLQCFEFSHLYVEIMPFKTSDTVEWGVINVLRKPLVACLNPHL | |
| CYP753A1 | MANYIIASNALVLLAFVTFFFVYFLRAFILRDKKLRPKYPPSPWKWPILGNLPQLLRGGPACHTTFRLLAKELGPVYNVWLGGSFPMVIVTGEETVHEALIKQSSVFSSRPKLLSWQHISAGFKTTMTSPFGPHWQKLRKTISVDLLGPSKLASYKPIRDSEIQKLLARLREQAHANAGLVSPLDQLRTSAVDVIMRIGFGEEFALMEAVNSNRRHAKIVELDRCFRQLMDAGSIFQLVIDSSVVARTLLFPLARSANRNIETVADNTVSLVMPIVQQRKRYLQDHPATETRTFVDALISCKGESALTDLEIVWNVVELMVGGTDNTSHILEWALANMVKYPHIQEKVYTEVRCAMGPNLERRLVEESELDKLPYLQAVVKESMRRHMMTPLAIPKLAAQDCKLSGYDIPKGTMVVFHAGALAMDDDIWTDPLNFRPERFLAGTGSSNAPVTQTHKHAFMPFGAGRRSCPGAAMGFLHLHHLFANLIYAFEWGPESPRKAVDFTEKFRMVVTMKNPLRATIKERTHFRMM | |
| CYP754A1 | MVEESWLWVLFVGALSFSILLQWGLNRKRKLKLPPGPTAWPIVGCVFGLPRLNPPEKLFNKLSEKYGELMLLQLGSWSIVVTSSARMAMEILKTHDNEFANRPDVISSRLNFNNTGLIQMHSTNPLFKRTRRMFSAEIVSPRTVLETGVIRRKQLRTLRSIVQDFDAGRSVNFTHEMKTLAMNLSMSICFGTDYATKVNDEAEALIHTYKIMAIWTRRSLGAIFPALRWLDLDGIESGFADVELQLRTNITALIEKKKQEMSMWSAEDIQAGANEGDVMTKFLSMEGEDRCSEDQLISVVFTILLAGTDTVFNVVTEAMYALLMHPNFYHRAVEELDAVVGKSRLVEEADIPKLPMIQNIIKETFRIKPAGPSLVPRKNFEACEVAGYHIPANTTVFVNCIPLMRDPSFWDSPDEFNPDRFIDSKVTVLGSDFNYLPFGYGKRTCPGLNLGMITVQYILAACLQCISWKLSRPRRLDIETDDDPRKVDDVMVDGKQRVDPALLEFAPQPVK | |
| CYP754B1 | MVGDVWIWVLITMVVAVIVGVGIDKKTKRGLKLPPGPPAWPVVGCLASLPAGHPPEVMFAKLAEKHGELMLLWLGSKPYVVASSARMAMEFLKRHDQEFANRPMSVVREYVSFKGNSIISMSASDPKYQRLRRTFVMELLSPKKIAATRDLRKDQVLKMLRAIREDLDAKHEANFTEAVLTLGMSLSIGLLFGRDYGGKVFSEEIQTLVLTFKTMVKYLSMINISDLIPSLRWLDLQGIERGLGLGEVQLRKSIMALIEQKRLDKIRLSSDEIESGACQRDILSKLLSLEGEDRLDDDQLMGVVFALMLAGSDSISRGVGRAMQELLKQPLLHQRALDELDEVVGRRRLVEESDISSLPLINNIIKETLRLHPPAQLLIPHGNVEQCEVAGYHIPARSTVLVNLYALSRDPSFWNSPLEFAPDRFVDSNLTVQGSDFHYIPFGYGRRGCPGLNLGMITVQYALALCLQCILWRLPAGATISETYIDWKNSPDLIVDGDLRVDLHLLEGL | |
| CYP755A1 | MVFTVRREVADWSHSFPPMSYGTAVVVGFVLLLLLLVFGYSRRVGKKKTLPPGPFAFPVIGNLFLVGKHPHVTFAKLAKQYGNIMRLHFGAVPVVIVSDANMARELFSVQDMKFASRPIYDLMSTAYKYMNYGTDEEVSLAISEYGPKVRDLRQLCTTELFTQRKIDMKKSVRAEEIQRMFGKIKTMIRDEEPVEIRPIVSEFSLRISCRTTFNKAFLNFENLPWRPGALHPQAFRNMETENTKLLGEHQILDMIPMLKFVLERFDVFGINARWKEVSALKEECTRPVIEWYRKHSSDDESTLDFVEVLLRLSEEGKLSKTCVKSLILELLTAGSDTIASVLEWTLLELVRHPHGMERLSAEIDGFFGINRPVDEDEFTKLPYLQAVAKEVLRLHNPTTLGIPHSNMEEATLAGYHLPARTTVLANFWAISRDPTTWGQDALTFNPDRFLACDLNVNGTNYEYLPFGAGRRICPGRAVAMRVLAAAIGSFVHAFEWSALPGVELNANEGKDGLNIRPETPLVLKLSPRPSAMLY | |
| CYP756A1 | MAHLHTRLSEEAEAWIATGVDSFSRWQEYAAGFGRATYIVAALGFFAVVILELHNSRKRRLSKLPPGPFQWPYLGSLPNLLLTVGVTSSFRLREKVSELGRNHGPLMFLQIADTQILIVSSGTAAKEVLIARDEEFNFRPQCAVGKYLGFGSSDIAFAEGRHHWYLRKLCDTRLFSADSFVSYGHIPRAEALKMLHSVWEASKKGNGISVRETVTAFVRNSLCGMLLGSAHLDIENVSLQFTEKTLITLLDETICVVGEITLSDLAPGLKRVDFHGRTRKLKELHERWEKYLRVILEDRSHRLEKSAKPEALVDVLLSLDDADMKLSNEAIMGVLLDTLVGGVYSTSATIEWALAELVRHPGVLEKVQLEMSEVVGPYHIVEDAEISQLPYFQATVKETLRLHPVVPMSLPHMNKVATSISRYQIPANTSVVIDYKAIARDPAAWHKPLRFDPSRFLHTSAASQIDNIFKFLPFGYGRRGCPGANFAAVLLQLALAHLIQAFDWAPTKGQLPHDIDVKESPGLVCFRFSPLVLSSTPRLANSLYQVSP | |
| CYP757A1 | MMEIGGMRAEWHVVLSACVTIATMVLTIMKLRKKIGKLPPGPRALPLIGNIHQIGDFSRRNLMQMAEKYGPIMYMRIGSKPLLVVSTAEAAHEFLKTQDKEWADRPTTTADKIFTNDHRNIVCAPYAAHWRHLRKICTMDLFTPKRLMSFRTPRTEEINQMMTSIHEDVAAGKEVKLHVKLGHLTTNNITRMLLGKRFFTVDEKGQMEAHRFKELVFELFRASSTPMIGDFIPWLKWVSIASGYVKYLKRVKADLDAFLQEFLEIKKAASDQATAERAKDFVDLLLEQKTVSGDGPLEDATIRSDMLLAGTDTVSNAMEWTIAELMRHPECMRKLQQELDTVVGKSRIVSETDLPNLPYLQAVVKEVMRFYPPAPLSLPHQSIVPTTVCGYDLPAGTQLCINLYAIQRDPKYWPNPVQFNPDRFLNCDVDVGGTHFQLIPFGAGRRQCPGMPLGNLLLQISVARLVQAFEYSLPRGTKRNYFMNYYSGANKLTSGIMYLICYPAPHYSCOMITRELLAPATENSMOSSMATPDSSGGAFDLAKWINGLVAHWGSVAVAVVAAAVIAKFIFNSTVGRRKLPPGPAPWPILGNIASLAGLPHRSLEKLARKYGSLMYLRLGEVPCIVISSADVAKQLFKTHDILFSNRPGGCFFEQLTEYRNITASRYGPHWRHLRKTCVHELFTQKRLEAYQATRLEEISISIKELFEESDKKGPVDLHAWLHRLLFNNLTRVIMNNRYFGTDEKGMKDAMDFNNVTALMFSQAGDVVISDFLPYLGFLTRLQGKPLLYRKTREIVLEMMRRMTNFDERKKLHAEGRSTGEPEDFVDVLLSSTLSDGTTPLPDDICLMLLMDVLVAGTDTSATTVEWTITELLRHPEAYKRVREELNSVVGSDQLVKEEHLEHLPYLNAVLQESFRLHPATPLGLPRESSEAFEFLGYSLPAGTRLFVNQWAIHRDPAVYEQPEEFNPERFLGREALKFIGDTQFQLVPFGSGRRNCAGLPMAVIVIPLVLAHLLHSVEFSLPDGQQPKDLDMTETFGVAAPKASPLMIYATPRESAALY | |
| CYP758B1 | MLATAFLVGFLAWAAMILGKFILEGIQRRNLPPGPWAWPIVGSLFSLGPLPYKTLRVLAKKHGELMYLRLGSIQSVVVSSASMAKEVVTNHDLQFAYRPTKLFGKLLFNSKDIVHASNGPAWRHLRMICTSQFFTKKRLASYEATRTFEIHTLMKDILRKSSSEDCVVNLPFQLRNTSTNFISQMVFNKRYFVEGEESNVEDAKRYQKILKIHFSSYAIFVVSDYIPCLRFITKLQGIRGKFQQIADKIHKKMDEIIDINGHERRRIDANHKQDADRKKDFVDLLLETTSHDGKGTLDHETVRGDMLFAGAETQSSTLEWAMAFLIRNPGVMKQVQAELDGVVGTERVVQESDLEKLPYLEAVVKEVMRVKPGAPIGINHESREPRQVAGHYLPAKTRLIFNIHAIHRDPSVYDRPDEFDPTRFLSPGKGNVPTGQELFQLMPYGAGRRICPGMPLAIVNIPHVLAHLVHSFDWSLPAGQDHRELDMTEKFDGVTSPRLHPLHLIPHPRKPAFLYK | |
| CYP758C1 | MMDSASYSAPFAALWDTFGRGTVVAVLVVVVVGELLLYARFQAQRRSTLPPGPRPWPILGNFFVFSDVNHAHHDLRRLAAKFGPLMYLQLGSVPCVVVSTAEAAKELFRGHNDECLISRPKMLGLEILSDNYQLMAYAPAPGKLWHSLRKFGSMELFSFKRVAFYRSLREEELRHWIKFVLESREGEAMNLKSCVFELAANMMTRMLVNKRMFDITGADTQQQLLRSEFESFMEEHYKCLMPNVISDFLPFLRFFCEKLQGWRAYIQDHQEKSVEFWTRIIEVEKHRQRAAERQNDGSYVPDLVDFMSTAPLDDGKVLSDRNITLQILDFFLGGTDTTPLTLEWAMAELVTHPNFMKRAQEELDRVVGLERLVEETDFPNLPFLQAIVKETYRLHPVGPLGGPRESTEPVEALGYKIPAKTRVILNIFAIHRDPAVYERPDEFDPTRFLDRPLAAFDSYELMPFGVGRRMCPAFNLGNTTVHLILANLIHNFDWALADGQNIDTFDMTERLHGVTFSLKYALSLIPTARSGILARAL | |
| CYP758D1 | MGFVEMTQNWRLWLQEGSNVSVYGTVLFVMFTTSCILHVLSAIERRKKLPPGPWPWPFIGNLGVVLRKTGARHKFLQALGAKYGGLMYLGLGQIPCLVVSSVRVVESMFKSHDATFSDRLQTYFRKVQYGDSAMRSLSSAGYGSYWRQVRRMCNTELFSPGTHASQEGVRREEIQNMLDVLVHECKRRKPIDLGDWLFGVSTNNMTRMLINKRYYGTGAEIPEKKEEFQGMVKSRTRAAGTFVISDFIPSLTFIAKLQGLPKRFRESHESAKAQMESVLDVEEHRKNAIARASVDIKSEYSPDFVDVLLKAPLDDGQPLADSDIKFLLTDLMIAGTETTGITVEWAMVELMLRPELRKQAQEEIDAVVGADPERFVQESDIQKLPFLVAILKETFRVHPVAPLNVMRSSYEPCEFAGYYLPAQTRLIVNQYAIHRDPSVYENPDKFEPRRFMENPEVNPLSGRDSYQLIPFGVGRRMCPASNLAFTMALLMLANLLHTFDWSFPDGVTADNFDVSEEFLGTVLRKKTPTILMAKPRSHVQ | |
| CYP758E1 | MASAHSHTRRWWSQEAHGIRVSGEGTIATLLISSLVIYVTVVYQRRKKLPPGPWPWPVVGNLAVLAGLPHRNLQNLAAKYGGLMYLQLGQVPCLVVSTAAAAKELFRTHDVIFSYRPKRLDHEIISGKSYKSLTSAPYGPYWRQIRRICNTELFSPAIHASHVSVRSEEIHSMMKVLLAESRTEKAIDLKSWLTGVTANNMTRMLINKRFFGTGVSDQQEKKDFEEIFDHIFAAAGTFFISDFIPKLRFVEMLQGKIAKLTAFRKFLHSVIGKIFEVEKHRQRALERGNDPIYVPDFVDVLLNTPLDNGERLTDREIISILSSMIGAGTDTTATTVVWAMSELMVNPKIRKQAQEELDAVVGDSRLVEESDIPNLPFLRTIVKETFRLHAPVPLSLPRCSEQPCEVAGSQFPANTRLILNVFAIHRDPIVYENPDSFQPSRFVDHPEVDHMSGKDFYGLIPFGAGRRMCPGYHLGNVMVSLMLAHLLHSFDWRLPAGVTEENLDMSETYKLVGLRKKPLFLIAKPRSPAYLY | |
| CYP758F1 | MAEGGLLFGFALADVLVAAVLISVVVLYFHAETLQRRRCPPGPWPWPVVGNFSALGDLPHRNLAGKYGGLMYLRLGAKPCLVISTAAVAKEFYTTVDASFASRPKRFSWTVWNNNDENYRNIGLAEYGPYYRKLRRLLNTELFSPRRHASHEVTRAQEIQCMMKVLLEESEKGNPVNLQTWLHGTTSNNMTRMVVGKRFYGVRVDDSEKERQDLQKMTSSVFELLGSVDLSDFVPYLSFITKLQGHASKFSKIRDVSDKLTADFFDLDSHRNNYKKMKNDPSYVPDFEDVLMETPFENGTNLPDQDLLKLLQELLNAGTETSSNTSEWAMAELIRRPELIERAQTEMDSVIGSKRLVEESDIQQLPFLQAVMKENFRLHPPAPLLLPHESREPTELLGYHFPAGTELLVNAFAIHRDPSVYDNPDSFDPDRFLARPHVDHMSTSDPYELMPFGKGLRMCPGYRLANTMVALMLANLLYVFDWSLPEGQTEVDMTETIGISVRKKQPLFLVPKPRFELSLESVAEN | |
| CYP758G1 | MGALDHSNDMWLQILLALTLVSVVLTWILQCSSSAQKVHPPGPTPWPVIGNLFLFFRAPLPHRMLHNLAEKYGDLMYLRLGFTPCIVVSSPALADYIHKNHDTEFSSRPDGLITGILNGDSQSVSMAKHGDLWKTLRSICWQILRPANIARYETRRMEEINIMLQSIQIAAEAGETVDLSSMLYKLSSNSMTQMLINRRYFTAGGNEENLREAVIFKKMISERLKIASQFAIGDYIPYLRFIDYLFRYNAKAQEIQSMTMRVCDEIMNLEERRRRLTREENGDAQAVREEDFVDDLLSIQAEYTADNSRKIKLTDHQIKLLVQDMLVAGTETSATTVDWAMAELLCHPKVLQQLRSEIVTVVGSRSAVTEQDTKQMPYLNAVVMETLRLHPAAPLNLPRESKGACLFLGRYQLPAKTRVIFNTHSIHRSLEAYDSPNAFKPERFLGVPQANVSGSSFFQLSPFGFGKRVCPGQALGTISVCAALANLVHRFAWSLPCGLPPSHLDMIESFGLTAPRRCPLILLPTPRLKV | |
| CYP759A1 | MEFNQLVSVAAVVVVLGASLVFLRLFTKKKLNLPPSPKGRMPIIGHLHLMDDNEAAHRTFARISEQNGPLTMIYMGNKPTLLVSTAAMAEQVLKHNDQAFASRPFITAGKTLGFDFKSIVFAPFGNYYRRLRRIYTVELLSPKRVALSQVLRQHEIKHVINSVLAENQAEGRVNMTSILQEMGIDNLVRMIFAKPHMGATECLTKEEMATLKSVVKEAVNLAGVIYVGDFIPLLDIYDFTGYKKKTNKLAAKMLDIATQLIEKHKSDAGTGVDNDKLNLVDILLSQKGEDQLPPHAMAGILFDFIIAGSDTTSVSIEWAIAELLHYPHYLKRAQEEIDQVVGKERLVTEQDIKHMPFLQAVVKELFRLHPAAPLGIPHCNMEETKLAGYDIPAKNTVMMNLWAIGRDPAHWDDALEFKPERFLNKDITLLGRDFHLIPFSVGRRQCPGAGLGLAVVQLAVASLLHGFEWSTYNQKPEEIDMREKPGLVTPRKSDLIVTAVPRLPLHVYQGDKNGVQNGH | |
| CYP75A14 | MVSLNLNEFMLWFLSWLALYIGFRYVLRSNLKLKKRRLPPGPSGWPVVGSLPLLGAMPHVTLYNMYKKYGPVVYLKLGTSDMVVASTPAAAKAFLKTLDINFSNRPGNAGATYIAYDSQDMVWAAYGGRWKMERKVCNMHMLGGKALEDWQPVRDAEMGFMLRNILSHSQRGETVNVPDLLNICAANMIGQIILSKRVFETEGDEANEFKDMVVELMTCAGYFNIGDFIPSVAWMDLQGIQRGMKKLHKKWDALIQRMIDEHQSTAKQRASKPDFLDVVMSQRDNCDGQGGRLSDVHIKALLLNLFTAGTDTSSSVIEWTLAELMNNPKLLKRVHEEMDAVIGRERRLKESDLANLPYFVAVCKEGFRKHPSTPLSLPRVSTEACEVDGYYIPKNTRLMVNIWGIGRDPEVWEKPEEFNPDRFVGSKIDPRGNDFELIPFGAGRRICAGTRMGITMVEYNLGSLIHAFDWDVPPNQEGLNMDEAFGLALQKAVPLVAKVSPRLPLHLY | |
| CYP75A17 | MDSLLLLKEIATSILIFLITRLSIQTFLKSYRQKLPPGPKGWPVVGALPLMGSMPHVTLAKMAKKYGPIMYLKMGTNNMVVASTPAAARAFLKTLDQNFSNRPSNAGATHLAYDARDMVFAHYGSRWKLLRKLSNLHMLGGKALDDWAQIRDEEMGHMLGAMYDCNKRDEAVVVAEMLIFMANMIGQVILSRRVFETKGSESNEFKDMVVELMTVAGYFNIGDFIPFLAKLDLQGIERGMKKLHKKFDALLTSMIEEHVASSHKRKGKPDFLDMVMAHHSENSDGEELSLTNIKALLLNLFTAGTDTSSSIIEWSLAEMLKKPSIMKKAHEEMDQVIGRDRRLKESDIPKLPYFQAICKETYRKHPSTPLNLPRISSEPCQVNGYYIPENTRLNVNIWAIGRDPDVWNNPLEFMPERFLSGKNAKIDPRGNDFELIPFGAGRRICAGTRMGIVLVHYILGTLVHSFDWKLPNGVRELDMEESFGLALQKKVPLAALVTPRLNPSAYIS | |
| CYP75B1 | MATLFLTILLATVLFLILRIFSHRRNRSHNNRLPPGPNPWPIIGNLPHMGTKPHRTLSAMVTTYGPILHLRLGFVDVVVAASKSVAEQFLKIHDANFASRPPNSGAKHMAYNYQDLVFAPYGHRWRLLRKISSVHLFSAKALEDFKHVRQEEVGTLTRELVRVGTKPVNLGQLVNMCVVNALGREMIGRRLFGADADHKADEFRSMVTEMMALAGVFNIGDFVPSLDWLDLQGVAGKMKRLHKRFDAFLSSILKEHEMNGQDQKHTDMLSTLISLKGTDLDGDGGSLTDTEIKALLLNMFTAGTDTSASTVDWAIAELIRHPDIMVKAQEELDIVVGRDRPVNESDIAQLPYLQAVIKENFRLHPPTPLSLPHIASESCEINGYHIPKGSTLLTNIWAIARDPDQWSDPLAFKPERFLPGGEKSGVDVKGSDFELIPFGAGRRICAGLSLGLRTIQFLTATLVQGFDWELAGGVTPEKLNMEESYGLTLQRAVPLVVHPKPRLAPNVYGLGSG | |
| CYP75B1 | MATLFLTILLATVLFLILRIFSHRRNRSHNNRLPPGPNPWPIIGNLPHMGTKPHRTLSAMVTTYGPILHLRLGFVDVVVAASKSVAEQFLKIHDANFASRPPNSGAKHMAYNYQDLVFAPYGHRWRLLRKISSVHLFSAKALEDFKHVRQEEVGTLTRELVRVGTKPVNLGQLVNMCVVNALGREMIGRRLFGADADHKADEFRSMVTEMMALAGVFNIGDFVPSLDWLDLQGVAGKMKRLHKRFDAFLSSILKEHEMNGQDQKHTDMLSTLISLKGTDLDGDGGSLTDTEIKALLLNMFTAGTDTSASTVDWAIAELIRHPDIMVKAQEELDIVVGRDRPVNESDIAQLPYLQAVIKENFRLHPPTPLSLPHIASESCEINGYHIPKGSTLLTNIWAIARDPDQWSDPLAFKPERFLPGGEKSGVDVKGSDFELIPFGAGRRICAGLSLGLRTIQFLTATLVQGFDWELAGGVTPEKLNMEESYGLTLQRAVPLVVHPKPRLAPNVYGLGSG | |
| CYP75B3 | MDVVPLPLLLGSLAVSAAVWYLVYFLRGGSGGDAARKRRPLPPGPRGWPVLGNLPQLGDKPHHTMCALARQYGPLFRLRFGCAEVVVAASAPVAAQFLRGHDANFSNRPPNSGAEHVAYNYQDLVFAPYGARWRALRKLCALHLFSAKALDDLRAVREGEVALMVRNLARQQAASVALGQEANVCATNTLARATIGHRVFAVDGGEGAREFKEMVVELMQLAGVFNVGDFVPALRWLDPQGVVAKMKRLHRRYDNMMNGFINERKAGAQPDGVAAGEHGNDLLSVLLARMQEEQKLDGDGEKITETDIKALLLNLFTAGTDTTSSTVEWALAELIRHPDVLKEAQHELDTVVGRGRLVSESDLPRLPYLTAVIKETFRLHPSTPLSLPREAAEECEVDGYRIPKGATLLVNVWAIARDPTQWPDPLQYQPSRFLPGRMHADVDVKGADFGLIPFGAGRRICAGLSWGLRMVTLMTATLVHGFDWTLANGATPDKLNMEEAYGLTLQRAVPLMVQPVPRLLPSAYGV | |
| CYP76 | MDYLTIILTLLFALTLYEAFSYLSRRTKNLPPGPSPLPFIGSLHLLGDQPHKSLAKLSKKHGPIMSLKLGQITTIVISSSTMAKEVLQKQDLAFSSRSVPNALHAHNQFKFSVVWLPVASRWRSLRKVLNSNIFSGNRLDANQHLRTRKVQELIAYCRKNSQSGEAVDVGRAAFRTSLNLLSNLIFSKDLTDPYSDSAKEFKDLVWNIMVEAGKPNLVDFFPLLEKVDPQGIRHRMTIHFGEVLKLFGGLVNERLEQRRSKGEKNDVLDVLLTTSQESPEEIDRTHIERMCLDLFVAGTDTTSSTLEWAMSEMLKNPDKMKKTQDELAQVIGRGKTIEESDINRLPYLRCVMKETLRIHPPVPFLIPRKVEQSVEVCGYNVPKGSQVLVNAWAIGRDETVWDDALAFKPERFMESELDIRGRDFELIPFGAGRRICPGLPLALRTVPLMLGSLLNSFNWKLEGGMAPKDLDMEEKFGITLQKAHPLRAVPSTL | |
| CYP760A1 | MEDNRMGDGQVEEYSSRVMHLSALTLCAAMAVILLRRVMXSWNADKTLPPGPKGWPIVGSLYSLGPRTIPACRRFTTLADKYGPVMFFRLGSRPTVIVSNDKMARELLRVHDQTFASRPKLATGKHFGYNYSSVVFSPSGAHFVRMKKIYTHELLSPKKVELLSALRMEEAHILLVDVLRNSGTEANGVVNITSLVFKANLNLMGRIVFSKRLFGESATISAPPREVENFKFFVKSATKLVGLFNIGDYIPALRWLDLQGVEGALLQLKPHQEGLLRPIIQEYRKMSLNLEGGMKQKEDGRVDFIAALVSNDSGLSDENIMAVAIDVMVGGSDSTSTAVEWSITELLRHPDCLQAAQEELDSVVGRDRLVEEADCANLPFLNCIVKETLRLHPPSPLAIPHFSAEECTLGGYRIPANTTAYVNIYAIGRDAATWENPNRFNPTRFKDSKVNVYGHDFNLLPFSSGRRGCPGVHFALPTYKLELANLLHCFKWSPPPGVDFKDIDTKEAVGVVCSRLNPLMASVTPRIPRHVILAK | |
| CYP761A1 | MVPAGYGGVSEFPFLKFAVAVLGVYFVAVLIRGASRKLPPGPVGFPIIGSVHLLGPRSHVSLAQLARKYGAPLMSLYLGQKLFVVASSAEAAMEVLKKQDAVFCSRPPLRGFKVIFPHDVTFADLTPESNYLRKFIRLHLTTARSIEAFQHIRVDEMLQMVRSIVASPRDVVVNLRTSLEVMTANVLTRSIIGKRFMGRTGLSESEKKEIMEFIHIAAEIGECLGAKNPGDLIPALKLVDWNGLDQRMKNLRRKMATFLANIVRERREKSSLGTSNPPGKEMLGVLLDEMENAAAGEKITEDILNTIIWESFTAGMETTVLATDWTLAEVLRNPEVLQKCQAELDAVVGRNRRAQESDIPDLHYIKAVVKESFRLHPVIPLLIPHYSHDPIKVLGYDIPAHTQLLINVWAIGRDPKVWADPLKFHPERFLEGPHRETEMFGKSFNLLPFGSGRRACMGITLGTLLVEASVVVLLHSFDWILPAEGIDMTEGQGLSVRKNVPACAFATPRLPPHVYAE | |
| CYP761B1 | MPFSGGQGTFMFQGSAIAVVAIFLLARFITTPKNIPPGPFAWPIIGSLHLIGPYPHRSLAKLAEKYGSLMSVWFGQRLIIFATSPETALEFVKTQDANFCSRPKQQAPSVLLPHDLTFSDVTSHSKLLRKIFQQQFTTSKKMEATQQLRANEFAHMLRTIPHDTTVNVKFHLEVLAGNIFSQLVMSRRLLQPSSIEDTTTDSTEKLKDLMKITADLDRIIGTFNPGDFIPAVKRFDLAGIGCKFKQFRNRMDSFVEKIIQERLEERKSSRAPKELREKDYLDALLDEADQQKEIDLNVVKTMIWEIFAAGMETNIASSEWAMAELVNAPHTMKKAQAELDAVVGRDRMVKESDLPNLPYIKAIAKESLRLHPPVPFLAHQCIKSCKAFGYDIKSGTSVFVNVYGLGRLESIYPDPNTFNPDRFLPGGSNVGLDYQGQNFELLPFGSGRRICAGMPVASLMVQTAVATXLHAFTWIAPKDHELMEGLGAASLSKAVPLKAHATPRLPSHVYSL | |
| CYP761C1 | MAAFAVPTLKNVYYVATVLVVILLVRRLLTWPHQPPGPPGLPLVGHMHFLGANPHISLWKLADKYGPLMSLRLGNKPYVVATSPETAKEFLKTLDANFGSRHYSSQSQYLLYGGQDVAFQESSPSWRNLKKIFTMELASPARLEASRHIREEEMIVLLRTIHSKGELELKSQLIDMISHVISRMVINKRFDDSVESDFPTLVQTHFRLAGAFVPGDYIPAVKWLDLGGFEAQMKKQKERMDAFIDDILVQHRERRAKGPVPMKEYDMVHVLLDRIETKDDQIQLTDTHVKALVLDAFLGASETIILTSEWAMAELLRHPSLMAKAQAELDAVVGRDRMVTESDLRHLTYLNTIIKETFRLHPAAALLLPRESAQPSQAFGYNFPAKTRVLINCYAIHRDPAIWHDPLVFNPDRFLQADLKDVDVKGRHFQLLPFGAGRRVCPGLSMGILTVQFILASLLHSFDWSLPGDMKPEDVDMTEIYGLTLPRAAPLPCAAKLRLPSHLLTTAQKP | |
| CYP761D1 | MASSSLDFPTFFIIGATVFAIFIFKKFLTKHSNLPPGPIALPVIGSMHLLGTSPHHNLQKLSTKYGPLMSIRLGQAQCVVASSTETAMEFLKNQDSNFTSRPALRVGEAVFYGQDLVFQNSTPLWRHLKKIFQVEFTSTKRLDTTRHVREEEIAHLTSTLPHNCEVNLRIHLKSMIGNIISRMAVGQRLCAKPEECESEEQLREVASLREVMDNVAFCIGAVNLADYIPALKWLDLQGLERRFKKTFQIMNSVSGEIIAKHQERRKLSNPTDKQKDLIDVLLDDMEKPQDGSPRVTMDSIKAVTWNAFAGATDAIAMSLEWAMSEILLHPHVQAKAHAELDVVVGKNRRVEESDIQNLSYIGAIIKETLRLHPVAPMLAPHAALNPCKAFGFDIPGGTWVIINAWAIARDPAVWKDPTEFNPDRFMQDDPNALNPRVFEMLPFGAGKRMCPGVAMANVTMQRAIAKLLHEFWGLTSELDMSEGTMSIVVPRAVPLHAVAKPRLSSEFYT | |
| CYP761E1 | MDFAKSTVARISFEGLKPEDGLSNQRVEIIVFLAAMFILPFVLLKLMRRPKLKLPPSPPAYPIIGHLHLLGKLPHHSIANIAKTYGEIYSLRLGSVPAIVVTTPEMAKEFLLTHDKIWASRTVRDVSGYYLSYNHTGIAFAPFTPVWRNLRKICTSELFTQKRMEASQGVRDVEMQCMIRSILNDANQRRLIDLKLEVNALTANVVTRMVLNKRFMRCVDSTAEEESRAQQFKEIMKDHFTLQGIFMIGDYIPWLRPLDLGGKEKRMKALRKRLDAFLNEILDDHEVKRAKGPIAEEDQDMIDVLLNEMHQQDPNEPHKMDLNNIKSTILNMFAGGTDTATITIEWAMSELLRNPPIMAKLKAELDALIGQDRRVRETDVPNLPYLQAITKETFRLHPAGPLLVPHESTHDCEVAGYRIPAGTRLFVNIYAIGRSSKAWDRPLEFDPERFMTGPDASVDTKGKHYRLLPFGTGRRGCPGMSLGLLLVQFTLAALVHALDWSLPPGMDPEDVDMTEACGLKVPREHALSLNAKPRAAAQFY | |
| CYP761F1 | METSQLSDYWAGSQLLGNSSFGPGVRVDSVSGSQYFVVEFFLSAIVFTVFNLVFQRLHEPSLIPPRLSAWNFLCQTHVLRRNPTVVLHNLVKRYGPVTHVKLWSQDLLVLSSVXAVEEFYKLHDMEFGDRPSSMNRVTLSNSINSSCFPPLATYWKHLRFVLVSASTIPSFSSSFAFFRMEWALEALHHHPSIVAQVSEEVERSLGSRSHIEDSDLAKLPYLQAVVKELFRLYPPCAFSFPHESFDEYCHIFGYEVSPRTQVLINIYTIQRDPAVWTNPNEFNPTRFITHPGIDMHGQHYQLLPFGGGRQCPATKLAIRYVQSGLARYFHDARSSHMIPHSTCLEDDL | |
| CYP762A1 | MATVSLQEPGLVVGLFLGAPLLLFLYILYYAISLHTTSVEGVRVPRGNFWLLPLLGESISALTVPPKQFIDRQTRKYGAMFTTHIGGDPMIMTTDVDLTRWVYQQTNRLFSVLSPKATYELLGHESIFYAKGDHHLRLRKVFAGYLSTQKLVPFTPRIDKMAASIMESWKRKERVIVFDEAKMYAIHLALAQLISIDTQEYPCMDHIFAHVPGENRLEKLVYLHYDIESGMMSVPLNIPGTAYHKANKAKILFRKALKVIINERRTGDVKCNDLLEGLLSPLEDGTLLDDEQVMDNVITGVGAAEVTTTTALVWMVKWIQENPELHRELQNEMDAIKKTKANGEELTYDDIKKMNLTLWTMYETLRLRKVTGFFIARTADQDVRYKDVVIPKNWVVAMTHGYHLDPNYYPEPEKFNPYRFQTMPPAHTFTPFGASVRLCPGKEMAKIEILTFMYHMLTSFSWEPAEPEGETIWHLFPHPRNKLPIKVTPRT | |
| CYP763A1 | MAELGVSEMERMNTFGIGADAQRGLGAGMTLPLLFLATVVWWIWQRHKANLESGLPGTFGLPFIGETLTYVAKMKSPLGNFVDEKTKRYNGAQAFKSSLFFQPTVIATEVETVKMIVAKEGRSFVSNYPSSFALLLGRFNGLNMNGENWKRLRKFVISHIMRVDLLKERMADIEDLVVRTLDSWADDEGRTIYVEDETKTIAFNITALIVLNLKPGKVSQTMQRDYYPLIEGMFSLPINLPWTIYGKATQARVRILKTLEEFLQSRTVKDDVFDNYVQLLQEELPPGSPPALKHEMGLDLLTSLLFAGHDTTAATMVFSVKYIGENPKVLAELRREHEELLKRKQPGERISWDDCKTLSFSNSIITETLRMCNISTTVFRKSLEDVHVGDYVIPKGWLVLPYFRAVHFNPSIYPDPYTFNPFRYQDAAGSKLPFFGFGGGARLCPGMDLARAELCLFLHHLVMKFESWELLGNDVVSYFPFPRLSARLPIRVKRRTPPQQPST | |
| CYP763B1 | MDGRLFLQGLETVAFVCVSVLLISQLWPKNEERAKINTRLPRGSYGLPLVGETLKYMASMMTSAPAFMAEHRQKYGEMFKSKLMGAFCIITTKADTIKWVLNHEGKQFVTGYPKSFRKVLGEYAALSLHGDQWKSTRRFLVNSLRVELLRERIPTIEQAVLENLNPWAAKESVSIREETKTLAFNVVAQYLLGSRLKSGPVNDSLRNDFYTLTEGLFALPINLPGTQYRKGLEARARIIETLERDVVSHARPVGDEDQYADYMDYMRKENLPGTTEELLLEKTRCHVLGMLFAGHETAASAMLFAVKYIMDNPRVLNELRAEHENIRISKFEGGSLTWDDYKNMRFTQSVITETLRLANPVALLWREATEDVQLNGYVIPKGWKTVCAIREAHHDPALFDRPSEFNPWRHEQEVMNPAKKLPLLGFGGGPRYCPGAELARAEICIFLHHLVTKFDLKSCGEETVSFFPVPKFSNGLQVQVQERDLSTRISHKIRVH | |
| CYP765A1 | MPLAVAILYAANKLALAPALLHTMTIITILTWILGGALTLGLGFIVKEWLWNPLMLIELCKRQGIKGFPFVPFVGQMPAIDEVLSGRNRRVQKQDNDEVEDEDRLTAVTNCYRNHGSTFYFTVGRTVRLSIADPPLIKDILIANSESYSKPLHIRKLGVLGDGIFASSGSTWSPQRSLFTGAFHTKEVKSKIPTMIDCAHSAVEKWSRELNDGYSELDMYQKFAELTLDVIGKTAFGTEEIGGASEAASVIGSFNRYLLYCRELVFGPPATFPTSLKWLRTYMGRIISARRNSHHSGAAETVSDRHDLLDVIIGAVDNIGHSEEGAKKALNEAPDQTISEKRKRAAEMTRLTEKRLLDNALTVLLAGHETTASLLTWTIYLLAEHPLWQKRARAEVEEFCPGGVVEPQVLSHLKLLGMILLESLRLFPPVPLIGRMCIKDNKVGPDLLIPEGLEIVIPVAVLHRDRTIWGDNADEFAPARFGNGISGACGNPLAFLPFGAGPRTCIGQTLALSEAKAVLAVMLPLFSWKLSTSYRHSPDVTLTMMPEFGMPVVLEKIEK | |
| CYP766A1 | MMVEYSQSWTALAVVELSVVAITAVFVPLWNVCSTFLLEPLRLRRVMGKQDVRLAPFNLVFGNAFEIGAHAQSFPETLPLKFDDLEPTATPQFDLYFSKYGKRFLYHVGSETRLVVRDPEMAKEVLFNRMGWYERSPLDLHIFSQVIGKGMFVVKGEEWEMQRRMLNPCFSNESLKPMVERMVKSAAQEMRNWEEMAAQAGGRVEHDVEHDIHIIAYNIISYTAFNEGFDKGKQIYLMIYLMQDEIMGHLFAAGNPSFWIPGLRVLAGLLPTKHATAIAQLNGRTEKLIMELVKDRREAVQKGERDSYGDDLLGRMLTATERTDGSSHKFILDAVINNCKNFFFAGSDSAANLTTFSLLMLANYPEWQDRARKEVLEVFGDNDPCEMNDISRLKIVGMISQEIARIFAVSPSIARLAVKDCELGDLLIPKGLVIEIATLAMHRDPELWGKDVAEFRPERFANGASAACTHHQAFLPFGAGPRSCIAEKISWLEVKVVLCMILRRFRILPSPKYKHHPHFAMVNRPKYGLPLILEILPQSRSDSIMAEI | |
| CYP766B1 | METVPVNVRNALAVVVASVIVYSVIKFLRVSVWQPLRLRRIMAKQGVSGPPFRFVRGQFVEMWKFTESFPDALPIDDFANLTPTVTPQNALYYPKYGKIYLYWWGTITRLAVRDPKIVKELMVSNHESLTRLQSESQFLAEVVGKGLLTQVGEKWASERRTLGPFFHQKSLEGMVGAIMEGAATELQKWEQEVEERGGTAELDVEPDLHKISGRIISRTAFGDEFEIGEQIFKFQTLLSQELLKGFRSTAYWLVPGYRNLPTKRNRSMNLYGSQVDALVRGIINARREAVQKGVTSSYGDDLLGRMLTAATEGWSANTKEFNQLAVFNICKFFYFAGQDTVANAIGFMILMLALYPEWQDRCRQEVTEILGDEQDWRASDISRLKVVGMVFNETLRIFPPASTLTRVAAKDLQLEGLFIPKGMAIEFSLAAMHQDKDYWGDDVGKFNPERFVNGAASACTHPQAFSPFGLGPKFCIGNNFAVMEAKIVLAMMLRRFQLVLSPNYKHHPTSIMVQSPKFGLPIILKALKIT | |
| CYP766C1 | MVFTQWVRFAALAIPEDVRNALGVVLLAFVASAIVRVVFSLVKTYLYDPLSIGRIMAKQGIEGPPFHPIFGTTAELNAYVKSVPESLPLDEDHDSMRTVSPHFHMYFPKFGKRFLYWRGPHAKLVSKDPGLAKEVLLSQYEFFQRHPQDIKMLSNFVGMGLDNLTGEKWAIERRTLNPFFYHDPLKGMVEGMVKGAEPVLKSWEEEVARAGGTAEFNLEEDLHTISGNIIAHTAFGTDHEKAKEIYQTQREYVNLLFQNLHSGWYWIPGFTYLPTQTNVTMARLRSTIDSSLHELITERRKAAERGDTASYGNDLLGIMLAAASNSTDETATEFNLASVFNNAKLFFFAGQDTVATVLTFTLLQLARYPEWQDRARQEVLEEVGETEAYDSTTLNRLKIVGMIVNETMRLFPAVISVSKVATKDMQINELFIPKGLTVEIPIVSYNQDPEIWGDDAHKFKPDRFEHGVSKACKHPRAFLPFSMGPKMCIGKEFALMELKLVVAMVLRRFLSVSPHYKHHPYSSLLTRPKYGMKLIFSSRQASKLEH | |
| CYP767A1 | MYSGRWWELPRDLSDLARRSRRHAAAHLAIGASAAKRNGQPKYDLDLIPGPWTHALPFIGNLLQFLRPDFHRVCLRWADKYGGIVRIKFLWHDGLLVTDPPALAAICGRGEGAVDKAANIYSPINQMCTPHAYPNLLTSLADDRWRAVRKAIALSFAFGNIRKKFPLIRDRTGELLEWLRGVGPLESVDVDQAALRVTLDVIGLSAFGHDYGCTRLQQVPYNHLLRVLPRAFTEVMRRIANPFRSFAPGLVKNGKKGLTSFKDFQRHMQELLGEIKARGPPARGDADIGAQLYRVLEAARPAITDERILSEIGILFVEGFETTGHTISWTLFNIATTPGTQEAVAEELSSLGLLVRPKSEGGRSAARQLELDDLKRLRYLTACVKESMRMYPVVSIMGRTTDKPTRVGPYVVPSGTPVATALFAIHNTIHNWRDPMTFKPERWLGECSLGVLGSFMPFSEGPRSCVGQSLAKLEVMTVLAMLLANFRIELSDEMGGREGVRQRESTHLTLQTRGTRGIRMHLHPRDQE | |
| CYP768A1 | MWDTLRFYYSTHGPLGAWTPAIVLLLNILGIALALAVTKFIGLYFAPSYDLRKIPTPPVGDAILGHVKFLLRPDYHRVILAWTRKYGKIFRLRILTQWTVVITDPAAAAQVLAVVPGRTHNYTLVDEGLGGPGKISMFGTRDEAHWRNVRKATAPAFSMANVPDARALPGFDLLVPRILLLMAEANRQIVDPLWALWYRTPLAPLLSKHVSECRAAVREVRAFHTATAARLLDRPDPPSDNTLLWACLHRLRHHITGARLTPTQLHPEVGMYTTAGFDTTASTLGWCLYAAALHPDQQQKVADELQQACVFGNGAVVEDLVKLPYLTAFVNEAMRLYPTTAVAAERVSPDRPVAVGPFTLPPGVVLWPLVYGIHMSDANWDEPEAFRMERWLEDPRCAFARGERGPGASGAPRRFLPFADGPKNCVGQNFGLVVVRAVLALLLSRYRVALHGDMGLERVAVVTKLSKLRLVMTPRD | |
| CYP769A1 | MSIDARLDRRLNYRCNLRGRVSRRALQDVHLSTRWTKTAPPPGVPLLGHSLTLRAWPSWTWWWFRSGGPRGDQLLLRALLRWSEQYDGAFQLRNGWLVLHPNAVPSSATATSSAQWRLLRRSLLHAFSDSELQLDFEGPGAVVDVNDAALRLSLDVMGLSKLGYDFQVGMAVAVESQGEVLMLRLLGEVAAEWAVRRRRLLGRWAPWISDGAAEGQTRCRILHHFIEQLLLAHGPTGHSIAWALGCLAARRGVQEKLVAELKKEGIFNDPLRLTYDMLSKLPYLDCVVREVLRLYPTMPCPATVRTLKKDVALHGRTLTAASDVWVDVFSMHRSPKWWRDPHHFKPERWTASPPPLAPLCSPEAFMPFSFGSRSCLGQKLAVAQIKAALAMLLCFLVFEPSVAPWGLGLFLRPEGGMQLLVAPRKKNS | |
| CYP76A3 | MVLSESNFLLCLISISIASVFFFLLKKTSRSYKLPPGPSGLPIVGNMFDLGDLPHIKMEGMRNQYGPVMWLKIGAINTLVIQSAQAATAFFKNHDANFLERVVVEVNRVCNYLQGSLALAPYGNYWRMLRRICSMELFVHSRINNSESIRRKSVDKMIQWIETHGKKEQGQGIEITRFVFLASFNMLGNLIMSKELAADPDSTTASEFFDAMMGQVEWSGTPNISDVFPLLRWLDIQGLRRKMKRDMGKGKEILSTFIKERIKEQENGRAKGTDFLDVLLAFEGKGKDEPAKLSEHEINIFILEMFLAGTETSSSTTEWALTELLRNPETMARVKAEIAEVVGPNKKFEESDIDKVPYMQAVVKETFRLHPPLPFLLPRKATQDTKFMGYDVPKGTQIFINAWAIGRDPECWHDPLDFIPERFIGSKIDFKGLNYELIPFGAGRRMCVGVPLGHRMVHFVLGTLLHEFNWELPHNMSSKSIDMTERLGTTVRKLEPLKVIPNKCKLS | |
| CYP76A4 | MVLDWCYFAWFSIFLVPFFFLVLSRKKSCSHRLPPGPPGWPIFGNLFDLGTLPHQTIAGMKLRYGPVVLLRIGSVKTIAILSAKVATEFFKNHDACFADRKIIDTMLVHNYNKSSLVLAPYGTYWRVLRRICTVEMFTNKRINETAHLRQKCIDSMLQWIDKEAKSMKKGSGIEVARFIFLASFNMMGNLMLSRDLVDPESKKASEFFTAMEGLMEWSGQPNISDIFPCLRWLDIQGLRQKAGRDMGKAIEVASTFVKERLKEHKEGEYKKDFLEVLLEFEGSGKDEPAKLSEHQINIFILEMFIAGSETSSSSVEWALAELLCNPEAMTRVKAEINEVVGSNRKFEESDIDNLHYMQAVVKETLRLHPPAPLLVPRRAIQDTSFMGYDISEDTQVFVNAWAIGRDPECWEDPWAFKPERFLNLSSKTTDFKGQNFEFIPFGAGRRMCAGLPLGNRMSHLLLGSLLHAFDWELPSNVTPKSMDMKERMGMTVRKLQPLQVVPIKNEHILY | |
| CYP76AA1 | MAMPACSSWKFLLYSTVFEIILLSFLLIILRDKKKRGKLPPGPPGWPIVGNLFQLGKKPNESLLQLAKKYGPLMSLRLGMKTAIVVSSPAMAREVFKNHDHLFAGRTVIQAAKCASHDKSSLVWSQYGPRWRMLRKICNTELFGVRRLNALQHLRRDQIFQTIRSIYEENYLKRNTVNVGHTAFLTSLNVLGNMIFTQNIFGRDSQAAEELKQTISKVMEISGTPNLADYFPFLQIGDPQGITRAKTLYLKRVYALLDKFVEDRLSSTSTPQNGSSAEKDFLDVLIDCYRNADDGEGAGISRTDITPLIYDLIVAGSETTSTTIEWGLAEVIRNLQAMKRTQAELDDVVGRDRQVEESDIGHLPYLSAVVKEVFRLHPPAPLLLPHRADSCCEIAGFFIPKDAQVIVNVWGMGRDPSTWNDPMEFVPERFTESEVDFKGNNMELIPFGAGRRICPGLPLANRMLHFLLAALLHSFDWSLPDGHNSQQMEMTGKFGLTLQKASPLMAVPSPRLPANLY | |
| CYP76AB1 | MEELTQYLLWSIFFFIAIAMLLRRRSSRNLALPPGPRPLPVLGNLLELGQNPHRSLALLARIHGPVMYLKLGSITQSSSPLQPPQKKSLKQKITPPPPDKSQILSQAVGHHQVSVIWLSPNQSWRYLRTLMKANLFNAKSLNATELLRRRKVRELIAYIKGKNGEAVHVARAAFCTVLNLISTTFLSIDMVDIFQSESAQELKDLMSGIMEEVGRPNVSDFFPFLAPIDLQGCRRRFAAYIKKLSDFFDEVIENRLAGGGGRNKHDILNALLQLSREENSKLSRNTIISFLIDSFAAGSETSSATLEWAMVELLRSPEQMATAREEIATVIGLEREVEESDMSRLPFLQAVLKETLRLHPPGPLLVPHKTEESTEINGYAVPKNSQFLVNVWAIGRDERLWENPDCFMPERFVAGGEIDFRGHHFELLPFGSGRRICPGMPLGVRMVQLMLASLLQSFEWGLPDGMKPEDLDLTEKHGLSTVLAAPLKAIATPTKHN | |
| CYP76AC2 | AAIHTTALTTEWGIAELLKHPHCITRLRQEMEEVLGDKKGQLIVEADIAKLTYLQCVIKEILRLHPVVSLLLPRMSSQECEVGGYTIPAKTLTFVNVWAIGRDEDVWENALEFRPERFESNKDIDVKGHHYELLPFGSGRRICAGLPVALSMVSLTLANLVHCFDLELPHGQTPDSMNMEERKGIAANKAVPTVLVPKPRFSMNFC | |
| CYP76AH1 | MDSFPLLAALFFIAATITFLSFRRRRNLPPGPFPYPIVGNMLQLGANPHQVFAKLSKRYGPLMSIHLGSLYTVIVSSPEMAKEILHRHGQVFSGRTIAQAVHACDHDKISMGFLPVASEWRDMRKICKEQMFSNQSMEASQGLRRQKLQQLLDHVQKCSDSGRAVDIREAAFITTLNLMSATLFSSQATEFDSKATMEFKEIIEGVATIVGVPNFADYFPILRPFDPQGVKRRADVFFGKLLAKIEGYLNERLESKRANPNAPKKDDFLEIVVDIIQANEFKLKTHHFTHLMLDLFVGGSDTNTTSIEWAMSELVMNPDKMARLKAELKSVAGDEKIVDESAMPKLPYLQAVIKEVMRIHPPGPLLLPRKAESDQEVNGYLIPKGTQILINAYAIGRDPSIWTDPETFDPERFLDNKIDFKGQDYELLPFGSGRRVCPGMPLATRILHMATATLVHNFDWKLEDDSTAAADHAGELFGVAVRRAVPLRIIPIVKS | |
| CYP76AH4 | MAKKTSSKGKLPPGPFPLPIVGNMLQLGTQPHETFAKLSKKYGPLMSIHLGSLYTVIVSSPEMAKEIMHKYGQVFSGRTVAQAVHACGHDKISMGFLPVGGEWRDMRKICKEQMFSHQSMEDSQWLRKQKLQQLLEYAQKCSERGRAIDIREAAFITTLNLMSATLFSMQATEFDSKVTMEFKEIIEGVATIVGVPNFADYFPILRPFDPQGVKRRADVYFGRLLAIIEGFLNERIESRRTNPNAPKKDDFLETLVDTLQTNDNKLKTDHLTHLMLDLFVGGSETSTTSIEWTMSELVMNPEKMAKLKAELKSVAGDEKIVDESEIAKLPYLQAVIKEVMRIHPPGPLLLPRKAESDQEVNGYLIPKGTQVLINAWAIGRDPSVWKNPDSFEPERFLEQKIDFKGQDFELLPFGSGRRVCPGLPLASRILHMTAATLVHNFDWKLEDEATAEADHAGELFGLAVRRAVPLRIIPIVKS | |
| CYP76B1 | HEWVLGVGKPKNLPPGPTRLPIIGNLHLLGALPHQSLAKLAKIHGPIMSLQLGQITTLVISSATAAEEVLKKQDLAFSTRNVPDAVRAYNHERHSISFLHVCTEWRTLRRIVSSNIFSNSSLEAKQHLRSKKVEELIAYCRKAALSNENVHIGRAAFRTSLNLLSNTIFSKDLTDPYEDSASGKEFREVITNIMVDSAKTNLVDVFPVLKRIDPQGIKRGMARHFSKVLGIFDQLIEERMRTGRFEQGDVLDVCLKMMQDNPNEFNHTNIKALFLDLFVAGTDTTSITIEWAMTELLRKPHIMSKAKEELEKVIGKGSIVKEDDVLRLPYLSCIVKEVLRLHPPSPLLLPRKVVTQVELSGYTIPAGTLVFVNAWAIGRDPTVWDDSLEFKPQRFLESRLDVRGHDFDLIPFGAGRRICPGIPLATRMVPIMLGSLLNNFDWKIDTKVPYDVLDMTEKNGTTISKAKPLCVVPIPLN | |
| CYP76B4 | MDMLQSSTLSYLVIIFTFSMLLLIKFLIPTNKTNQKNHSKLPPGPSPLPIIGNLLKLGNKPHHSLANLSNIHGPIMTLKLGQVTTIVISSADIAKEVLQTHDTLLSNRTVPDALSVLNHDQYSLSFMRVSPRWRDLRKICNNQLFSNKTLDSSQALRRRKLQDLLDDIKKCSEIDEAVDIGRVAFMTTINLLSNTFFSADFVHSAEEAGEYKEIVVSILKEVGAPNLSDFFPMLTVFDLQGIRRRSVVSVKKVLSIFRRFVGERLKLREGTGSIENDDVLDALLNISLDDGKIEMDKDEIEHLLLNIFVAGTDTTTYTLEWAMAELMHNPEIMSKVQKELEQVVGKGIPIQETDIAKLPYMQAVIKETFRLHPPVPLLLPRKAETDVEIGDYIIPKDAQVLVNAWVIGRDPNKWDNANVFVPERFLDSEIDVKGHHFELIPFGSGRRICPGLPLAIRMLPMMLGSLVNCFDWKLEDGLNIDDLNKEDEYGITLEKSQPVRIVPIKLTIQ | |
| CYP76B6 | MDYLTIILTLLFALTLYEAFSYLSRRTKNLPPGPSPLPFIGSLHLLGDQPHKSLAKLSKKHGPIMSLKLGQITTIVISSSTMAKEVLQKQDLAFSSRSVPNALHAHNQFKFSVVWLPVASRWRSLRKVLNSNIFSGNRLDANQHLRTRKVQELIAYCRKNSQSGEAVDVGRAAFRTSLNLLSNLIFSKDLTDPYSDSAKEFKDLVWNIMVEAGKPNLVDFFPLLEKVDPQGIRHRMTIHFGEVLKLFGGLVNERLEQRRSKGEKNDVLDVLLTTSQESPEEIDRTHIERMCLDLFVAGTDTTSSTLEWAMSEMLKNPDKMKKTQDELAQVIGRGKTIEESDINRLPYLRCVMKETLRIHPPVPFLIPRKVEQSVEVCGYNVPKGSQVLVNAWAIGRDETVWDDALAFKPERFMESELDIRGRDFELIPFGAGRRICPGLPLALRTVPLMLGSLLNSFNWKLEGGMAPKDLDMEEKFGITLQKAHPLRAVPSTL | |
| CYP76C1 | MDIISGQALLLLFCFILSCFLIFTTTRSGRISRGATALPPGPPRLPIIGNIHLVGKHPHRSFAELSKTYGPVMSLKLGSLNTVVIASPEAAREVLRTHDQILSARSPTNAVRSINHQDASLVWLPSSSARWRLLRRLSVTQLLSPQRIEATKALRMNKVKELVSFISESSDREESVDISRVAFITTLNIISNILFSVDLGSYNAKASINGVQDTVISVMDAAGTPDAANYFPFLRFLDLQGNVKTFKVCTERLVRVFRGFIDAKIAEKSSQNNPKDVSKNDFVDNLLDYKGDESELSISDIEHLLLDMFTAGTDTSSSTLEWAMTELLKNPKTMAKAQAEIDCVIGQNGIVEESDISKLPYLQAVVKETFRLHTPVPLLIPRKAESDAEILGFMVLKDTQVLVNVWAIGRDPSVWDNPSQFEPERFLGKDMDVRGRDYELTPFGAGRRICPGMPLAMKTVSLMLASLLYSFDWKLPKGVLSEDLDMDETFGLTLHKTNPLHAVPVKKRANIN | |
| CYP76C2 | MDIIFEQALFPLFCFVLSFFIIFFTTTRPRSSRKVVPSPPGPPRLPIIGNIHLVGRNPHHSFADLSKTYGPIMSLKFGSLNTVVVTSPEAAREVLRTYDQILSSRTPTNSIRSINHDKVSVVWLPPSSSRWRLLRKLSATQLFSPQRIEATKTLRENKVKELVSFMSESSEREEAVDISRATFITALNIISNILFSVDLGNYDSNKSGVFQDTVIGVMEAVGNPDAANFFPFLGFLDLQGNRKTLKACSERLFKVFRGFIDAKLAEKSLRDTNSKDVRERDFVDVLLDLTEGDEAELNTNDIVHLLLDLFGAGTDTNSSTVEWAMAELLRNPETMVKAQAEIDCVIGQKGVVEESDISALPYLQAVVKETFRLHPAAPLLVPRKAESDVEVLGFMVPKDTQVFVNVWAIGRDPNVWENSSRFKPERFLGKDIDLRGRDYELTPFGAGRRICPGLPLAVKTVPLMLASLLYSFDWKLPNGVGSEDLDMDETFGLTLHKTNPLHAVPVKKRGRN | |
| CYP76C4 | MDIISGQALFLLFCFISSCFLISTTARSRRSSGRAATLPPGPPRLPIIGNIHQVGKNPHSSFADLAKIYGPIMSLKFGCLNSVVITSPEAAREVLRTHDQILSGRKSNDSIRCFGHEEVSVIWLPPSSARWRMLRKLSVTLMFSPQRTEATKALRMKKVQELVSFMNESSERKEAVDISRASYTTVLNIISNILFSVDLGSYDSKKSNEFQDTVIGAMEAAGKPDAANYFPFMGFLDLQGNRKAMRGLTERLFRVFRGFMDAKIAEKSLGNYSKDVSNRDFLDSLLILNEGDEAELDNNDIEHLLLDMFTAGTDTSSSTLEWAMAELLRNPKTMVKAQAEMDRVLGQNSVVQESDISGLPYLQAVVKETFRLHPAAPLLVPRKAESDVEVLGFMVPKDTQVLVNVWAIGRDPSVWENPSQFEPERFMGKDIDVKGRDYELTPFGGGRRICPGLPLAVKTVSLMLASLLYSFDWKLPNGVVSEDLDMDETFGITLHRTNTLYAIPVKKQTIN | |
| CYP76D1 | WRSLRRACATKIFSPQQLDSTQFHRKRKVQDLLNYVQKCCEKGEALDFGEVVLATVMNSISETFISMDLFHYCDPSNDDDNKKSREFKEMVFGIMEEVGRPNVVDFFPFLKLFDPQGVRTRMRNHFEKLLAFFYEVMKERMRLRASGESKEYKDVLDSFLDLLNEENSQLCRHDVLHLFTDLFVAGIDTTSTTMEWAMAELLHNPSKLARLRKELEQIHGKFGQIEESDASKLPYLRAVVKEILRLHPSVPFLVPHKSKDDGELGGFMVPKNAQILVNVWSIGRNSSIWDNQIHLNLKDFWRVKLISKGRDFELVPFGAGRRICPGLPLASRSIHYIMASLLHHFNFKLADDLKPDDMDMSHKFGVTLHKAQPLRVVPIKA | |
| CYP76E1 | MDHQTLLLVITFVSATILIFFLRKSNQTQNSTKLPPGPYPLPIIGNILELGKNPHKALTKLSKIYGPIMTLKLGSITTIVISSPQVAKQVLHDNSQIFSNRTVPHAITAVDHDKFSVGWVPTLNLWKKLRKNCATKVFSTKMLDSTKILRQQKLQELLDYVNEKSHNGEVFDIGETVFINVLNSISNTLFSMDLAHSTPDEKSQEFKTIIWGIMEEAGKPNISDFFPILRPLDPQGLYARMTNHMKKLCEIFDGIIEERICLKDSKGDYEVCNDVLDSLLNINIGEATSELSRNEMVHLFLDLFVAGIDTTSSMIEWIIAELLRNPDKLTKVRKELCQTIGKGETIEESHISKLPFLQAVVKETFRLHPPIPLLLPHKCDELVNILDFNVPKNAQVLVNVWAMGRDPAIWDNPNTFVPERFMECDINYKGNNFELIPFGAGKRICPGLPLAHRTMHLMVASLLHNFEWNLADGLIPEHLNMDEQFGLTLKRVQPLRVEAISSA | |
| CYP76F1 | NSARVMTLKLGQVTTVVISSADMAKEVLLTHDLITSNRTVPDALSVLNHDQYSLSFMRVSPRWRDLRKICNYQLFSNKTLDSSQALRRRKLQDLLNDIERCSKVGEAVDVGKAAFKTTVNLLSNTFFSVDFVHSAKEAGEYKEIIVSILKEVGVPNVSDFFPMLKFLDLQGIRKRSIVSVKKVLSIFKRFVGERVKMREGTGSIGNDDVLDALLNMSSDGGKIEMDKDEIEHLLLNIFVAGTDTTTYTLEWAMAELIHNPEMMSKLKEELEKTVGKGIPVEETDIAKLPYMQAVIKETFRLHPPVPLLLPRRAEIDVKIGDYVIPKDAQILINAWVVGRDPTKWENPNVFIPERFLDSEIDIKGHHFELIPFGSGRRTCPGLPLAIRMLPLMLGSLVNCFDWKLEDGLNVEDFNKEDEFGITLEKSQPVRIVPTKLY | |
| CYP76F38v1 | MDFLSFILFVLFAWALVRALPTLSRGSKAAGGRLPPGPVPLPVVGNLLKLGSKPHKSLAELAKSYGPIMCLKLGHIITIVISTPTVAKEVLQKQDVAFCNRTIPDAVRAHRHDLHSMVWLPVSTRWRTLRKISNSHIFSSQRLDENHHLRRRKLDELLARVAESSLVGAVVDIGAVAFLTSLNLLSNTVFSKDLVEPGLGAVQEMEEVVWGITEEAGRPNLVDYFPVLRRLDPQGTRRRMMGYFGKMFEVFGDIIDERLELRKQQSDGDSPAATTNDVLDVLLNIIEDAEIEEKPNRTDVEHFIVDLFVAGSDTTSSTVEWAMTELLRKPETLERARSELHETIGPKNLVQEADMPRLPYLQAVVKETFRLHPPVPLLLPRTAEKDAELCGFTVPAGAQIMVNAWAIGRDPGTWEDPESFLPERFLGSDVDVKGRSFELIPFGGGRRICPGLPLAIRMVHLMLGSLIHGFRWKVFDDGMGSPETAMDMDEKFGITLQKAKSLCAVPIRG | |
| CYP76F39v1 | MDFLSCILFVLFAWALVRALPTLSRGSKAASGRLPPGPVPWPVVGNLLKLGNKPHKSLAELAKSYGPIMCLKLGHMTTIVISTPTVAKEVLQKQDVAFSNRTTPDAVRAHGHDLYSMAWLPVSTRWRTLRKISNSHIFTSQRLDENHHLRRRKLDELLARVAESSLVGAVVDMGAVAFLTSLNLLSNTVFSKDLVEPGLGAVQETKEVVWGMMEEAGRPNLVDYFPVLRRLDPQGIRRRMTGYFGKMLEVFGDIIDERLEWRKQQSDGDSPAGTTNDVLDVLLNIIEDAEIEEKPNRTDVEHFLLDLFAAGSDTTSSTVEWAMTELLRKPETLERARSELHETIGPENLVQEADLPRLPYLQAVVKETFRLHPPVPLLLPRTAEKDAELCGFTVPAGAQIMVNAWAIGRDPGTWEDPESFLPERFLGSDVDVKGRSFELIPFGGGRRICPGLPLAIRMVHLMLGSLIHGFRWKVDDDGMGSPETAMDMDEKFGITLQKAKPLCAVPIRG | |
| CYP76F40 | MDFLSCILSVLFAWALVRALRKLSRGSKAASGRLPPGPVPWPVVGNLLKLGNKPHKSLAELAKSYGPIMCLKLGHMTTIVISTPTVAKEVLQKQDVAFSNRTTPDAVRAHGHDLYSMAWLPVSTRWRTLRKISNSHIFTSQRLDENHHLRRRKLDELLARVAESSLVGAVVDMGAVAFLTSLNLLSNTVFSKDLVEPGLGAVQETKEVVWGMMEEAGRPNLVDYFPVLRRLDPQGIRRRMTGYFGKMLEVFGDIIDERLEWRKQQSDGDSPAGTTNDVLDVLLNIIEDAEIEEKPNRTDVEHFIVDLFVAGSDTTSSTVEWAMTELLRKPETLERARSELHETIGPKNLVQEADMPRLPYLQAVVKETFRLHPPVPLLLPRTAEKDAELCGFTVPAGAQIMVNAWAIGRDPGTWEDPESFLPERFLGSDVDVKGRSFELIPFGGGRRICPGLPLAIRMVHLMLGSLIHGFRWKVADDGMGSPETAMDMDEKFGITLQKAKSLCAVPIRG | |
| CYP76F41 | MDFLSCILFVLFAWALVHALRTLSRGSKAASGRLPPGPVPWPVVGNLLKLGNKPHKSLAELAKSYGPIMCLKLGHMTTIVISTPTVAKEVLQKQDVAFSNRTTPDAVRAHGHDLYSMAWLPVSTRWRTLRKISNSHIFTSQRLDENHHLRRRKLDELLARVAESSLVGAVVDMGAVAFLTSLNLLSNTVFSKDLVEPGLGAVQEMEEVVWGITEEAGRPNLVDYFPVLRRLDPQGTRRRMMGYFGKMFEVFGDIIDERLELRKQQSDGDSPAATTNDVLDVLLNIIEDAEIEEKPNRTDVEHFIVDLFVAGSDTTSSTVEWAMTELLRKPETLERARSELHETIGPKNLVQEADMPRLPYLQAVVKETFRLHPPVPLLLPRTAEKDAELCGFTVPAGAQIMVNAWAIGRDPGTWEDPESFLPERFLGSDVDVKGRSFELIPFGGGRRICPGLPLAIRMVHLMLGSLIHGFRWKVADDGMGSPETAMDMDEKFGITLQKAKSLCAVPIRG | |
| CYP76F42 | MDFLSCILSVLFAWALVRALRTLSRGSKAAGGRLPPGPVPLPVVGNLLKLGNKPHKSLAALAKSYDPIMCLKLGHMTTIVISSPTVAKEVLQKQDVAFCNRTTPDAVRAHGHDLYSMAWLPVSTRWRTLRKISNSHIFTSQRLDENHHLRRQKLDELLARVAESSLVGAAVDIGAVAFVTSLNLLSNTVFSKDLVEPGLGAVQEMKEVVWGIMEEAGRPNLVDYFPVLRRLDPQGIRRRMMGYFGKMFEVFGDIIDERLELRKQQSDGDSPAATTNDVLDVLLNIIEDAEIEEKPNRTDVEHFIVDLFVAGSDTTSSTVEWAMTELLRKPETLERARSELHETIGPKNLVQEADMPRLPYLQAVVKETFRLHPPVPLLLPRTAEKDAELCGFTVPAGAQIMVNAWAIGRDPGTWEDPESFLPERFLGSDVDVKGRSFELIPFGGGRRICPGLPLAIRMVHLMLGSLIHGFRWKVADDGMGSPETAMDMDEKFGITLQKAKSLCAVPIRG | |
| CYP76G1 | MINQLTKNELIGLFTSIAVLIYVTCLFYTKRCRTRLPPGPNPWPVIGNIFQLAGLPPHDSLTKLSRRHGPIMTLRIGSMLTVVISSSEVAREIFKKHDAALAGRKIYEAMKGGKSSDGSLITAQYGAYWRMLRRLCTTQFFVTRRLDAMSDVRSRCVDQMLRFVEEGGQNGTKTIDVGRYFFLMAFNLIGNLMFSRDLLDPDSKRGSEFFYHTGKVMEFAGKPNVADFFPLLRFLDPQGIRRKTQFHVEKAFEIAGEFIRERTEVREREKSDEKTKDYLDVLLEFRGGDGVDEEPSSFSSRDINVIVFEMFTAGTDTTTSTLEWALAELLHNPRTLTKLQTELRTYFKSSNQKLQEEDLPNLPYLSAVIMETLRLHPPLPFLVPHKAMSTCHIFDQYTIPKETQVLVNVWAIGRDPKTWIDPIMFKPERFISDPDARDFKGQDYEFLPFGSGRRMCPALPLASRVLPLAIGSMVRSFDWALENGLNAEEMDMGERIGITLKKAVPLEAIPIPYRGT | |
| CYP76H6 | MASALFLWLSWLVLSLLSIYLLDLLAHSRRRLPPGPRPLPLIGSLHLLGDQPHRSLAGLAKTYGPLMSLRLGAVTTVVVSSPDVAREFLQKHDAVFATRSAPDAAGDHTRNSVPWLPPGPRWRELRKIMATELFATHRLDALHELRQEKVSELVDHVARLARDGAAVDVGRVAFTTSLNLLSRTIFSRDLTSLDDRGASKEFQQVVTDIMGAAGSPNLSDFFPALAAADLQGWRRRLAGLFERLHRVFDAEIEHRRRVAGEEHGKVKDDFLRVLLRLAARDDDTAGLDDDTLRSVFTLLKDLFAAGSDTSSSTVEWAMAELLRNPLPMAKACDELQRVIGSTRRIEESDIGRLPYLQAVIKETFRLHPPVPFLLPRQATTTIQILGYTIPKGAKVFINVWAMGRDKDIWPEAEKFMPERFLERATDFKGADFELIPFGAGRRICPGLPLAVRMVHVVLASLLINFKWRLPIKVERDGVNMTEKFGVTLAKAIPLCAMATST | |
| CYP76K1 | MELTTISPVFLISLLGVPLLYLLWSKASKSPSGAPAAPPPPPGPTPFPVIGNIPDLLRGGELHRALTGLAASYGPVMSLRLGMASTVVLSSPDVAHEALHKKDGAISSRWVPDNANVLGHQDVSMAWLPSSSPLWKHMRTLASTLLFTSRRLGASRGIRERKARELVDYLGARSGRPVRVGLAVFGSVLNFMSNVFFSEDVVELGSETGQEFQQLIADSVAETAKPNISDFFPFLSALDLSRRRRAAAKNLKKFYDFFDDVIDRRLSSGEKPGDLLDSLLELHAKSQLERPLIRALMDLFIAGSHTTTTTVEWAMAELLRNPSKMAKARAELGEAFGRGAVEEGELARLPYLNAVIKETLRLHPPAPLLLPHRVSSDSEPAGGVTLGGYSVPSGARVLINAWAIGRDPAAWSPEPDAFSPERFLGREADYWGRTLEFIPFGSGRRACPGIPLAVAVVPMVVAAMVHSLEWRLPEGMAPGDVDVGDRFGAVLELATPLWAVPVKV | |
| CYP76L1 | MEASTILWLLYVSLASCLLYKVFVSTKNGHPKIAARRPPGPTPVLLLGNVFDLRGELHLALARLAEEHGPVMSLKLGTATAVVASSAAAARDALQRYDHVLAARAVCDAARALGTHERSIVWLPGSSALWKRLRAVCTNHLFSARGLDATRAVREAKVRELVEHLRGHAAGAGEEEAAAVDVGRVVFSAVINLVSNVLFSEDVADLSSDRAQELEMLVRDTVEEATKPNLSDLFPVLAALDLQGRRRRTAVHIRKFHDFFDEIISRRQNAGGEGERKEDFLDVLLQLHSADQLSLDTIKTFLGDLFTAGTDTNSITVEWAMAELLRHPAAMSRARAELRDALGAKPHPDESDIGRLPYLSAVVMETMRLHPPSPLLMPHEAVADGAAVGGYAVPRGTKVIVNVWSIMRDPASWPRPEEFEPERFVAAGGSFRGGEMLEFMPFGAGRRACPGTPMATRVVTLVLASLLHAFEWRLPGGMRPCDVDVRGRFGTSLNMVTPLKAVPVPVPARP | |
| CYP76M15 | MATPELWWYWWLWVTTMLAVVVSTVVCYLTNQHRRWGGWGSSSGRRRPPGPRPLPLIGNLLDLRRAPGSLHHTLARLARAHGAPVMRLDLGLVPAVVVSSRDAAREAFAAHDRRIAARPVPDSKRALGFCDRSMLSLPSSAPLWRTLRGVMAAHVLSPRSLAASRAARERKVADLIGYLRARAGTVVDLKEAVYGGVANLVSTAMFSIDVVDVGAAESSSSSSAAAHGLQELLEELMQCMAQPNVSDFFPFLSALDLQGCRRRVAVQLGQVLQVLDDITDRRLASSSSSSTSSKGGDRRGDFLDILLDLQSTGKITRDNVTLTLFDIFAAGSDTMALTVVWAMAELLRNPGVMARLRAEVRDALGGRDAVEEADAAGLPFLQAVVREAMRLHPAAPVLLPHKAVEDGVQIGGYAVPRGCTVIFNSWAIMRDPAAWERPDEFLPERFLARDLDFRGKQLEFVPFGSGRRLCPGVPMAERVVPLVLASLVHAFQWQLPAGMSADQVDVSDKFTTTSVLAFPPIKAVPLL | |
| CYP76M6 | MEKLKSELWMTAVATCMSLLLYLTILRRRHASGGRSLALPPGPTPLPLIGNLLCLGGIFHQTLAKLARVHGPVMTLKLGLTTAVVVSSAEAAREAYTKHDQRLAARPVPDAFRANGFSERSIVFSPSSDPQWKNLRGIHATHIFSPRALAALRGIRERKVRDIVGYIRTVAGEEMCVREVVHNGVLNLISTSFFSMDMADVRSESARGLRGLIEDIIATVAGPNVSDFFPFLRQLDLQGLRRQTGSHLGIVFGLLDDIIDRRMAETRDHPDKQRHGDFLDALISLASAGKIPRYHITYLLFDVFAAGADTMTTTVEWAMAELLRNPRVMAKVRAEVTDALGGRESFDEGDAASLTYLQCVFKEAMRLHPVGSILVPHLAVQDGVEIGGYAVPKGTTVIFNAWAIMRDPAAWESPDQFLPERFLHKEESSSPPLELRGKDYEYIPFGSGRRLCPGLPLAERAVPFILASLLHAFEWRLPDGMSPDDMDMTEKFATANVLATPLKAVPVASHTS | |
| CYP76M7 | MENSQVWLLWGALSVAVLFYLSTLRRRYAGGKPLPPGPTPLPLIGNLHLAGGTFHHKLRDLARVHGPVMTLKLGLATNVVISSREAAIEAYTKYDRHLAARATPDTFRACGFADRSMVFIPSSDPQWKALRGIQGSHVFTPRGLAAVRPIRERKVGDLIAYLRAHAGEEVLLGQAMYTGLLNLVSFSYFSIDIVDMGSQMARDLREVVDDIISVVGKPNISDFYPFLRPLDLQGLRRWTTKRFNRVFSIMGDIIDRRLAHIRDGKPRHDDFLDSLLELMATGKMERVNVVNMLFEAFVAGVDTMALTLEWVMAELLHNPAIMARVRAELSDVLGGKEAVEEADAARLPYLQAVLKEAMRLHPVGALLLPHFAAEDGVEIGGYAVPRGSTVLFNAWAIMRDPAAWERPDEFVPERFLGRSPPLDFRGKDVEFMPFGSGRRLCPGLPLAERVVPFILASMLHTFEWKLPGGMTAEDVDVSEKFKSANVLAVPLKAVPVLIK | |
| CYP76M8 | MENSQMWLLWGALSVALFFYFSTLRRRYAGGKPLPPGPTPLPLIGNLHLVGGGTFHHKLRDLARVHGPVMTLKLGLATNVVISSREAAIEAYTKYDRHLAARATPDTFRACGFADRSMVFIPSSDPQWKALRGIHASHVFTPRVLAAVRPIRERKVGDLIAYLRAHAGEEVLVGHAMYTGILNMVSFSYFSVDIVDMGSQMARELREVVDDIILVVGKPNVSDFYPFLRPLDLQGLRRWTTKRFNRVFSIMGDIIDRRLAHIRDNKPRHDDFLDSILELMAAGKIDRVNVLNMLFEAFVAGADTMALTLEWVMAELLKNPSVMAKARAELRDVLGDKEIVEEADAARLPYLQAVLKEAMRLHPVGALLLPHFAMEDGVEVGGYAVPKGSTVLFNAWAIMRDAAAWERPDEFVPERFVERTPQLDFRGKDVEFMPFGSGRRLCPGLPLAERVVPFILASMLHTFEWELPGGMTAEELDVSEKFKTANVLAVPLKAVPVLIK | |
| CYP76N1 | MAASLAWLLVAIVLASLYLAMHHRVAAARRRRLPPGPTPLPLVGNLLSVSRSGPHRSLARLAERYCPLMRVRLGVVDYVVASSPAVAGDIHHHSHNAHLASRPLFDVWRGAEHHRNSVIVLPLHGVWRAQRRLATEEVMSPRRLDALAPTRREKVRELMRCVARRAARGEPVEVGLEAFEAFLGILSCTAFSADLVDPDLRDAVQEATKLAATPNASDFFPAMAAADLQGLRRRMGKLVARAYGIIDELLARRKGGREAGEPRKDDMLDVALDNEDEWKNNNPVIDRNNIKGLIADLFVAGTDSGSTAIEWAIVELLQNPQSMQKVKDEFRRVLGTRTEIEESDISQLPYLQAVLKETLRLHPSVPMTYYKAEATVEVQGYIIPKGTNIILNIWAIHRKPDVWADPDRFMPERFMETDTNFFGKHPEFIPFGGGRRICLGLPLAYRMVHMVLASLLFHFDWKLPEGAEKDGVDMREKYGMVLHKETPLKALAIETYNR | |
| CYP76P1 | MAIFIGCICSLALLLLCSHVFQLLSDARRRLPPGPRPLPVIGNLLDVAGELPHRSLACVAERYGPLVTLRLGTMLAVVASSPATARDVLHRHGASITDRGTPDAWSTDGHDGNSIFAFPTRHHRWRALRRLGAEQLFSPRRVEEQRPLRRDAVRGLLRHVAELAAASGGGGAAVVDVGRAAFAAMASLLFGALFSAGIDAATSCRFRDAAREFALLTMTPNVSEFFPVVAMADLQGLRRRTARHITWMYQLIDGHVERRMRGRETAGGCGAAHGEKEKDLLDVMLDMSEKEEQNDDSSLTMNDLLMAGSETSSAVIEWAMAELLQNPQTMTKLQEELKKVIGSKTCIDEEDIDQLPYLQAVIKETHRLHPAIPLLMYKAAVPVEIQGYKIPKETTVIVNTWAIHQNSEVWIEPDKFIPERFLQKEISLSSGSTNMELIPFSAGRRFCLGYPVANRMLHVMLASLVHQFQWTLPEVVKKNGGVDMAEKFGITLSMATPLHAIAKNIV | |
| CYP76Q1 | MAFFLVACLPWVCFILLSLYVFQLFADARRRLPPGPWPPKPLIGDLLALGKGDQQHRSLARLADRYGPVMSLRLGTVLTVVVSTPDAMREIFHKNKDNLAGRPTADAFNAMGHSANSLLGLEHPGVRWRAIRRFSTAELLAPRRLAALQPLCRDKVRGLVRGVSELAARGEPVHVRRVALDMALSLILSAIYSVDLDPESTAVFRSVVEEAMLLIGTANLSDLFPAIAALDLQGVRRRVAELFTITYRQYDEQVARRRPERDAGEAGKNDLLNVVLDMEREWQQKGSVLSHDAMRVLFTDLYGAGASTTSVLIEWAIADLLQNPESMRKIKEEITNVIGTNAQIQESDIARLPYLQAVVKETLRLRAVAPLVPRRAEATIEVQGFTIPKGTNVILNLWAINRDARAWNDPDKFMPERFIGNDINYLGQNFQFVPFGVGRRICLGLPLAQKVMYLVLGTLVHQFEWTLPEELKDTGIDMTEKCGMVLCLANPLKVMAKKM | |
| CYP76S1 | MDVIFPLLVAFITWAIASSLTFRRFGRLPPGPFPVPVIGNIHQLGKHPNQSLAKLSKIYGPLMSLKLGTQTAIVASSSTVVREILQKHDQVFSSRTIPSALHAHDHHKFSMALLPASSRWRHLRKITKEQMFSVQRLDESQGLRQDKLKELRDYLHSCCVTGQAVNIGEAAFTTTLNLMSCTLFSVNFASFDSKFSDELKRDICAFVQVIAAPNLADFSPVLRHVDPQGLLKRTKTYMQKVFDSFEDIITKRLQERGTSQQDSLRRHDLLEALLDEMEKNDSAFTINDMKHLILDLFIAGADSTSSTTEWGMAELLHNPEKMEKAKAELNEVIGQKNLVEESDISRLPYLQAVVKEVFRLHPPGPLLVPHKADADVEIDGYVVPKNANVLVNVWALGRDSSSWADPEAFMPERFLDNEIDVKGQHFELIPFGAGRRMCPGLPLSYRMLH | |
| CYP76T1 | MEYLFYLLLISFCWACLHVLNASVLLRRKSGCTVLPPAPRQLPIIGNILALGDKPHRTLAKLSQTYGPLMTLKLGRITTIVISSPNIAKEALQKHDQALSSRTVPDALHVQYYNYHKNSMIWLPASTQWKFLRKLTATQMFTSQRLDASRALRGKKVQELLEYVHEKCNNGHAVDVGRSVFTTVLNLISNTFFSLDVTNYNSDLSQEFSNLVVGFLEQIGKPNIADYFPILRLVDPQGIRRKTNNYLKRLTQIFDSIINERTRLRSSSVASKASHDVLDALLILAKENNTELSSTDIQVLLIDFFIAGTDTTSSTVEWAMTELLLNPDKMVKAKNELQQVEGPVQESDISKCPYLQAIVKETFRLHPPSPFLPRKAVSEVEMQGFTVPKNAQVLITIWAIGRDPAIWPEPNSFKPERFLECQADVKGRDFELIPFGAGRRICPGLPLGHKMVHLTLASLIHSFDWKIADDLTPEDIDMSETFGFTLHKSEPLRAIPMKT | |
| CYP76U1 | MAFFLPLAFSLFLAVISAYVLQLLADARRRLPPGPWPLPLIGNLHQLDHLPHRSLARLAARHGPLMSLRLGTVRAVVASSPEMAREVLQRHNADIAARSFGDSMRAGGHCENSVVCLPPRRRWRALRRLSTVGLFSPRRLDAMRALLEEKVAELVRRVSGHAARGEAVDVGHAAHVAALGVLSRTMFSVDLDPEAAREVSDIVDEASVLGTGPNVSDFFPAIAPADLQGVRRRMARLVKRMYAIIDEQIERRMHGRTAGEPRKNDLLDVMLEEGESKEDSNEINRDAIRGLDLFTGGETTSHTMECAMAELLQCPNSMRRVHELKSVIGSKQQMDEHDITKLPYLPPYEAEATIEIQGYTIPKGAKVLINLWAINRCANTWTEPDKFMPERFYDSDITFMGRDFQLIPFGAGKRICLGLPLAHRMVHLMLGSLLHRFTWTLPAEAGKNGVDMRERFGLTLSFVAPLYVIAQEIQ | |
| CYP76V1 | MAFFHLCISSLLLVFIISYIFQPLLDARRRFPPGPHRLPVISNLHNIGKNPHHAFARLADRYGPLMSIRLGGVRAVVAMSADAAREILQRNNADITGRGGMDSWHACGHHANSSIALWPRWKWCAMRMLCTEELLGVTHAMREEVARELAHRVSDGSAGGMPVSVAREAFAAVAGVLWWSMFSEDMDAATTRQLRDVIEEAVVVAGAPNLSDYFPVIAAADVMGVRRRMDNLVGWVYGIIDVQIDRRRRRRIVCEPRKNDLLDVAFDMEGEVESEGWVMNQDTMRGMAIYQDLLVAGSGSTSSTIEWAMAELLQNPKSMIQLPEELKGLMGTKTHVAESDISQLPYLQAVIKETLRLHPTVPIAFNKAEATVEIQGYKIPQGTTVYVNIWAICRRAKIWDDLDKFMPYRFLGRDINFLGTNFEFIPFGAGRRICLGMPLAEGMLHLMLASLLHRFEWTIPDEVKGDDLDMAEEFGLVLSMAKPLRAVAKET | |
| CYP76X1 | MDYVGSGMLLLLTCIVACFIGSLYARSRKSNYRLPPGPSIFTIMSHVFELYYKPQQTLAKFAKFYGPVMLIKLCTETTVIISSSDMAKEILHTNDSLFTDRSVPDNTTTHNHNNFSLVFLPFSPLWQHLRKICHNNLFSNKTLDGSQELRRMKLKDLLNDMHKSSLKGETVDIGRAAFKACINFLSYTFVSQDFVESLDDEYKDIVSTLLSAVGTPNIADHFPILKILDPQGIKRHTTKYVAKVFHALDIIIDQRMKLRKSEDYVSKNDMLDSLLDISKEDSQKMDKKQIKHLLLDLLVAGTETSAYGLERAMTRLVHDPKAMSKARKELEETIGLGNPIEESDIDRLPYLNAVIKESLRLHPPAPMLLPRKARVDVEISGYTIPKGAQVLINEWAIGRTDIWDDADSFSPERFLGSEIDVKGRHFKLTPFGSGRRICPGSPLAVRMLHLMLGSLINSFDWKLENNMEAKDMNLDKPLRAIPVALNKVY | |
| CYP76Y1 | MELNSFLLLCMPLVLCLFFLQFLRPSSHATKLPPGPTGLPILGSLLQIGKLPHHSLARLAKIHGPLITLRLGSITTVVASSPQTAKLILQTHGQNFLDRPVPEAIDSPQGTIAWTPVDHVWRSRRRVCNNHLFTSQSLDSLQHLRYKKVEQLLQHIRKHCVSGTPVDIGLLASATNLNVLSNAIFSVDLVDPGFESAQDFRDLVWGIMEGAGKFNISDYFPMFRRFDLLGVKRDTFSSYRRFYEIVGDIIKSRIKCRASNPVTRNDDFLDVILDQCQEDGSLFDSENIQVLIVELFYAGSDTSTITTEWAMTEFLRNPGVMQKVRQELSEVIGAGQMVRESDMDRLPYFQAVVKETLRLHPAGPLLLPFKAKNDVELSGFTIPSNSHVLVNMWAIARDPSYWEDPLSFLPERFLGSKIDYRGQDFEYIPFGAGRRICPGMPLAVRMVQLVLASIIHSFNWKLPEGTTPLTIDMQEHCGATLKKAIPLSAIPFIEEN | |
| CYP76Z1 | MMNSMAIESVYDPLVFGVVLSFIFLLLLHWKKKNSRLPPGPPGWPIIGNVLQLGDKPHESLFGLAQKYGPLMSLRLGCKLTMVVSSPSMAKEVLKDNDQTFSSRSINMAARTFAYQGTSLVWSPYGPHWRFLRRICNAELFSPKRLDALQHLRREEVNRTIRSIFEVSMEGQSVNIGEIAFLNSLSLVGMMVCSRNLFNPGSKEVAEFKEMVWEVLKLTGTPNLSDLFPFLERFDLQGLKKGMKTLARRFDSLFDSIIEERLGEDGAGVHHEGKDFLEIMLGLRKAGTQFTLENIKAVLMDMFIAGTDTTSVTVEWAMAELLGKPAVIRKAQAELDEIVGQAKRMEESDIAKLPYLQAIVKEALRLHPAAPLIIPRRSDNSCEIGGYVVPENTQVFVNVWGIGRDPSVWKEPLEFNPERFLECNTDYRGQDFELIPFGAGRRICIGLPLAHRMVHLVLGSLLHAFNWSIPGATKDDDFVIDMSEVFGLTLQKKVPLIAVPTPRQPINLLY | |
| CYP770A1 | LLVSEGQQWRLMHALATPAFKAELLERGAFAAALRGVMEEWHRRAVALLPLWRLQAAGVALTLVGMGHENVSATAAWALLLLAAHPEQQQALYRELRQGCGFPTSRFIQSHPSRTAALLRLPYLDAVLRETLRLYPPVPMLSRQLMQDTTIGGVMLPKDVELVVSPYVLHRLPRLWGPHAACFQPERFMPPPPRPPPAAGGGCTEPAAAGPYLPFGAGPRACPGASFGSAEVKLLVAHVVMRYSLELLQPPPPSPRQLFVSLRPGPGVRVCFVPRHQQQVE | |
| CYP771A1 | MRAGYVRAKAVSCLWPKCRQLPTRVRFIRHVRWKPKRSPPPLLRPAHVRYLGKRKLLLREPDDVAAVLARPGEDAFRKHPRQQRVSAFLGAGLATQPDRQRHAAQRDALAPAFRPDAVRQLDAVMAAAAERLAEALMAAAEAEAEEAEAVAAASGSSSGAAGAAAGAGAGAAAGELQVEMQDLLKRHSLDLLGLAALRSDMGALRRSPVMAAAAAAAAAAGGGYAAVGADVDVVTLMTEIEAASLWLLMALPVPNELLPGYGTYEANVRRLDELLVTMLLGGTDTSALTVAFAAWHLAAEPQLQAELRREVLGVLGGRALGELRAEDVKAMPLLAAVVNETLRLHPPLAEITRVATQPNAFLPFGVGSRSCIGRHFGLLSTQLTLAALVARFEVLPPAPPAPTALDWSQSIVITSRSGVWLRLRPIRQ | |
| CYP772A1 | MTMVQDSMIQALDALPVPAVAASVVAVIITTVLLAVFRSRPGDAPSVPGLPLLGSAMALGRHGVAFINKCRQQFGNSFSLSLAGVKMTFLFDPQHIDYFFGAPDSKITFRPAVEQFTQRVFGLTSRLFFPLHFKMLTELRHLLVPASIAAHMQALGGRVLALLPLYVHHPQVDLYSLCRGLVFHCAGGEGGHQRPPEGVHRLARDFFAFEDGFELAASPVPHAFQPEFTAARQRLLALLAAADARGLFAGTLAGQLLERTAGLPPALRPNLLLAVLWASQANTVPATFWATGFLLLPENAHHRAAVLAELQAELKGAVSAAGSPGGSAAYSNEELVAAAARVASSRRSAVSRCVAEALRLRVQSIDVRIAADHLELPLAGVKGGGGDVLRLPRGRLLAICPFVSHHDTQLYGGAAAAAAAAAAGCPAVTGAAAAGDVSSPWAFNPDRPELKLGDGTAVVSSVAGLAFGGGPYRCPGRFFAEQELGLLVQLLLWTYDIQLSYTPQLRQVAGGSWLYGVLSGLVGARALAWGCGWFDGVDGPLEDFRHSGDPGGLLPPCDLKRLVGVKVPRRPLWVQLGVPHWQARRLGLVGVAPAATSRRWADIGLG | |
| CYP773A1v1 | MDLIWNVVTAGVSLIATICVWQLARDFLWRPRRLLQAFKQQGVLGPVPRLFLGNLDQVRELMAVEVVKSSTGEIRDDNHGGVVAKVLPYYAAWSRSYGETFLVWWGSQPRLMISDPELMKEVLCDKSGSLDRDPGQHAARDLFGDGIALLTMNERWSQKRKMVSLAFHNEKLKLMIDAMVACVEENLKQWKTTEGPVDVASKLRDITQDVLCRTAFGTSYAAGKEVFEMQIEQQYIHLEWQGQVHLPGFRFLPTSANRRRWTLKQQIDSKLRKIVVNRLKESSVSGSYGKDLLGLMLAAKDGVLDFNNGKKLDIQVTMQDVIDECKTFFFTGQETSAALLAWTMLLLALNPDWQTRLRQEVCQVCGQVSAPNTLEMLGNLKSMTMVINEALRMYPPVPLLNRYTHNKVKLKELVIPKGTLLLVPLIVINYNEKFWGVDAKSFNPDRFVSQQQRPFLPFSVGPRTCVGQSFAMIETKIILAMILRKFKFELSETYVHSPFQVLTLQPKFGMPMNLLANQ | |
| CYP774A1v1 | MEDFLWKILCSCGVFLVVWLLVPAARSLWHAWRFRCFYAKQGIPGPPFRLIVGNIPEIRKLFNSVPKFETSFHAATKFRVIPDLATFQQTYGRISVHELGSTTRILVADTELVKQVLMSRSSSYIKADLSRQILRAVVGRGVVVTDGDFWRQQRKILNPAFKLAYLKGLMRHMSGAGEDLARKWSSRETTRIDAHREMAALTLDVITRASFGATIGGTNTGYAAFECLDRLLSTGLLYMNSYKRLIPGYSFLPTRENLHLRRSEQYVNTLLRDIIRNRWAEKTRNPDENGKPVYDLLDMMLEAVENKSPTMTMDQLLDECKTIFFAGHSTTALTLTWSLIMLSVHQEWQQRARDEIFAAHKRCGGRDLSAEDLSSLEVVGWIIHEVLRLFPPVSTVTRQCHQAHEIGEFSILPGTLVLCPLALLLQSKEDWGDDVSEFNPERFINKKTKDISEFMAFGAGPRMCLGMNFALIEARLLLSLLLAKFSFTLAEDYVHAPGSPVSMKPVYGAPLLVKKL | |
| CYP775A1v1 | MQGLEGPRPKFLVGNMDEITRMKETAFHQPMEIGDHNLLQRICPYYLEWSKLYGRTFVFWWGTEPRITVTRPDMIKEILYSKAAHFGKSALQRKGGAVLLGNGLIMANGSDWAHRRAIVCRAFKMDKIKEMVPSMLESTKNLIRRWDAHLELNGGAPCEVDAYRDLAVVTADIIATTAFGSSYSDGIKLFHTLTSIQKLFVQSNKYLWLPGSRLLPTRTNRKIRKLQREMQALLQDLIKARLSSPSLGTDLLALMLSAVEEDPGNKVQSSKFKFTIQQLIEECQTFFFVGHETTLMLVTWAMMLLCLHPEWQDLARKEARQVLQESNRVVNADTLAKLKTVGMIINETLRLYPPAPNLVRAALQDTCVGDLYVPKGTTFWIPILALHQDKHLWGEDAHEFRPQRFSQGVSRACKTYDFLPFSSGPRICVGQSFAIMEAKLILAMILQHYHLGLSPRYKHSPVSSVTLKPGLGMQLMIKRCD | |
| CYP775B1v1 | MLLLAAPMLVFLASLLVILVWSASVAFRYVHLRKSLLEQGLDGPPPKFLIGNMHQVSEMRELATSKDMRVGDHDLLPRICPHFTYWSAIYGKRFLFWWGMEPRITVVEPEMIKEILSTKAEHFGKSLLLKKGGVLLLGNGMVYANGESWAHRRRIVGPAFHAEMLKKMVPEMVASTSQMLGQWSQIIDNKSSRRSGDGSSAEIDINYYLSMATADVIARTAFSSTSSHEKGKRVFQLLTCLQKVFAQSNRFLWLPCNRMLPTAANRRASRIKRDMERALRELVLERRAGRQKHGYGSDFLGLMLSESERDKSEAAAAAQQFDTPELVEECKTIFFTGHETTSALLTWTLMLLALNPEWQQRGRAEVMEHLPSKSSVPDADVLPKLKILGMILNEVLRLYPPAPALVRESLVDLSIQDVKYPRGTTFWIPIVALHHSKDVWGDDALHFNPARFADGVAAACKLQHQKLWSFMPFSLGPRACLGQSFAMMEAKVVLAMILQRFEFKISPNYRHAPVTAITLKPRYGMQLMLAHYNIEDGEKSPG | |
| CYP776A1v1 | MAMEWWSAIIAALALAKSIALNLVIARVVGFVFSLCRLHVFVRRRLAKQGILGPKPSWLAGNAVEMKRLVASATSADMKSTSNDISARLLPFHHKHAQTYGKRFLAWSVGWEPFVSISEPELIHEILNSTDFEKSGIQNRFMMPLFGRGLVMATGKAWDHQRRLLNPAFYVERIKGFLPTINFCASGLVQEWKGLIRSSSSNVVEVDVHSVLTSVTADIIARTSFGHEFTHREEYVRLERELEVCVLNQPAFCLIPGYRYLPTKQNRKLWEITRKIRSYLYELIDARLATGKDHFGDDILGLLLAATFSSSPSSTKKVPPMSKDVLIDDCKTLFFAGHESSADLVTWSMMLLALNPEWQARARSEVLQVLDGCEVLTSEMLPKLKLIGNILSETLRLYPAAVAIRRKAVKDVVFTKGKLVIPKGVCAEVPILRVHHDPELWGDDVLEFNPDRFSKSEAVAAGSYLPFGWGPRICIGRNFALAEAKVVLSTLLDNFEWEISPSYRHSPRAGVTLYPQHGMQLLLRQLPQN | |
| CYP777A1v1 | MDRVVWVGLIAAACWIAVLKLVELVVKSWWRRRRICQVMEGQGIRGPPCNLLDGNYSEIKRMQAEAAAVDMPALTHDIVARVFPFQHKCTQLYGKHFLHWWGQDPIIHITEPELIVEVLSLKFGHWQKSSQLRRAMEFLFGKGLLVAVGEDWVRQRHAVNSALSAEKIKCFVEVVICCVKPMVRKWEQRVEEGGEAEVEVKQDMLDMATEIILRSSFGDECYDEARRYPELVYRLLGLTSKSSPFNSLIPSFVPTKKNQLLKEIEQCFYRVVATHTQQRNTILSSLLGCAARSSLSVQHVIDECKNIVFAGHETTAHMLTWTMMLLGLHPEWQQRAFEEVAEVCKGRDPTSDTLSKLRVMNMIVNESLRLYPPGAQTAREALKDMKLGDRITIPAGVSVAINIVEVHRSVEMWGDDALEFKPQRFAEGVSRACKQPVGGYLPFLLGPRVCVGQGLALMEAKLALVLILQRLSWRLSPNYRHAPIVALTLQPQHGMQLVISPRRTHDR | |
| CYP778A1v1 | MDVISVLLVALVLALSVLWMVCSFLMRYYWVPSALRAVMEKQGIRGSPNPPNPIFDVFDGKPDMKEISHDILPHVLPWAAQNMKFYGNVHLNWWLREPRIVISEPKMIWDLFMKKHKDFVKSHFIKLLSDDIFHKGLFLANGEAWARQRQIVAPRYYIDEVKAMVRAVNNATSQVMAKWEAFVKDSGDVERELDVQVEFMCLNVDVVARTTLGLEDNDFQNILKYNITLLKLQNDQETWSWLPFARLIPFGINVERWKVRKQLNDLVRKQVRERRKKMTEGNNIDFIGKLLDNPDVREDVIVAELKTLYATGFISLAPLLSFTMLMLALYPSWQEKARQEVDQVLDGEVVSPKDVTKLITIEMILQETLRLYPTMPLIARVCIKDSMLGDVFIPKGLGVSVNVVALHHDRDLWGDDVNEFNPSRFKNGTATAAKHPMAFMPFAYGVRTCIGRAFSEVQCKVIIAIILQRFEVKLSPNYRHHPVITGPLIPKNGMPVILKPRQNF | |
| CYP778B1v1 | MWLVLLAVLAGLLLILWFQCCSFLVYHLWRPKVLEKVMSAQGVTGPPQKNLLTQIWELPDMEQVSHDIVPHAIPFHHARLQKCGPLHINWWRVEPRVELADIDLMRKALLKGPEFFGKSPVLGMIADDIFGGGVFDASGRDWVEQRRVVVPVFHADKIKGMVRTMYENTQSFLENWVTLIRNGGTGEKALDVFPEFVELTAPIIGQAAFGASSSTSIAIVKLLRLLFALQWQQVRYASLPFLPTNRERWRIKREIHRLLRVEIDSRRALTRENCAASHGSDLLGTMLDSNWDDELIITESKTFYTTGHMSLTSLYSWVMLLLAVNPEWQEKARVEVLELVAREGPLDNAQALDKLKLVEMIIMETMRLYPAFPIIPRIALEDCYVDHLFIPKGLAVSVHNTVIQHSAEMWGEDANEFNPGRFANGSLAASKHPMAFMPFSFGARACVGRAYSQVQAKVVVASLLQRFRWSLSPDYRHNPVAAGLLLPKNGVPIVLKLLDSKTIVTNGMKATGERS | |
| CYP779A1v1 | MRKLEAALRGQGLKGPPPIFLAGNVVEILFRREAARNKGMDGISHDIVAHVSPDVAAWSKLYGKPYLIRWLSEPRVVVFDPDSIREILSKQFDKFEKSEQQLEFVLDFIGAGLVGLNGNKWSHHRSVLSPAFHTQRLKAMLSSMTNCTEKLVEKWSRRVGHAKGLETEVEVQQDLKRLAADVISHTSFGSNYEKGERVFQGLTLLGVLLVRCFHNSWLPFFRYLPTKLNFQIWKLRREIDGTLLSLIRERRIAAAKLGERSSHPYGSDLLGLILEEGETGGKSVKFPEQAIVDECKTFYLAGHETSSSLLAWALLLLATHPDWQEKARAEVQQHFPNGVDDGETLSKLKVVGMIILETLRLYPAAGEMNRASSHDTVLSNGIKLPRGTGITIPILSLQHDPELWGPDANEFRPERFANGTTKACKHPNAFLGFSFGPRVCIGQGLAVMEAKVVLAMLLQNFSFRLSPNYRHNPTVQIVIQSFTGIQLLVQKI | |
| CYP779B1v1 | MVFVIPVLATGLVGVIVYALLWKFWIAPSRKEAVLRKQGIKGPSLGPPQLFKGGNKDEVLKRRFSKRKFTLEFDGAHDILSHVLPDIHSFSKKYEMPYMYWWGNELRMTVTDPEVVRWVLSKNPQSFGKSASIQATLIKLLGYGLVASNGEHWAQHRRVVGPAFHLEKLKNIMAGTMVDCTSKVLSRWDNDGEFEIDVEKEFSFLAADVISHTAFGSSFEKGRRIFHLLNLQAELLTKIAFSPMQWMPFGRLHPLRENLQLWEVQKELDAILLGLVKDRRKSASYGRDLLGLMLEQSQDNPAFKDDKLVGECKTFYIAGQETTATLLTWAMYLLSQHREWQDRARKEVLEVCKEDEINAEALNKLKLVGMILNETLRLYPPIPIIQRGTFNDTTMGDKISIPKGIVLVIPILAMHHDKEQWGGDAHEFNPERFARGASKACKHPNAFMPFSFGPRVCIGQTFALIEAKIALAMILRRFSASLSPNYQHCPVSGVTLKPLHGMQLTFIRR | |
| CYP779C1v1 | MAVWLWTIAALCIALVWKGAAKLLLKPWILEAKLRQQGIRGPPRSILSGNVYEIFQMRARTEAECIQGPITHDIVEYVQPHLLHWAKLYGLPLLWWWGTEPGVVLTDLDMIKEVLYNKSGAFWSPEWQRKFQVDILGRGLAVVNGDEWAFRRRILAPAFHAEKIKASNCEMLEKWNALTEGKDEPIELEVCKELTTLTSDIISRAAFGSSYKKGHKVFELLDQVGGLTCFPLAKRFTHCHSMLPICKLNREIKTANSKLRSTLEEIVQARRDQKLAGEIDNYGSDLLGIMLDEVDAGHHDDKTGLSFTTDSLMEECKTFYIAGQETSAKWLAWTMMLLAANPSWQEQAREEVRQVCQSQAPDAESLSKLKIVGMVLNESLRLYPPAVFNVRSCYKDAKLGHLSFPEGSGVIIPILYLLHDKDIWGDDANEFNPQRFADGISSASKSRHSCAFLPFSQGQRVCLGQSFAQIEAKVAMAMILQRFSFRLSPTYRHSPVHRLALQPQHGLPLLLGRP | |
| CYP779D1v1 | MEGIGKAVWTGALLVLLALVAKLWRSIVTRYWLEPRSLDTRIRSQGIQGPPRTFLAGNMLQVMKMRDTPKERDMAGLNHDIVEHVLLDYHQWSKEYGKMYFYWWATEPRIMVTEPELIREVLAKKVTQFEKSDMMVSAVASIIGRGLIAVNGNEWSHHRRVVAPAFYLEKLKKMVPRIGLCALEMLDRWEEALREQPEIEMSSEFSKLTADIISHTAFGSSYLKGQKVFETLRAIPEELSKVDRYNYVPGKSMNPFSELNRAIRNGQKKVNNLLLEIVHARQQLKDSGASSNYGSDLLGLMLDEVDSSRSFSGSGIKPALAFTSESLIEECKTFYVAGHETTAKLITWAMMLLATNPTWQERARAEVLEVCKSGVPDSEAASKLKIVGMVLNETLRLYPPAVFLVRTAMEDTKLGNLIVPEGTGVLVPILSILHDKEVWGEDANEFNPQRFADGVANASKHPFAFLPFSHGPRVCLGQGFALMEAKVALTMILHRFSFEISPSYQHSPVLRLTLTPKHGMPLLLSRRGV | |
| CYP779E1v1 | MEGSWILSTCIVLFTAALWRFVTVYWWRPRVIAAQLKKEGIQGPPPRFMVGQIAEIQNMRSAIKDHDMGSFSHDIFHRVHPSLLKWRKQYGKRFVFWWGTEPRISVSEPEIVREVLSKKFSQFDKSEAGLRLANLFLGRGLVSVTGEEWSHHRRLVAPAFFHERIKQMTGTITGCASRMLDQWEATRQQNPEIEISGEVRKLTGDVISHTAFGTSYLEGQRVFEILSKKFPELMPKLVSFSWIPGFRFLPLPINLRLWKLHQKLDSLITGIIDERRNSVKSGGSNTYGNDLLGLMLKECDSSTNFTSRDLIEECKTFYIAGHATTATLLTWTLMLLGGYPEWQERARAEVHEVCGNEIPDGESVSRLKLVGMILYETLRLYPPVVEMTRECVEESWLQDLHVPRGVSVSFPIAGLHQDKELWGEDAGQFNPDRFKDGISSACKHPNAFMPFSFGPRVCVGQSFAMIEAKVILAMILQRFSFRLSPNYRHNPAMKHGLKPTHGVPLVLSKM | |
| CYP77A4 | MFPLISFSPTSLDFTFFAIIISGFVFIITRWNSNSKKRLNLPPGPPGWPVVGNLFQFARSGKPFFEYAEDLKKTYGPIFTLRMGTRTMIILSDATLVHEALIQRGALFASRPAENPTRTIFSCNKFTVNAAKYGPVWRSLRRNMVQNMLSSTRLKEFGKLRQSAMDKLIERIKSEARDNDGLIWVLKNARFAAFCILLEMCFGIEMDEETIEKMDEILKTVLMTVDPRIDDYLPILAPFFSKERKRALEVRREQVDYVVGVIERRRRAIQNPGSDKTASSFSYLDTLFDLKIEGRKTTPSNEELVTLCSEFLNGGTDTTGTAIEWGIAQLIANPEIQSRLYDEIKSTVGDDRRVDEKDVDKMVFLQAFVKELLRKHPPTYFSLTHAVMETTTLAGYDIPAGVNVEVYLPGISEDPRIWNNPKKFDPDRFMLGKEDADITGISGVKMIPFGVGRRICPGLAMATIHVHLMLARMVQEFEWCAHPPGSEIDFAGKLEFTVVMKNPLRAMVKPRI | |
| CYP77B1 | MDLTDVIIFLFALYFINLWWRRYFSAGSSQCSLNIPPGPKGWPLVGNLLQVIFQRRHFVFLMRDLRKKYGPIFTMQMGQRTMIIITDEKLIHEALVQRGPTFASRPPDSPIRLMFSVGKCAINSAEYGSLWRTLRRNFVTELVTAPRVKQCSWIRSWAMQNHMKRIKTENVEKGFVEVMSQCRLTICSILICLCFGAKISEEKIKNIENVLKDVMLITSPTLPDFLPVFTPLFRRQVREARELRKTQLECLVPLIRNRRKFVDAKENPNEEMVSPIGAAYVDSLFRLNLIERGGELGDEEIVTLCSEIVSAGTDTSATTLEWALFHLVTDQNIQEKLYEEVVGVVGKNGVVEEDDVAKMPYLEAIVKETLRRHPPGHFLLSHAAVKDTELGGYDIPAGAYVEIYTAWVTENPDIWSDPGKFRPERFLTGGDGVDADWTGTRGVTMLPFGAGRRICPAWSLGILHINLMLARMIHSFKWIPVPDSPPDPTETYAFTVVMKNSLKAQIRSRT | |
| CYP77C1v1 | MVAFLAVAVAILAASLLLLSRRRTHLPPGPRGFPLLGNLLQMRSVLGSPMNLQNLARQHGAIMTLRVGSVPLVVISSSQLAHEALIEKGSIFSSRPSLSERQVRLSNYRRSINAAPYGHHWRTVRRNMVSHVLSPHRVHAFEPARQRVISELVEKLRQTSQRADSPDGPSAVPVLATLRFTVFSLLSYMCFGQWLDKDAVNGVERMLRHLITSAGRGGRMSDFVPLLKIVQRSPRDLKLEELVGERRELLLPLIQRAKLLAAEDKLDQNSYLSSLFSLQRQEDHQLKLTDEDLIVLCSEFLNAGADTTANTLEWSLANVIKHPAVQKKLLEEIHSSVGDKPVTEKDIDKLVYLKAVVKETLRKHPPGYTTLPHAVTEPCKLGGYDIPVHATLLFNIYAINNDPELWTNPDEYKPERFLEGPGASADFTASSGALNLIPFGAGRRICPGLGLATLHVHLVLARLVQEFEWNTVPGETAVDLTPIQEFTVVMKQPLRATLKARRL | |
| CYP77D1 | MDLAALVATIALLALASWSLFFLQHLNLRRRMPPGPIGWPVLGSMREIPRLLSDPQKFQQLVARYGPIVTLWNGSVATILISSPDIAREALVEKGSVLASRPDVPSMRLLTSGFKTINSSPYGVHWRATRKNLVSGILSPRVMSGFAPVQEQAAEDLVRKLASEAKQSGGTVESLSVSVRCVLFQILSFVCFGRKLEEAKLEELNALMKEATTMLHPVLGDLVPFLKVFTSHTKQKSFLVRQNQLLKGLLSRECPAASESYVQTLLSLQGKNLEDVDLDLAVLVRELFIAGADTTTNCVEWSMANLIKYPGIQERVFRELAENVGQKSGVKVADLPKLPYLHAVVKEALRKHPPVYLSAPRTPVHATKLAGYDIPKESTVVVHLQSLSNDAGVWKNPDKFLPERFFEQTELSKRMSMIPFGAGRRDCPGKHLGMLHVHLIVANLVQAFEWRAEGKEVDLSPRTVFTVQMKNPLRASICQRRS | |
| CYP780A10 | MELFLQSWNALYFLVFLLSLWLISQKFYKSSTIKLPPGSHGLPLVGESLSLFWGSPLDFLSTRRKRFGGVFWSNLLGSPTIVATTVGSAKFFLSCADCGPSGLFARLIGPKSASEVIGSEHALYRRIILGMMVPETLKCHVQMIDILAQETLESWGSKKTVSVMEETVKFSYCTVIGLVCQKLLPSTPEMIDLMRDVQTIENGVLQFPINLPFSPYRKALQQARARLHRFLDGLINERRAELAANGETDKDALDEFITHKDDKVGFLSNQQVEDNLMTMLFGGHHTTALALMWLIKHLNENPQAFKEVEEEQRRILLGKRSTKYSLTWEDTRQMPATLRVAVHESLRLSNVVGVVTRKITKDISYKGYTLPKDWMIHVYMPPIHLDDSIYPNAAKFNPSRFEVPAKTGTFIPFGYGDRICPGRALSQLEQMIFIHRLITKYRWEPVNPNSTTSYWPMPSVKDGYLVHAMSI | |
| CYP780B1v1 | MALWSLYLLLVIPAVAAFLISSKRKSGSVQTPPGNRGWPILGETIQLLRGTAEDFVFQRRKRFGDIFSAHLFGRQSIVISTPEAVKFFLTNPGARNCCSPSNSGFLIVGKESVGHVEGATHARYHRAILSSMSGDPLNNHVQRFDKIAMDLLTSWQRKGCVTVLEETLQLTFDVVTAFICDDPRIFQTKTGDFMHDVTVASRGLFKLPINLPFTDYHRALQARKRLHYHLDRLINERRISKITHDDLLHKLMNDKDLNSTNQQIEDNIVGLLFAGQHTTPLTLVWMMKRLQENPEILKEVVEEHQKILREREQPHLTWEDTRRMPVTMRVLQETLRLASGGMLVREMKHAVEYNGYVFPKGWTLHIFHTAIHLNEDYFADPYKFDPSRFLVPQKPGTLIGFGCGLRTCPGAELAKLEILVFFHRLVTQYSWKPKAPNGAIRNWPLRIPEDGYVVEINRK | |
| CYP781A1 | MFWIGWLCVAAAGALLASLGNVYSHWQKLPPGPWGWPIVGCLFCVSRRNLHRSFAELATKYGPIVYLNMGSRATVVISSPEVARAVFREHDVQFASRPRYSTPFKHISQNFKDLVFAPYGGRWKNLRKICSTELFTASKVNMFGGIRKAELHDFCNSIAMRAAAGEEVNLSVCFQELLTNLMSSVLFGKKFYTSDLPPVAEAAAYRATWGMLTQESGKIYLGDYIPALHWLDRLRGKDQRIRKTIIPALQGLLNSVIEERRKQLRRDKPRDFVDVMVALNDQKSLSNDEIVAIIQDMLLAGTGTTRSTLEWGFSELVRHPEVQRRAQEELDRVVGRERYVQESDLSGLPYIQALVKEIMRLHPAAPLGLPHFNSCPVSLAGYTIPANSTLHVNIWTICRDSSSWERAHEFRPERFLGSCHNLLGQHFELIPFSSGRRRCAGINLALLHVSLTLAYLLHRFEWRPPPGVDVSEIDMSETTGLACFRTVPLRVSVRPRLELP | |
| CYP781B1v1 | MARKYGPVMSFRLGVRPHIIISSPEMARQVLKEHDVEFASRPLFSTISRLVSHNFQDLIFAPHGERWKMLRRVCGTELFTASKVSHFASTRKRELGAFGAIVEASAKDGHEFDLSSMLHEYFTNLMTCVLFGRKFYGTDTPLTPEAEAYKASWAIQAKESRRLFAGDYIPAMRWLDTLRGTQNRLKNEVLPARSRFLEAVIEEHAKDFDPENPRDFVDVMLTLGGEDKLSNDQIIALLQDLLLAGTGTSKGTIEWAISELIVNPRVQEKAHEELDRVVGRDRPLEESHLNDLPYIQAIVKEVFRKRPIAPLGVPHYNDREVTLAGYTIPAHTTVLVNIWAIHHDPSVWSDPELFLPERFLGSDHSVLGNDFDLLPFSSGRRRCVGIPLAMPHVTLTLAYLLHRWSWRSPLGKPIEMAELAGAGIAGVASPRIVCASHR | |
| CYP782A1v1 | MEGHLAFAAIILGFLLFVLRARKSRDRAALPPGPFQWPLIGCLPSFPFHHRHRGFLELSKKFGPIVTMPIGSSKIFLVHGKDLAMEVLRFKDAQFSSRPLSMTGKYIGFEHSDPNLCPLNENWRVVRKAFSNELMAPSRLSSQAWLRREEVLKIVDSLLGITGHGRDWASVDVRKIAEGVVGRIIMRMLFGDHYLGKNIDNPDAGRITRELEREFEKYLAEGNFLWGELNLADYFPALGIFDLQGLEGRFKRLMSKLEPLFTMIIQEHRKNTVMIQDEKGKDVIDVLLQNQLSDKQIMGILSDALLPGIGTTSAAVEWAMAELAANPHTLSRAQQELDSVVGRSRLMDESAIPSLPYLQAIAKEVLRLHPSAPLDDPHLNEEESSLGGYAIPAKSTIFVNLWALGRDDRLWSDASRFDPDRFLGTEIGVHGSHFELLPFSSGRRRCPAHALAMIKLQHIVGALVHGFDWSSAGAVDLIEGNGIIASPRTPLRLRARRRLDDEAY | |
| CYP783A1v1 | MEGAWFLALALLILLWWSRNLRARLKLPPGPFPWPIVGSLFIVKEPLPIFFAELGRKYGPVVYFKLGMVPTVAINSAAAAREVLKSRDLEFASRPDLGNLRQISFDYNDLGVAPYGETWKLMRRVSATHLFTPSKLNTTASVRHREVKAMIKNILDEGPEVVDLTAATNAAVVQGVVNLLVGTDDKSLGIDARSLNGIFEKAGEELLNVNLGDLFPFLRRFDVQGLERRFKYVVMPPIKSLMEKIIAHHKSSSREVEDFVDVLVNLNGEDGLTHIQTIGLLSDFFIAGINTSQTSIDFTLAELVRHPAILSRAQKEIDQVVGSSRLVQESDLPRLPYLHAVIKESLRLHPPLPLLLPHHNPAASKIGEYDIPAKSTIFVNAWAIGRDPSTWDRPLEFVPERFLERDVKLTGDDFSLLPFGAGRRTCAGYLMAMRMLPLSVATVIQAFDLATLEGREVDMGESTGGATRRNKNLMVSATPRLAKELYA | |
| CYP784A1 | MELLDLVIASCILALSCTWLMSFRKPQGRLPPGPLSIPLFGSIFSLKQPLHEHFIHLSKKYGPLIYLKVGMCDLLVANNSAMAKEVLQTHDVEFAYRPDTRSFRLFSLGYKDLLFAPYGDSWKKLRKVSTTHMFTSSKLNISARQRETELLSIIRSIKTSFDSGNSVELRDLVAEYNTNVICLMLFGQKLEAAKTVVGLVEKTASLSLLINIGELFPLMDWLDLHGIYKIMKKEILPDIKKVLGEIIEQRNGTRKEGQEARDILDVLLTLKDDDGVSEASVMALLMVMFTAGLESSQNVAEFSIAESLNHPHIIQRAQQELDAVVGRKRLVREEDLVKLPYVQGIVKETLRMHPPGPLGIPHANPKPVSIAGYTVPANCKVLVNMWAIGRDPACWDRAEEFLPERFINSDYDVAGNHFHFIPFSAGRRICVGYPLAMRSIPLVVATLLHSFEWKRQDGNSLETAKGLLSIKLASKINLSGHPRLDESAYY | |
| CYP784B1v1 | MGITIAILVCLLATAAIIKSLRARRSSRLPPGPISFPVVGSLLSLRQPLHRHFARLADRYGPIVFLKIGMVPYVIANTARAAEFFLKIHDAEFANRPQSEEFFRIFSFGWSDLAFRSPGPEWKLMRKICATNLFSNAMLATSAPYRRSQLQSAMDAILDRSRGGEPVNLRTLFARYTSGSLCLTLFSEECPEVVETINNMAGQAINLNIGEIIPSLDWMDLHGVYAKMRGEIMPRIKALLDDQVREHQERKKAAGDGFVCRDFVDVLISLDESDKLSDQEIIGLLCDMVGAGFKTSMESIEWCMAEVISKPEIMRKAQEELDQIIGRERAVEEHDLQNLPFIQAILKEALRLHPAVPLGMPHYNLRPVELGDGHGTIIPAKCKLLVNLWAANRDPAHWTSPHEFQPERFLGTNISPGGQHFQIIPFSAGRRMCAGYGLAMRSLFFLLASLLHGFIWSEISDNPIALEESIGTISCPPAKDLIVAASPRIEERILAQY | |
| CYP785A1v1 | MDLLSLPSLSALVLLAAALWCSSTRRRNPPGNLPPGPLNLPVIGCLHKLGSLPHISLHKLSKRYGDVMHLKLGSVSTVIISSERAAREIFKRHGLEFASRAPLICGKYFGNDYSGLVFSQYTPEVKLYRKLINTHLLSPTKLKSYDGIRREEQRRLARSLSDDRGNPVLLRQKLHIMNMNVITYMLFGKHFCGHYKNTANVDEFVQTVVEMVRLAGIFNVSDYIPGIRWLDVQGLEKKYKQLMNQVNWHLLGILRDRLVDPPVFTSEEPMSFIDVLISMGEKLSDTTKITLLLDVLMGAVDTSALSLEWAMAELLRHPAEFSRVQSQIDTIVGKKKLVDESDIAKLPYVEAIAKETMRLRSVVPLGLPKIVQGGPIELDGYTLPNGTVIYISSYSIGLDERFWKDPLEFRPQRFIDLPDIDVFGQNFNLLPFGTGRRVCPGAKLGFDAVQMGIATLVQGFDWKLDGDLDDPAKLNMDQTFGLVCQKSQPLVAIPIPRLDSHVY | |
| CYP786A1v1 | MAAQASSTAALSLLWSLLVAIWKKTLGKLFFRDDRHQQDHMPPGPRPLPVIGNLHQLLGRPPHQALLDLSKRHGPLMFLRLGCVPTFVASSAEAAREFLHTHDLVFASRPRYAVARELTYNFADIMWAPYGDHWRHLRKVCSLELFSGKRVDSFERLRKEEISSALATVEEAARASSVVDLRAVLSDITLYSILRMATSQEFGGKKKQLSRFERRVKETIEHAVEMIGALNVGDYLPSLRWMDLQGYGRRARKLHALQDAFFQSLIDRKRQYQGRGGAGAGGVDDLLDVLLASQEKNALTDDTIKAVIQDVIGAGSDTAWVTCEWAMAELLRHPTAMRRAQREIDAVVGRDRVVEESDLPGLNFLHAIVKETLRLHPPSPVILYESTMPCVSSAGYRIAQGARLLVNVYAISRDANSWERALDFWPERFEEGAKKGVDVRGQNFELIPFGSGRRICPGMGMGLRMVQCVLARLLQGFDWEKVGEIDMREKFGLAMPKLVPLQAIPCPRRS | |
| CYP786B1v1 | MNLFAAAAFLVIGLVYWFVNRQRPSTPPGPWKLPVVGNLHQLLGKQPHRVITELSKKYGHLMSLRLGSVQAVVASSSQTAKIFLQTHDVIFSSRPEVANAKLLTYGFSDIMWAPYSQQWRELRKLSVLELFTAKRLESFQGIRRDETLNMIHRLLKLAREKKVVNFRDAATELSWSIIGTMVSNRQEFVNLEEGLKVKSSLDRALQLAGAFNLADYIPFFRAFDVQGFRQQSQILHEQLDFFFQGLVDSHRRQERPPNASEDFIDVLLSIQKQNGVEYVSDDTIKATIQDIFAAGTDTSSMTLEWALTELVRHPRSLQKAQDEISFIVGNDRMVSEADIPKLQFLQAVVKETLRLHPPGPLLQHQSMEDCKVGPYSFPAGTRVIINVYGISRDPSLWEQPLEFDPWRFLDKPTASIDMKGQHFEFIPFGSGRRICPGLAMGVRTVELALAQSLHCFHWHSPDDRVPDIEEVCGMTLPKKNPLLLAPSPRLADAVYGEIQRM | |
| CYP787A1v1 | MPTSCCKMKLPGTFVFVIGSLLFTLLVVACQALDHLSTSPGTQMNWFLLTFACILTAVISVSWWLMLKSRLRLPPGPMALPIVGHLHLLLKLPHQSFHKLSHKFGPIMTIKLGNKTAIVISSKKAAKEILTSYDRVFASRPVLISPQSLCYNSKNISCCKYGPYWREMRKICTTELFSSKRLSSFQNTRLEETQNLLQRVAEQLKVPLNMKIELSTLTLNVITRMAIGKKFRHGECSEDAEPLNVILEAVRLMGAVNLGDYIPFLKRLDPGGYIPRLKTTSKKIDCILQRLVDDHREEKVKSGDLVDVLQSVGIEDSAIKAVILDILAGGTDTTAVTTEWALSELIRNPDCLRKVQQEIHVIVGDSRLVNENDLHHLHYLKAVVKETFRLHPAAPMMAPHESIEACTLKGYTIPAKTWLLINAWSMGRDPAQWDSPEEFMPERFINSSIDVKGCDFELIPFGAGRRMCVGMSLALCMVELTLARLVQAFHWALPDGSTMNMEERQGVIVARKHPLIAVANRRLPPEVYINTL | |
| CYP787B1v1 | MEALSIILVGAATLVLCSLFASRFLYPLPPGPWGTPLFGHLYSLGELPHQTLSKLSKKYGPIMTVRLGMVPALVIDSPQWAREFLTTHDIAFASRPQNTNSKYLFFNGSDVGFSPYGEHWRNLKKLITMELFTAKKMEVFKALRANGILRVLKSIAAEEGNVVSIRNLLSMLNMNNISQMAFSKQVIDDPIFQRFLAVLEESLDLMAVFVLGDFIPFLKWFDPYGYVAKMKANRKEISGIYQMIIDEHKLKRKKNCTPTDLVDILLSQGVDETTIKGTIMGMFVAGTDTSSLTSEWALTSLINNPGCMRRAQEELDRVVGRERRVQEEDLSSLVYLKAIVKETFRLHPPAPLLLPRESTQECTVKGGYKIPKGTRLIINTWSIGRDPAETPSPEEFKPERFLGKSIDIKGQDFELIPFGAGRRICAGLPLGQTMVELTLASLLQAFEWKTDKTLDMEESEGLTTRMKVPLAAHVTRRTSLKF | |
| CYP787C1v1 | MAALVVTLLIVLLPLLLWWLRIYQSKKNVAPGPLAIPLLGHLHLLGRHPHKALSILSKKFGSVMSINLGSVPTLVISSPDAAKTILSTQDIFFASRPRTAAAKFIFFNARDMVWCEYGSYWRTMKKVSTLELFTAKRVEESKKLRMEEISRLVTSIAREGDNGRVAIDMNAKLSMTNMNLVSFMAFSQRFEESSFVELLQEAIDLVTSFVPSDYFPYLSWMDDYLGTVPKMKAVQGKLDKIFQAIIDEHRRVNGEKQRAPDLVDVLLSLDEVDDNDRKGLIMDMFGAGIDTSSITTEWALSELIRNPACMLKAQREIDQAVGFDRAVNEDDLLNLGYVRAIAKETFRLHPPVPLLIPHESTQESLVNGLRVPARTRATVNVWSIGRDPRWWERPEVFDPDRFAARSVIDVKGQHFELLPFGSGRRMCPAMGLGLAMVELSLARLIQGFEWNLPAGLQELNMEEEFGVTLRKRVHLSALAMPRLKAELY | |
| CYP787D1 | MASFVALFLLTLSLGLLWRILTKIVDRSLPPGPPRVPLLGHLHLLGVLPHKSLSDLSSRYGPVMLLWFGFAPTLVVSSPDAAREVLCTQDLAFASRPKISIAKYMFYNSKDLGWTSYGPYWRLMRKVTTVELFTAKRLEESRMVRHAQVSKLIGFIVNNGQNGKASVNMKFLLSILNLNVVSLITFGREFPAGSVELIEEVMQLMGSFVLGDCFPFLSWLGSPVIRKMISAHTKLDQLLQEIVDEHKSKFKSSERARDFVDVLLSLEDQGEIDIQCVKAMIMDMMLAGTETSAITTEWALSELMNNPTCMIKAQKEIDTIVGRERMVVEADLCKLSYINSVVNEVFRLHLPAPMLLPRHSTQDCLVNGYKIPKNSRVLVNVWSIARDPSLWESPNLFNPDRFAESSISFKGKNFELLPFGSGRRICPGLSLGVAMVSHTLARLVHGFEWKVSGKELSMDEISEGVAVRRKVPLEVFATPRLASHAYL | |
| CYP788A1v1 | MNLSSIMGEYTQHDNFTAVASLSLVLAAAIALLAALFSRLRNSKRPPLPPSPPSKLITGHLHLLDQLPNQSLYKLAKIYGPLIQLRLGVVPVVVASTAEMAREFLKVNDSVCASRPRMAAQKIITYNFTDIGWAAYGAHWRQLRKICTLELFTHRRMQETAKVRARELADTMAGIYRDRETSINMNTRIFSLTMNVINQMVMRKKPFSGSDTKEAREFIDLINGVFMVWGAFNIGDYIPGLSIFDFQGYIGMAKVLHKKLDHLLDKVIEEHIQRRMAKSDEPPDFVDVLLALTLEDGSKVSHKTIKGIIVDMIAGGTDTAAVTIEWALSELMRKPHILKKAQEEMDRVVGRDRVVDESDLPNLPYLECIVKEALRLHPSVPILRHESIEDCVVAGYRIPKGTGIMINVWAIGRDSATWENPMEFDPDRFISAGNTLDVRGNHFDLIPFGSGRRMCPGMPLGISMLQMSLGRFIQCFDWGLPPEMKSAEEIDMTETFGLTVPRKYPLHAVPIPRLPAHLYQA | |
| CYP789A1v1 | MVEIPQLLSSSTPELYLKLAVAGSLLVLLLLLLNLPSSRGARRKSSSSSSSSGSSSSSSPPLPPGPRGWPIIGNLLDVGTVPHEGMMKLTRAYGPLVYLRLGAIPHVVSDDPAIIKEFLKIQDHIFASRPGNVILAELLTYGGKDIGFAPYGAHWRNMRKICTLELFSAKSVDSFQRLRRMEMIHTLGLILDAAVDRRAVDLRDAFNGLTSNMMTRMLLGKRYFGPGDPGPEVGAELKAMIAEGILMMNGFNISDYLPFLRFLDLQGQERRMKQIMRHIDGLATALLLELAPRIGKKPESFVDILVNLRGENGEPHLPEDVMKAVMVDMMAAGTDTPGVSCEWAMAELLRDPALLARVREEVDRVVCVDRLVDESDLAHFRLLRAVLKESFRLHPVGAILIPHLAMEDAVVAGYGIPKDTRVLINVFALNRNAQVWERPHEFDPERHLRGLGEGAVVEFGDPECRLIPFGSGRRMCPAASLGLTMVLLALANLVHAFDWEVPANLSMERAPGKMVKAQALTALARPRLPRHLYSQQI | |
| CYP78A5 | MSPEAYVLFFNSFNLVTFEAFASVSLIIATVAFLLSPGGLAWAWTGSSKSRVSIPGPSGSLSVFSGSNPHRVLAALAKRFKASPLMAFSVGFSRFVISSEPETAKEILSSSAFADRPVKESAYELLFHRAMGFAPYGEYWRNLRRISSTHLFSPRRIASFEGVRVGIGMKMVKKIKSLVTSDACGEVEVKKIVHFGSLNNVMTTVFGESYDFDEVNGKGCFLERLVSEGYELLGIFNWSDHFWFLRWFDFQGVRKRCRALVSEVNTFVGGIIEKHKMKKGNNLNGEENDFVDVLLGLQKDEKLSDSDMIAVLWEMIFRGTDTVAILVEWVLARMVLHQDIQDKLYREIASATSNNIRSLSDSDIPKLPYLQAIVKETLRLHPPGPLLSWARLAIHDVHVGPNLVPAGTIAMVNMWSITHNAKIWTDPEAFMPERFISEDVSIMGSDLRLAPFGSGRRVCPGKAMGLATVHLWIGQLIQNFEWVKGSCDVELAEVLKLSMEMKNPLKCKAVPRNVGFA | |
| CYP78D1 | MRNEVLSTIFLLLIFFTTTINPSSSQLPWLFSLLYLSLAMAVVALPPLLAKRHGHARRVNGGGAAIPGPRGWPLLGSLPAVSGPLMHRRLAALAYAHGGGARRLMSLTLGATPVVVSSHPDTAREILAGAAFRDRPARAAARELMFLRAVGFAPAAGDDGGAYWRRLRRAAGAGMLSPRRAAALAALRARVARRTSEAVSRGMAVPPGRVAMRALLHAASLDNMVGSVLGLEHHDHHGGVISDMGDMVREGYELVGKFNLGDYYSTTQYQCLWGLLDFHGVGPRCQRLAARVREQFGRVMEERRKVSDLHKRDDLLSYMLSMPQEERIEDSDVIAVLWEMIFRGTDVVAILLEWAMARMVLHPDIQSKVQEELDRAVGHRPMTDSDIPSLRFLHCVIKETLRMHPPGPLLSWARLAVHDTYVGKHLVPAGTTAMVNMWAISHDETIWGDPWVFRPERFMEEDINVLGSDLRLAPFGSGRRVCPGRMMGLSTSYLWFGRMLQEYKWSPAQPVKLTECLRLSMEMKKPLVCHAVPRSKTG | |
| CYP78D2 | MKSIPANLSSILFCLAVITHQTPWPVALLLFSLSSFFAFSLNYWLVPGGFAWRNHHDNQNPSRFRGPIGWPIVGTLPQMGSLAHRKLASMAASLGATKLMAFSLGSTRVIISSHPDTAREILCGCSFADRPIKESARLLMFERAIGFAPSGDYWRHLRRIAANYMFSPRKISALEPLRQRLANEMVAEVREEMKERRVVVLRDILQKGSLSNVLESVFGSDVSIEREELGFMVKEGFDLIAEFNLDDYFPLRFLDFHGVKRRCCQLAGKVNSVVGQIVKERKGAGDSRSGSDFLSALLSLPEEDQLNESDMVALLWEMIFRGTDTVALLLEWIMARMVVHPEIQAKAQEELDTCIGGHREVQDSDIPNLPYLRAIVKEVLRLHPPGPLLSWARLAIHDVHVDKTFIPAGTTVMVNMWAITHDPSIWRDPWSFNPDRFIEEDVLIMGSDLRLAPFGAGRRVCPGKALGLATVHLWLARLLHEYRWLPAKPVDLSECLRLSLEMKRPLECHVVQRRSKVTQ | |
| CYP78E1 | MAERPARLWPLTDFPIFISKGDIVCKDSCIGRFQKYQNVGRAVAKKFREFLSALTKSKACKPVNSVIKALAAPLILIAIAQEFSRDAVKQFLLDGFLTQPLRWLFQYISPFIQQVGTVDTATWTDVHASSILVFFIAAISLIISIVGWCGPGGPAWSFSRIFSPSNKLPTPNGPRGCPVIGSWTLMQGSEMHRELARQAWAGGPSTRNLMALSVGTTLIVLTSDANVAKEILRSAVFGERPLKQAALDLGFERAIGFALQGPYWRHLRKVAVTHMFSHRQIVTHSELLQRETLRMISAMVHSIRTDCVKDYRVGLCARPFLQRAAVNNIMTIVFGRHFDFGNSCDEAEALEAMIREGFELLGGFNWADHLPLVRHIPFLSFSRRCRNLTMKVRAFVQSILDERRRCHHQSHSATSSVLNTSFVDALLSLEGDQKLQDEDIISILWEMVFRGTDTIAVLTEWALAEVILNQGIQARIHEELDAVVGSNRLVQQKDIENLPYLQAVLKETLRSHPPGPLLSWARLANEDTQIAGCHIPRGTTTMVNMWAITHDSSVWPNPEVFDPSRFLKSEGGSDLDVLGTDLRLAPFGSGRRVCPGRALGIATAQLWLASLLHHFSWSQDLSHPIDLTDNLTLSCEMASPLHGCPTVRFPL | |
| CYP790A1 | MDSLLLELPLVAFIVSLFCFILNRFSRSKQQFSLPPSPPALPVIGHLHLIGDLPHHSMLELSKKYGEFMFLKLGSLNTLVVSSPDAAKIVLKTLDPEFAMKPEHLEAKYASYGGRGIIFAQYGEHWRQARKLCTVQLLSTKRVESAEPNRKLEMGLLLADLWKCADDGAVVNLTNKLSDFAFNVMLKMVTGKSHSSSASSRDEEEQARSIKEGLMEFVREGTGMHIATFFPWLTWVDKQVYKLASVHKRVDKILESEIDRHREKLGKSQPSMQHENFIDVMLMDSDANDAHIKAMTVDMLAASTDTASITSEWAISELLNHPAALAKVQAELDEVIGQERTMQESDIRSLTYLQAVINETLRLHPPVPIYPRENSAQACMISAKWGVPARTRVFINAYAIGRDETLWKEAHRFKPERFLEEKVGIDARGQDFELIPFGAGRRMCPGMQLGHTNVMLAVGSLLHAFNWIIPGADNGTGKVDMQEHFGMTVARAAPLQLLPVPRLPAHALALKPGI | |
| CYP790B1v1 | MVMEQVCTKPQLPPQLPPSPTGLPFIGHLHLLGKLPHQSLLKLAQQYGDVMFLKLGKVNTLVVSSSDSAKEVLNTQDHIFGSRPKTTFSETIGYGGAGLAFANGENWKSTRKVCMYEVLTTKRVESFHPIRKFEVSLFMNELLKASREGSAVDLSSKLSDLTFNVISTMVLGKSYSASALSEAEKKETMFFKETLDEAAIMAGFHAGDYLPIPDWMDTQVNKIKQLQRDLDQFIQKEVESHRQRRDPGQAPRDFVDVLLSNSHISDTSIKALIVDMVGGGTESSAVSVVWALAELIKNPRLMERAQRELKEVVGEDRSLEESDIPNLPFLQAIVKETMRLHPPGPLLIPHESTEECEIGGYTVPARTRTVVNIYAIARDEDNWEDPLNFDPDRFMGSNIDLKGRHFEYLPFGSGRRICPGLMLAMATVQFILGSVLHGFNWRLPSGQTIDDLDMSESFGLTVPKAVPLKLVPSPRLEPQIYVKSLSS | |
| CYP790C1 | MEFLRLALAFLKALVFKLGCMSKSSSKSFSLPPSPRAVPLLGHLHLLGKLPHQSLQKLASRYGDVMLLKLGSHRTLVISSAEAARAVLKTHDHVFSSRPSTVAGKIFGYGGAGLVWAPYGEHWRTVRKLCTLELLTAKRVETSHPVRKREMAFVLDELSRHQQSDKQLEPVDLTTKLSDLTFNIMTRMVMNKSYLTGTSAEKEAAVRFKDLITEAFVVGTSCLSDSFSWLAWVDPQARKMERIHQQQDAYLSKQIAEHRQQPGSNGDFLDVMLAMEELSDTSIKSLSQDMLGAGTDTTAVTVEWALSELVKDPALLRRAQEELTEMVGDKAMVDESDLPKLRYLQAVVKETLRLHPAGPLLLPHESAEACVLENYTIPAKTRVIVNAYAIARDSRWWDEPLKFDPERFLEKCQGMDVRGQSFEYLPFGSGRRGCPGVTLGMTTVMFILANLIHAFDWKLASGEEMDMTEAFGVTVPRASPLKLVPSSLNLEFPPKFKS | |
| CYP790D1v1 | MLEMILTIVLTLALILVVLFCTNKRNQSLPPSPRALPIIGHIHLVGKKLPHEYLFRLAKQHGGLMYLQLGRIKTLVASTPAAAEEVLKTHDRAFASRPANSAAKYFGYDATDLVWAPYGDHWRHLRKICTLEFFITKRVQMFQPVRKLEMSMLITELVEACNQRRPVDMTSRFFQFAFNTMSRMVLNKSISDASGSESEKLKEFLNNLNEASKVGNGLQIGDLIPCLSWADPKVFRIKWLQTQLVNYLGEQLQEHKKNRESHDEVKDFMDVLIAGGVLDDTRIKALTSDMLAAGTDAIAVTIDWALAELMRNPELMQEVKQELEEVVGSKGTVEEEHIPKLEFLQAIVKETLRLHPPAPLLAPHESVESCNIWGYNIPAGTGLLVNAYALGRDESTWSEANKFNPKRFLETKSDVQVTGQNFELIPFGSGRRMCPALSMGLTMVHYALATMLHTFEWSLPDGKDEVNMKAYFGIVLIREEPLMLVPRLAKSCP | |
| CYP791A1v1 | MEGLFQIGTAAIFLSWIAWSLFFAPRTRIYRGNLPPSPGFALPIIGHLHLLGNLPHVSFIELAKRYGPCLMLKLGSYPSLLISSPEFAREALKVNDIVFSSRPSLAASRILADNAAGILWAPYGQEWRNLRKLCSLELLTSRRIEESRPVRAAEVAAAMARAKEISKAGICVNLTSLLEDLTFDIMRVWVMGSSESSRSSAGVYKRVMKESFVAGGEVHVGDYVPWLWWLDLAKVARMKRVHGEIDDIIQKEIDEHCGKRSGSDDFITATLRNNEICRTDRDRKGLITDVIGGSTDTSALTVEWAMAELINNPRSLERAQDELLQTFGKNSLVEEDRLEELEFLTAVVKETLRLHPTAPILIYETTHECQLERYTIPPKTRVFINIYGIARSEASWSDPLAFKPERFLGSGAIDVRGRDFEVLPFGSGRRGCPGIQLGFTMVMLVLANLLHGFHWSLPPGLSRLDMSEESGLTIPRAIPLELLAVPRLDARSYSV | |
| CYP791B1v1 | METWIAPLAVLFGLSVALLLKSWILRQPSGGAGCSLPPSPRALPLIGHLHLVLGKHLHRAFGEIAREHGPCVFLKFGSSPHLVISSAQAAREAIRVRDSTYSSRPFLSPAARAEEMALAVAKLVEASRGPMPRTVSLTQVISDVTYGMILRKVVGNGHRRNEEALRFKHLLKEVFVAAGEFYVGDTMPWLQWLDLRKAAHAKRLYKQVDEYMQRLVDEQRRKGGDIGDDFISIMLRNELFSKSDSFMKAIVLDMIGAGTDTSAVTIEWAMAELINNPRIMSRLLEELHSVVGPSSLKVEEAHLDKLVYLDAVVKETLRLHPPAAILIFQAAQPCQVMDYFVPEGTRVFINNYEIARDERCWEEPLKFKPERFVERNIDIVGVRDFEMLPFGSGRRGCPGIQLGLRVVHFVLANLVHGFEWKNPSGKELDMSEGSGLTLPRAVPLELTISSRI | |
| CYP791C1v1 | MEFHYWSLSIFSLLAILGTVWCLVRILYPSKRYKGLPCPRMFPVIGHLHLLRRDPHRVLLALAREFGRCMYLKLGQYPCLVLSSAEVTKEALQGHDIAFSSRPALSAARIFGFNGSGVLWAPYGEHLKMVRKLCILELLTPRRVDSFESIRAEERSRFVSQLRDIANRNEAADLTAMLLNMTLNIMMRIVLGTSSATVDKETSTVKELIAEAFVSTGEFLVGDYLPWLSLLDTKKKSRMKALKEQMSSYLQKQIEEHHNQNDKSADFMTLMLQSPEIGSNDVAIKAVIAVQDMIAGGTDTSAITVEWALAELLKHPDLMAKAQEELDNVLGRKSQVQGGHLPKLEYLAAVIKETLRLHPPGPLLIHETTQNCQLKNVFVPQKTLAFINLYALGRDESTWVDPLKFDPNRFIDKKNDGCGHDFGDYLPFGAGRRGCPGMHLALTVVSVTLASLLYGFNWKMPDGMSFEHLDMSEGAGFTIPRALPLKLVPLPR | |
| CYP792A1v1 | MEHITAFFTFLASFILVIFYYDRSKSRSSHVMPPSPRAFPILGHIPLLASNSRGPHLILFDLAKKLGPIFYLRLGYTPTLVISSAKIAQEILKTHDRIFSSRPSLTFAEAILPDDLIFARYGARWRELRKICTLELFTARRVGSFAAVRQAEMEKFLAMLSQNLRRTVNMTQELSVLTLEIMQTLVFGTSRTFGANDFLRLAHQANELGGRLHIGDYIPWLKWMDLSLPKLRTLATKFHALLQAHIEEHRSSIAKQGHGGESFLDVLLSLDNMSDLTIRCLMLDAVSAGLDTTATAIEWALAELLLHPQILAKAQKELDDVIPASSAMVSEADIPKLKYLGAIVKETLRKHPPAPLMVPRESTTDCKVTGYTIPAKTQVLINLYAIARDPNIWENPLEFIPESMSSEFNAAVELMTFGFGRRSCPGMNLGLAAVHLVLANLLYRFNWTTPDGKEVDVGESGSRSCVLVHLHWFHFSASNETY | |
| CYP793A1 | MEVFLLLLVVITFGFFLRTRNRNNILPPGPLAIPFVGHLHLLLKGHPHVVLKALAEKYGPVMFLRFGVVPIVVVSSSQSAVEFLKVQDKVFTSRPRFLSAGRLLLGFDGEDMVFAPYGMRWKQLRRLCTTKLFTARNFADVRMSEVRSLVRAIQAFGEASPNSALDLRTKFKHLTFNIITRMLMSKRYFEGDTADSKEAEEFIYLMEESFSLAGAFPVSDYLPYSFVKWLNMNQDDRIKTLSVRSRQFVDKIITEHELRSPTCSDDFLGLLLKLRSTEDVLQRNTIRGLMINLLQAATDTSSVSLEWTLAELINHPACMSMVQDEIASVVGSNRMVEERDISKLPYLQAIVKESLRLHPPGPLLLPRECSKTCEVMGYKIPEATTLMVNAYAIGRDPKVWKEPLKFKPERFLDYSCFDVGGNNLDVIPFGAGSRACPGISIAFSILHLALANLVHAFHWTLPAEVVHVDTSNEKYGLTVTLAKKLEAIPLCKIDTIVCGDE | |
| CYP793B1 | MSLRFGHVPVVVASSPAAAKEFLKTHDAAFASRPLSAAGRTIVHYNADIVFAPYGDSWRHLRKIATLELLTARRIDMFRGARMEEVRSMCRSLLVADDREMGVVDVRGQVTALTFNLITLMLMGKRYFGKDIENEKGAKKFLEVIASTFKVCGEFPIGDYFPWLPKFLDPAERRMHSLAKSLHEFLSDNIIEHENKRKNKKKNNNDEDFLDILLSLKDNGDEHLQNENIISVMTNLVTAGTDTSAVTLEWAMAESIKNPTIAAKAREEIELVLGEKWRTKMVEEPDLSQLTYLQAIVKETLRLHPAGPLLVPHQSTEAVSNVMGYHVPRGTTVLINAYAIARDSSAWGDDALLFRPERFLGTDLDIRGRDFEAVPFGSGRRQCPGMALALTTVHLTLANLLHGFEWREPSGESIDTSKEQYGLTLLLAKKLRLIATPRLEQGTL | |
| CYP794A1 | MGAFGLLLYLRNKMKKIQGNKQQLPPSPQSLPIIGHLHHFVSSGKEPHQLFQSLAAVHGPIFSLRLGYMNVVVVSDRSTAKQVLKTNDLALASRPKLISVKHALYNFQDVVFSDYTKELREIRKFLAMELLSAKKLDMFTNVKEDELSWLVLTLANASEQLNTFKMRDYLVGLTYNVITRMLMGKRYYGAPPDDKEYEEGVAFKKVVDDAIKIGVAGSIADFFPQLEFLDWKVSQAKKVQRELDKFLQRMLDEHRVPNRGNSQEDFLDMILEASFMSDDRIKATESMTLLHLQDLITGGTDSSSSFLEWTLAELIMHPQVLAKAQEEIDTVVGHGRKVKESDIPRMPYLQAVIKEGFRLHSPVPLLVPHYANQECSINGYTIPCNTTVFVNTYAMGRDPKVWDNPLEFDPERFLSGPHKEVEVLGQNVNFELLPFGSGRRSCPGSALGNSIVHFTLATLLHCYDWKAGDKIDFAESSGAAKIMKFPLCVQPTPRLQIQDMYVTNQYTNPIHM | |
| CYP795A1v1 | MEFLVGFAILLILVVFSSVFYLRVASQSPSLPTPLPIIGHLYLLGKLPHHSLLAIARKYGPLVQLRLGSVPVVIASSPEMAREFLRNQDLTFASRPTLLTTKYILYDSKDMVFAPYGEHWRSMRKLCVVELLTDRRLASSQQARLEELQRLLAKIAKVVETSEPFLLLDLLTEFTFNVITRMVMNKAYFGSGETMEELAATRDFIHMQEQGTILLGEFHIGDYIPFLKWFDSSVAKMKALHKIQDEFPQKVVDQHVLARQSREQTQAHDGDGDFVDTLLSLDSPDPNNQARNIKALIQNLLGAGTDTSITTIQWAMAELLNNPRALEKAQEELRAKFGNARQEIIQEHELKDLPYLHAVIKETFRLHPPAPLLIPHQSTQDTTVAGLAIAKGTRLFVNVYAIGRDPALWKSPDDFLPERFLGSSIDVHGKNFELLPFGSGRRGCPGMALGLITVQLALANLLHRFQWSLAPGVDAHPMAECFGVVTTMEIPLRARASPNKD | |
| CYP796A1v1 | MASLVNATAALQDEGPASNTIRATLTIALVAAVIAWWAIAKSRYGLKNLPPGPRGLPIIGHFHLIGRLPHVSLQQLSAKFGPLMSLRFGFVPVVVVSSPAMAREILKTHDTAFADRPYKIAANFIFYGQRSISWSSYGDHFKKARKLCATELFTARRVTSFTHVIRDELWKLSGELRAASASGEVVKLRRHLRGLSFNLMTRILMKKVYFGPGASTDESALQEAKEFVNIIDSVLTVGGAFAITDFFPGTKWIDWTVPAAKAASDKLNSFLTKVLDEQRPGEVPDFVALTKSYFDGPDQMKYTKALLVDMFLGGSETSSTVVEWAMAELLHYPKVIAKAQEELERVVGRERMIEESDLPKLEYFSALVKEVFRLHPPLTMMVPHTTAQNQKVAGYDIAKNSMIFVNVFAIGRDPSVWSNPLEFNPDRFMGTSFNVHGHDFELLPFGSGKRGCPGLPLGLRNVQLVLSNLLHGFDWSYAGDIEKHQMTEAMAVVNFMEHPINVRASPRLDDATYKTLSINT | |
| CYP796B1v1 | MFWAVHIVVFLLTAFILKQWLSSISLNLPPGPRGLPLIGHFHLLAMGKIPHIALQQLSKRFGPLFHLRLGSVPVFVVSSPEMAKEFLKNHDTEFAYRPRNNVVSIVMDSRSMSFSPYGDYWKKLRKLCATEIFTAKRMSMNTQIIRDELWELSGELLRASKAGQVVGVRPHLRALSFNVMTRILMKKTYFGSKASGDPAIAAEASNFIAMIDEILEVGAAFSITDYFPYLSWLDLVARRAKVAGDKMNGFLQKVLDEQRPGEVPDFVEVTRSHIGNDLVSLRALLMDLLLGGSETSSTVTEWALAELLHHPDWMVKAQQEIESVVGRTRMVEEGDISKLEVLNAIIKESFRLHPPVSLLIPHASVEAQNVAGYDISKNAMLIVNVYAIGRDPRVWSDPLEFQPQRFIGSSIGVNGQDFELLPFGSGKRACPGLHLGLRNVQLVLSNLLHGFEWKFPGSPKDQTMDEAMGNISFMAHTLKAKITPRLNESLYRLT | |
| CYP796C1v1 | MLPAIVLVLTLAFFVTQWMWSKRAIKLPPGPRALPLIGHFHLLGRIPQISLYHLSKKFGPLMYLRLGSAPLIVISSPAMAREFLKTHDAAFARRPPRVAVDILMYKFKSLSYSEGEYHKNIRRMCSMELFTARRVTSFTKIIRDELWDLTAELAKASKAGQPVALRGKLRSLSFNVMTRILMNKTYFGSKASSDDPQAREFVGVIDEVMDAAGAFSIADYFPSVGWLDWSIARCRRAHQRMDAFLDKVLNEQRPGEIPDFVEMTKARVDGPEQAQYLKALLMDLLLGGSETSSTVVEWAMAELLHNPEWMEKLQQEIESVVGRDRMVEESDLAKLELVNAVIKETFRLHPPLSLMVPHTSPEPRLVAGFEIPAKATVLINTYAIGRDSQAWPNDPDKFKPGRFVGSNINVYGHDFELLPFGSGRRGCPGLPLGLRNVQLVLSNLIHGFDWRFRDGATRKLSFDSGPGFINIIADAVVAQVSPRLEQCAFGTLAAS | |
| CYP797A1v1 | MEHFDLALYLGLILLAGALWRQYRSFKVRLPPGPRGLPLIGHLHLLSTLPHRSLQKLSQAHGPLMHLRFGTVPVIVASSPAMAKEVLKTHDLAFASRPYLLVGEYAAYNFHNIGLAPYGDHWKMMRKLCSTELFTAKRIDSFSWVRVEELSGMVSGLLAKSASKEVVQIKSFLTDFTFNVMTRILMDRAFFGPAGADSQGKAREFRGIVEEILQVAGSFNVSEYIPSAFKWIDWNIPRFKRLHARQDRFLQEIIDEHKVGHDALAKPRDFIDILLSYFNHGDSRIDLDNIKAVLSDLLPGGTDTSITTVEWILAELLRNPLALKKAQDELDAVVGKDRMVNESDFPKLHYLHAIIKETFRLHPPIALLVPHMSRYECKVAGYDVPKGATTLVNVYAIGRDPTVWEDPTRFSPDRFLEGAGKGMDVRGQDFELLPFGSGRRSCPGLQLGLKTVELALSNLVHGFDWSFPNGGGGKDASMDEAFGLVNWMATPLRAVVAPRLPPHAYEKV | |
| CYP797B1v1 | MDLTLSVVVSSLLLLFILAVVIISYKTSPPGPWGLPLIGHLHLLARMPLHRALQSMSQKHGPIVSLSLGMRPAILISAPALARELFTSQDVNFPSKPYTSVSEHIGYNFRSIGTAPYGEYYSSIRKLCLTELFTARNIDSFSWIRREELSHLLSAILSRASHGQALDLRKTLSVFTFNSITGALMSKRYLSHDTGAASSKEAMEFKNWLIEVLQLVMEPSLSNFVPWYLRWLDWKTPGLRRLHAKLDKFLQMVVEEHKKSTREQKDFLDILLKAFGEEEAYAKANLLDLMVAGTETSVTGTEWLMAAVIQEPRILKKAQQELHDAVGNRRMVQESDLSKLGYLDAIIKESLRRYPIVPIYIRECQGQASKLGGYDVPKGTIVIVNSWALGMDPVVWENPTQFLPERFLASSIDIKGQDFELLPFGSGRRRCPGMPLGLRTMKLLVANLIHGFDWSVEPGKIQSMEDCFKSTCIMKHPLRPVVTPRLHKDAYTTQIHDFFI | |
| CYP797C1v1 | MELALSSFALPFLLLVLTGALSILVTSWDKKKNLPPSPGWALPLIGHLHLITKQPHRSLQALSKKYGPIMFLKLGMIPSIIVSSPEMAKEALMNNGLAFASRPYLLISEIIGYDFQSIGIHYSEHSRRLRKMCVTELIAPQKLESSLWVRFQELSRAFRILQKSNEEKVAVDMRYLFSTFTFNAFTMILMSKRYFGDTTDDNDQHREIKHVINEIFSLAIKFHITEFVPSYLRWLDPTIPQFKRLHERQDKFMKKIIKEHKEPTARPKDFMDALLESFSAEDTVKAFITVSSLHILLLASDSTAVAAEWVMAQLLHNPHVLEKAQFELNLVVGPNRLVQESDFSKLEYLQAIIKETLRLCPPGPLLIPRSSDEACTIGGYYVPKGSTLFVNAFAIGRDPSIWERPTEFMPERFLGRSVDFKGQHFDLIPFGSGRRMCPGMPLALKALELLLANLVHGFDWSFPPGEIQTLEDCFETTLLLKSPLKLLAVPRHSASVYAEI | |
| CYP797D1v1 | MATIFGGVLVFLVLFFLTKRLSFTRQRLPPSPLSLPLIGHLHLLTRMAHQSLQVLSNKYGPILYLKLGMVPTIVVSSPDMAREILKTHDAKFSSRPYFLVGEYFSYGYCGMGFTSGGEHWKNLRKLCATELFTINRIDSFEWVRKEEISRMISTIENTTGVINMRNLLITYGFNVMTETVMSKRFFCENGALLDADQAREFKKVSIETVEMALKFHISEFVPSYLRWIDWNIPKVKILQAKSDKFMQQIVQEHKRSKNSRKTKDFMDVLLESFTDSSNKQSLKAENTVKALTMELLAGGTDTSASSIEWALMELLLNPHTMVKAREELVKFVDLTNSTVNEGDLPKLTYLNAVIKETMRLHPPAPLLVPHKSTVECKIAGFDIPKGTTTIVNLYAIGRDPNVWENPTKFCPERFLGDSRIDVKGQNFELIPFGSGRRTCPGMILGLRNVQLVLANLIHRFEWALIPGREYGVEETTGTVNWAKTPLEVLKR | |
| CYP797E1v2 | MEFPVYLLVALVVCFLGRSLLQSRKRLPPSPWGLPLIGHVHHLSRLPHQSLQNLSRKLGGIMYLRLGMTPAIVISSPDLAKEALRSNDSSFGFRPYLLVGEYLTYNFKGIGLSNGDHWKNMRKICITELFSVKRMESFRGLRLAEVSHLVSRLAQASKSQSVVNVRELVTDFNFNIHLTVLTFNVQTRILMSKRFFGENLSDDELAEARVFKELIDESVKFAFQFHISEFVPSWLKWIDWNIPQAKRVAAKQDEFLQKIIDEHKAKKSRPTKDFMDILLEQRGDDQEVVKAILMSFAQEILIAGMDTSACTVEWALLELVHNPEVMKKAQEELDVVVGRNRMVTETDFSKLTYLEAVIKETLRLHPPVPILVPHMSNKACVLAGFDVPKGATTIINFYSISRDPNVWEHPTKFWPERFGQITADVKGQDFELIPFGAGRRMCPGMSLGLKTVHLVLSNLLHSFHWERVPGESYNLDEGVGSVTWPKSPLQAQLTPRLRNLDVIFNFAQ | |
| CYP797F1v1 | MEFLVYVLLGSVFLFYLLVRPFLQPRKLLPPSPRGLPFIGHLHLLGRQPHISLQELSNKFGDIVCLRLGLVPAILISSSAAAREALKTHDQTFSGRPYFLLGDYVYSSKSMVLSPPNEHWRRMKKLFNAELFTANRLASFLEVRREELASMVSFLIDNQSRVVNVRELVRSYTFNTITRIVMSKRFFGEKNTVNEEEAMEFMEVMEEIIKFGFAFHISELVPAWLRWIDWKIPAVKRIAAREDIVIQKILDEHRKTKSSRGTKDFLDILLEHDTKGDGGGNDLDNARGTIMELVGAGTYTTACVIEWAILELLRNPDVLEKAQHELESIVGQTNRLVEESDIEHLTYLQAIVKETFRLHPPAPLLLRMSTQECVISNYHIPKGANTFVNVYAIGRDPGLWENPMEFWPERFVGSSMDVRGQDFELIPFGAGRRTCAGLTLGLKVVQVGLANLLHGFDWSCVAGRDYNVAESSVSVIWPKKPLEAIVILKSR | |
| CYP797G1v1 | MDLVTAFIVLLVILLPILRGLFQRSRLPPSPSFGLPLIGHLHLLGRMPHQSLQALAKKHGSILFLRLGIIPAVVVSSVDLAKEVLKNQDLTFASRPYFLVGEDVGYHFMGMSLAGYGDHWKKLRRLYTLELFTAKRIDSFLSLRLEELSHMLSAVLYAHEKNQAVNMRNLLTCFTFNTITRILMNKRYFQHQGEELQGIDSSEASVFKAVLSEITEISLQFHISEFVPAYLRWMDLSVYHMRRLHADQDKFLQKIVDEHKYEKNKSSKDFMDLMLELFDGDPKGDNMIKAALQELVSAGTETSATTVEWTFGEILHRAPHVLTKAHEELDSVVGRSRLVDEADLPRLPYLQAIIKEAFRLHVPVPLLVPHMSMHEASLDGYHVPKGATTIVNAYAIGRDPALWDNPLEFRPERFLGSSMDVKGQDFELLPFGSGRRACPGMGLGLKTVQLALANLIHGFDWKASGQNALEEAAGAVIWLKTPLEAVGSPRLQVEVLTSCHI | |
| CYP797H1Pv2 | MEVAQYAVVLLITLLGLPLIGHLHLLGRILHLSFQTLSTKYGPIVFLRLGMVPAVVISSLELVKEVLKIQDANFALGPYLTMGEYNYNFRDIGFVLYCDYWKSMRKLCATELFTVKRIESFQGVRTRETHGVLSELVNAADYQKPINMYVFHVVMQILMSKPFFEYREHEAEMSSKGKDFKHIVFEITEQMLQFHISEFVPAFMRRIDWKIPEMMRIHARQDKFFKRIIDDHKARLESETSQPKDFMDTMLQSLKSEGARGEEELLSGRDTSASLIEWTLLELMHNPLVLQTVVGKERLVAERGFDKLEYLACKVAGYDIPKGTPTFVICFTIGRDPAVWEDALRFKPERFLGNLIDIKGQDFGL | |
| CYP798A1 | MDNGMMVWIVLAGVVAMAVWYLLVQYQQPKQSHNVPRETLPPGSVGWPFLGEIISFYFRTPDFVKQRRGRYGNLFRTVLIGYPTVISTDPEVNKFILNNDGRLFVPAYPSYWSQIIGECNIFVARGDFHKRMRGAFLHFISISVVKNRLLSEIQNIITFSLAGWEGRNVNVLHEAEEMIFSVMANHMLSLSAGTALESMKRDFLVMMKGLRSLPLRVPGTTFYKSLQKKQVLFNQIKSIIEERKLNMSAYDSYDDLLSSILKSASEKEFTTTQIVDLIVQSVIGSLETTPKIMASVVRHLSENPHIIKYLKEEHETIIQAKENNQSLSWDDYKSMVFTKSVIKETLRFGMQPLNNIMFKKTLQDVKIEGYTIPKGWTCIIYDLVSDMDNKYCKDPLSFNPQRWQSKEMNEVPFLAFGGGPRLCPGYELAMLTMSFFLHHLVTKFRWEYLPSKSELRWFDSPLNSVFDCRIH | |
| CYP799A1 | AVQYLTQKPKALQQLRREHEAILLAKKQKPEKILTWEDYKSMEFTRSVIKETLRLSNVGPFLFRECVQDTEIKGYRIPKGWKVVACTTAVSHDPAIFPEPSCFNPWRWQEDMAEEKHLQVFGGGSRYCVGAELAKLEMAVFLHHLVTKFSWDICEGEIIRSPLVLFKDGYPISVRKRAS | |
| CYP79A2 | MLDSTPMLAFIIGLLLLALTMKRKEKKKTMLISPTRNLSLPPGPKSWPLIGNLPEILGRNKPVFRWIHSLMKELNTDIACIRLANTHVIPVTSPRIAREILKKQDSVFATRPLTMGTEYCSRGYLTVAVEPQGEQWKKMRRVVASHVTSKKSFQMMLQKRTEEADNLVRYINNRSVKNRGNAFVVIDLRLAVRQYSGNVARKMMFGIRHFGKGSEDGSGPGLEEIEHVESLFTVLTHLYAFALSDYVPWLRFLDLEGHEKVVSNAMRNVSKYNDPFVDERLMQWRNGKMKEPQDFLDMFIIAKDTDGKPTLSDEEIKAQVTELMLATVDNPSNAAEWGMAEMINEPSIMQKAVEEIDRVVGKDRLVIESDLPNLNYVKACVKEAFRLHPVAPFNLPHMSTTDTVVDGYFIPKGSHVLISRMGIGRNPSVWDKPHKFDPERHLSTNTCVDLNESDLNIISFSAGRRGCMGVDIGSAMTYMLLARLIQGFTWLPVPGKNKIDISESKNDLFMAKPLYAVATPRLAPHVYPT | |
| CYP79B2 | MNTFTSNSSDLTTTATETSSFSTLYLLSTLQAFVAITLVMLLKKLMTDPNKKKPYLPPGPTGWPIIGMIPTMLKSRPVFRWLHSIMKQLNTEIACVKLGNTHVITVTCPKIAREILKQQDALFASRPLTYAQKILSNGYKTCVITPFGDQFKKMRKVVMTELVCPARHRWLHQKRSEENDHLTAWVYNMVKNSGSVDFRFMTRHYCGNAIKKLMFGTRTFSKNTAPDGGPTVEDVEHMEAMFEALGFTFAFCISDYLPMLTGLDLNGHEKIMRESSAIMDKYHDPIIDERIKMWREGKRTQIEDFLDIFISIKDEQGNPLLTADEIKPTIKELVMAAPDNPSNAVEWAMAEMVNKPEILRKAMEEIDRVVGKERLVQESDIPKLNYVKAILREAFRLHPVAAFNLPHVALSDTTVAGYHIPKGSQVLLSRYGLGRNPKVWADPLCFKPERHLNECSEVTLTENDLRFISFSTGKRGCAAPALGTALTTMMLARLLQGFTWKLPENETRVELMESSHDMFLAKPLVMVGDLRLPEHLYPTVK | |
| CYP79C1 | MDYYLNNVIFSVVVTFSITLNIVFLIKSVVARFLGRRKKLPPCPRGFPIIGNLVGMLKNRPTSKWIVRVMNDMKTDIACFRFGRVHVIVITSDVIAREVVREKDAVFADRPDSYSAEYISGGYNGVVFDEYGERQMKMKKVMSSELMSTKALNLLLKVRNLESDNLLAYVHNLYNKDESKTKHGAVVNVRDIVCTHTHNVKMRLLFGRRHFKETTMDGSLGLMEKEHFDAIFAALDCFFSFYVADYYPFLRGWNLQGEEAELREAVDVIARYNKMIIDEKIELWRGQNKDYNRAETKNDVPMIKDWLDILFTLKDENGKPLLTPQEITHLSDLDVVGIDNAVNVIEWTLAEMLNQREILEKAVEEIDMVVGKERLVQESDVPNLNYVKACCRETLRLHPTNPFLVPHMARHDTTLAGYFIPKGSHILVSRPGVGRNPKTWDEPLIYRPERHITGNEVVLTEPDLRLVSFGTGRRGCVGAKLGTSMIVTLLGRLLQGFDWTIPPGTTDRVELVESKENLFMANPLMACVKPRLDPNMYPKLWTGPA | |
| CYP79D1 | MAMNVSTTIGLLNATSFASSSSINTVKILFVTLFISIVSTIVKLQKSAANKEGSKKLPLPPGPTPWPLIGNIPEMIRYRPTFRWIHQLMKDMNTDICLIRFGRTNFVPISCPVLAREILKKNDAIFSNRPKTLSAKSMSGGYLTTIVVPYNDQWKKMRKILTSEIISPARHKWLHDKRAEEADNLVFYIHNQFKANKNVNLRTATRHYGGNVIRKMVFSKRYFGKGMPDGGPGPEEIEHIDAVFTALKYLYGFCISDFLPFLLGLDLDGQEKFVLDANKTIRDYQNPLIDERIQQWKSGERKEMEDLLDVFITLKDSDGNPLLTPDEIKNQIAEIMIATVDNPSNAIEWAMGEMLNQPEILKKATEELDRVVGKDRLVQESDIPNLDYVKACAREAFRLHPVAHFNVPHVAMEDTVIGDYFIPKGSWAVLSRYGLGRNPKTWSDPLKYDPERHMNEGEVVLTEHELRFVTFSTGRRGCVASLLGSCMTTMLLARMLQCFTWTPPANVSKIDLAETLDELTPATPISAFAKPRLAPHLYPTSP | |
| CYP79E1 | MELITILPSVLPNIHSTATVLFLLLLTTALSFLFLFKQHLTKLTKSKSKSTTLPPGPRPWPIVGSLVSMYMNRPSFRWILAQMEGRRIGCIRLGGVHVVPVNCPEIAREFLKVHDADFASRPVTVVTRYSSRGFRSIAVVPLGEQWKKMRRVVASEIINAKRLQWQLGLRTEEADNIMRYITYQCNTSGDTNGAIIDVRFALRHYCANVIRRMLFGKRYFGSGGEGGGPGKEEIEHVDATFDVLGLIYAFNAADYVSWLKFLDLHGQEKKVKKAIDVVNKYHDSVIESRRERKVEGREDKDPEDLLDVLLSLKDSNGKPLLDVEEIKAQIADLTYATVDNPSNAVEWALAEMLNNPDILQKATDEVDQVVGRHRLVQESDFPNLPYIRACAREALRLHPVAAFNLPHVSLRDTHVAGFFIPKGSHVLLSRVGLGRNPKVWDNPLRFDPDRHLHGGPTAKVELAEPELRFVSFTTGRRGCMGGPLGTAMTYMLLARFVQGFTWGLRPAVEKVELEEEKCSMFLGKPLRALAKPRQELLQSF | |
| CYP79F1 | MMSFTTSLPYPFHILLVFILSMASITLLGRILSRPTKTKDRSCQLPPGPPGWPILGNLPELFMTRPRSKYFRLAMKELKTDIACFNFAGIRAITINSDEIAREAFRERDADLADRPQLFIMETIGDNYKSMGISPYGEQFMKMKRVITTEIMSVKTLKMLEAARTIEADNLIAYVHSMYQRSETVDVRELSRVYGYAVTMRMLFGRRHVTKENVFSDDGRLGNAEKHHLEVIFNTLNCLPSFSPADYVERWLRGWNVDGQEKRVTENCNIVRSYNNPIIDERVQLWREEGGKAAVEDWLDTFITLKDQNGKYLVTPDEIKAQCVEFCIAAIDNPANNMEWTLGEMLKNPEILRKALKELDEVVGRDRLVQESDIPNLNYLKACCRETFRIHPSAHYVPSHLARQDTTLGGYFIPKGSHIHVCRPGLGRNPKIWKDPLVYKPERHLQGDGITKEVTLVETEMRFVSFSTGRRGCIGVKVGTIMMVMLLARFLQGFNWKLHQDFGPLSLEEDDASLLMAKPLHLSVEPRLAPNLYPKFRP | |
| CYP800A1 | MGGAVRRTRAGRFGAASATETAKAEDDEVASAEVGVSVGASAMGGAEDEASGTACPYTAAKEALGLAPPPPTRRDENGNPAMVDNVSFLKSLIKASQHPVGMPIAMLDWAAEKGECVGIKNAIGPFCVSILDPEIVEYVCFTNAKNYRLRMLPDAFRYVIQNKGITGSDGQYNRDHRLMCQKPFINSFSLAEFSSTVEERIAHMCNTWQQAHAMGGGKPYEINIDYDSQQVTLDVIGKVAFAYDFKRCEAHEAKTLRGEADEDGNVSKLLAAYNGSSEIMGELFITPGPILKLQNFLGLGRVRELKEQYAILESVGTKLMSERRAIVKERLAAGDEEDYCLLDVLVKAKDADGQPLSDADLWGDINDIMAAGHRTTASNFTVNLWHVARYEHIQEQIEKEVAALGGRPPTFQDVQEGKLPYTQRVVKESLRKYAPINLFPRLAEGPDTLPSGHKVEEGDFILLSTYAMGRNPRVWEDPNKFDPDRFTDEALYAQAEKQASAVARGDPAKLEQARERMRRRMNAGRDFTYTPFGAGPRSCIGGVFALLAATTMLASTVQKFKLSKASHSKGAVGEELNIMYDTTICFPEGVWINLEPRERPLGA | |
| CYP801A1 | MRSSEVIGNLVNAFDVASNASTIAAVALLGIFTLGLFFILFVRARARATLARASVPAVTWRPKFLWRLYSRSARSLLQRVEQRFRRGDGSGRGRRAFGAVVGACPFVHVGEARLARDVLRETSRKAPLYHAFEAFSGTGIFTAEGDDWDGKRTEVLRAFHDVGLASLRDRAVEESASAVEEMREVVERGGGEIEARALPILQRLALRVTFAYLTGVSLRRACEDVGRERSEVEGEYLDAATTLRHLIPARARSIWIFSDLLYGLTPVGRLEARKIRTTRSLSALALRTAKEESPLGRLRVGEAHLREKSIKVGKDEYPKGLLDEATTLLFAGHDTQSATLSWCLLRLVQDVEVQSELRASLRDDIIEEALGLPPSKSGSRQRRSSEKTTKPAWATSAFAPTLEAVIRETLRLHPVAPLVVRMLSSDTHSEKMTIPKGCAVGVWLSSVHRDESVWERPEEFDPKRWFGGPAHARTGSMGGSSNDDDDNGGILTPSRDRSNALRHKGVGYMPFAYGPRSCVGQHLAQVTMRVALAHLVHAFEFAPSADLDASMPSVGFTVTPSTGAPVRVRLAARAPA | |
| CYP802A1 | MASRATARAAASHDARAATRRARVGGAARESAARRRASGRATSDVDGASGRATSDVDGASGRATSADAFDLGQAIGTLARGERGATLPPGRVGALGVRETLEYLADSNGFVRRRVERYGPIFKTALFFKPAIVFGSREAVREFLKFEGELPADEALPETFRELHTEYGALRMTGSRHAATRANFGKVLGRAALESYAPAIGERTREFVEDVARRSGKETSSFRPGAECVDFALDLLFELFLGHVPEAKYKDAMKAYNGGLLSLGKWSSEFKAGKLALEDLTSYVEAHYRGVKARGELDRPEYFFYKQYSQAVDEFDEVFSDDRIATTCVLMVWGSYIEAAALMGHACVLLGEHDDARRAVLREFERVCCDDENGCRRIGTLADIMSMQYTSAVAKESLRVMPQTAGGLRVNPSPRKFASFDVPAGYVLTADPRIPFRDEANFPDPDAFKPERFVPGTHEAKQNDVSSETYYPGGMGQHQCPGISLATVMTQIFLAELVSAFPNGWRGKTAPKYVQVPIVILDREYEIEFLR | |
| CYP803A1 | MSALVANAVGPVAVARRPPRRMRHAPRERVSAPRASRADDLPGILLGAAAKKLEEDVNSFIGLFDEDAPTHERPPTLPVAGNTLDIAQGGHRQLLEWAETYGVADGVHEVKMLSQTILHLTDPKLARELMFERSDSFPDRGVSAMAKFFREDQAAFVNTSGEQWMAYRKMGTATVNGGALDRLAGKVAERSEALVTRWVRDASASGDGRNATEVDISDASQAVTLEVIHEALFSEQLDVIDGERNAVALARSFREFNVANQDLLNDFLTLYQRFETPERARRDTHRRRLRAHFDERADARRAAIARDGAAAAPRDLLTALLTARDPATGAALTRDDVNLTLTEMMVAGHDTTAATVACMMCLLASHPEVRASVTGEVDEFRRNNGGRLPSSVADANALVKLDDAMKETMRLYPAVLIVVRKAEEGTGGVFTKGPGREVRIPEGSGMWVSPYVLGRLARHWGGDEEDVKRFRPSRFEEARERGDSLDAYMPFGGGPRVCLGSRFAMLEGKVLAAHILADWDVELAAETRDAIARNDGELPIAYAAGLMSFPEPLRLRVRRRGAASGPR | |
| CYP804A1 | MGGYFTSDRAAKYGWGVPRLALVTLFAFEIVPRAFLAGSRAPVDALVSTPLPFTARAAAAPFAPLTLVPYLHSLRWIAHAYTGSIGAGVNVQVLGLLGNLAIMAVAGGLCLWDLTKDISLVVYFLKTFCGDQLAGKLDTAEWKLVLAFTFALPLAMWGAAGANGAAFAMFLPYARIAPILFAIQAVCEFGDAHLEYHPVIGKFFRHRYGFEAFTLCALTMMPGGITAPELRVVQFDLVICLFYRVANLGIILHKQGFAVAAAASLAVTIRKGCFRVFGALSGQRIVNVTDAEVATAVMRASDVKGDALERHVATPAWRPLLSLESVDHELYRNMLRDFHAVVKACPPPQRVGEIARAKVDELMYRTYSEEAEEASPVRGGAEPSPPPSPERPVVDVTLADSPVDSPHPHGGKGGDVGKCPFVQMQRTMRGGASGQSNAAGRSTPARSDAAPVIDADDVARLSLSVFIEYLFGREWEPKFETLLAASWEWRKEIAVRGRADPGVKKAAVELVVDDLIKNSHLWDLFGEKWREPRYYSLIMQPFLVSPAINVGDIAVAMKAHPDLALEPAMRRMHPFPIFERWVDKDVVVDGRIAVRADTQVIMFTSDFANSKHLWPAFGTGPRACAGTSMALGVLNAIHQKMLGRPGFEPERGHKFSGRNNDGVTSLSEVWYFAKTVLPVVFGFGGEKTTEAAALERAAAAALE | |
| CYP80A1 | MDYIVGFVSISLVALLYFLLFKPKHTNLPPSPPAWPIVGHLPDLISKNSPPFLDYMSNIAQKYGPLIHLKFGLHSSIFASTKEAAMEVLQTNDKVLSGRQPLPCFRIKPHIDYSILWSDSNSYWKKGRKILHTEIFSQKMLQAQEKNRERVAGNLVNFIMTKVGDVVELRSWLFGCALNVLGHVVFSKDVFEYSDQSDEVGMDKLIHGMLMTGGDFDVASYFPVLARFDLHGLKRKMDEQFKLLIKIWEGEVLARRANRNPEPKDMLDVLIANDFNEHQINAMFMETFGPGSDTNSNIIEWALAQLIKNPDKLAKLREELDRVVGRSSTVKESHFSELPYLQACVKETMRLYPPISIMIPHRCMETCQVMGYTIPKGMDVHVNAHAIGRDPKDWKDPLKFQPERFLDSDIEYNGKQFQFIPFGSGRRICPGRPLAVRIIPLVLASLVHAFGWELPDGVPNEKLDMEELFTLSLCMAKPLRVIPKVRI | |
| CYP80B2 | MEVLSIAIVSFSFLLFLFFILRDSRPKNLPPGPRPSPIVGNLLQLGDKPHAEFAKLAQKYGELFSLKLGSQTVVVASSPAAAAEILKTHDKILSGRYVFQSFRVKEHVENSIVWSECNDNWKLLRKVCRTELFTPKMIESQSEIREAKAREMVKFLRGKEGEVVKIVEVVFGTLVNIFGNLIFSKDVFDLEDPTGGSVELKEHLWKLLDMGNSTNPADYFPIMGKLDLFGQRRAVAEVLQQIYDVWGVMLKERRGTKGSESKNDFVDVLLNAGLDDQKINALLMELFGAGTETSASTIEWAITELTKKPLVVSKIRLELVNVVGDNTVKESDLPHLPYLQAFVKETLRLHPPTPLLLPRRALETCTVMNYTIPKECQIMVNAWAIGRDPKTWDDPLNFKPERFLSSDVDYKGNDFELIPFGGGRRICPGLPLASQFSNLIVATLVQNFEWSLPQGMSTSELSMDEKFGLTLQKDPPLLIVLKARASNI | |
| CYP80C1 | MDQRFLQRLFSLVSSAEILFLLLLPLTFIILKNIIRSCSESKYLPPGPKPWPIIGNLLHVGNQPHVSLAEIAKIHGPLISLRLGTQLLVVGSSAKAAAEILKTHDRFLSARHVPQVIPRESHVLRRVALVWCPESIDTWKLLRGLCRTELFSAKAIESSATLREKKVGELMDFLVAREGKVVSIGEVVFSTVFNTISNLLFSNDLAGLEEKGMSSGLKSHVRKLMLLVATPNIADFYPIFAGLDPQGLRRKLSKLVEETFAIWAINIKERRNSYVHDSPKRDFLDVFLANGFDDDQINWLAAELFSAGTDTTATTIEWAVAEILKNKEVMKKVDEELEREITKNTISESDVSGLPYLNACIKETLRLHPPVPLLVPHRATETCEVMKYTIPKDSQVLVNVWAISRDPSTWEDPLSFKPDRFLGSNLEFKGGNYEFLPFGAGRRICPGLPMANKLVPLILASLIRCFDWSLPNGEDLAKLDMKDKFGVVLQKEQPLVLVPKRRL | |
| CYP80D1 | MVSISVLANSYPSFPMLFLLAILLLLSLVLKHKSSKVPAIPPGPKSWPIIGNVLQMGNKPHISLTKLAQVYGPLMSLRLGTQLVVVGSSREAASEILKTHDRELSGRCVPHASFAKDPKLNEDSIAWTFECTDRWRFFRSLMRNELFSSKVVDGQSRTRETKAREMIDFLKKKEGEGVKIRDIVFVYTFNVLANIYLSKDLIDYDQTGECQRVCGLVREMMELHTTLNISDLYPILGSLDLQGVSRKCNECESRIQELWGSVIKERREGRNDTGDDDDNSSKRKDFLDVLLDGEFSDEQISLFFVQELLAAVSDSTSSTVEWAMAELMRNPQAMKQLREELAGETPEDLITESSLAKFPYLHLCVKETLRLHPPAPFLIPHRATEDCQVLDCTIPKDTQVLVNVWAIARDPASWEDPLCFKPERFLNSDLDYKGNHFEFLPFGSGRRICAGLPMAVKKVQLALANLIHGFDWSLPNNMLPDELNMDEKYGITLMKEQPLKLIPKLRK | |
| CYP80E1 | MATIVTEISSNTLFTILFLLPLIYLIAKQLKALYSSRFAPLPPGPYSWPILGNALQIGNSPHITLASLAKTYGPLFSLRLGSQLVIVAASQEAATEILKTQDRFLSGRFVPDVIPAKWLKLENLSLGWIGEVNNEFKFLRTVCQSKLFSNKALLSQSCLREKKAADTVRFIRTMEGKVLKIKKVAFAAVFSMLTNILISSDLISMEQESTEGEMTEIIRNIFEVGAAPNISDLFPILAPFDLQNLRKKSKELYLRFSTMFEAIIEERRERKMSSDNASGKEDFLDTLISNGSSNEHINVLLLELLVAGSDTSTSAIEWAMAELLRNPQCMKKAQAELASEINQDLIQESDLPRLKFLHACLKESMRLHPPGPLLLPHRAVNSCKVMGYTIPKNSQVLVNAYAIGRDPKSWKDPLDYKPERFLTSNMDFRGSNIEFIPFGAGRRACPGQPMATKHVPLVLASLLHFFDWSLPTGHDPKDIDMSDKFHTSLQKKQPLLLIPKIKN | |
| CYP80F1 | MYIEDTSEIFTIFFTHILLPLLSFFIIRCVISSRKKLPLPPGPFPWPIIGHLFYLGNKPHVSLAKLANVHGPHLMSIRLGGRLVIVASSPMATAEVLKTHDRLLSGRFVSHPMRVEGSYIRNLATETLEECDENWKKVRSMYQIVLFSHKAVESQVNIREKKVMELVKFVASKEGELVNIKGIAFVTILNILSNSTISNDLVDFEGKGIGEGMREWIRNYTKLEGVPQLADLFPILDGCTWDFQGTYKKLKDTFERISDVWRDIINKKRMEISNKYYEGEDFADALIRNGFEDKQINALLMELYSAGTETTITTVEWTLVELLKNPEAMKRLRNEIKKELTTIDDREIMIVKDSNLPNLPYLEACMKETLRLHPPAPLLFPHRAVQTCEVMGYRIPQDTQIIVNVWKMARDSEYWNDDPWSFKPDRFLDSSTDYKGHDFEFIPFGSGRRICAGQSLALRMLPMIVGSLVHNFELILPNNMNPMEMNMDDIIDVTMAKKDPLFIIPKIRNS | |
| CYP80G1 | MDLQVALFSLIPIILVCILFFKSKHKNLPPGPHAWPLIGSLPVLFTNTEVPLHITLTNMARTHGPMMILWLGTQPTLVASTAEAAMEILKTKDRVCSGRHIRMSFRLKHHIKYSLVWSDCTDYWKLLRKIARTEIFSPKMLQAQSHVREQKVGELVEYLRSKEGQVVKLTQFVFGTLLNILGNVVFSRDVFVYSDDDGNKDGIQNLIREMLMIGAEPNIAEFYPILEELDLQGLKRKCDERFLRVMKLWKGTVNERKEKRNEETKDMLDVLLANDFNDAQINALFLETFGPGSETSSATIEWVMSELIKNPKEMAKVRKELDEVVGTSTVKESHLPQLPYLQACIKETMRLHPAAPFLLPRRAIEACELMGYTIPKDCQILVNAYAIGRDPNSWTDPLTFRPERFFESDVDYHGGHYQFIPFGSGRRTCVGMPLATRTIPLIVGSLVHTYDWGLPDGKRPEELELKEMLSLSLAIDPSLCVVPKMRV | |
| CYP80G2 | MDLQIALFSLIPVILVFILLLKPKYKNLPPGPHPWPLIGNLPILFTNTEVPLHITLANMARTHGPIMILWLGTQPTVMASTAEAAMEILKTHDRIFSARHIRMSFRLKHHIKYSLVWSDCTDYWKLLRKIVRTEIFSPKMLQAQSHVREQKVAELIDFLRSKEGQVVKISQFVFGTLLNILGNVVFSKDVFVYGDETDKGGIQNLIREMLMIGAEPNVAEFYPSLEELDLQGLKKKCDERFIRVMKMWEGTVKERKANRNEESKDMLDVLLANDFNDAQINALFLETFGPGSETSSATIEWVIAELIKSPKEMAKVRKELNEVVGTSTIKESDLPQLPYLQACIKEAMRLHPAAPFLLPRRAAETCEVMGYTIPKNSQVLVNAYAIGRDPKSWKDPSTFWPERFLESDVDFHGAHYQFIPFGSGRRTCVGMPLATRTIPLIVGSLVHNYDFGLPGGNRPEDLKMNEMLSLTLAIDPSLCVVPKARA | |
| CYP80H1 | MEQSNLFIFILLLILFVLFLVLHHMRQKFSKYPPGPYPLPILGNFLHLRKTPHISLANLAKVHGPLISLRLGAKLLVVASSQEAATEILKIHERVMSGRPVPLTIEAVSKGLDFFTLIGATSCTKNWKILRTIVKAELLSTEVVDKTTAAREEKVKELINLLSSKQGKVVNLGDYVFATAANTVSRLLFSKDCISLENQGMVGGFSKENIKTIVKMASTPNLGDHYAIFNGLDIQGLGKKSVECLGRLYASWNSLIDGRHVSKCRDHVGKDKDFLDVLLANGFSKDQINLILSEMFITGIDTTSTAVEWAMAELIKNQDAMTKLSEELAKEIGGNIIRDSDLPRLPYLNACVKETLRLHPSVAMIPRRAVETCQVMNYTIPKNAEIWVNLWALGRDPTKWEDPLAFKPSRFLQSHLGFMGSHFEYIPFGSGRRMCPGLPLAVRLVPLVLASLVHSFDWYLPENLSPGELNMDAKLGLTLQREKPLYLIPRPK | |
| CYP80J1 | MQSLNLESILNNADSSATLPLLLLLFSIVFLVAIKQKSSRKLPLPPGPRPWPIVGNLPQLGKKMHVSMADLAKTHGGLMSLRLGTQLVIVGSSPEAATEILKTHDKELCGRHVPMVSFATDPKLNKDSIAWSYECGKEWMAFRALMRTELFSTKIVETQYHVREKNVRAMVDYLSRKQGELVAMRDVVYIYTFNTLGNVYFTRDFMDFDGEEGRRVSALVREMMELWSAPNISDLYPILSRFDLQGLRKKADVCVKKMCDLWDHSIRERREIRGKSRHDSSDLAPKNKDFLDILLESGFDDEQISYIFLELLAAVSDGSTSTVEWALSEMVKNPEAMKKARQELSEQISESFVTESQLSKLPYLHACIKETLRLHPPAPVLLPRRAAQDLEIMNHTIPKNAQVLVNVWAIARDEKIWKDPMSFKPERFINSSVDYKGNDFEYLPFGAGRRICAGLPMATRQVLLALANLIHQFEWSLPDNLRPEQLDMEELFGVTLLKENPLALIPKRKF | |
| CYP80K1 | MDPDTVTADISIFSFLYALLLLPFLVILKHIFLKPPPLPPGPYPWPIIGNLLQMGKNPHAKLANLAKLHGPLMSLRLGTQLMVVASSPAAAMEVLKTHDRALSGRYLSXSVPVKNPKLNHLSIVFAKDCNTNWKNLRAICRMELFSGKAMESQVELRERKVTELVEFLATKEGEVVKVMDLVFTTICNILSNKFFSMDLCDFEDEGRVGGALKDLIHKNAEFGATPNLSDYYPILGGLDIQGINRKAKEMFERIPTTWEDILKERRTQRSNRSSHRDFLEALLEIGFEDDQINQVILELFSAGAETSSLTVEWAMAELIRNQDAMDKLRGELRQIVGESPVRESHLPRLPYLQACVKEALRLHPPAPLLLPHLAAETCQVMGYTIPKDSQIFVNIWAMARDPKIWDDPLSFKPERFLDSKLDFKGNDFEYIPFGAGRRICPGLALGGRQVPLILATFVHLFGWSLPGNMDSAQLDMEEWLVITLRKEQPLRLVPRVRK | |
| CYP81A1 | MERFYYVAVATFVLVFLLHHLLTRKKQQRLPPGPRFAYPILGHLPLLKKPLQTSFADLVSRHGPIIHLRLGRRHAVVVGSAAVAKECFSGELDVAIANRPHFPSAREVTFDYSVLTAVNYGALWRTMRRVSTVHLLSAHRVNVMSDTVIARELRVMVRRLARASASAPGDAARVELKRRLFDLSHSVLMEIMAQTRNTYSDDPREEMSREARDMKDIIEEIIPLVGAANLWNYVPLLRWLDLYGAKRKLADVVNRRDLIFDNMIGAERQKLRQLERKKGEAHASESDKMGMIGVMLSLQKTEPDVYTDTFINALVSNLLAAGTETTSTTLEWAMSLLLNHPDVLKRAQEEIESNVGRDRLLDKNDLPRLPYLHCIISETLRLYPPTPMLLPHEASTDCKIHGYDVPAGSMVLVNAYAIHRDPAMWEDPEEFRPERFELGRAEGKFMMPFGMGRGRCPGENLAMRTMGLVLGALLQCFDWTRVGDREVDMATATGTIMSKAVPLEAQCKPRANMSAVLQKI | |
| CYP81B1v1 | MEIPYLLTTTLLLLFTTLYLLLRRRSSTLPPTIFPSLPIIGHLYLLKPPLYRTLAKLSAKHGQILRLQLGFRRVLIVSSPSAAEECFTKNDIVFANRPKMLFGKIIGVNYTSLAWSPYGDNWRNLRRIASIEILSIHRLNEFHDIRVEETRLLIQKLLSACNSGSSQVTMKFSFYELTLNVMMRMISGKRYFGGDNPELEEEGKRFRDMLDETFVLAGASNVGDYLPVLSWLGVKGLEKKLIKLQEKRDVFFQGLIDQLRKSKGTEDVNKKKTMIELLLSLQETEPEYYTDAMIRSFVLVLLAAGSDTSAGTMEWVMSLLLNHPQVLKKAQNEIDSVIGKNCLVDESDIPNLPYLRCIINETLRLYPAGPLLVPHEASSDCVVGGYNVPRGTILIVNQWAIHHDPKVWDEPETFKPERFEGLEGTRDGFKLLPFGSGRRSCPGEGLAVRMLGMTLGSIIQCFDWERTSEELVDMTEGPGLTMPKAIPLVAKCKPRVEMTNLLSEL | |
| CYP81C3 | MEFLYYHLALLFFLFIVVKNLFHRKRNLPPAPFALPVIGHLYLLKQPLYKSLHALLSRYGPALSLRFGSRFVIVVSSPSVVEECFTKNDKIFANRPKSMAGDRLTYNYSAFVWAPYGDLWRKLRRLAVAEIFSSKSLRKSSTVREEEVSCLIRRLLKVSTSGTQNVELRLLFSILASNVVMIVSAGKRCVEEEHAGTKMEKQLFQDFKDKFFPSLAMNICDFIPILRVIGFKGLEKNMKKLHGIRDEFLQNLIDEIRLKLKKTTSLKTDEVTDGEERRSVAEILLCLQESEPEFYTDEVIKSTVLMMFIAGTETSAITLEWAMTLLLNHPKVMQKVKAEIDEHVGHGRLLNESDIVKLPYLRCVINETLRLYPPAPLLLPHFSSEACTAGGFDIPQGTMLVVNAWTMHRDPKLWEEPNEFKPERFEAGLGEGDGFKYIPFGIGRRVCPGASMGLQIVSLALGVLVQCFEWDKVGTVEDTSHGLGMILSKAKPLEALCSPRRDLITLLSHL | |
| CYP81D1 | MEETNIRVVLYSIFSLIFLIISFKFLKPKKQNLPPSPPGWLPIIGHLRLLKPPIHRTLRSFSETLDHNDGGGVMSLRLGSRLVYVVSSHKVAAEECFGKNDVVLANRPQVIIGKHVGYNNANMIAAPYGDHWRNLRRLCTIEIFSTHRLNCFLYVRTDEVRRLISRLSRLAGTKKTVVELKPMLMDLTFNNIMRMMTGKRYYGEETTDEEEAKRVRKLVADVGANTSSGNAVDYVPILRLFSSYENRVKKLGEETDKFLQGLIDDKRGQQETGTTMIDHLLVLQKSDIEYYTDQIIKGIILIMVIAGTNTSAVTLEWALSNLLNHPDVISKARDEIDNRVGLDRLIEEADLSELPYLKNIVLETLRLHPATPLLVPHMASEDCKIGSYDMPRGTTLLVNAWAIHRDPNTWDDPDSFKPERFEKEEEAQKLLAFGLGRRACPGSGLAQRIVGLALGSLIQCFEWERVGNVEVDMKEGVGNTVPKAIPLKAICKARPFLHKIIS | |
| CYP81D1 | MEETNIRVVLYSIFSLIFLIISFKFLKPKKQNLPPSPPGWLPIIGHLRLLKPPIHRTLRSFSETLDHNDGGGVMSLRLGSRLVYVVSSHKVAAEECFGKNDVVLANRPQVIIGKHVGYNNANMIAAPYGDHWRNLRRLCTIEIFSTHRLNCFLYVRTDEVRRLISRLSRLAGTKKTVVELKPMLMDLTFNNIMRMMTGKRYYGEETTDEEEAKRVRKLVADVGANTSSGNAVDYVPILRLFSSYENRVKKLGEETDKFLQGLIDDKRGQQETGTTMIDHLLVLQKSDIEYYTDQIIKGIILIMVIAGTNTSAVTLEWALSNLLNHPDVISKARDEIDNRVGLDRLIEEADLSELPYLKNIVLETLRLHPATPLLVPHMASEDCKIGSYDMPRGTTLLVNAWAIHRDPNTWDDPDSFKPERFEKEEEAQKLLAFGLGRRACPGSGLAQRIVGLALGSLIQCFEWERVGNVEVDMKEGVGNTVPKAIPLKAICKARPFLHKIIS | |
| CYP81E1v2 | MDILSLLSYSVFYLALFFIFNIVIRARKFKNLPPGPPSLPIIGNLHHLKRPLHRTFKGLSEKYGHVISLWFGSRLVVVVSSASEFQQCFTKNDVVLANRPRFLSGKYIFYNYTTLGSTSYGEHWRNLRRITALDVLSNHRINSFSGIRRDETQRLITRLADDSSTNFAEIELSYRFYDMTFNNIMRMISGKRYYGEDCDMSDLQEASQFRDMVSELLQLSGANNKTDFMPLLRFLDFENLEKRLKDISGKTDAFLRGLIQEHRAKKERANTMIDHLLNLQDSQPEYYTDQIIKGLALAMLLAGTDSSAVTLEWSMSNLLNHPEVLKKVKDELDTHVGQDRLVDESDLPKLSYLKNVINETLRLYTPAPLLLPHSTSDECNIGGYKVPQDTIVLINAWAIHRDPELWTEATTFKPERFEKKGELEKLIAFGMGRRACPGEGLAIRAISMTLALLIQCFDWKLTNGDKIDMAERDGFTLTKLVPLKAMCKSRPVINKVFKQ | |
| CYP81F1 | MLYFILLPLLFLVISYKFLYSKTQRFNLPPGPPSRPFVGHLHLMKPPIHRLLQRYSNQYGPIFSLRFGSRRVVVITSPSLAQESFTGQNDIVLSSRPLQLTAKYVVYNHTTVGTAPYGDHWRNLRRMCSQEILSSHRLIIFQHIRKDEILRMLTRLSRYTQTSNESNDFTHIELEPLLSDLTFNNIVRMVTGKRYYGDDVNNKEEAELFKKLVYDIAMYSGANHSADYLPILKLFGNKFEKEVKAIGKSMDDILQRLLDECRRDKEGNTMVNHLISLQQQQPEYYTDVIIKGLMMSMMLAGTETSAVTLEWAMANLLRNPEVLEKARSEIDEKIGKDRLIDESDIAVLPYLQNVVSETFRLFPVAPFLIPRSPTDDMKIGGYDVPRDTIVMVNAWAIHRDPEIWEEPEKFNPDRYNDGCGSDYYVYKLMPFGNGRRTCPGAGLGQRIVTLALGTLIQCFEWENVKGEEMDMSESTGLGMRKMDPLRAMCRPRPIMSKLLL | |
| CYP81G1 | MIDLFLLALMAGFIAVAYVFRSKQKKNLPPNPVGFPVIGHLHLLKEPVHRSLRDLSRNLGIDVFILRLGSRRAVVVTSASAAEEFLSQQNDVVFANRPLATLTEYMGYNNTLVSTAPYGEHWRRLRRFCAVDILSTARLRDFSDIRRDEVRAMIRKINVELVTSGGSVRLKLQPFLYGLTYNILMSMVAGKREEDEETKEVRKLIREVFDFAGVNYVGDFLPTLKLFDLDGYRKRAKKLASKLDKFMQKLVDEHRKNRGKAELEKTMITRLLSLQESEPECYTDDIIKGLVQVMLLAGTDTTAVTLEWAMANLLNHPEVLRKLKTELNEVSKEGRVFEESDTGKCPYLNNVISETLRLFPAAPLLVPHASSTDCEVAGFDIPRRTWLFINAWAIQRDPNVWDDPETFKPERFESETHRGKFLPFGIGRRACPGMGLAQLVLSLALGSLIQCFDWERDNDVAVDMSEGKGLTMPKSVPLVAKCKSLPILDKLVL | |
| CYP81H1 | MDCILLILTTLVAIFIVKIVLLVTKPNKNLPPSPNICFPIIGHLHLLKKPLLHRTLSHLSHSLGPVFSLRLGSRLAVIISSPTAAEECFLTKNDIVLANRPRFIMGKYVAYDYTSMVTAPYGDHWRNLRRITALEVFSTNRLNASAEIRHDEVKMLLQKLHDLSVERPAKVELRQLLTGLTLNVIMRMMTGKRFFEEDDGGKAGISLEFRELVAEILELSAADNPADFLPALRWFDYKGLVKRAKRIGERMDSLLQGFLDEHRANKDRLEFKNTMIAHLLDSQEKEPHNYSDQTIKGLILMMVVGGTDTSALTVEWAMSNLLNHPQILETTRQNIDTQMETSSSRRLLKEEDLVNMNYLKNVVSETLRLYPVAPLMVPHVPSSDCVIGGFNVPRDTIVLVNLWAIHRDPSVWDDPTSFKPERFEGSDQFGHYNGKMMPFGLGRRACPGLSLANRVVGLLLGSMIQCFEWESGSGGQVDMTEGPGLSLPKAEPLVVTCRTREMASELLFFGSEPSNKNV | |
| CYP81J1 | STEIFSPVRIRSLAAVRQEEVKLMITGILASTSTDNSVKVNMKVVFSELMFNVIMKIIAGKRYFGVNTDSEVEEGQKFRVVFDEMFSTLEVASPQDFLPFLKWFGFKRMENRLTKLAKELDQLFQKLIEERRSERGKVQSTVIDVLLSLQETDREQYSDKLIKGMILSLIAAGTHTTAGTMEWAMSLLLNHPEALLKVRDEIDKKVGQDRLIDHSDLQNLSYLNNAIKESLRLFPTAPLLLAHESSAECTVGGFTIPSNTILFANAYALHRDPKVWTDPVSFKPERFENNGQQGSRIYVPFGLGRRSCPGEGLATQVVGLALGTLIQCFEWDRNGEEKVDMTDGSGLAMHMEKPLEAMCKPRQSIVDVINRL | |
| CYP81K1 | MEDLWFIFFSFLTATLIFFITKKFLWSLNSKLPPSPTPLPIIGHLHLIKKYPLPQALRHLSSNYGPVLFLKFGCRNVLTLSSPDSIEECFTNHDVTLANRPKTITSDHFSYGYKNFGFAPYGDLWRTLRRLSTLEVFSSASLQKNSSIRNEEVSNLCLIIFRLSRDSRIVDLKYQFTLLTAHIMLRLVSGKRGVKKSDPESEKRFLDDFKLRFFSSMSMNVCDYFPVLRWIGYKGLEKRVIDMQRMRDEYLQRLIDDIRMKNIDSSGSVVEKFLKLQESEPEFYADDVIKGIIVLMFNGGTDTSPVAMEWAVSLLLNHPDKLEKLREEIKSNVKHKGLIQDSDLSSLPYLRCVIYETLRLYPAAPLLLPHCSSKRFNLGNYEIPENIMLLVNAWAVHRDGELWEEANVFKPERFEGFVGDRDGFRFLPFGVGRRACPAAGLGMRVVSLAVGALVQCFEWEKVEAGDIDMRPVFGVAMAKAEPLVALPKPWSEMVPILSQL | |
| CYP81L6 | MDVALSTIVFVIAIFIPALLTLVQRSRSRHAGHNPPPPPEPRAIPLVGHLHHLLRKKPLHRCLAHLAERHGDVLGLRFGSSRVAVVSSASVAQQCLVALDTSFGNRPRLPSARILSYEWSTMGHSNCGPYWRQARRTTSTEFSSVERVQHFADVHEQEARAMARRLCRVAHASGGRALVDVKSRLLEMLMNGLLDMLFRRTTSRSRSSDEKDEAVEVSEEARCFMAMAEETMELTLTVWDFLPPLARWLDVDAVGRRLQRLQANRTEFLQRLIEEHKEMEKSGQVTRRTLVGVMLELQDKDPEAYTDQLIRSLCVSALEAGTLSTGYTIEWVMSLLLNNPHIMKKARDEIDACVGEPKRLLDATDLPKLPYLRCIILETLRLYPVVPLLVPRESSTNCTVNGFNIAKGTMLLVNTFAIHRDPRTWDDPETFLPERFEDGSNQSGKTTMDLSFGMGRRRCPAENLGMQLAGIALGTMIQCFNWERVGTELVDMAEGSGLTMAKKVPLEAFCQPRASMVDLLANI | |
| CYP81M1 | MANTTLSSLLFLSMASALFLLTLLRILRSKKQQRPPPPPAEPAVPPRHRGHLHLFKKPLHRALSGLAATHGPVLLLHFGSRAVLHVTDPAVAEECLTDHDVTFANRPRLPSSCHLSNGYTTLGSSSYGPNWRNLRRIATVEVFSAHRLLRSADVRGGEVPHMARWLYLAAPAAGPSEPARADVKARAFELVLNVVARMVAGKQYYGGEGDAEAETEEAARFREMVREYFAMHGASNLQDFVLLLGLVDIGGAKRRAVKLSRERNTWAQRLIDEHRATATAAAATEARTMVGDLLKMQASEPEAYSDKVITALCLSILQTGTDTSSSTIEWGMALLLNHPAAMAKARAEIDRFVGTGRVVEEADLPNLPYLQCIIRENLRLYPVGPLLAPHESSADCSVSVAGGGRYAVPAGTMLLVNVHAMHRDARFWGPDPESFSPERFEGGRSEGKWMLPFGMGRRRCPGEGLAVKVVGLALATLVQCFEWRRVGDEEVDMTEGSGLTMPKAVPLEALYWPRPEMVPALSGIFFIYNFFY | |
| CYP81N1 | MTGGLEVAMVAGGGNGGAAVLVGITVLLFVVVVVVVVLVRWWSGGEGGAAPSPPALPVLGHLHLLKKPLHRSLAAVAAGVGAPVVSLRLGARRALVVSTHAAAEECFTACDAALAGRPRTLAGEILGYDHTIVLWTPHGDHWRALRRFLAVELLSAPRLAALAADRHAEAASLVDAILRDAAGGAKVTLRPRLFELVLNVMLRAATTRRRHASVDARKLQEIIEETFSVNGTPSVGDFFPALRWVDRLRGKVGSLKKLQARRDAMVTGLIDDHRQWRSGSAGDGDQDKEKKGVIDALLALQETDPDHYTDNVVKGIILSLLFAGTDTSALTIEWAMAQLVTHPETMKKARAEIDANVGTARLVEEADMANLPYIQCVIKETLRLRTAGPVIPAHEAMEDTTVGGFRVARGTKVLVNAWAIHRDGDVWDAPEEFRPERFVDSDAGGAVTAPMMPFGLGRRRCPGEGLAVRVVGVSVAALVQCFDWEVGDDDVVDMTEGGGLTMPMATPLAAVCRPREFVKTILSTS | |
| CYP81P1 | MEISQAFVFASLLLLLLLTWLLFHLLSYQAPPPNGDGGRRIPSPPALPVVGHLHLLKKPLHRSLAALAARYGGGAGLLLLRFGARPVVLVSSQAAADECFTAHDAALAGRPGLASRRLLTDGCPTIATAGHSARWRHLRRLATVHALCARRLAATSPARDAEARAMAARLYSSSSSSSAASAVVVGVKPAAYGFVASVIMSMVAGERMAEEDVLRFKAITEAGLAAAGAANRQDFLPFLRLLDFGRARRRLAGIAKERHDFGQRIVDEYRRRHRRRLAVAADDFSSSPPRRTVIGDLLRQQESSPESYADEVIRTVCLSLLQAGTDTSASTIEWAMALLLNNPDVLRKATDEINSVVGMSRLLQEPDLANLPYLRCIITETLRLYPLAPHLVPHEASRDCMVAGHVIARGTMVLVDVYSMQRDPRVWEDPDKFIPERFKGFKVDGSGWMMPFGMGRRKCPGEGLALRTVGMALGVMIQCFQWERVGKKKVDMSEGSGLTMPMAVPLMAMCLPRVEMESVLKSL | |
| CYP81Q1 | MEAEMLYSALALTFAIFMVYRILSNSQDKRSLTKLPPSPPGWLPVIGHAHLMKNLLHRTLYDFSQKLGPIFSIRFGSRLVVVVSSSSLVEECFTKYDIVLANRPQASVDRRSLGFSTTSVIGAPYGDHWRNLRKLCDLEVFAPTRLASFLSIRLDERDRMISALYKISSAGFAKVNLEAKIVELTFNNIMRMVAAKRYYGEEAEDDEEAKRFRDLTKEALELTSASNPGEIFPILRWLGCNGLEKKLAVHSRKTDEFMQGLLDEHRRGERQNTMVDHLLSLQESQPEYYTDEIITGLIVALIIAGTDASVVTTEWAMSLLLNHPKVLEKARKELDTLVGHERMVDEHDLPKLRYLHCIVLETLRLFPSVPTLVPHEPSEDCKIGGYNVPKGTMVLVNAWAIHRDPKVWDDPLSFKPDRFEIMEVETHKLLPFGMGRRACPGAGLAQKFVGLALGSLIQCFDWERTSPEKIDLNEGSGITLPKAKTLEAMCKPRHVMEKVLRQVSNV | |
| CYP81Q7 | MEITWLSTSLCLLFLSFAFNIFLQRRRIHPHLPPSPPAIPILGHLHLLLKPPIHRQLQSLSKKYGPIFSLRFGSSPVVIISSPSTVEECFTKNDIIFANRPRWLIGKYIGYNYTTIASASYGEHWRNLRRLSALEIFSSNRLNMFLGTRRDEIKILLHRLSQNSRDNFARVELRPMFTELTCNIIMRMVTGKRYYGEDVDSEEAKRFQKIMRGIFELAGASNPGDFLPLLRWVDFGGYEKKLVKLNREKDVIFQGLIDEHRSPDQGLVNKNSMIDHLLSLQKSEPEYYTDEIIKGLALILTFAGTDTTATTIEWAMSLLLNHPDVLKKARAELDTHVGKDRLMEESDFPKLQYLRSIISETLRLFPATPLLIPHISSDNCQIGGYDIPRGTILLVNAWAIHRDPKSWKDATSFKPERFENGESEAYKLLPFGFGRRACPGAGLANRVIGLTLGLLIQCYEWERVSEKEVDMAEGKGVTMPKLEPLEAMCKARAIIRKVL | |
| CYP81R2 | MNYMYYCLAFFLSSFLVFKLVFQRSRNLPPSPFGFPIIGHLHLVSKPPMHKVLAILSNKCGPVFTLKLGSRNIVAVCSLSAAEECYIKNDIVFANRPQSIFVHYWSYNYAAFLFAPYGHLWRTLRRFSVTELFSRSCLDRSAAISEEVRTLVRLILSKVSDDGAKKVDLNYFFTITSLNVIMKMNAGKKWVEEEKAACIDSGKQCIEDVQKIFPSNPGTTVLDFFPFLKWFGYRGEEESVIKVYKERDEFLQGLIEEVKRKETSSVTSNPAEGVKDQTTVIGSLLALQKSDPELYTDEVVKGTMATLYLAGVDTVDFTTEWAMTFLLNHPERLERVKAEIDREVGHERLVQESDLPKLRYVRCVVNETLRLYPPAPLLLPHAPSEDCIVGGYKIPRGTIVMVNAWAIHRDPKLWEDPESFKPERFEGLNNEGEKQGFIPFGIGRRACPGNHMAMRRVMLALAALIQCFEWERVGKELVDMSIVDALISVQKAKPLEAICTPRPFTTTLISPP | |
| CYP81S1v1 | MEEDYSASLWLRYSFLLPCMVFLVLSTKFLLHKRKQGKINHLPPSPFALPIIGHLYLLKQPIHRTLHSLSKKYGPIFSIKLGSRLAVVISSPSAVEECFTKNDIVLANRPYFLSSKYLNYNNTTMGSVEYGEHWRNLRRISALEIFSPPRLTSLFSIRREEVMALLRRLHGVSKHGNYAKVELRSMLLDLTSNIIMRMVAGKRYYGEDVKEIEEARIFKEIMEEFAECIAVRNLGDMIPLLQWIDFTGHLKKLDRLSKKMDVFLQGLVDEHRDDRDRNTMINRFLALQEEQPEYYTDEVIKGHVLVLLIGGTETAATSMEWALANLLNHPNVLKKAKAELDAQVGDRLIDESDFAKLHYLQSIISENLRLCPVTPLIPPHMPSSDCTIGGYHVPAGTILFVNAWSLHRDPTLWDEPTSFKPERFESAGRVDACKFIPFGMGRRACPGDGLANRVMTLTLGSLIQCFEWERVGENKIDMTEKTAMTMFKVEPLELMCRARPILDMLLSLSGQKI | |
| CYP81T1 | MEVSHWFNFAALFFFFVLASKLVIYKLGNPKNLPPSPPSRPIIGHLHLLKQPIHRTLCELSKKYGDILFLRFGARKVLVISSPSAVEECFTRKDVIFANRPRTLAGKHLNYNSTTMGFSSYGEHWRNLRRLTTIELFSASRVASFSDIRKEEVQLLLNQLFRDSSKQQAKVGLTASFMELTFNVMMRMIAGKRYYGKEVVDEEAGQFQNIIKEMEALRGSSNMNDFFPVLQWIDFQGLEKRMMGLKKKMDKFLQDLIEEHQKVRSQSSQSTKITGLGNQKRNMTLIDVMLSLKETEPEFYTDQTIKGVIMSTLTAGSQTSAATLEWAMSLLLNNPETMRKASEEIDAIVGTEHILDEVDVTKLSYLQNILNETFRLFPPAPLLLPHESSEDCTISGFHVPRGTMLLVNTWSIHRDTKLWVEPTKFMPERFEGGEGEGYKLLPFGAGRRACPGAGLAKRIIGLTLGVLIQCFEWDRVSKEEINLTEGTGLTIPKAEPLEALCRPRQSMVNLLSSM | |
| CYP81U1 | MVGILVYVALFILSLLLFLTAAAKLHNSKSKIKNQAPSPPSLPVVGHLHLLKKPLHRSISLLSARHGPILLLRFGSRPALAVSSLPLAEECLSGKNDLAFANRAHFPHEAAPLQLLTLGSANYGPHWRMLRRISAVELLSSHRINSFSQLRSEEVHSMISTLFRESSDKELNRVELKSKLFELAMNNMMRMIFGKDLASSEGAGRFREMVKESHSLLGASTRLGDFFPFLGWMDWRARRMVLRLVRRRDEFLQSLIDAHARKMEEVEEKTMIRVLVELQKSNRESNNDEGFMLKPLIIGLLQAGTDTSSDTIEWAMSLLLNNRDKLKKARDEIDARVGKERLLRESDLPNLPYLQCVITETLRLYPAAPLLVPHESAEECTVGGYAVPQGTMLLVNAYAIRVVGIVLGTLIQCFEWERVGEEEVDMTEGSGLTLPRANPLEAICRPRQSMISVLAGL | |
| CYP81W1 | MGNLYHYAVILLPIILIIKFLFHGRQRQRYRLPPSPFALPVIGHLHLLKPPLYQGLQALSSQYGPILFLRFGCRPFVVVSSPSAVQECFTKNDVVLANRPRSMIGDHVTYNYTAFAWASYGHLWRVLRRLTVVEILSSNKLLLLSTVREEEVRYLLRQLFKVSNDGAQKVDMRLYLSLFSFNFIMKTITGKRCIEEEAEGIETNRQFLERLKRIFVPTTTTNLCDFFPILRWVGYKGLEKSVIQFGKERDGYLQGMLDEFRRNNSAVEWQKKRTLIETLLFLQQSEPDFYTDDVIKGLMLVISAGTDTSSVTLEWAMSLLLNHPEALEKARAEIDSHVKPGHLLDDSDLAKLPYLRSVVNETLRLYPTAPLLLPHLSSEDCSVGGFDIPRGTTVMVNVWALHRDPRVWEEATKFKPERFEGMENEEKEAFKFAPFGIGRRACPGAALAMKIVSLALGGLIQCFEWERVEAEKVDMSPGSGITMPKAKPLEIIFRPRPTMTSLLSQL | |
| CYP81X1 | MEEEADYRLIVITASVGFLLLFLYVLKSILLKSKNLPPSPPYALPLIGHLHLIKEPLHLSLHKLTDKYGPIIFLCLGTRKVLVVSSPSAVEECFTKNDITFANRPQTLAAKHLNYNKTTIGVASYGHYWRNLRRLTTVELFSTTRLAMLTSVRVEEVQLMVKQLFEECKGRQQIMIDLRARLLEVSFNIMLRMISGKRYYGKHAIAQEGKEFQILMKEFVELLGSGNLNDFFPLLQWVDFGGVEKKMVKLMKKMDSFLQKLLDEHCTRRNVMSEEEKERRKSMTLIDVMLDLQQTEPEFYTHETVKGVILAMLVAGSETSATTMEWAFSLLLNHPKKMNKVKEEIDTYVGQDQMLNGLDTTKLKYLQNVITETLRLYPVAPLLLPHESSNDCKVCGFDIPRGTMLLVNLWTLHRDANLWVDPAMFVPERFEGEEADEVYNMIPFGIGRRACPGAVLAKRVMGHALGTLIQCFEWERIGHQEIDMTEGIGLTMPKLEPLVALCRPRQSMIKVLSNI | |
| CYP82A6 | MDFVLNYLNTTTIAFISLISLLFFLFRFSKVSHTKEPPIVSGSWPLLGHLPLMRNTQTPHKTLGALVDKYGPIFTIKLGATNALVLSNWELAKECFTKNDIVVSSRPKPVAVELMSYNQAFIGWAPYGTYWRQLRKIVTLEILSNRRIELLSHIRVSEVQTSIKELVNVWSNQMSSQYGLLDDTKSSSTNDYASVELKKWFAQLTLNMVLRMVVGKRCFGDVDVENKEEAKKFLENIRDFMRLIGTFTVGDGVPFLKWLDLGGHEKEMKKCAKKFDEMLNEWLEEHREKKGLGSEDKVVGERDFMDAMLLVLKDKPIEGFDVDTIIKATTLELILGGSDTTAGTLTWAMCLLLKHPHVLEKLKEELNTYIGKERCVKESDINKLVYLHAIIKETLRLYPPAPFSSPREFTEDCTIGGYHIKKGTRLMPNLWKIHRDPNVWPDPLEFKPERFLSTHKDVDVRGQNFELLPFGSGRRMCAGMSLGLHMVHYILANFLHSFEILNPSPESIDVTEVLEFVTTKATPLEVLVKPCLSFKCYESM | |
| CYP82B1 | MEKPILLQLQAGILGLLALICFLYYVIKVSLSTRNCNQLVKHPPEAAGSWPIVGHLPQLVGSGKPLFRVLGDMADKFGPIFMVRFGVYPTLVVSTWEMAKECFTSNDKFLASRPPSAASSYMTYDHAMFGFSFYGPYWREIRKISTLHLLSHRRLELLKHVPHTEIHNFIKGLFGIWKDHQKQQQPTGREDRDSVMLEMSQLFGYLTLNVVLSLVVGKRVCNYHADGHLDDGEEAGQGQKLHQTITDFFKLSGVSVASDALPLLGLFDLGGKKESMKRVAKEMDFFAERWLQDKKLSLSLSSETNNKQNDAGEGDGDDFMDVLMSILPDDDDSLFTKYSRDTVIKATSLSMVVAASDTTSVSLTWALSLLLNNIQVLRKAQDELDTKVGRDRHVEEKDIDNLVYLQAIVKETLRMYPAGPLSVPHEAIEDCNVGGYHIKTGTRLLVNIWKLQRDPRVWSNPSEFRPERFLDNQSNGTLLDFRGQHFEYIPFGSGRRMCPGVNFATLILHMTLARLLQAFDLSTPSSSPVDMTEGSGLTMPKVTPLKVLLTPRLPLPLYDY | |
| CYP82C2 | MDTSLFSLFVPILVFVFIALFKKSKKPKHVKAPAPSGAWPIIGHLHLLSGKEQLLYRTLGKMADQYGPAMSLRLGSSETFVVSSFEVAKDCFTVNDKALASRPITAAAKHMGYDCAVFGFAPYSAFWREMRKIATLELLSNRRLQMLKHVRVSEISMVMQDLYSLWVKKGGSEPVMVDLKSWLEDMSLNMMVRMVAGKRYFGGGSLSPEDAEEARQCRKGVANFFHLVGIFTVSDAFPKLGWFDFQGHEKEMKQTGRELDVILERWIENHRQQRKVSGTKHNDSDFVDVMLSLAEQGKFSHLQHDAITSIKSTCLALILGGSETSPSTLTWAISLLLNNKDMLKKAQDEIDIHVGRDRNVEDSDIENLVYIQAIIKETLRLYPAGPLLGHREAIEDCTVAGYNVRRGTRMLVNVWKIQRDPRVYMEPNEFRPERFITGEAKEFDVRGQNFELMPFGSGRRSCPGSSLAMQVLHLGLARFLQSFDVKTVMDMPVDMTESPGLTIPKATPLEILISPRLKEGLYV | |
| CYP82C2 | MDTSLFSLFVPILVFVFIALFKKSKKPKHVKAPAPSGAWPIIGHLHLLSGKEQLLYRTLGKMADQYGPAMSLRLGSSETFVVSSFEVAKDCFTVNDKALASRPITAAAKHMGYDCAVFGFAPYSAFWREMRKIATLELLSNRRLQMLKHVRVSEISMVMQDLYSLWVKKGGSEPVMVDLKSWLEDMSLNMMVRMVAGKRYFGGGSLSPEDAEEARQCRKGVANFFHLVGIFTVSDAFPKLGWFDFQGHEKEMKQTGRELDVILERWIENHRQQRKVSGTKHNDSDFVDVMLSLAEQGKFSHLQHDAITSIKSTCLALILGGSETSPSTLTWAISLLLNNKDMLKKAQDEIDIHVGRDRNVEDSDIENLVYIQAIIKETLRLYPAGPLLGHREAIEDCTVAGYNVRRGTRMLVNVWKIQRDPRVYMEPNEFRPERFITGEAKEFDVRGQNFELMPFGSGRRSCPGSSLAMQVLHLGLARFLQSFDVKTVMDMPVDMTESPGLTIPKATPLEILISPRLKEGLYV | |
| CYP82D1 | MDVTIEYLYTIVAGVICIILISYSKFFRGDARAQPKLPPLASGGWPLIGHLHLLGSSNQPPYITLGNLADKYGPIFTLRVGVHNAVVVSTWELAKEIFTTHDVIISSRPKFTAAKILGHDYANFGFSPYGDYWQMMRKVTASELLSTRRFETLRDIRDSEVKKSLMELCNSGFDGDLKVEMKRFLGDMNLNVIMRMIAGKRYSNNESGDEREVRKVRWVFREFFRLTGLFVVGDAIPFLGWLDLGGHVKEMKKAAREMDSVVCGWLEDHRHKNVIGETKMEQDFIDVLLSVLHGVHLDGYDVDTVIKATCLTLIAGATDTTTVTITWALSLLLNNRHTLKKVQDELDEKVGKDRLVNESDINNLVYLQAVVKETLRLYPAGPLSGARQFTKDCTVGGYNIRAGTRLILNLWKMHRDPRVWSEPLEFQPERFLNTHKDVDVKGQHYELLPFGGGRRSCPGITFGLQMTNLALASFLQAFEVTTPSNAQVDMSATFGLTNIKTTPLEVIAKPRLPYHLLFVKEH | |
| CYP82E1 | MYHLLSPIEAIVGLVTFAFLLYLLWTKKQSKILNPLPPKIPGGWPVIGHLFYFNNNGDDDRHFSQKLGDLADKYGPVFTFRLGFRRFLAVSSYEAMKECFSTNDIHFADRPALLYGEYLCYNNAMLAVAKYGPYWKKNRKLVNQELLSVSRLEKFKHVRFSIVQKNIKQLYNCDSPMVKINLSDWIDKLTFDIILKMVVGKTYNNGHGEILKAAFQKFMVQAMEIELYDVFHIPFFKWLDLTGNIKAMKQTFKDIDNIIQGWLDEHIKKRETKDVGGENEQDFIDVLLSKRSNEHLGDGYSHDTTIKATVFTLVLDATDTLALHIKWVMALMINNKNVMKKAQEEMDTIVGRDRWVEENDIKNLVYLQAIVKEVLRLHPPAPLSVQHLSVKDCVVNGYHIPKGTALLTNIMKLQRDPQIWVDPDTFDPERFLTTNAAIDYRGQHYELIPFGSGRRACPAMNYSLQVEHLSIAHLIQGFNFATTTNEPLDMKQGVGLTLPKKTDVEVLITPRLPPTLYQY | |
| CYP82F1 | MDLIMLFLLSALFIFPVLILIKSRLRPKNKKSTAPMVPGAWPLLGHLHLFDTVNPTHVTFGAMADVYGPVFMAKLGSIKVMIINSKEVAKEIYTVHDKLLERPELTASKLLGYNDSFLTFSPYGLYWREIRKIAVSELFSTSGVDMHMVSRAREADLAFRALYVRWEKRGKPKEGVLVDMKQEFIDLTANISLMMVSGKRYFGENPNCEVKEARRCGKLIREFLDYFALFLLSDVAPVLGFLDWKTKRGMKRTAKGLDKVAEGWIEEHKNKRSDHGRSENDYLDILIKILGQDKIPGLSDTHTKIKALCLNLVLAGSETAIVVLVWAVSLLLNNPHVLRKAQEELDSKIGKERVVEELDIKDLVYLQAIVKETFRLYPPVPLVAYRAVVEDFDIAFCKCHVPAGTQLMVSAWKIHRDPNVWSNPEQFEPERFLTSNRELDVGGQSYKFFPFGLGRRSCPAIPLGMRMVHYLLVRFLHSFDLARPSSQDVDMTESNGLVNHKATPLEVNIIPRLHKSLYEVDHIGTDN | |
| CYP82G1 | MTFLFSTLQLSLFSLALVIFGYIFLRKQLSRCEVDSSTIPEPLGALPLFGHLHLLRGKKLLCKKLAAMSQKHGPIFSLKLGFYRLVVASDPKTVKDCFTTNDLATATRPNIAFGRYVGYNNASLTLAPYGDYWRELRKIVTVHLFSNHSIEMLGHIRSSEVNTLIKHLYKGNGGTSIVKIDMLFEFLTFNIILRKMVGKRIGFGEVNSDEWRYKEALKHCEYLAVIPMIGDVIPWLGWLDFAKNSQMKRLFKELDSVNTKWLHEHLKKRSRNEKDQERTIMDLLLDILPEDIVISGHVRDVIVKATILALTLTGSDSTSITLTWAVSLLLNNPAALEAAQEEIDNSVGKGRWIEESDIQNLKYLQAIVKETHRLYPPAPLTGIREAREDCFVGGYRVEKGTRLLVNIWKLHRDPKIWPDPKTFKPERFMEDKSQCEKSNFEYIPFGSGRRSCPGVNLGLRVVHFVLARLLQGFELHKVSDEPLDMAEGPGLALPKINPVEVVVMPRLDPKLYSLL | |
| CYP82H1 | MITCEMGIYLQMQDIILFSLVFFSTLILWRIFSTYVIRKKTCSGPPEPAGRWPLIGHLHLLGGSKILHHILGDMADEYGPIFSLNLGINKTVVITSWEVAKECFTTQDRVFATRPKSVVGQVVGYNSRVMIFQQYGAYWREMRKLAIIELLSNRRLDMLKHVRESEVNLFIKELYEQWSANGNGSKVVVEMMKRFGDLTTNIVVRTVAGKKYSGTGVHGNEESRQFQKAMAEFMHLGGLLMVSDALPLLGWIDTVKGCKGKMKKTAEEIDHILGSCLKEHQQKRTNISNNHSEDDFIYVMLSAMDGNQFPGIDTDTAIKGTCLSLILGGYDTTSATLMWALSLLLNNRHVLKKAQDEMDQYVGRDRQVKESDVKNLTYLQAIVKETLRLYPAAPLSVQHKAMADCTVAGFNIPAGTRLVVNLWKMHRDPKVWSDPLEFQPERFLQKHINVDIWGQNFELLPFGSGRRSCPGITFAMQVLHLTLAQLLHGFELGTVLDSSIDMTESSGITDPRATPLEVTLTPRLPPAVYQ | |
| CYP82J1 | MDFSFHLLAVSTVLALVLWYTLRRVRETRRKTEKGLQPPEPSGALPLIGHLHLLGAQKTLARTLAAMADKYGPIFTIRLGKHPTVVVSNLEAIKECFTTHDRILSSRPRSSHGEHLSYNYAAFGFNNSGPFWREMRKIVTIQLLSSHRLKSLRHVQVSEVNTLINDLYLLSKSNKQGSTKIDISECFERMTINMITRMIAGKRYFSSTEAEKEDEGKRIGKLMKEFMYISGVFVPSDVIPFLGWMNNFLGSVKTMKRLSRELDSLMESWIQEHKLKRLESTENTNKMEDDDFIDVMLSLLDDSMFGYSRETIIKATAMTLIIAGADTTSITLTWILSNLLNNRRSLQLAQEELDLKVGRERWAEDSDIGNLVYIQAIIKETLRLYPPGPLSVPHEATKDFCVAGYHIPKGTRLFANLWKLHRDPNLWSNPDEYMPERFLTDHANVDVLGHHFELIPFGSGRRSCPGITFALQVLHLTFARLLQGFDMKTPTGESVDMTEGVAITLPKATPLEIQITPRLSPELYYEC | |
| CYP82K1 | MIIWRILSTSHKRNKTLPPPEPSGAWPLIGHLRILNSQIPFFRILGDLAVKHGPVFSIRLGMRRTLVISSWESVKECFKTNDRKFLNRPSFAASKYMGYDDAFFGFHPYGEYWLEMRKIATQELLSNRRLELLKHVRVSEIETCIKELHTTCSNGSVLVDMSQWFSCVVANVMFRLIAGKRYCSGIGKDSGAFGRLVREFFYLGGVLVISDLIPFTEWMDLQGHVKSMKRVAKELDHVVSGWLVEHLQRREEGRVRKEEKDFMDVMLESLAVGDDPIFGYKRETIVKATALNLILAGTDTTSVTLTWALSLLLNHTEVLKRAQKEIDVHVGTTRWVEESDIKNLVYLQAIVKETLRLYPPGPLLVPRESLEDCYVDGYLVPRGTQLLVNAWKLHRDARIWENPYEFHPERFLTSHGSTDVRGQQFEYVPFGSGRRLCPGISSSLQMLHLTLSRLLQGFNFSTPMNAQVDMSEGLGLTLPKATPLEVVLTPRLENEIYQH | |
| CYP82L1 | MILEALILVFLYGFWKILARNSEGKKSTRAPEPSGAWPLFGHLPSLVGKDPACKTLGAIADKYGPIYSLKFGIHRTLVVSSWETVKDCLNTNDRVLATRAGIAAGKHMFYNNAAFALAPYGQYWRDVRKLATLQLLSNQRLEMLKHVRVSEVDTFIKGLHSFYAGNVDSPAKVNISKLLESLTFNINLRTIVGKRYCSSTYDKENSEPWRYKKAIKKALYLSGIFVMSDAIPFLEWLDYQGHVSAMKKTAKELDAVIRNWLEEHLKKKIDGELGSDRESDFMDVMISNLAEGPDRISGYSRDVVIKATALILTLTGAGSTATTLVWTLSLLLNNPTVLKAAQEELDKQVGRERWVEESDIQNLKYLQAIVKETLRLYPPGPLTGIREAMEDCSIGGYDVPKGTRLVVNIWKLHRDPRVWKNPNEFKPDRFLTTHADLDFRGQNMEFIPFSSGRRSCPAINLGLIVVHLTLARILQGFDLTTVAGLPVDMIEGPGIALPKETPLEVVIKPRLGLELY | |
| CYP82M1v1 | MDYHISFHFQALLGLLAFVFLSIILWRRTLTSRKLAPEIPGAWPIIGHLRQLSGTDKNIPFPRILGALADKYGPVFTLRIGMYPYLIVNNWEAAKDCLTTHDKDFAARPTSMAGESIGYKYARFTYANFGPYYNQVRKLALQHVLSSTKLEKMKHIRVSELETSIKELYSLTLGKNNMQKVNISKWFEQLTLNIIVKTICGKRYSNIEEDEEAQRFRKAFKGIMFVVGQIVLYDAIPFPLFKYFDFQGHIQLMNKIYKDLDSILQGWLDDHMMNKDVNNKDQDAIDAMLKVTQLNEFKAYGFSQATVIKSTVLSLILDGNDTTAVHLIWVMSLLLNNPHVMKQGQEEIDMKVGKERWIEDTDIKNLVYLQAIVKETLRLYPPVPFLLPHEAVQDCKVTGYHIPKGTRLYINAWKVHRDSEIWSEPEKFMPNRFLTSKANIDARGQNFEFIPFGSGRRSCPGLGFATLVTHLTFGRLLQGFDFSKPSNTPIDMTEGVGVTLPKVNQVEVLITPRLPSKLYLF | |
| CYP82Q1 | MEFHLSLLTTVTAILVVTAFFLQISKRKKVDTTKNKLLPPKAKGAWPLIGHLPLLGKNRIAHRVLGDLADKYGPIFTIKLGVYQVLVVSSADAVKDCFTTSDKAFASRPKSTAVEIMGYNYAMFGLAPYGEYWRQVRKIAVLEILSQKRVDMLEWARVSEVRTSTNEVYDAWRVNKENEGSDMVLVDMKQWFTNLILNVLVRIISGKRFPFKSVEGIRFQKMEKKLFELLGAFVVSDLIPSMKRFDIGGYQKQMKMAAEEINDIMEGWLNNRKTQKESGEQKEGDQYFMDVLISVLKDASDADFPGYDHDTVIKATCMALLAAGSDTTSVTIIWALALLLNHPEKMKIAQDEIDKHVGRDRLVEESDLKNLVYINAIIKETMRLYPAAPLSVPHEAMEDCVVGGYHIPKGTRLLPNFWKIQHDPNIWPEPYEFKPERFLSTHKDVDVKGKHFELLPFGTGRRMCPAITFVLQILPLTLANLIQQFEIRKPSNDPIDMTESAGLTTKRATPLDVLIAPRLSLKMYPVDV | |
| CYP82R1 | MHNPTLKQWIRTSTLPAPIIVSFIVILLFYFFKKRSSNMIRTKKAPEVVGAWPVIGHLNLLSVPKPAYIVLGELADQYGPAFSIQFGVHPILVVSSWELVKACFTTNDKFFSSRLVNKAIKYMFYDQKTISFAPHGPYWRELRKMITLNLLSNERLKMLKHQRISEMDACLKKLYELSTKRKDENAGVLVDMSKWFGDISFNVVTRIVAGKHIFGPKTERYMNVMEEARRLMDVMVFSDVIPYLGWLDRLRGVDSEIKRTAKELDSALESWVDEHRQKRVSISAGIGGIVNITEEEEIDFIDIMLSIIAKNKLLGDDPGTLIKAIVQEMYLAAWDNTTVTLTWALCLLLNNKQVLKRAQCELDAQVGKERQVEDSDINTLPYIQAIVKESMRLYPPGPIIERETTEDCDVGDFRIPAGTRLWINLWKLQRDPNVWPNDPQEFQPERFLNGHADIDMKGQHFELIPFGSGRRMCPGVSFSLQVMHLVLARIIHGFELKTPTDADIDMSTTLGMISWKATPLEVLLTPRFPPVFYM | |
| CYP82S1 | MDLPSHFLAIAGLILGLVLWYNHWRGKTLTHKSKGMSPPEPSGAWPFVGHLHLLHGKVPVFRTLGAMADKVGPVFVIRLGMYRTLVVSNREAAKECFTTNDKIFASRPNSSAAKILGYNYAAFAFAPHGPYWREMRKLSMLEILSTRRLGDLMHVQVSELHAGIKDLYILGKDNNWVNPKKVVISEWFEHLTFNVVLRMVAGKRYFNNVVHGGEEARSAIAAIKKLLLLVGASVASDVIPFLEWVDLQGHLSSMKLVAKEMDSLIESWVKEHTGRLNSEASSSQDFIDIMLTKLKDDSLFGYSRETIIKATVLTMIVAGSDTTSLTSTWLLSALLNNKHVMKHAQEELDLKVGRDRWVEQSDIQNLVYIKAIVKETLRLYTTFPLLVPHEAMEDCHVGGYHISKGTRLLVNAWKLHRDPAVWSNPEEFQPERFLTSHANVDVFGQHFELIPFGSGRRSCPGLNMGLQMLHLTIARLLQGFDMTKPSNSPVDMTEGISVALSKLTPLEVMLTPRLPAELY | |
| CYP83A1 | MEDIIIGVVALAAVLLFFLYQKPKTKRYKLPPGPSPLPVIGNLLQLQKLNPQRFFAGWAKKYGPILSYRIGSRTMVVISSAELAKELLKTQDVNFADRPPHRGHEFISYGRRDMALNHYTPYYREIRKMGMNHLFSPTRVATFKHVREEEARRMMDKINKAADKSEVVDISELMLTFTNSVVCRQAFGKKYNEDGEEMKRFIKILYGTQSVLGKIFFSDFFPYCGFLDDLSGLTAYMKECFERQDTYIQEVVNETLDPKRVKPETESMIDLLMGIYTEQPFASEFTVDNVKAVILDIVVAGTDTAAAAVVWGMTYLMKYPQVLKKAQAEVREYMKEKGSTFVTEDDVKNLPYFRALVKETLRIEPVIPLLIPRACIQDTKIAGYDIPAGTTVNVNAWAVSRDEKEWGPNPDEFRPERFLEKEVDFKGTDYEFIPFGSGRRMCPGMRLGAAMLEVPYANLLLSFNFKLPNGMKPDDINMDVMTGLAMHKSQHLKLVPEKVNKYM | |
| CYP83A1 | MEDIIIGVVALAAVLLFFLYQKPKTKRYKLPPGPSPLPVIGNLLQLQKLNPQRFFAGWAKKYGPILSYRIGSRTMVVISSAELAKELLKTQDVNFADRPPHRGHEFISYGRRDMALNHYTPYYREIRKMGMNHLFSPTRVATFKHVREEEARRMMDKINKAADKSEVVDISELMLTFTNSVVCRQAFGKKYNEDGEEMKRFIKILYGTQSVLGKIFFSDFFPYCGFLDDLSGLTAYMKECFERQDTYIQEVVNETLDPKRVKPETESMIDLLMGIYTEQPFASEFTVDNVKAVILDIVVAGTDTAAAAVVWGMTYLMKYPQVLKKAQAEVREYMKEKGSTFVTEDDVKNLPYFRALVKETLRIEPVIPLLIPRACIQDTKIAGYDIPAGTTVNVNAWAVSRDEKEWGPNPDEFRPERFLEKEVDFKGTDYEFIPFGSGRRMCPGMRLGAAMLEVPYANLLLSFNFKLPNGMKPDDINMDVMTGLAMHKSQHLKLVPEKVNKYM | |
| CYP83A2/CYP83B1 | MDLLLIIAGLVAAAAFFFLRSTTKKSLRLPPGPKGLPIIGNLHQMEKFNPQHFLFRLSKLYGPIFTMKIGGRRLAVISSAELAKELLKTQDLNFTARPLLKGQQTMSYQGRELGFGQYTAYYREMRKMCMVNLFSPNRVASFRPVREEECQRMMDKIYKAADQSGTVDLSELLLSFTNCVVCRQAFGKRYNEYGTEMKRFIDILYETQALLGTLFFSDLFPYFGFLDNLTGLSARLKKAFKELDTYLQELLDETLDPNRPKQETESFIDLLMQIYKDQPFSIKFTHENVKAMILDIVVPGTDTAAAVVVWAMTYLIKYPEAMKKAQDEVRSVIGDKGYVSEEDIPNLPYLKAVIKESLRLEPVIPILLHRETIADAKIGGYDIPAKTIIQVNAWAVSRDTAAWGDNPNEFIPERFMNEHKGVDFKGQDFELLPFGSGRRMCPAMHLGIAMVEIPFANLLYKFDWSLPKGIKPEDIKMDVMTGLAMHKKEHLVLAPTKHI | |
| CYP83D1 | LVLLSLLSIVISIVLFITHTHKRNNTPRGPPGPPPLPLIGNLHQLHNSSPHLCLWQLAKLHGPLMSFRLGAVQTVVVSSARIAEQILKTHDLNFASRPLFVGPRKLSYDGLDMGFAPYGPYWREMKKLCIVHLFSAQRVRSFRPIRENEVAKMVRKLSEHEASGTVVNLTETLMSFTNSLICRIALGKSYGCEYEEVVVDEVLGNRRSRLQVLLNEAQALLSEFFFSDYFPPIGKWVDRVTGILSRLDKTFKELDACYERSSYDHMDSAKSGKKDNDNKEVKDIIDILLQLLDDRSFTFDLTLDHIKAVLMNIFIAGTDPSSATIVWAMNALLKNPNVMSKVQGEVRNLFGDKDFINEDDVESLPYLKAVVKETLRLFPPSPLLLPRVTMETCNIEGYEIQAKTIVHVNAWAIARDPENWEEPEKFFPERFLESSMELKGNDEFKVIPFGSGRRMCPAKHMGIMNVELSLANLIHTFDWEVAKGFDKEEMLDTQMKPGITMHKKSDLYLVAKKPTT | |
| CYP83E1 | MLPMLLLLVLCLTLPLLMFFHKHKTNTNNPPGPKGLPIIGNLLQLDISNLHLQFSQFSKIYGPLFSLQLGLRPAIVVSSAEIAKEVFKNNDHVFSNRPISYGQNILSYNGSEIVFAPYGDFWREIRKICAIHIFSSKRVSYYSSIRIFEVKKMIKNISVHADSSNVTNLSELLISLSSTIICRTAFGKSYEDDGIEKSRFHGLLHEFQALLAASFFADYIPFTGWIDKLRGLHGRVDRNFKEFDEFYQEIIDEHLDPNREQITDEEDIVDVLLELKKKRSFSFDINFDHIKGILTDMLVAATDTTSAASVWAMTALIKNPRVMSKVKGEIRNLGVKKDFLYEDDIQNCPYLKAVVKETLRLHLPAPLLVPRESIENCTINGYNIPAKTILYVNAWAIQRDPDIWINPEEFYPERFLESSINFIGQDFELIPFGAGRRICPSIPMAVASLELILANLLYSFDWKLPHGLVKEDIDTSMLPGITQHKKNPLCLIAKVPK | |
| CYP83F1v1 | MALLIFVILFLSIIFLFLLKKNKISKRACFPPGPNGLPLIGNLHQLDSSNLQTQLWKLSQKYGPLMSLKLGFKRTLVISSAKMAEEVLKTHDLEFCSRPLLTGQQKFSYNGLDLAFSPYGAYWREMKKICVVHLLNSTRVQSFRTNREDEVSHMIEKISKAALASKPFNLTEGMLSLTSTAICRTAFGKRYEDGGIEGSRFLALLNETEALFTMFFLSDYFPYMGWVDRLTGRAHRLEKNFREFDVFYQQIIDEHLDPERPKPDHEDILDVLLQIYKDRTFKVQLTLDHIKAILMNIFVGGTDTAAATVIWAMSLLMKNPEAMRKAQEEVRKVIGDKGFVYEDDVQQLPYLKAVVKETMRLQPTAPLLVPRETTTECNIGGYEIPAKTLVYVNAWAIGRDTEVWENPYVFIPDRFLGSSIDLKGQDFELIPFGAGRRICPGIYMGIATVELSLSNLLYKFDWEMPGGMKREDIDVDHTQPGLAMHTRDALCLVPKAYAVMGNDA | |
| CYP83G1v1 | MNKNMSPLILLPFALLLFFLFKKHKTSKKSTTLPPGPKGLPFIGNLHQLDSSVLGLNFYELSKKYGPIISLKLGSKQTVVVSSAKMAKEVMKTHDIEFCNRPALISHMKISYNGLDQIFAPYREYWRHTKKLSFIHFLSVKRVSMFYSVRKDEVTRMIKKISENASSNKVMNMQDLLTCLTSTLVCKTAFGRRYEGEGIERSMFQGLHKEVQDLLISFFYADYLPFVGGIVDKLTGKTSRLEKTFKVSDELYQSIVDEHLDPERKKLPPHEDDVIDALIELKNDPYCSMDLTAEHIKPLIMNMSFAVTETIAAAVVWAMTALMKNPRAMQKVQEEIRKVCAGKGFIEEEDVEKLPYFKAVIKESMRLYPILPILLPRETMTNCNIAGYDIPDKTLVYVNALAIHRDPEVWKDPEEFYPERFIGSDIDLKGQDFELIPFGSGRRICPGLNMAIATIDLVLSNLLYSFDWEMPEGAKREDIDTHGQAGLIQHKKNPLCLVAKKRIECV | |
| CYP84A1 | MESSISQTLSKLSDPTTSLVIVVSLFIFISFITRRRRPPYPPGPRGWPIIGNMLMMDQLTHRGLANLAKKYGGLCHLRMGFLHMYAVSSPEVARQVLQVQDSVFSNRPATIAISYLTYDRADMAFAHYGPFWRQMRKVCVMKVFSRKRAESWASVRDEVDKMVRSVSCNVGKPINVGEQIFALTRNITYRAAFGSACEKGQDEFIRILQEFSKLFGAFNVADFIPYFGWIDPQGINKRLVKARNDLDGFIDDIIDEHMKKKENQNAVDDGDVVDTDMVDDLLAFYSEEAKLVSETADLQNSIKLTRDNIKAIIMDVMFGGTETVASAIEWALTELLRSPEDLKRVQQELAEVVGLDRRVEESDIEKLTYLKCTLKETLRMHPPIPLLLHETAEDTSIDGFFIPKKSRVMINAFAIGRDPTSWTDPDTFRPSRFLEPGVPDFKGSNFEFIPFGSGRRSCPGMQLGLYALDLAVAHILHCFTWKLPDGMKPSELDMNDVFGLTAPKATRLFAVPTTRLICAL | |
| CYP84A1 | MESSISQTLSKLSDPTTSLVIVVSLFIFISFITRRRRPPYPPGPRGWPIIGNMLMMDQLTHRGLANLAKKYGGLCHLRMGFLHMYAVSSPEVARQVLQVQDSVFSNRPATIAISYLTYDRADMAFAHYGPFWRQMRKVCVMKVFSRKRAESWASVRDEVDKMVRSVSCNVGKPINVGEQIFALTRNITYRAAFGSACEKGQDEFIRILQEFSKLFGAFNVADFIPYFGWIDPQGINKRLVKARNDLDGFIDDIIDEHMKKKENQNAVDDGDVVDTDMVDDLLAFYSEEAKLVSETADLQNSIKLTRDNIKAIIMDVMFGGTETVASAIEWALTELLRSPEDLKRVQQELAEVVGLDRRVEESDIEKLTYLKCTLKETLRMHPPIPLLLHETAEDTSIDGFFIPKKSRVMINAFAIGRDPTSWTDPDTFRPSRFLEPGVPDFKGSNFEFIPFGSGRRSCPGMQLGLYALDLAVAHILHCFTWKLPDGMKPSELDMNDVFGLTAPKATRLFAVPTTRLICALXXXCYP | |
| CYP85A1 | MGAMMVMMGLLLIIVSLCSALLRWNQMRYTKNGLPPGTMGWPIFGETTEFLKQGPNFMRNQRLRYGSFFKSHLLGCPTLISMDSEVNRYILKNESKGLVPGYPQSMLDILGTCNMAAVHGSSHRLMRGSLLSLISSTMMRDHILPKVDHFMRSYLDQWNELEVIDIQDKTKHMAFLSSLTQIAGNLRKPFVEEFKTAFFKLVVGTLSVPIDLPGTNYRCGIQARNNIDRLLRELMQERRDSGETFTDMLGYLMKKEGNRYPLTDEEIRDQVVTILYSGYETVSTTSMMALKYLHDHPKALQELRAEHLAFRERKRQDEPLGLEDVKSMKFTRAVIYETSRLATIVNGVLRKTTRDLEINGYLIPKGWRIYVYTREINYDANLYEDPLIFNPWRWMKKSLESQNSCFVFGGGTRLCPGKELGIVEISSFLHYFVTRYRWEEIGGDELMVFPRVFAPKGFHLRISPY | |
| CYP86A1 | MEALNSILTGYAVAALSVYALWFYFLSRRLTGPKVLPFVGSLPYLIANRSRIHDWIADNLRATGGTYQTCTMVIPFVAKAQGFYTVTCHPKNVEHILKTRFDNYPKGPMWRAAFHDLLGQGIFNSDGDTWLMQRKTAALEFTTRTLRQAMARWVNGTIKNRLWLILDRAVQNNKPVDLQDLFLRLTFDNICGLTFGKDPETLSLDLPDNPFSVAFDTATEATLKRLLYTGFLWRIQKAMGIGSEDKLKKSLEVVETYMNDAIDARKNSPSDDLLSRFLKKRDVNGNVLPTDVLQRIALNFVLAGRDTSSVALSWFFWLVMNNREVETKIVNELSMVLKETRGNDQEKWTEEPLEFDEADRLVYLKAALAETLRLYPSVPQDFKYVVDDDVLPDGTFVPRGSTVTYSIYSIGRMKTIWGEDCLEFRPERWLTADGERFETPKDGYKFVAFNAGPRTCLGKDLAYNQMKSVASAVLLRYRVFPVPGHRVEQKMSLTLFMKNGLRVYLQPRGEVLA | |
| CYP86B1 | MNFNSSYNLTFNDVFFSSSSSSDPLVSRRLFLLRDVQILELLIAIFVFVAIHALRQKKYQGLPVWPFLGMLPSLAFGLRGNIYEWLSDVLCLQNGTFQFRGPWFSSLNSTITCDPRNVEHLLKNRFSVFPKGSYFRDNLRDLLGDGIFNADDETWQRQRKTASIEFHSAKFRQLTTQSLFELVHKRLLPVLETSVKSSSPIDLQDVLLRLTFDNVCMIAFGVDPGCLGPDQPVIPFAKAFEDATEAAVVRFVMPTCVWKFMRYLDIGTEKKLKESIKGVDDFADEVIRTRKKELSLEGETTKRSDLLTVFMGLRDEKGESFSDKFLRDICVNFILAGRDTSSVALSWFFWLLEKNPEVEEKIMVEMCKILRQRDDHGNAEKSDYEPVFGPEEIKKMDYLQAALSEALRLYPSVPVDHKEVVQEDDVFPDGTMLKKGDKVIYAIYAMGRMEAIWGKDCLEFRPERWLRDGRFMSESAYKFTAFNGGPRLCLGKDFAYYQMKSTAAAIVYRYKVKVVNGHKVEPKLALTMYMKHGLMVNLINRSVSEIDQYYAKSFDEGYIN | |
| CYP86C1 | MNVLISAVVWVYTHLRLSDVALALVGLFLLSYLREKLVSKGGPVMWPVLGIIPMLALNKHDLFTWCTRCVVRSGGTFHYRGIWFGGAYGIMTADPANVEHILKTNFKNYPKGAFYRERFRDLLEDGIFNADDELWKEERRVAKTEMHSSRFLEHTFTTMRDLVDQKLVPLMENLSTSKRVFDLQDLLLRFTFDNICISAFGVYPGSLETGLPEIPFAKAFEDATEYTLARFLIPPFVWKPMRFLGIGYERKLNNAVRIVHAFANKTVRERRNKMRKLGNLNDYADLLSRLMQREYEKEEDTTRGNYFSDKYFREFCTSFIIAGRDTTSVALVWFFWLVQKHPEVEKRILREIREIKRKLTTQETEDQFEAEDFREMVYLQAALTESLRLYPSVPMEMKQALEDDVLPDGTRVKKGARIHYSVYSMGRIESIWGKDWEEFKPERWIKEGRIVSEDQFKYVVFNGGPRLCVGKKFAYTQMKMVAAAILMRYSVKVVQGQEIVPKLTTTLYMKNGMNVMLQPRDW | |
| CYP86E2 | MTTRALVAMALRFLREYVRASDLAVAAAVLFACSAARSRLSSRPGEPMLWPVVGIIPTLFAHLAAGDVYDWGAAVLLGRSRGTFAYRGTWGGGSSGVVTSVPANVEHVLKANFDNYPKGPYYRERFAELLGGGIFNADGDSWRAQRKAASAEMHSARFVQFSAGTVERLVRRELLPLLESLSGREGPESAAAAVDLQDVLLRFAFDNICAAAFGVEAGCLADGLRDVPFARAFERATELSLTRFYTPPFVWKSKRLLGVGSERALVESARAVREFAERTVADRRAELRKVGDLAGRCDLLSRLMSSPPAAGYSDEFLRDFCISFILAGRDTSSVALTWFFWLLASHPHVEARVLDDVARGGGDVSAMDYLHAALTESMRLYPPVPVDFKEALEDDVLPDGTLVRARQRVIYYTYAMGRDKATWGPDCLEFRPERWLSKSGAFAGGAESPYKYVVFNAGPRLCVGKRFAYMQMKTAAAAVLARFRVEVLPGQEVKPKLNTTLYMKNGLMVRFVRREQRHELGHPLPAAAADAGED | |
| CYP86F1 | MQYLVMSRGDNCTHFHSQNQGQTGLGMCTGPNRMDSWMLTQVMLAGVVTFLVWHVIKYSRIKGPIVWPVFGTTPQFLWNLPRMHDWTTDMLVKHDGTYTSIAPKCTCLTAVATCRPENLEYVLKTNFANYPKGRSFTYPSHDLLGQGIFNTDHDLWKMQRKTASLEFSTRTLRDLMVKANRSSVGQRLLPVLADVARNRAPIDFQDLFLRYTFDNICMVGFGVDPGCLAPGLPTVPFAQAFDLATEGTLTRMVVPEIFWRITRALGWGMEGRLAKAISTIDKFAADVITERRRELNMLKTLNATEYPCDLLSRFMQTTDHEGNPYTDRFLRDVTTNFILAGRDTTAIALSWFFYLITQNPAVEEKILNEIREILQSRRQSGGVGEPDDDDAGRTTQEASLSFEELKQLHYLHAALSESMRLYPSVPIDNKDVTADDFLPDGTFVRKGTRLMYSIYSMGRMESIWGKDCLEYKPERWLRNGVFTPESPFKYAVFNAGPRLCLGKELAYLQMKSVASAILRNYHVKLVPEHKVEYKLSLTLFMKYGLHVTLHPRVTVAY | |
| CYP87A2 | MWALLIWVSLLLISITHWVYSWRNPKCRGKLPPGSMGFPLLGESIQFFKPNKTSDIPPFIKERVKRYGPIFKTNLVGRPVIVSTDADLSYFVFNQEGRCFQSWYPDTFTHIFGKKNVGSLHGFMYKYLKNMVLTLFGHDGLKKMLPQVEMTANKRLELWSNQYSVELKDATASMIFDLTAKKLISHDPDKSSENLRANFVAFIQGLISFPFDIPGTAYHKCLQGRAKAMKMLRNMLQERRENPRKNPSDFFDYVIEEIQKEGTILTEEIALDLMFVLLFASFETTSLALTLAIKFLSDDPEVLKRLTEEHETILRNREDADSGLTWEEYKSMTYTFQFINGTARLANIVPAIFRKALRDIKFKDYTIPAGWAVMVCPPAVHLNPEMYKDPLVFNPSRWEGSKVTNASKHFMAFGGGMRFCVGTDFTKLQMAAFLHSLVTKYRWEEIKGGNITRTPGLQFPNGYHVKLHKKRD | |
| CYP87B6 | MLPIGLCVVSLVIIWITYWIRRWKNPRCNVTLPPGSLGFPLIGESIQFLISCSNSLDLHPFFRKRIQKRYGPLFKTSMLGRQVVVTADPEANHFILEQEGKSVEMCYLDSVAQLCGHDESSAGATGHIHKYLRTLILNHFGYERLRYKLLKKVEAMAHKSLGAWSSQPSVELNRATSQIMLDFISKELFSYDPKGCTESMGDAFIDFLDSLASVPLNIPGTTFHKCLKNQKKTMKILREIVDERCASPEIRRGDFLDYFLEGMKKEAFITKDFIAFVMFGLLFASFESIPIMLSLALKLIMEHPLVLQELEEHEAILRNKDTSNFTLTWEDYKSMTFTVIDETLRMANVGLGNFRKALEDIKIKGHTIPAGWTILVVSSVLHMDPNIYPDPLVFNPWRWKGSXKITTKNFTPFGGGIRFCPGAELSKLTMAIFLHVAVTKYRFTKIKGGNLVRNPVLKFKDGFHIKVSKK | |
| CYP87C6 | MSMHYLAALSVTLLGAILLRWAFKWMNYGRTGGEEGMLLPPGSRGLPFLGETLEFFAASPTLELVPFFKRRLERFGPIFRTNIVGEDMIVSLDPELNARVLQQEERGFQIWYPSSFMRILGADNMVSMLGPLHRHIRNLVLRLFGPEALRLVLLRDVQRSARDELRSWLDRPEVEVRTATSRMIFGVTAKKLISHDDVASGGSLWKCFDAWTKGLMSFPICVPGTAFYRCMQGRKNVMKVLKQQLDERRNGAERKTVDFFDLVIDELDKPNSIMSESIALNLLFLLLFASHETTSMGLTVILKFLTDNPKSLQELTEEHEKIMERRVDPDSDITWEEYKSMKFTSHVIHESLRLANIAPVVFRQANQDVHIKGYTIPEGSKIMICPSAAHLNSKVYEDPLAFNPWRWKDTPEPVGGSKDFMAFGGGLRLCVGAEFAKLQMAMFLHYLVTNYRWKALSKGTMMLYPGLRFPDGFHIQLHKKT | |
| CYP87D1 | MWAIGLVVVALVVIYYTHMIFKWRSPKIEGVLPPGSMGWPLIGETLQFISPGKSLDLHPFVKKRMQKYGPIFKTSLVGRPIIVSTDYEMNKYILQHEGTLVELWYLDSFAKFFNLEGETRVNAIGAVHKYLRSITVNHFGVESLKESLLPKIEDMLHTTLAKWASQGPVDVKQVISVMVFNFTANKIFGYDAENSKETLSKNYTKILNSFISLPLNIPGTSFHKCMQEREKMLKLLKDTLMERLNGPSKRRGDFLDQAIDDMKTKKFLTVDFIPQLMFGILFASFESMSTTLTLTFKFLTENPRVVEELRAEHEAIVKKRENPNSRLTWEEYRSMTFTQMVVNETLRISNIPPGLFRKALKDFQVKGYTVPAGWTVMLVTPAIQLNPDTFKDPVTFNPWRWKDLDQVTISKNFMPFGGGTRQCAGAEYSKLVLSTFLHVLVTSYSFTKVKGGDVSRTPIISFGDGIHIKFTARN | |
| CYP88A3 | MAETTSWIPVWFPLMVLGCFGLNWLVRKVNVWLYESSLGENRHYLPPGDLGWPFIGNMLSFLRAFKTSDPDSFTRTLIKRYGPKGIYKAHMFGNPSIIVTTSDTCRRVLTDDDAFKPGWPTSTMELIGRKSFVGISFEEHKRLRRLTAAPVNGHEALSTYIPYIEENVITVLDKWTKMGEFEFLTHLRKLTFRIIMYIFLSSESENVMDALEREYTALNYGVRAMAVNIPGFAYHRALKARKTLVAAFQSIVTERRNQRKQNILSNKKDMLDNLLNVKDEDGKTLDDEEIIDVLLMYLNAGHESSGHTIMWATVFLQEHPEVLQRAKAEQEMILKSRPEGQKGLSLKETRKMEFLSQVVDETLRVITFSLTAFREAKTDVEMNGYLIPKGWKVLTWFRDVHIDPEVFPDPRKFDPARWDNGFVPKAGAFLPFGAGSHLCPGNDLAKLEISIFLHHFLLKYQVKRSNPECPVMYLPHTRPTDNCLARISYQ | |
| CYP88B1 | MDFYNLALFFIALILGIFTFYAILMRINGWYYAIKFCSNKYNIPNGYMGLPYFGNTLSYFKASMCGDPKSFIDFFATRFGEGGMYRAYIFGKPTIMVTKPEIIRKVLMDEEYLERGLPNYMKKLIGLTTSIEEDKYFRRLTAPVKSHGLLSDYFDYIDKTVSSTLEKYATTEEPVEFLHKMHKLTFEVFMRLLIGDEVNQELFDEMFEEITAVISGVHNLPINLPGFAYHKGLKARKVLXEVFKKLIDERREAMKDGKSMPKANIIDMLLSNNNQDYEANMLSDKKIIEILVLFSFAGFEPVALMSVKAIFHLQKHPHFLEKAKEEQEEIVKRRASSNAGLSFDEIRQMTFVSKIINETLRIATDQSVFLRDTSTTFNINGYTIPKGWKFFAVVWNIHMNPDVYVQPKEFNPSRWDDIETKPGIFLPFSMGPKSCPGSNLAKLQISVILHYYLLHYRVEQINPEARCYPPENCLVKFKKLSISSNGN | |
| CYP88C1 | MEYDSMFLYTALAVGILTIWSILKNGNGWFYTFKFSSNKCRLPPGDMGWPFFGNMLHFVKCLSNYDLASFVSYFVTRFGKGGLYKAYMFGKPTILVTSPELCRKVVMDDENFDLGFPQYILELLRKEPIGGTTNQEDKLARRLTTPIKSHGLVSFFFDFLSENVKTSFEKWSASEKPIELLAEMKKPTFAVLMRVLLGGEELVARELLDVIFKENNFRFAGLRSLPINIPGFAFHRAMKGRKEIIKVFERVINERKVLIAKDKTRAKSNILDIMLSTQDDDGKGLRDGNILKTLLWYTFSGYESVAKVATQTMMLLQNHPECLKKAKEEQEEIVKRRTSPNEGLNFSEIGQMKYVTNVINETLRLGSTETVLFRDARTDVNLNGYTIPEGWKCLALLGNFYKDPDTYVKPNEFIPSRWDDLEVKPASFLPFGVGLRMCPGANLVRLEVAVVLHYFLLNYRLEMLDPDSTPEKCLARFKKLSA | |
| CYP88D3 | MEMQWVYICTAALFACYVFVNKFLRRFNGWYYHLKLRNKEYPLPPGDMGWPLIGNLLSFNKNFSSGQPDSFTTNLILKYGRDGIYKTHVYGNPSIIICDPEMCKRVLLDDVNFKIGYPKSIQELTKCRPMIDVSNANHKHFRRLITAPMVGHKVLDMYLERLEDIAINSLEELSSMKHPIELLKEMKKVSFKSIIHVFMGTSNQNIVKNIGSSFTDLSKGMYSIPINAPGFTFHKALKARKKIAKSLQPVVDERRLIIKNGQHVGEKKDLMDILLEIKDENGRKLEDQDISDLLIGLLFAGHESTATGIMWSVAHLTQHPHILQKAKEEQEEILKIRPASQKRLSLNEVKQMIYLSYVIDEMLRFANIAFSIFREATSDVNINGYLIPKGWRVLIWARAIHMDSEYYPNPKEFNPSRWKDYNAKAGTFLPFGAGSRLCPGADLAKLEISIFLHYFLLNYRLERINPDCPVTTLPQCKPTDNCLAKVIKVSRA | |
| CYP88D4 | MELYWAWVCAATLATCYVLRRLNGWYYDVKLRKKQYPLPPGDMGWPLIGNLIPFYKDFSSGRPNSFINNLLLKYGEGGIYKTHLFGNPSIIVCEPEICMRVLTDDVNFRVGYPTTIKELIRLKHISRAEHKQYRRLVNTLPILDHQALATLYLERIENIVTNSLEELSSMKHPVELLKEMKKVTFKVFIHILMGSSIHHMIIENMDTSFAELTNGILSAPINAPGFVFHKALKARKKLAKILQSVVDERRLRSKNGQEGKDKVFLDNLLEAKDENGRKRDDEYIVDVLIAQLFAGHETSATALMWTILYLTQHPHILEKAKKEQEEIMKARVSSQGRLNLQEIKQMVYLSQVIDETLRCANIVFTTFREAISDVNINGYVIPNGWRVLVWARAVHMNPKYYPNPEEFNPSRWDDYHGKAGTFLPFGAGSRLCPGKDLAKLEISVFLHYFLLNYKLERINAECPITFLPILKPVDNCLAKVIKVS | |
| CYP88D5 | MELYWAWVSAATLATCYVFVDIFLRRLNGWYYDLKLCKKQHPLPPGDMGWPLIGNLISFYKDFSSGHPNSFTNNLLLKYGQSGMYKTHLFGKPSIIVCEAEICRRVLTDDVNFKFAYPESLRQLIPVQSISRAEHRQFRRLINTPIMNHQALAVYLERIENIMINSLEELSSMKHPVELLKEMKKVTFKVIIDILMGTSIPHMITQNMESFFAELCNGMLSAPINAPGFVYHKALKARKKLAKTVQSVVDERRLKSKNGQEGKDKAFIDSVLEVNDENGRKLEDGYIIDLLIAILFAGHETSATTMMWTIVYLTQHPHILNKAKEEQEKIMKVRVSSQTRLNLQEIKQMVYLSQVIDETLRCANIVFSMFREATSDVNMSGYVIPKGWRVLIWGRAVHMDPENYPNPEEFNPSRWDDYHGKAGTSLPFGVGSRLCPGKDLAKLEISIFLHYFLLNYKLERINPDCPITFLPIPKPVDNCLAKVIKVSCN | |
| CYP88D6 | MEVHWVCMSAATLLVCYIFGSKFVRNLNGWYYDVKLRRKEHPLPPGDMGWPLIGDLLSFIKDFSSGHPDSFINNLVLKYGRSGIYKTHLFGNPSIIVCEPQMCRRVLTDDVNFKLGYPKSIKELARCRPMIDVSNAEHRLFRRLITSPIVGHKALAMYLERLEEIVINSLEELSSMKHPVELLKEMKKVSFKAIVHVFMGSSNQDIIKKIGSSFTDLYNGMFSIPINVPGFTFHKALEARKKLAKIVQPVVDERRLMIENGPQEGSQRKDLIDILLEVKDENGRKLEDEDISDLLIGLLFAGHESTATSLMWSITYLTQHPHILKKAKEEQEEITRTRFSSQKQLSLKEIKQMVYLSQVIDETLRCANIAFATFREATADVNINGYIIPKGWRVLIWARAIHMDSEYYPNPEEFNPSRWDDYNAKAGTFLPFGAGSRLCPGADLAKLEISIFLHYFLRNYRLERINPECHVTSLPVSKPTDNCLAKVIKVSCA | |
| CYP88E1 | MNLKWAIAIATVAAATFFELLRNFNRFWYEPKLKPGQAPLPPGSLGWPIFGNMASFLRAFKSHNPDSFITKYLHKYDRTGVYKAFLFWQPTVLATTPETCKVVLSRDSLFETGWPSSTRRLIGTRSFAGVTGEEHLKLRRLTEPALSNPKALEDYIPRMSSNIKSCLEEWSCQERTLLLREMRKYAFRTIHDILFSKDSGLDVEEVSSLYYEGNQGIRSLPINLPGTSYNRALKARKKLDVLLHRVLNKRRFSEKPEKTDTLSLLMDATDENGKHLDDKQIVDLLVMYLNAGHDSTAHLILWLLIFLLKHEIVYDKVKEEQELIASQKPLGDSLSLSDVKKMSYLSRVINETLRVANISPMVFRRAVTDVEVNGFTIPKGWYVEPWLRQVHMDPAVHSNPQNFDPDRWAVIRPFTHLPFGLGSRTCPGNELAKLEACIIVHHLVLGYEVKPLNPDCEVTFLPHPRPKDYFPVQVRRRR | |
| CYP88F1v1 | MDLWLPSIAVALIIVLISCILNFNSWFYAPKLRPGSPPLPPGSLGWPVFGNMGDFLQAFKSSNPESFVGGFISKYGCGGLYKAFLFRQPTILATSAEVCKTVLCNHDVFEIGWPERVVKELLGLKVLSAVTGDDHLKLSKLVKPALSSPKAIQHQMPCIEENVKKLLDEWADRGNIVFLDEARMFTLKTIHEILVGEDTGIDFKQVSGLFHTMNKGLRALPLNFPGTAYSNAVKARATLANDFWRIFYERKESKKRGGDTLSMLLDATDEGGQPLEDDQIVDLIMSFMNGGHESTAHLVTWLAILLKEHPAVYQRLKAEQDEIALKKMPGESLTLADMRSMTYMSRVIDETLRLINISPFVFRKVLSDVQLNGYTIPRGWFVEAWLRQVHMDPLVHKNPREFDPDRWINEKPQPHTYVAFGLGNRKCPGSNLSKIQSSIIIHHLITKYNWEPLNPHYKLVYLPHPRPADHYPVKITKRALV | |
| CYP89A2 | MEIWLLILASLSGSLLLHLLLRRRNSSSPPLPPDPNFLPFLGTLQWLREGLGGLESYLRSVHHRLGPIVTLRITSRPAIFVADRSLTHEALVLNGAVYADRPPPAVISKIVDEHNISSGSYGATWRLLRRNITSEILHPSRVRSYSHARHWVLEILFERFRNHGGEEPIVLIHHLHYAMFALLVLMCFGDKLDEKQIKEVEFIQRLQLLSLTKFNIFNIWPKFTKLILRKRWQEFLQIRRQQRDVLLPLIRARRKIVEERKRSEQEDKKDYVQSYVDTLLDLELPEENRKLNEEDIMNLCSEFLTAGTDTTATALQWIMANLVKYPEIQERLHEEIKSVVGEEAKEVEEEDVEKMPYLKAVVLEGLRRHPPGHFLLPHSVTEDTVLGGYKVPKNGTINFMVAEIGRDPVEWEEPMAFKPERFMGEEEAVDLTGSRGIKMMPFGAGRRICPGIGLAMLHLEYYVANMVREFQWKEVQGHEVDLTEKLEFTVVMKHPLKALAVPRRCH | |
| CYP89B17 | METSWLLLSGALLLSLLVLRLHAKNRRLPPGPPAVPLFGNLLWLRNSAVQVEPLLLKLFKRYGPVVTLRMGSQLTIFVADRRLAHAALVGAGAVTMANRPQAATSSLLGVSDNIITRTDYGPVWRLLRRNLVAETLHPSRVRLFAPARAWVRGVLMDKLRAGGAAGDDEPRDVMEAFRYTMFCLLVIMCFGERLDEPAVRAIQDAERKWLLYISQQMSVFFFFPSVTRHVFRGRLQTARALHRRQTELFVPLINARREYKRLAKDGQAPERETTFQHSYVDTLLDITLPDEEGHRPLTDDEIVRLCSEFFTAGTDTTSTGLQWIMAELVKNPAVQDRLYAEIKATCGDGDAEAVSEEAVHGMPYLKAVILEGLRKHPPGHFVLPHKAAEDMDVGGYLIPKDATVNFMVAVMGRDEQEWERPMEFVPERFLEGGDGAEVDLTGIKGIRMMPFGVGRRICAGMSIAMLHLEYFVASMVREFEWKEAPGH | |
| CYP89C3 | MEDWLFYSLTTLLCLLCSLILRARTPGKKARNADSSSPLPPLPPGPTPLPVLGPLLFLARRDFDIEPVLRRIARDHGKVFTFAPLGRARPGIFVADRGAAHRALVQRGAAFASRPPSTASSAVLTSGGRNVSSSPYGATWRALRRNLASGVLNPARLRAFSPARRWVLGVLARRVRADGRHGEAPVAVMEPFQYAMFCLLVHMCFGGDRLGDDARVRDIEATQRELLGSFLSFQVFSFLPWVTKLVFRRRWEKLVSLRRRQEELFVPLIQARREAGGDGDSYVDSLVKLTIPEDGGRPLTDGEIVSLCSEFLSAGTDTTATALQWILANLVKNPAMQDRLRDEVSSAGAGADGEVLEEELQAMPYLKAVVLEALRRHPPGHYVLPHAVHEDTTLDGYRVPAGAPVNFAVGDIGMDEEVWRAPAEFRPERFLPGGEGDDVDLTGSKEIKMMPFGAGRRVCPGMALALLHLEYFVANLVREFDWRQADGEEVDLTEKLEFTVVMKRPLRARAVPLRPPPPAVAAA | |
| CYP89D1 | MEVILLPLVVIITSTMLLLLIISTAKKRHHGTANLPLPPAPPSVPVVGPLLWLVRARSNLEPAIRELHRRHGPILSLTFLSPRAAIFVSSREVTHRALVQRGHTFASRPPAIAPFAVLTSGQCTVSSAPYGPLWRSLRRNLTSGVLGHGSRAPLYAPARRWALHLLTSDLAAASGNTGGGVAVAVVDCLQFAMFSLLTYMCFGKRLDRRGVREIEAVQRELFSSYISFQVFAFCPTVTKRLFFRRWQKVLSIRRRQEDIFLPLIEERRKRIKISSMDNDGSMVCCYVDTIISHKLPKEAGDRRLTDGELVSLCTEFLTASVDTIVTALQWIMARVVEQPEIQAKLLDEINRVVSSDKEHVDEEDIKSMAYLKALVLEGLRRHPPAHFLLSHAAVEETSLDGHRIPAGRSVNFSVADVAHDENVWSRPEEFLPERFLDGGEGAGTDLTGSREIKMMPFGVGRRICPGLGLALLQLEYFVANMVREFEWGMVDGDCGGGINLAERPEFTVIMEQPLRALVVPRRRE | |
| CYP89E1 | MEETWLFLLFSISLVAVLLATARRRRSSSIKARLPPGPSPLLFLAKFLRLRRSIFDLGPLLRDLHARHGPVISIRLFGTTLVFVADRRLAHRALVQGGSTFADRPPLPELGRLFTSDTRDINSSPYGPYWRLVRRNLASEALSPARVALFAPARRRARDVLVRGLRDRGGDGSRPVELRPLLRRAMFELLLYMSLGARLAPEALEEVERLELWMLRAFTSFPVFSFFPAITKRLFRNQWAAHVAVRRRVGEIYVPLINARRAGDGDGDDPPCYTDSLLQLRVAEEGDRPLTDDEIIALCSEFLNAGTDTTVTLVEWIMAELVNRPDIQAKVHDEVRRRPELTEADLQAMPYLKAVVLEGLRLHPPAQFLLPHGVQSDAEVGGYVVPRGAELNVWVAELGRDEVVWTAAREFMPERFMDGGEVEVDVTGSREITMMPFGVGRRMCPGYTVGTLHAEYLVGSLVRELEWLPETEGEAADMAEELDFTTVMKHPLRARVLPRPSSLY | |
| CYP89F1 | MHAELTSTTTMEMGSLLPHAASLFAVSMASLMIAAVLSIVRRPWPWKTAAISREAVLRLLGVRLGDVPTTVVRDGAVAVDALVRRADAFSDRPAGGGATSIVSGGRAHNINTVPHGPLWVALRRNLTSEAFHPVHGLARAAPGRSSRTSRPPRRAPPAEGQAVRDCLYAALFALNVATCFGDGVDGELVGAMRAAQQEFLRFLPRARVFSTFQKAARLVYPDRWKQLLRHRRRQEEMYLPLIRAINEQRRTRGTPSPPPPTTYVDTLLYLEVPADDGRRRRKLSDGEMVGLVSEYLGAATGTVVAQLEWALANLVRRPDIQTRLCGEVEAAAGGEPCAYLRAVVMECLRRHPPVSSVQRHMVRDVMLGGAHVARGNVVSFAIEEIGRWTSSEEFSPERFMEGGEEGVRLAIGSKQEATTKVKMMPFSAGRRTCPGMGYAILHLEYFLANLVTAFEWRRVPWEEEVDLTADYGFITTMQHPLRALVVPLSNDRSTVV | |
| CYP89G1 | MALLLLLVSSSCLVVAASIAVLCYVNNDADERLPPGPRVRLPLIGNLFLHAPTMAFLPSALRRLRRSHGPVVTLWAGNRPAVFVIGRDFAHRTLVLAGAALAHRPPSPFASSRALSFNRHGVNAAEYGDRWRRLRSNICSCLAATEALRRRSVDRLVATLELEARAGAGATGVVAPTDAFRHGVFSFFAVLCFGEWVRDGEHDAVLRDLRRAHADILALTVELGAFHLVPAVLMVPYLHRWWKLSGLQRSHRDIVAALISVRRLRREKADGDVADSATFCYVDTLLELELGEDEMVSLCWEFMNAAAKTTSTALEWTMARLVHHSDIQRKLRHDIAKTTNSGGVGVSPSPYLKAVVQESLRLHPPAHYLLAHTVDRDVPLGAGGYVIPKGAIVNYAVAEIGRDATAWTDPDEFVPERFMEGGEGAMVDAVSCGGAEIRMMPFGAGRRACPGASFAVSVLHLFVGRLVEQFEWWPVAEDEKAAAVDFSEKTGLVTVMKTPLRALLVPITSS | |
| CYP89H1 | MDDLVTMFPHLSHSRSVTLLFLFLTTAFLLVGCSRKSGAVMLAVLRWLAAPVLTLPWHRASGGRGTRRGLSVQVTDRAVARRALVQHSAAFLDRPTGAVPSTILTRNRHYNILSSPYGPYWRAARRNVATGVLHPSQLRMLGGTRARVLGDLVRALKSGAPAGESLYFAVYSVLAGMCFGEDVVAELGETRLRAMQKFQRDILLALPSFGVFVRYPRIGRFLYRSRWHRLLALRRQQEESFLPLVAAIRNRREASRGNTTLTTYVESLLDLRIHEDGGRAVTDGELVSLISEFLGAGTESTAAALEWTMANLVKSPDLQQKLRLEANAMACGKRVIEEEDLARMPYLRAVVLESLRRHPPVPFVIRRVDGDDAKKVIGVSRLPDGGATVNFLVGKIGRDPAAWSDPMSFKPERFMPGGEGDGTDLTCTTELKMMPFGAGRRVCPGLATAMLHLKYFVANLLTEFEWWEAEDDKVDLTEFRGFFFTVMNRPLQARLVPTDAAAAPWLSN | |
| CYP89J1 | MQHVLVILTVTLVLLVVVVRRYAPSKAVYTRLAASIKSTMARRFRPPAIVIKDRATAHRLLVRGCAGGNFCNRPASLTPTAVVSQLRHHNIITAPYDPFWRVTRRNLTSEVLHPLRLHQYAAARREALRVLVADLRAQCTSNPDGLVLAAESIRNAMFGLLATMCFGDGIDKGLVRAMADAQYEFMQLFPDLRLFARVPALARLIHRKRWSKIIALRRKQEDMYLPLIHARRTRQRQSGETPAYVDTLIDLWVPDEHNAGKRRRQRRLANGELVGLCSEFVGAGTETVAAELQWIMANLVKHPHLQEAVRRETDAAVDANAEEVGEEVLPKLEYLNAVVMEALRLYPTVTLVIRQVMEEDDVVHDSRRIPAGTNVIFRPLSLGRDKTAWANPDEFRPERFLACRGGQSVNLVAAAGSRGGEMSMMPFGAGRRVCPGMGVAMLHTAYFLANLVKEFEWRDAEGELAVDLRPRFAFFTVMERPLRARLLLRSRTQNGQVN | |
| CYP90A1 | MAFTAFLLLLSSIAAGFLLLLRRTRYRRMGLPPGSLGLPLIGETFQLIGAYKTENPEPFIDERVARYGSVFMTHLFGEPTIFSADPETNRFVLQNEGKLFECSYPASICNLLGKHSLLLMKGSLHKRMHSLTMSFANSSIIKDHLMLDIDRLVRFNLDSWSSRVLLMEEAKKITFELTVKQLMSFDPGEWSESLRKEYLLVIEGFFSLPLPLFSTTYRKAIQARRKVAEALTVVVMKRREEEEEGAERKKDMLAALLAADDGFSDEEIVDFLVALLVAGYETTSTIMTLAVKFLTETPLALAQLKEEHEKIRAMKSDSYSLEWSDYKSMPFTQCVVNETLRVANIIGGVFRRAMTDVEIKGYKIPKGWKVFSSFRAVHLDPNHFKDARTFNPWRWQSNSVTTGPSNVFTPFGGGPRLCPGYELARVALSVFLHRLVTGFSWVPAEQDKLVFFPTTRTQKRYPIFVKRRDFAT | |
| CYP90B1 | MFETEHHTLLPLLLLPSLLSLLLFLILLKRRNRKTRFNLPPGKSGWPFLGETIGYLKPYTATTLGDFMQQHVSKYGKIYRSNLFGEPTIVSADAGLNRFILQNEGRLFECSYPRSIGGILGKWSMLVLVGDMHRDMRSISLNFLSHARLRTILLKDVERHTLFVLDSWQQNSIFSAQDEAKKFTFNLMAKHIMSMDPGEEETEQLKKEYVTFMKGVVSAPLNLPGTAYHKALQSRATILKFIERKMEERKLDIKEEDQEEEEVKTEDEAEMSKSDHVRKQRTDDDLLGWVLKHSNLSTEQILDLILSLLFAGHETSSVAIALAIFFLQACPKAVEELREEHLEIARAKKELGESELNWDDYKKMDFTQCVINETLRLGNVVRFLHRKALKDVRYKGYDIPSGWKVLPVISAVHLDNSRYDQPNLFNPWRWQQQNNGASSSGSGSFSTWGNNYMPFGGGPRLCAGSELAKLEMAVFIHHLVLKFNWELAEDDQPFAFPFVDFPNGLPIRVSRIL | |
| CYP90C1 | MQPPASAGLFRSPENLPWPYNYMDYLVAGFLVLTAGILLRPWLWFRLRNSKTKDGDEEEDNEEKKKGMIPNGSLGWPVIGETLNFIACGYSSRPVTFMDKRKSLYGKVFKTNIIGTPIIISTDAEVNKVVLQNHGNTFVPAYPKSITELLGENSILSINGPHQKRLHTLIGAFLRSPHLKDRITRDIEASVVLTLASWAQLPLVHVQDEIKKMTFEILVKVLMSTSPGEDMNILKLEFEEFIKGLICIPIKFPGTRLYKSLKAKERLIKMVKKVVEERQVAMTTTSPANDVVDVLLRDGGDSEKQSQPSDFVSGKIVEMMIPGEETMPTAMTLAVKFLSDNPVALAKLVEENMEMKRRKLELGEEYKWTDYMSLSFTQNVINETLRMANIINGVWRKALKDVEIKGYLIPKGWCVLASFISVHMDEDIYDNPYQFDPWRWDRINGSANSSICFTPFGGGQRLCPGLELSKLEISIFLHHLVTRYSWTAEEDEIVSFPTVKMKRRLPIRVATVDDSASPISLEDH | |
| CYP90D1 | MDTSSSLLFFSFFFFIIIVIFNKINGLRSSPASKKKLNDHHVTSQSHGPKFPHGSLGWPVIGETIEFVSSAYSDRPESFMDKRRLMYGRVFKSHIFGTATIVSTDAEVNRAVLQSDSTAFVPFYPKTVRELMGKSSILLINGSLHRRFHGLVGSFLKSPLLKAQIVRDMHKFLSESMDLWSEDQPVLLQDVSKTVAFKVLAKALISVEKGEDLEELKREFENFISGLMSLPINFPGTQLHRSLQAKKNMVKQVERIIEGKIRKTKNKEEDDVIAKDVVDVLLKDSSEHLTHNLIANNMIDMMIPGHDSVPVLITLAVKFLSDSPAALNLLTVEENMKLKSLKELTGEPLYWNDYLSLPFTQKVITETLRMGNVIIGVMRKAMKDVEIKGYVIPKGWCFLAYLRSVHLDKLYYESPYKFNPWRWQERDMNTSSFSPFGGGQRLCPGLDLARLETSVFLHHLVTRFWIAEEDTIINFPTVHMKNKLPIWIKRI | |
| CYP90E1v2 | MISSSTAWAWTSLAGVAGVFWLAALVYWRSWRFRKLQRLPPGSMGWPLIGELVPYVTIVRSETPFRFTRERESKYGPVFKTSLLTGKTVMITDVEGVKFVLHNEGVLFETGYPRSLKDVLGEHAMLFQHGDLQKRMHAMLKRFVSSTPLKKHLTREMELLTMQGMSTWSRGQRILLQDEIQRITHDFLMKQLFGLEPGKLSTTILKEFHTLMAGIIGIPMMIPGTPYFKAMKAREKLSKIIMDMVATRRAKPDIEHKDILNALIEEVKQEDGDMEKIIIDNVLVNIANAENVPAVVIALAVKNLSETPKALEQIREENLAIRKGKDPSEGLSWNEYMSLEFTQAVFNETLRLANGAQGVMRKALKDVEYRGYIIPKGWTVLPYFLNIHFDENMFPNSAKFHPWRWLEKNIPPSYVLPFGGGSRLCPGQELAKVQTAVFLHHLVTQFKWDAEPEKVINFPMISTRNHVPVVLYDLN | |
| CYP90F1v1 | MNLPPGRMGWPLVGETLEYLATRPIGVPQPFIAKRVARYGSIFKTHLFGCPTIVTTDPDFNRFVLANEGKLFQSSYPAGVDRVLGKFSMVQASGELHKRMRALTVSFMQAQSLKDNFLQTIQARVISLLSTWEGRVVKIQDEAQSLSFDCIVGHVLGMDPGAENTKTIKEDFFNLVYGLTIPLRIPGTRYWTAMKGRQNIVRLVEQMVAERTTKPCTARKDFLQQLLQDDNGKNLTLEQISDFIVFMLFAAHDTTATAMTMAIKYLLANPQALNQLQEEHLEIRRNKRSPDEPLEWNDYLQMTFTQHVINETLRLTNVLTSAHRIALQDVQTEEGYVIPKGWKVVSSWTTIHLNPKLYAEPLEFNPWRWKTQSVKYFTPFSGGPRFCTGSELARLEIALLLHFIITKYSLHPAEDDEAVYFGTVKMRKGLPVTVTKLSQIL | |
| CYP92A1 | MEPATWAVFLGIALCAAAALFLSRGRRPVYNPPPGPKPWPIIGNLNLMGELPHRSMNELSKRYGPLMQLRFGSLPVLVGASVEMAKLFLKTNDAAFSDRPRFAIGKYTAYDFSDLLWAPSGPYLRQARRICATELFSATRLESFEHIRDEEVRVMLRQLRQAAGRTVRLRDYLQMLALGVISRIVLGNKYVMEEVADGEGDSAPAITPAEFREMVDEFFALHGAFNIGDYIPWLDWLDLQGYVARMKRMKARFGRFLERVLDVHNERRLREGGNFVAKDMLDVLLQLADDTSLEVQLSRDNVKAITQDLIIAGTDSNANTLEWAVSELLKNPKILAKAMEELNHVIGPDRLVTESDLPRLPYIEAVLKETMRVHPAAPMLAPHVAREDTSVDGYDVLAGTVLFINVWAIGRDPGLWDAPEEFRPERFVESKIDVRGHDFQLLPFGSGRRMCPGINLALKVMALSLANLLHGFEWRLPDGVTAEELSMDEAFKLAVPRKFPLMVVAEPRLPARLYTGA | |
| CYP92A1 | MEPATWAVFLGIALCAAAALFLSRGRRPVYNPPPGPKPWPIIGNLNLMGELPHRSMNELSKRYGPLMQLRFGSLPVLVGASVEMAKLFLKTNDAAFSDRPRFAIGKYTAYDFSDLLWAPSGPYLRQARRICATELFSATRLESFEHIRDEEVRVMLRQLRQAAGRTVRLRDYLQMLALGVISRIVLGNKYVMEEVADGEGDSAPAITPAEFREMVDEFFALHGAFNIGDYIPWLDWLDLQGYVARMKRMKARFGRFLERVLDVHNERRLREGGNFVAKDMLDVLLQLADDTSLEVQLSRDNVKAITQDLIIAGTDSNANTLEWAVSELLKNPKILAKAMEELNHVIGPDRLVTESDLPRLPYIEAVLKETMRVHPAAPMLAPHVAREDTSVDGYDVLAGTVLFINVWAIGRDPGLWDAPEEFRPERFVESKIDVRGHDFQLLPFGSGRRMCPGINLALKVMALSLANLLHGFEWRLPDGVTAEELSMDEAFKLAVPRKFPLMVVAEPRLPARLYTGA | |
| CYP92A15 | MEVQELVPSPWSSSSSFLVLVLATLLFVAAFLRRRQGARRKYNIPPGPRPWPVIGNLNLIGALPYRSIRDLSRRYGPLMSLRFGSFPVVVGSSVDMARYFLRANDLAFLDRPRTAAGRYTVYNYAGVLWSHYGEYWRQARRLWVTELLSARRLASTEHVRAEEVRAMLRGLSRRAGAGTAVVLKEHMLMVTLNVISRMVFGKKYIVEEGEGSSPTTAEEFRWMIEEIFFLNGVFNIGDMVPWLGWLDPQGYIGRMKRLGGMFDRFLEHILDEHVERRRREGDGFAARDMVDLLLQFADDPSLKVPIQRDGVKAFILELITGSTDTTSVSVEWAMSEVLRNPSVLARATDELDRVVGRRRLVAEGDIPNLPYLDAVVKESMRLHPVVPLLVPRVSREDAFSVSVAGAAASYDIPAGTRVLVNVWAIGRDPAVWGDDAEEFRPERFAAGGERGGVDVKGQDFELLPFGSGRRMCPGFGLGLKMVQLTLANLLHGFAWRLPGGAAAEELSMEEKFGISVSRLVQLKAIPEPKLPAHLYDE | |
| CYP92A15 | MEVQELVPSPWSSSSSFLVLVLATLLFVAAFLRRRQGARRKYNIPPGPRPWPVIGNLNLIGALPYRSIRDLSRRYGPLMSLRFGSFPVVVGSSVDMARYFLRANDLAFLDRPRTAAGRYTVYNYAGVLWSHYGEYWRQARRLWVTELLSARRLASTEHVRAEEVRAMLRGLSRRAGAGTAVVLKEHMLMVTLNVISRMVFGKKYIVEEGEGSSPTTAEEFRWMIEEIFFLNGVFNIGDMVPWLGWLDPQGYIGRMKRLGGMFDRFLEHILDEHVERRRREGDGFAARDMVDLLLQFADDPSLKVPIQRDGVKAFILELITGSTDTTSVSVEWAMSEVLRNPSVLARATDELDRVVGRRRLVAEGDIPNLPYLDAVVKESMRLHPVVPLLVPRVSREDAFSVSVAGAAASYDIPAGTRVLVNVWAIGRDPAVWGDDAEEFRPERFAAGGERGGVDVKGQDFELLPFGSGRRMCPGFGLGLKMVQLTLANLLHGFAWRLPGGAAAEELSMEEKFGISVSRLVQLKAIPEPKLPAHLYDE | |
| CYP92B3 | MENSWVVLALTGLLTLVFLSKFLHSPRRKQNLPPGPKPWPIVGNIHLLGSTPHRSLHELAKRYGDLMLLKFGSRNVLILSSPDMAREFLKTNDAIWASRPELAAGKYTAYNYCDMTWARYGPFWRQARRIYLNEIFNPKRLDSFEYIRIEERHNLISRLFVLSGKPILLRDHLTRYTLTSISRTVLSGKYFSESPGQNSMITLKQLQDMLDKWFLLNGVINIGDWIPWLAFLDLQGYVKQMKELHRNFDKFHNFVLDDHKANRGEKNFVPRDMVDVLLQQAEDPNLEVKLTNDCVKGLMQDLLAGGTDTSATTVEWAFYELLRQPKIMKKAQQELDLVISQDRWVQEKDYTQLPYIESIIKETLRLHPVSTMLPPRIALEDCHVAGYDIPKGTILIVNTWSIGRNSQHWESPEEFLPERFEGKNIGVTGQHFALLPFGAGRRKCPGYSLGIRIIRATLANLLHGFNWRLPNGMSPEDISMEEIYGLITHPKVALDVMMEPRLPNHLYK | |
| CYP92C5 | MELASTMSVAMALAAAIFVVLCSVVASARGRREKALKLPPGPRGWPVLGSLGALAGALPPHRALAALAARHGPLMHLRLGSYHTVVASSADAARLVLRTHDSALADRPDTAAGEITSYGYLGIVHTPRGAYWRMARRLCATELFSARRVESFQDVRAQEMRALARGLFGCAAGRRAVAVREHVAGATMRNILRMAVGEKWSGCYGSPEGEAFRRSLDEAFAATGAVSNVGEWVPWLGWLDVQGFKRKMKRLHDLHDHFYEKILVDHEERRRLAQASGGEFVATDLVDVLLQLSEESTKLESESEARLPRDGVKALIQDIIAGGTESSAVTIEWAMAELLRHPEAMAKATDELDRVVGSGRWVAERDLPELHYIDAVVKETLRLHPVGPLLVPHYARERTVVAGYDVPAGARVLVNAWAIARDPASWPDAPDAFQPERFLGAAAAVDVRGAHFELLPFGSGRRICPAYDLAMKLVAAGVANLVHGFAWRLPDGVAAEDVSMEEHVGLSTRRKVPLFXVXEPRLPVHF | |
| CYP93A4 | MADIQGYIILFLLWLLSTILVRAILNKTRAKPRLPPSPLALPIIGHLHLLAPIPHQALHKLSTRYGPLIHLFLGSVPCVVASTPETAKEFLKTHENSFCDRPKSTAVDFLTYGSADFSFAPYGPYWKFMKKICMTELLGGRMLDQLLPVKHEEIRQFLQFLLKKANARESIDVGSQLIRLTNNVISRMAMSQRCSDNDDEADEVRNLVHEVADLTGKFNLSDFIWFCKNLDLQGFGKRLKEVRKRFDTMTERIIMEHEEARKKKKETGEGDPVKDLLDILLDISEDDSSEMKLTRENIKAFILDIFAAGTDTSAVTMEWALAELINNPNILERAREEIDSVVGQSRLVQESDIANLPYVQAILKETLRLHPTGPIILRESSESCTINGYEIPARTRLFVNVWAINRDPNYWENPLEFEPERFLCAGENGKSQLDVRGQHFHFLPFGSGRRGCPGTTLALQMVQTGLAAMIQCFDWKVNGTVDMQEGTGITLPRAHPLICVPVARLNPFPSFCYPBVGLYCYRRHIZAECHINATAMEPQLVAVSVLVSALICYFFFRPYFHRYGKNLPPSPFFRLPIIGHMHMLGPLLHQSFHNLSHRYGPLFSLNFGSVLCVVASTPHFAKQLLQTNELAFNCRIESTAVKKLTYESSLAFAPYGDYWRFIKKLSMNELLGSRSINNFQHLRAQETHQLLRLLSNRARAFEAVNITEELLKLTNNVISIMMVGEAEEARDVVRDVTEIFGEFNVSDFIWLFKKMDLQGFGKRIEDLFQRFDTLVERIISKREQTRKDRRRNGKKGEQESGDGIRDFLDILLDCTEDENSEIKIQRVHIKALIMDFFTAGTDTTAISTEWALVELVKKPSVLQKVREEIDNVVGKDRLVEESDCPNLPYLQAILKETFRLHPPVPMVTRRCVAECTVENYVIPEDSLLFVNVWSIGRNPKFWDNPLEFCPERFLKLEGDSSGVVDVRGSHFQLLPFGSGRRMCPGVSLAMQEVPALLGAIIQCFDFQVVGPKGEILKGDDIVINVDERPGLTAPRAHNLVCVPVERRSGGGPLKIIEC | |
| CYP93C4 | MLLELALGLCVLAWFLHLRPTPSAKSKALRHLPNPPSPKPRPPFIGHLHLLKDKLLHYALIDLSKKHGPLFSLSFGTMATVGGSTPELFKLFLQTHEGTSFNTRFQTSAIRRLTYDNSVAMVPFGPYWKFVRKLIMNDLLNATTDNKLRPLRTQQIRKFLRVMAQSAEAQKPLDVTEELLKWTNSTISMMMLGEAEMIRDIAREVLKIFGEYSLTDFIWPLKYLKVGKYEKRIDDILNKFDPVVERVIKKRREIVRRRKNGEVVEGEASGVFLDTLLEFAEDETMEIKITKEQIKGLVVDFFSAGTDSTAVATEWALAELVRRSTAVVGKDRLVDEVDTQNLPYIRAIVKETFRMHPPLPVVKRKCTEECEINGYVIPEGALVLFNVWQVGRDPKYWDRPSEFRPERFLETGAEGEAGPLDLRGQHFQLLPFGSGRRMCPGVNLATSGMATLLASLIQCFDLQVLGPQGQILKGDDPKVSMEERAGLTVPRAHSLVCVPLARIGVASKLLS | |
| CYP93D1 | MVDLQYFSVIILVCLGITVLIQAITNRLRDRLPLPPSPTALPIIGHIHLLGPIAHQALHKLSIRYGPLMYLFIGSIPNLIVSSAEMANEILKSNELNFLNRPTMQNVDYLTYGSADFFSAPYGLHWKFMKRICMVELFSSRALDSFVSVRSEELKKLLIRVLKKAEAEESVNLGEQLKELTSNIITRMMFRKMQSDSDGGEKSEEVIKMVVELNELAGFFNVSETFWFLKRLDLQGLKKRLKNARDKYDVIIERIMEEHESSKKNATGERNMLDVLLDIYEDKNAEMKLTRENIKAFIMNIYGGGTDTSAITVEWALAELINHPEIMKKAQQEIEQVVGNKRVVEESDLCNLSYTQAVVKETMRLHPGGPIFVRESDEECAVAGFRIPAKTRVIVNVWAIGRDSNQWEDPLEFRPERFEGSEWKVMSEKMMSFGAGRRSCPGEKMVFRFVPIILAAIIQCFELKVKGSVDMDEGTGSSLPRATPLVCVPVAKEATQSFSLLEPNVNF | |
| CYP93E1 | MLDIKGYLVLFFLWFISTILIRSIFKKPQRLRLPPGPPISVPLLGHAPYLRSLLHQALYKLSLRYGPLIHVMIGSKHVVVASSAETAKQILKTSEEAFCNRPLMIASESLTYGAADYFFIPYGTYWRFLKKLCMTELLSGKTLEHFVRIRESEVEAFLKRMMEISGNGNYEVVMRKELITHTNNIITRMIMGKKSNAENDEVARLRKVVREVGELLGAFNLGDVIGFMRPLDLQGFGKKNMETHHKVDAMMEKVLREHEEARAKEDADSDRKKDLFDILLNLIEADGADNKLTRESAKAFALDMFIAGTNGPASVLEWSLAELVRNPHVFKKAREEIESVVGKERLVKESDIPNLPYLQALLKETLRLHPPTPIFAREAMRTCQVEGYDIPENSTILISTWAIGRDPNYWDDALEYKPERFLFSDDPGKSKIDVRGQYYQLLPFGSGRRSCPGASLALLVMQATLASLIQCFDWIVNDGKNHHVDMSEEGRVTVFLAKPLKCKPVPRFTPFAA | |
| CYP93F1 | MDHQLVARGLFKPLLLFVAGLIVLYALRRRRRHRRSSGLRLPPSPFGLPILGHLHLLAPLPHQALHRLAARHGPLLFLRLGSVPCVAACSPDAAREVLKTHEAAFLDRPKPAAVHRLTYGGQDFSFSAYGPYWRFMKRACVHELLAGRTLDRLRHVRREEVARLVGSLRASADGGERVDVDAALMGLTGDIVSRMVMGRRWTGDDNDAEEMRSVVAETAELTGTFNLQDYIGVFKYWDVQGLGKRIDAVHRKFDAMMERILTAREAKRKLRRQAAADGEDDEKDLLDMLFDMHEDEAAEMRLTRDNIKAFMLDIFAAGTDTTTITLEWALSELINNPPVLRKLQAELDAVVGGARLADESDIPSLPYLQAVAKETLRLHPTGPLVVRRSLERATVAGYDVPAGATVFVNVWAIGRDAAWWPEPTAFRPERFVSGGGGGGTAADVRGQHFHLLPFGSGRRICPGASLAMLVVQAALAAMVQCFEWSPVGGAPVDMEEGPGLTLPRKRPLVCTVSPRIHPLPAAASASLT | |
| CYP93G1 | MASLMEVQVPLLGMGTTMGALALALVVVVVVHVAVNAFGRRRLPPSPASLPVIGHLHLLRPPVHRTFHELAARLGPLMHVRLGSTHCVVASSAEVAAELIRSHEAKISERPLTAVARQFAYESAGFAFAPYSPHWRFMKRLCMSELLGPRTVEQLRPVRRAGLVSLLRHVLSQPEAEAVDLTRELIRMSNTSIIRMAASTVPSSVTEEAQELVKVVAELVGAFNADDYIALCRGWDLQGLGRRAADVHKRFDALLEEMIRHKEEARMRKKTDTDVGSKDLLDILLDKAEDGAAEVKLTRDNIKAFIIDVVTAGSDTSAAMVEWMVAELMNHPEALRKVREEIEAVVGRDRIAGEGDLPRLPYLQAAYKETLRLRPAAPIAHRQSTEEIQIRGFRVPAQTAVFINVWAIGRDPAYWEEPLEFRPERFLAGGGGEGVEPRGQHFQFMPFGSGRRGCPGMGLALQSVPAVVAALLQCFDWQCMDNKLIDMEEADGLVCARKHRLLLHAHPRLHPFPPLL | |
| CYP93H1 | MAFEILFYIILIFSLSSIFGRKFLSAKSQKCISNRLPPGPKGLPIIGHLHLLNNTPHQTFHNLCSRYGPFIHVRLGSVFCIVASSAEYAKETLVTNGLAFASRSVNIASDLLTYGSAGFGFAPYGPQWKFMKKLVTTELLSDKNMTQLKYVRSDEASQLVQLLLDNATSGTVVNVSNEVTMLSNNIISRMMWNIRCSGEDEDGKEIISIIRECTEILAQFNLSDFIPFLGKIDLQGVRKRAMNIHLRYDAILEIIIKKRHEERRKNKERNMQDAGGDNGDDHNSNNFLNILLDAMEDENAKTPVTIENIKALMFDFLNAGTDTSATVVEWSLSELINHPTIMAKARQEIDTIVGKDRLVQESDLPNLPYLQAIFKESLRLHPPVTLFGRESIQDCKIGGYDIPAKTVLFLNIWSINRDPNYWKTPLEFRPERFMPHSDQKEGDDNEYLLEYRGQHFNYLPFGAGRRGCPGMSLAALISPRVLALLIQCFDWKIACNDKGVAPKLVDLTERPGLTVPKLHPLMLIPSVRLNPFPISL | |
| CYP94A1 | MFQFHLEVLLPYLLPLLLLILPTTIFFLTKPNNKVSSTSTNNNIITLPKSYPLIGSYLSFRKNLHRRIQWLSDIVQISPSATFQLDGTLGKRQIITGNPSTVQHILKNQFSNYQKGTTFTNTLSDFLGTGIFNTNGPNWKFQRQVASHEFNTKSIRNFVEHIVDTELTNRLIPILTSSTQTNNILDFQDILQRFTFDNICNIAFGYDPEYLTPSTNRSKFAEAYEDATEISSKRFRLPLPIIWKIKKYFNIGSEKRLKEAVTEVRSFAKKLVREKKRELEEKSSLETEDMLSRFLSSGHSDEDFVADIVISFILAGKDTTSAALTWFFWLLWKNPRVEEEIVNELSKKSELMVYDEVKEMVYTHAALSESMRLYPPVPMDSKEAVNDDVLPDGWVVKKGTIVTYHVYAMGRMKSLWGDDWAEFRPERWLEKDEVNGKWVFVGRDSYSYPVFQAGPRVCLGKEMAFMQMKRIVAGIVGKFKVVPEAHLAQEPGFISFLSSQMEGGFPVTIQKRDS | |
| CYP94B1 | MEMLNAIILILFPIIGFVLIFSFPTKTLKAKTASPSNPTSYQLIGSILSFNKNRHRLLQWYTDLLRLSPSQTITVDLLFGRRTIITANPENVEHILKTNFYNFPKGKPFTDLLGDLLGGGIFNSDGELWSSQRKLASHEFTMRSLREFTFEILREEVQNRLIPVLSSAVDCGETVDFQEVLKRFAFDVVCKVSLGWDPDCLDLTRPVPELVKAFDVAAEISARRATEPVYAVWKVKRFLNVGSEKRMREAIKTVHLSVSEIIRAKKKSLDIGGDVSDKQDLLSRFLAAGHGEEAVRDSVISFIMAGRDTTSAAMTWLFWLLSQNDDVETKILDELRNKGSLGLGFEDLREMSYTKACLCEAMRLYPPVAWDSKHAANDDILPDGTPLKKGDKVTYFPYGMGRMEKVWGKDWDEFKPNRWFEEEPSYGTKPVLKSVSSFKFPVFQAGPRVCIGKEMAFTQMKYVVGSVLSRFKIIPVCNNRPVFVPLLTAHMAGGLKVKIKRREQCDSMYI | |
| CYP94C1 | MLLIISFTIVSFFFIIIFSLFHLLFLQKLRYCNCEICHAYLTSSWKKDFINLSDWYTHLLRRSPTSTIKVHVLNSVITANPSNVEHILKTNFHNYPKGKQFSVILGDLLGRGIFNSDGDTWRFQRKLASLELGSVSVRVFAHEIVKTEIETRLLPILTSFSDNPGSVLDLQDVFRRFSFDTISKLSFGFDPDCLRLPFPISEFAVAFDTASLLSAKRALAPFPLLWKTKRLLRIGSEKKLQESINVINRLAGDLIKQRRLTGLMGKNDLISRFMAVVAEDDDEYLRDIVVSFLLAGRDTVAAGLTGFFWLLTRHPEVENRIREELDRVMGTGFDSVTARCDEMREMDYLHASLYESMRLFPPVQFDSKFALNDDVLSDGTFVNSGTRVTYHAYAMGRMDRIWGPDYEEFKPERWLDNEGKFRPENPVKYPVFQAGARVCIGKEMAIMEMKSIAVAIIRRFETRVASPETTETLRFAPGLTATVNGGLPVMIQERS | |
| CYP94D1 | MALLIFIFLLCFPISIFFIFFTKKSSSEFGFKSYPIVGSFPGLVNNRHRFLDWTVETLSRCPTQTAIFRRPGKQQLIMTANPSNVEYMLKTKFESFPKGQQFTSVLEDFLGHGIFNSDGDMWWKQRKTASYEFSTKSLRDFVMSNVTVEINTRLVPVLVEAATTGKLIDLQDILERFAFDNICKLAFNVDCACLGHDGAVGVNFMRAFETAATIISQRFRSVASCAWRIKKKLNIGSERVLRESIATVHKFADEIVRNRIDQGRSSDHKEDLLSRFISKEEMNSPEILRDIVISFILAGRDTTSSALSWFFWLLSMHPEVEDKILQELNSIRARTGKRIGEVYGFEHLKMMNYLHAAITESLRLYPPVPVDIKSCAEDNVLPDGTFVGKGWAITYNIFAMGRMESIWGKDCDRFDPERWIDETNGCFRGEDPSKFPAFHAGPRMCVGKDMAYIQMKSIVAAVLERFVVEVPGKERPEILLSMTLRIKGGLFARVQERS | |
| CYP94E4 | MEAIHLAYVLVFLLPILLLRLRRRGPPPVKRPRTTTAHCPHPSPVLGNTLHFIRNRRRFFDWYADMLRAAPSGAIEAWGPLGAGHAVTTASPADVDHLLRSSFDKYAKGALFRDATADLIGDGLFAADGRLWSLQRKLASHAFSSRSLRRFTDGVLDVHLRRRFLPLLDAAARDGGAVDLQDALRRFGFRTICHVAFGVEGLDDDARRQDALFAAFDAAVEISFRRALTPATFVRRLTKLLDVGKSRRLREAVHAIDDYAMSVVESKVARRRNSLDDGAADLLSRFMAAMDDGGGSDSELGAMFPTPAAKLRFLRDVVVTFVLAGKDTTSSALTWFFWLLAANPRCERRAHEEAASCCGDGGDVKGMHYLHAAITEAMRLYPPVPFNGRVAVRDDVLPSGAALRAGWYANYSAYAMGRMEKLWGKDCLEFVPERWLGEGGEFVPVDAARFPVFHAGPRVCLGKEMAYVQMKTVAAAVLRRFRLDVVAPVANMEAPPAYEMTATMKMKGGLLVRLCSRED | |
| CYP94F2 | MMDLNAAALFFCLCLSFIFFRSLRSPPTKNSCPHSYPIIGNLIALLRNRHRFHDWVADMLSRTPSLTLQVNTFLNASHGVCTANPLNVNHLLVSNFPNYIKGSRFHDFFHELLGDGIFNVDGHLWTVQRKISSHEFNTKSLKHFISDTVQSELSTRLIPFLSSACENNQVIDLQDVLRKAMFDNICNLAFGADPACLSSEAVGENSLNLSFVQAFDDAVEISASRSLLPIHVIWKIKRFFNIGSEKRLKEAVGIINEYANMILKSKEDQIGSGDCGNLDLLSRFMSSSSNFGLGFDDQEHKRKFLRDIVISFILAGKDSTSTALTWFFWLMAGNPRCEGLILAELSEASPAPATSPVIFSYDDLKGLNYLHAAISESLRLFPPVPIDSRLAVDDDILPDGTHVRKGWFADYSAYAMGRMHQVWGPDCREYRPERWLDDDGRFRPSDQFRFPVFHCGPRLCLGKEMAYVQMKSIAASVMREFEIVAVDGGGCAGKMADPPYTPSIVLKMRGGLPVRVKRRRQPNAIDFC | |
| CYP94G1 | MMDRELVTLLYTAGILLVVTLWCIWYHHPKYGKNRGPKVYPLLGSYLSLLHNKSRILDWMVDLIRDSPTMTVRTVRPGGRQFHITAGPANVEHILKTNFENYPKGENSYANLHDLLGNGIFNIDGKSWKLQRKVASHEFTTQSLKNFMVGAVHDELRGRFIPVLQECCNTGRTVDLQDLLARFTFDTICKLGFGVDPACLDLCFPSVRFANAFDTATSITANRFITFASVWKTMRALNVGSEKKLRAAVADIDDFAMFVIQNRRKQVAGQSNRQTDNSSDADDAAHLDLLSRFMGLTAADQDRRDFDTQDPSCDQNEGPQLGYSDEFLRDIVISFILAGRDTSTSSLTWFFWNLEHHRQVEDAICKEVSEILKNRLVEDKDHNKHVPTSFFSFEELKKMHYLHAAVSESLRLYPPVPIEMKLAHSSDEWPDGTRIDPNSTIIYHPYAMGRMERIWGPDCMKFKPERWLKDGVFVQESPYKHAVFQAGPRMCLGRELALMQIKMVVAVLLQRFRFSSQKGFTPEYDLNLTLPMKNGFPVSVQSKVPM | |
| CYP94H1 | MSTPNYMSPEMGRFEKWALLLREESEEHTLAFVATILFVAVNALIFIWWHHPLYGKNIGPRVYPFVGSLPSAIQHAHRLLDFSVETLRKSPTLTIRYVQSGYTAYSTANVENVEYVLKTKFDNFVKGERMGDVLFDLLGRGIFNADGNLWKLQRKLASHEFSSRSLREFGVECVQKELQNRLVPVLSQFSENGNVVDLQDLLMRFSFDNICQLGFGVDPNCLEPSLPPVKFAEAFDKANECTLLRFRTFPIMLRLYKFFNIGIERGLKESMAVVHNFAQEVIEARRKEFNENHGDIGHARQDLLSRDAKEKQKASDIFLRDMVISFVLAGRDTTSLGLSWFFYALGHNPHVEAKIYDEIKEQLQLQAQEDDSLPSSRPPGQLFTFEQLKKLHYLHAALHESLRLFPPVPWDSKHAVRDDVLPDGTVILKGERVTFNIYAMARMEANWGPDCNEFKPERWLKDGVFVPESPFKFATFQAGPRICLGKEMALIQMKLVASSLVYCFKFTLLEDPPRTCLSFVFKMLNGFPGDVHKRAVST | |
| CYP94J1v1 | MEQGTLAAALLISCVGFFVWFYLKNRHENGETVPRMYPLLGTMPELLKNKDRILEWTTEYLAKSPGHTITLKRWGAKPFKLTSNAQNVEYILKTNFDNYPKGEYVCDTLRDLLGDGIFNADAGLWKLQRKLASYEFTTRSLHDYLMDSVAEKIEKRLLPTIASICGRRVDLQDVFMRFAFDSICKLAFGVDPMSLDPSFPTIAFARAFDESTRLSTERFYQVHPLLWKIKRYFNLGSEKHLKEYLAIVNEFAAMVIKNRRKKTGARENQDLLSRFMALEMEDTASSYSDKFLRDIIISFVLAGKDTTSVTLSWFFWLLSKHPKVENKIIQEIVDVAERNHEPGRRMKHFAYSELREMNYLQAALSESLRLYPAVPFDSKGAKGPDVLPDGSRIEKGTRVTYQIYAMGRMESLWGKDCLEFKPERWLSSTGSFVNESPYKFTAFQAGPRICIGKEMAMLQMKSLVAALLPKFKFEMASDTEPRYSINMTLAIKNGLPVIPRARE | |
| CYP96A1 | MALITLLEVSISLLFFSFLYGYFLISKKPHRSFLTNWPFLGMLPGLLVEIPRVYDFVTELLEASNLTYPFKGPCFGGLDMLITVDPANIHHIMSSNFANYPKGTEFKKIFDVLGDGIFNADSELWKDLRKSAQSMMTHQDFQRFTLRTIMSKLEKGLVPLLDYVAEKKQVVDLQDVFQRFTFDTSFVLATGVDPGCLSTEMPQIEFARALDEAEEAIFFRHVKPEIVWKMQRFIGFGDELKMKKAHSTFDRVCSKCIASKRDEITNGVINIDSSSKDLLMCYMNVDTICHTTKYKLLNPSDDKFLRDMILSFMLAGRDTTSSALTWFFWLLSKNPKAITKIRQEINTQLSPRTNDFDSFNAQELNKLVYVHGALCEALRLYPPVPFQHKSPTKSDVLPSGHRVDASSKIVFCLYSLGRMKSVWGEDASEFKPERWISESGRLIHVPSFKFLSFNAGPRTCLGKEVAMTQMKTVAVKIIQNYEIKVVEGHKIEPVPSIILHMKHGLKVTVTKRSNLL | |
| CYP96B2 | MAFSSILQLTLCFLCFSVFYYYHIKSKRKNPAIPVCWPLVGMLPDLLVNRHQLHDWITSFLTASQLNFRFIGPTMSSNMRFFFTCDPANVRHIFTSNFANYPKGPDFAEIFDDTLGDGIFNVDGDSWRRQRAKTQLLMYNHRFQSFVSRCSSDKVENALLPLLSHFAGTGERCNLQDVFMRLTFDMSTMLASGEDPGCLAISLPMPKVPFVRAVDYTTRVLLVRHIIPLSLWKLARRLGVGFERKMAEALRTINQFIYETIVKRRAKKATEGIEDSEDLLSSYLKDDDENADTFLRDTTMTLIAAGRDTIGSALSWFFYLLTKNPHVASKILEELDSVERATTTPDGMVTFDPDELKSLVYLHAAVCESLRLYPPVPLDHKGVVAADVMPSGHKVRPGDKIVVSIYAMGRTESVWGSDCMEFRPERWISDHGKLRYVPSYKFTPFITGPRTCLGKDMALVQLKVVAATVVKNFEAEAVPGHIVEPKLSMVLHMKNGLMVRVKRR | |
| CYP96C1 | MALASIEKVATGLICFILLIFSLKRIGHPRDWALVGILSVWFPSLGHIYEKLAKSLAKNDKTFVLKGSFLSKQDVIFTCDPANMHHVMSTNFSNYPKGPENRNVFDVYGEMLFTADHEKWKSHRKVTNAYFHDQRFNGFSQKVNKEVIEKELFPFLDHAAEEALVFDLQDVFQRLMLDSSSILTTGQNHRSLRVGLPYDETLEAINIANYQIFVRHILPAKIWKFQKWLGIMGEKKIKKAWRILDDISVEYMNRRKKEISSTISSQEDMDVVKFSEEDHVVLKSVDASDNLLRDTVKGILLAGTDTTATVLSWFFWLILKNPRVEQKIREEIELYLKQKNGEHGLYTNPEELNELMYLHAAIYETMRLYPAAPFTSRKSIQADVFPTGHQVNPNTTIVMAYYAVGRMKSIWGEDCLEFKPERWLSDKGKLIPVQTNKFLAFGTGPRICPGKELGLNRVKAVAAAIIPKYSFKIMRNKPVMPAACATLYLKDGLIVRVNKII | |
| CYP96D1 | MGPLWTFILLYPEIFLAIICFFWFSLFRPIRQRQKSNLPVNWPVFGMLPFLVQNLHYIHDKVADVLREAGCTFMVSGPWFLNMNFLITCDPATVNHCFNANFKNYPKGSEFAEMFDILGDGLLVADSESWEYQRRMAMYIFAARTFRSFAMSTITRKTGSVLLPYLDHMAKFGSEVELEGVFMRFSLDVTYSTVFAADLDCLSVSSPIPVFGQATKEAEEAVLFRHVIPPSVWKLLRLLNVGTEKKLTNAKVVIDQFIYEEIAKRKAQASDGLQGDILSMYMKWSIHESAHKQKDERFLRDTAVGFIFAGKDLIAVTLTWFFYMMCKHPHVEARILQELKGLQSSTWPGDLHVFEWDTLRSAIYLQAALLETLRLFPATPFEEKEALVDDVLPNGTKVSRNTRIIFSLYAMGRIEGIWGKDCMEFKPERWVSKSGRLRHEPSYKFLSFNTGPRSCLGKELSLSNMKIIVASIIHNFKVELVEGHEVMPQSSVILHTQNGMMVRLKRRDAA | |
| CYP96E1 | MELLPWLLGFVVKYPEIMASAACFLLLFCRFRRRSKRIPTNWPVVGALPAIVANAGRVHDWVTEFLRAAAMSHVVEGPWGSPGDVLITADPANVAHMFTANFGNYPKGEEFAAMFDVLGGGIFNADGESWSFQRRKAHALLSDARFRAAVAASTSRKLGGGLVPLLDGVAASGAAVDLQDVFMRLTFDLTAMFVFGVDPGCLAADFPTVPFAAAMDDAEEVLFYRHVAPVPWLRLQSYLKIGHYKKMAKAREVLDASIAELIALRRERKAADANATGDADLLTAYLACQDEIGMDGAAFDAFLRDTTLNLMVAGRDTTSSALTWFFWLLSNHPGVEARILAELRAHPPSPTGAELKRLVYLHAALSESLRLYPPVPFEHKAAARPDTLPSGAAVGPTRRVIVSLYSMGRMEAVWGKGCEEFRPERWLTPAGRFRHERSCKFAAFNVGPRTCLGRDLAFAQMKAVVAAVVPRFRVAAAAAPPRPKLSIILHMRDGLKVKVHRRQED | |
| CYP96F1 | MAILEYFHIIIALVCILLFCHWCRNTVTPVTNWPVVGMLPGLLFKAQNIHEYATQLLKQSGGTFEFKGPWFANMNILLTADPLNVRHISTTNFVNYPKGPEYKKIFEPYGDGVLNSDFESWKSFRKLIHSMIKDNKFQVSLERSLREKIVEGLIPVLEHASRQEIELDMQDVFQRFTFDNICLLVLGFDPQSLSVDLPEIAYKTAFDDVEEAVFYRHIVPESIWKLQKWLNVGEEKKLSQAMDTIDNFLEQCISSKKEEIRQRKAQNMVQVEDNDQDDYDLITACIKEGEEAEQMDASKRTDKYLRDIGFNFIAAGKDTVNAALTWFFWLVATHHEVEEKIVEEIRANMKSKGDHTINGMFFNLEELNKLVYLHGAICETLRLYPAVPFNYRVSVEADTLPSGHLVKENTKVMFSLYSMGSMEEIWGDDCLAFKPERWISERGGIIHVPSYKYIAFNTGPRSCLGKEITFIQMKTIATAILLNFHLQVVEGHPVSPGLSVMLHMKHGLKLRVTKRCV | |
| CYP96G1 | MAILTLIILFVATIFSIFFLPFIKKNSSPWILVRSLVNFYRMHDNQAEMLEQNNGTILVKRSWFGGKDILLTSDPANVRHIMSTNFSNYPKGPEWKKQFEFFGDSVFTLDFEEWKHHRKVIRSYISHRSFQQFAGKIVRDCIEIELSSVLDRVSNQQIVVDFEGLLRRYIYYFACCISTGYKPKFIDLAFSEDKFLKATDDACDAITVRYLVPESIWKLQRWFGLGKEKRLSEARKTIDKIIDDYISMKQEEMSKGEIRNDEEDFSALKSYTTGNEIFEQADHKVIKDGTMSLIFAIEDTTSSSLSRFFWIFTKNPKVETKIRQELEKIRPVTEARKSSSFFSEEEVSKMVYLQAALLETLRLFPPASMVSKTAVEADTLPSGHHISQNTMVVISAYAMGRMRTIWGQDCLEFKPERWIMEDGRIRHEPPHKFSAFGSGPRICPGKDLGLTLLKTFAATIIYNYHIQVVEDRVGAPKNNFMLHLDHGLMVRLNKRWT | |
| CYP96H1 | MVAESILAIAFFLVLICLFRSKNGLPWNWPLLGMSPTLLLNSHRLYELADEILEISGGTYLFKGLWFSNMDMWFTSDPENVHYITTTNYWNYPKGPESMQVFDTLGNSLFNLDFEEWTYYRGLLHGFFSHQKFHQFVPKVLVDNVNKGLVPFLEDVAKQALVVDLQDMFKRHIYDAACAIATGYNPKTLSIGFEENAFVRAMDDACVAMLTRHILPGRCWKLLRWLQIGSEKRLSVAKGTLRQIVTNYMATKREELSAGAKTKEDEETFDVLRSFLTINDVNDKEHPDEIVRDSTIGIIFAAYDTSSATLSWFFWLLSKNPHVETKIREELDSNFSVKEGQNWQLNSRKELSKLVYLHATLCETLRLYPPVPLQRRTPVRPDIFPSGHHVDPKAIVVLSGYAIGRMARVWGQDCHEFKPERWINEKGDLKYERSAKFFTFNAGPRICPGKEMAFSIMKAAATTILYNYHVQVVETRPVTPKASIILQMKHGLRARICSRWT | |
| CYP96J1 | MQFANIHFSKTTMSIIQCIGGFVTILIFLYIYYSRRNRDELLLINWPIIGILPSFLCHLSDYHDYSTIVLKRYGGTCRFQGPWFTNTSFIALADQMNVNYITKKNCGNYRKGSKYHDIFEVLGGSIFNSDSDDVWKQEKTMFHLVLGRKSFKNMFEQSIQKKVENYLIPFLNDVSEAGAHVDLQDAFNRFTFDSSCMILFGFDPNCLPNKFNQLRKIPYKESLPVMEEVILYRHFIPSSLWKLQKWLNVGQEKKFKVAQEYLDRFLYESITFSHGEEQSKCSDEEMDQCFLGMVKALKKEGHGKGEISEKYLRDTAFTMIFAGNGTISSALSWFFWLLSTYPIVEEKIIQEIKDNWLTQEGNRITLRHVDLDKLVYLHGAICETLRLYPPIPFEHICSIKSDILPSGYHVSPNTTLIYSLYSMGRMEQIWGEDCMEFKPERWISERGDII | |
| CYP96K1 | MMAVLFVYLGFMVFGIIVLRFLYRFIDNNGLPRNWPFVGMIPTLLLNIHRPHDKVAQVLRRSNGTFFYRGLWFTNTSFLATSDPENVRYILSSNSSVYLKGPEWLKQFDIFGEALFNSDGEAWKCHRRVFHAFLNHPQYRQSLSKVLHQRIEEALVKVLEYVSGREMVVNLQDLLAGHAFDIGCITGVGFDPGLLSIEFPENRFQKAMSDTLEAAFYRYVVPDSLWKLQSWPQIGKGKKRSDAWKALDDLLTQFISTQRHKSTKSVASSGSNEEHDFNFLNCYLTGHEITGPTPKESLIRDNLIHFLFASDGTYSLTLTWFFYLISKAPVVENKIREEIKRHLSMKQVEGSLQIPSNYDELSKLTYLHAALCETLRLYPPIPFDFRTCTKQEYLPSGHRVDQNTRIIIGIHAMGRMESLWGEDCYAFKPERWIGEDGKIKRESPTKFSAFLAGPRICPGKEVSFLLMKATATAIIHNYNVHVVEGQNIGPKNSVVYQMKKGLMVRIKKRWS | |
| CYP97A3 | MAMAFPLSYTPTITVKPVTYSRRSNFVVFSSSSNGRDPLEENSVPNGVKSLEKLQEEKRRAELSARIASGAFTVRKSSFPSTVKNGLSKIGIPSNVLDFMFDWTGSDQDYPKVPEAKGSIQAVRNEAFFIPLYELFLTYGGIFRLTFGPKSFLIVSDPSIAKHILKDNAKAYSKGILAEILDFVMGKGLIPADGEIWRRRRRAIVPALHQKYVAAMISLFGEASDRLCQKLDAAALKGEEVEMESLFSRLTLDIIGKAVFNYDFDSLTNDTGVIEAVYTVLREAEDRSVSPIPVWDIPIWKDISPRQRKVATSLKLINDTLDDLIATCKRMVEEEELQFHEEYMNERDPSILHFLLASGDDVSSKQLRDDLMTMLIAGHETSAAVLTWTFYLLTTEPSVVAKLQEEVDSVIGDRFPTIQDMKKLKYTTRVMNESLRLYPQPPVLIRRSIDNDILGEYPIKRGEDIFISVWNLHRSPLHWDDAEKFNPERWPLDGPNPNETNQNFSYLPFGGGPRKCIGDMFASFENVVAIAMLIRRFNFQIAPGAPPVKMTTGATIHTTEGLKLTVTKRTKPLDIHPYRYFQWILTG | |
| CYP97B3 | MAFPAAATYPTHFQGGALHLGRTDHCLFGFYPQTISSVNSRRASVSIKCQSTEPKTNGNILDNASNLLTNFLSGGSLGSMPTAEGSVSDLFGKPLFLSLYDWFLEHGGIYKLAFGPKAFVVISDPIIARHVLRENAFSYDKGVLAEILEPIMGKGLIPADLDTWKLRRRAITPAFHKLYLEAMVKVFSDCSEKMILKSEKLIREKETSSGEDTIELDLEAEFSSLALDIIGLSVFNYDFGSVTKESPVIKAVYGTLFEAEHRSTFYFPYWNFPPARWIVPRQRKFQSDLKIINDCLDGLIQNAKETRQETDVEKLQERDYTNLKDASLLRFLVDMRGVDIDDRQLRDDLMTMLIAGHETTAAVLTWAVFLLSQNPEKIRKAQAEIDAVLGQGPPTYESMKKLEYIRLIVVEVLRLFPQPPLLIRRTLKPETLPGGHKGEKEGHKVPKGTDIFISVYNLHRSPYFWDNPHDFEPERFLRTKESNGIEGWAGFDPSRSPGALYPNEIIADFAFLPFGGGPRKCIGDQFALMESTVALAMLFQKFDVELRGTPESVELVSGATIHAKNGMWCKLKRRSK | |
| CYP97C1 | MESSLFSPSSSSYSSLFTAKPTRLLSPKPKFTFSIRSSIEKPKPKLETNSSKSQSWVSPDWLTTLTRTLSSGKNDESGIPIANAKLDDVADLLGGALFLPLYKWMNEYGPIYRLAAGPRNFVIVSDPAIAKHVLRNYPKYAKGLVAEVSEFLFGSGFAIAEGPLWTARRRAVVPSLHRRYLSVIVERVFCKCAERLVENVAALCKRRNSCKYERNFSSQMTLDVIGLSLFNYNFDSLTTDSPVIEAVYTALKEAELRSTDLLPYWKIDALCKIVPRQVKAEKAVTLIRETVEDLIAKCKEIVEREGERINDEEYVNDADPSILRFLLASREEVSSVQLRDDLLSMLVAGHETTGSVLTWTLYLLSNSSALRKAQEEVDRVLEGRNPAFEDIKELKYITRCINESMRLYPHPPVLIRRAQVPDILPGNYKVNTGQDIMISVYNIHRSSEVWEKAEEFLPERFDIDGAIPNETNTDFKFIPFSGGPRKCVGDQFALMEAIVALAVFLQRLNVELVPDQTISMTTGATIHTTNVCQWIVYEGEPKVKTRIYVFMIIDWCEWTCFIVLLRNNHKKMNYGK | |
| CYP97E5 | MVARARVHASRGVDARRVRARGRARVDVIARAVKEPSSAPEEALPDENFKPEQLKFQDIVSLWVTQILQTYGGKESKDNAPVCEGVIDDLVGGPIFLALYPYFRRYGGVFKLAFGPKVFMVLSDPVIVREVLKEKPFSFDKGVLAEILEPIMGQGLIPAPYAVWKNRRRQLVPGFHKAWLDHMVGLFGHCSNELVRNLDKSAEDGEVVDMEERFCSVSLDIIGLAVFNYDFGSVTKESPIISAVYNCLQEAAHRSTFYFPYWNIPFATDIVPRQREFKQNMKIINETLNGLIQKAQKFEGTEDLEELQNRDYSKVKDPSLLRFLVDIRGADVTDSQLRDDLMTMLIAGHETTAAVLTWGLFCLMQNPELMKRIQADIDEVMGDDDRTPTYDDIQKLESVRLCIAEALRLYPEPPILIRRCLEDVTLPKGAGDAEVTLIKGMDIFISVWNLHRSPECWENPEEFDPFRFKRPFANPGVKDWAGYNPELFTGLYPNEVASDFAFIPFGAGARKCIGDQFAMLEATIAMAMVLRRYDFELTTDPKDIGMTMGATIHTEKGLPCRVRRRQPVTTATAAAV | |
| CYP98A3 | MSWFLIAVATIAAVVSYKLIQRLRYKFPPGPSPKPIVGNLYDIKPVRFRCYYEWAQSYGPIISVWIGSILNVVVSSAELAKEVLKEHDQKLADRHRNRSTEAFSRNGQDLIWADYGPHYVKVRKVCTLELFTPKRLESLRPIREDEVTAMVESVFRDCNLPENRAKGLQLRKYLGAVAFNNITRLAFGKRFMNAEGVVDEQGLEFKAIVSNGLKLGASLSIAEHIPWLRWMFPADEKAFAEHGARRDRLTRAIMEEHTLARQKSSGAKQHFVDALLTLKDQYDLSEDTIIGLLWDMITAGMDTTAITAEWAMAEMIKNPRVQQKVQEEFDRVVGLDRILTEADFSRLPYLQCVVKESFRLHPPTPLMLPHRSNADVKIGGYDIPKGSNVHVNVWAVARDPAVWKNPFEFRPERFLEEDVDMKGHDFRLLPFGAGRRVCPGAQLGINLVTSMMSHLLHHFVWTPPQGTKPEEIDMSENPGLVTYMRTPVQAVATPRLPSDLYKRVPYDM | |
| CYP98A3 | MSWFLIAVATIAAVVSYKLIQRLRYKFPPGPSPKPIVGNLYDIKPVRFRCYYEWAQSYGPIISVWIGSILNVVVSSAELAKEVLKEHDQKLADRHRNRSTEAFSRNGQDLIWADYGPHYVKVRKVCTLELFTPKRLESLRPIREDEVTAMVESVFRDCNLPENRAKGLQLRKYLGAVAFNNITRLAFGKRFMNAEGVVDEQGLEFKAIVSNGLKLGASLSIAEHIPWLRWMFPADEKAFAEHGARRDRLTRAIMEEHTLARQKSSGAKQHFVDALLTLKDQYDLSEDTIIGLLWDMITAGMDTTAITAEWAMAEMIKNPRVQQKVQEEFDRVVGLDRILTEADFSRLPYLQCVVKESFRLHPPTPLMLPHRSNADVKIGGYDIPKGSNVHVNVWAVARDPAVWKNPFEFRPERFLEEDVDMKGHDFRLLPFGAGRRVCPGAQLGINLVTSMMSHLLHHFVWTPPQGTKPEEIDMSENPGLVTYMRTPVQAVATPRLPSDLYKRVPYDM | |
| CYP99A1 | RLISAVILAVCSLISRRKPSPGSKKKRPPGPWRLPLIGNLLHLATSQPHVALRDLAMKHGPVMYLRLGQVDAVVISSPAAAQEVLRDKDTTFASRPSLLVADIILYGSMDMSFAPYGGNWRMLRKLCMSELLNTHKVRQLAAVRDSETLSLVRKVVYAAGAGGGGRGQRGEAPVVNLGRLVLSCSMAITGRATLGKLCGDEIMSVVDVAVLYGSGFCAGDLFPSLWFVDVVTGLTRRLWTARRRLDAIFDRILAECEARQRQEEKMTGDDGFLGVLLRIRDDDGEPETGGISTTSIKAILFDMLAGGTETTSSAAEWIMSELMRKPEAMAKAQAEVRGALDGKSPEDHEGQMDKLSYTRMVVKEGLRLHPVLPLLLPRSCQETCDVGGFEVTKGTKVIVNAWALARSPERWHDPEEFRPERFADDDGSSAAVAVDYRGSQFEYIPFGSGRRMCPGNTFGLAALELMVARLLYYFDWSLPDGMRPEELDMDTVVGSTMRRRNHLHLVPSPYKETELTVGI | |
