# Supplementary material for: Functional characterization of the cytochrome P450 monooxygenase CYP71AU87 indicates a role in marrubiin biosynthesis in the medicinal plant Marrubium vulgare
Source: BMC Plant Biol. 2019 Mar 25;19:114. doi: 10.1186/s12870-019-1702-5 (PMC6434833; doi:10.1186/s12870-019-1702-5)

Supplemental Fig. S1: NMR analysis of labda-13(16),14-dien-9-ol (compound 2) formed by the coupled reaction of MvCPS1 and MvELS.

A) 1D NMR analysis

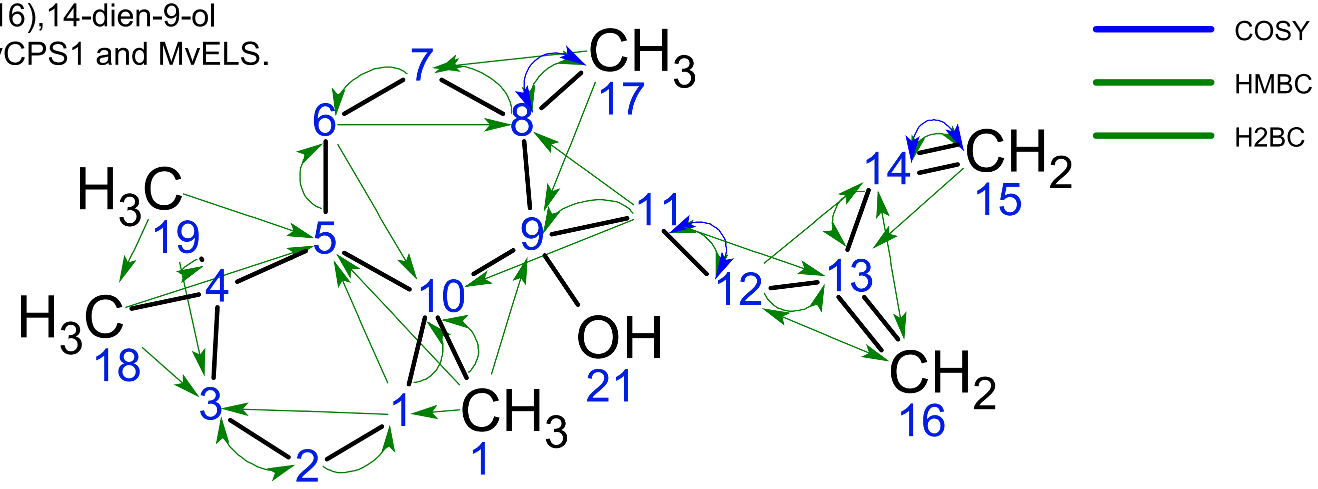

#	Atom#	C Label	C Shift	XHn	H Label	H Shift	C Calc Shift (Neural Net)	H Calc Shift (Neural Net)	H Multiplicity	COSY	H HMBC	C HMBC
1	1	C 1	16.172	CH3	H 4	0.934	22.651	0.950	m			1, 10, 5, 9
2	17	C 2	16.427	CH3	H 3	0.903	17.366	0.973	m	8	8	7, 8, 9
3	2	C 3	18.673	CH2	H 11	1.477	19.050	1.513	m		3	
4	2	C 3	18.673	CH2	H 14	1.562	19.050	1.513	br dd (8.86, 5.79)		3	1, 3
5	6	C 4	21.641	CH2	H 13	1.538	21.816	1.394	m		7, 5	8, 10
6	6	C 4	21.641	CH2	H 7	1.301	21.816	1.394	m		7, 5	
7	18	C 5	22.013	CH3	H 1	0.835	28.816	0.894	s		19	3, 5
8	12	C 6	27.936	CH2	H 18	2.272	29.114	2.462	m	11	11, 11, 16	11, 16, 14, 13
9	7	C 9	31.338	CH2	H 6	1.299	31.999	1.554	m		17, 8	6
10	7	C 9	31.338	CH2	H 12	1.487	31.999	1.554	m		17, 8	
11	1	C 1	31.914	CH2	H 10	1.470	35.637	1.347	m		1, 2	3, 10, 5
12	4	C 9	33.329	C			34.148				19	
13	11	C 10	33.489	CH2	H 17	1.818	30.145	1.534	m	12	12	12, 8, 10, 9, 13
14	11	C 10	33.489	CH2	H 15	1.574	30.145	1.534	ddd (14.39, 11.15, 6.13)		12	12
15	19	C 11	33.752	CH3	H 2	0.874	28.816	0.894	s			18, 4, 3, 5
16	8	C 12	36.676	CH	H 16	1.779	35.521	1.832	m	17	17, 6, 11	17, 7
17	3	C 13	41.751	CH2	H 5	1.155	42.254	1.088	td (13.20, 13.20, 3.58)	3	18, 19, 1, 2	2
18	3	C 13	41.751	CH2	H 8	1.350	42.254	1.088	m	3	18, 19, 1, 2	
19	10	C 14	43.275	C			42.861				1, 1, 6, 11	
20	5	C 15	46.315	CH	H 9	1.442	47.065	1.474	m		18, 19, 1, 1	6
21	9	M01	77.224	C			79.856				17, 1, 11	
22	15	C 17	113.353	CH2	H 20	5.071	113.139	5.170	d (10.90)	14	14	13
23	15	C 17	113.353	CH2	H 21	5.255	113.139	5.270	d (17.37)	14	14	14, 13
24	16	C 18	115.431	CH2	H 19	5.002	114.625	4.870, 4.897	br d (4.77)		12, 14	12, 14
25	14	C 19	138.880	CH	H 22	6.361	138.262	6.357	dd (17.71, 10.90)	15, 15	12, 16, 15	15, 16, 13
26	13	C 20	147.543	C			146.588				11, 12, 15, 15, 14	

B) ^{1}H HNMR analysis

Acquisition Time (sec)	2.9360	***Comment***	1H NMR-2min		
D	0.0002	***D1***	2	***DE***	18
DS	2	***Date***	19 Apr 2017 15:27:33		
Date Stamp	19 Apr 2017 15:27:33				
File Name	\\169.237.229.248\share$\ACDLabs\Zerbe\Prema\081717-mz191-Best\1\fid				
Frequency (MHz)	800.1540	***GB***	0	***INSTRUM***	<spect>
LB	0.1	***NS***	16	***Nucleus***	1H
Number of Transients	16	***Origin***	spect	***Original Points Count***	32768
Owner	pkarunan	***PC***	1		
PROBHD	<5 mm CPTCI 1H-13C/15N/D Z-GRD Z107231/0001 >				
PULPROG	<zg30>	***Points Count***	32768	***Pulse Sequence***	zg30
Receiver Gain	36.00	***SF***	800.15	***SFO1***	800.15400075
SI	65536	***SSB***	0	***SW(cyclical) (Hz)***	11160.71
SWH	11160.7142857143			***Solvent***	CHLOROFORM-d
Spectrum Offset (Hz)	3983.4666	***Spectrum Type***	standard	***Sweep Width (Hz)***	11160.37
TD	65536	***TD0***	1	***TE***	303
Temperature (degree C)	30.000	***UNC1***	<1H>	***WDW***	1

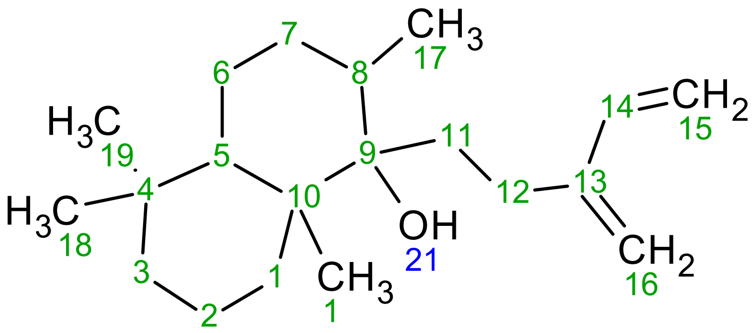

^{1}H NMR (800 MHz, CHLOROFORM-*d*) δ ppm 0.83 (s, 7 H) 0.87 (s, 9 H) 0.89 - 0.92 (m, 8 H) 0.92 - 0.94 (m, 6 H) 1.16 (td, *J*=13.20, 3.58 Hz, 4 H) 1.27 - 1.32 (m, 5 H) 1.28 - 1.32 (m, 6 H) 1.33 - 1.37 (m, 8 H) 1.42 - 1.46 (m, 10 H) 1.46 - 1.50 (m, 6 H) 1.45 - 1.49 (m, 6 H) 1.47 - 1.51 (m, 6 H) 1.52 - 1.56 (m, 4 H) 1.54 (br dd, *J*=8.86, 5.79 Hz, 7 H) 1.57 (ddd, *J*=14.39, 11.15, 6.13 Hz, 3 H) 1.75 - 1.81 (m, 3 H) 1.80 - 1.84 (m, 2 H) 2.25 - 2.30 (m, 2 H) 5.00 (br d, *J*=4.77 Hz, 2 H) 5.07 (d, *J*=10.90 Hz, 1 H) 5.26 (d, *J*=17.37 Hz, 1 H) 6.36 (dd, *J*=17.71, 10.90 Hz, 1 H)

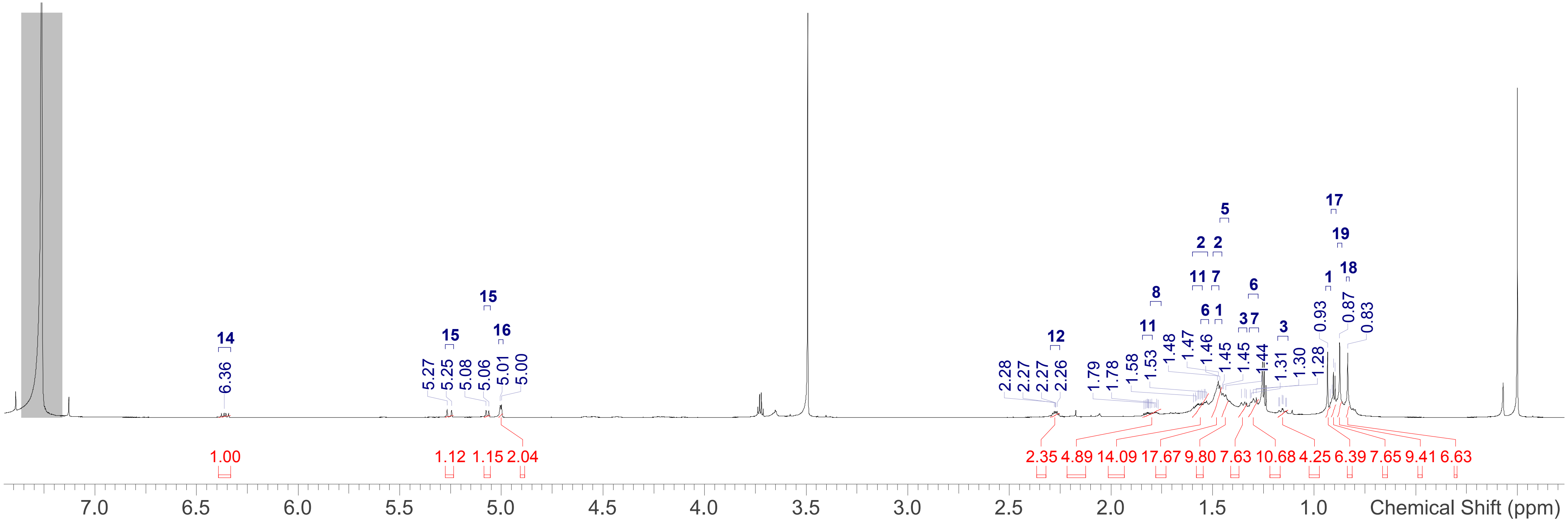

C) ^{13}C NMR analysis

Acquisition Time (sec)	0.6816	***Comment***	C-NMR-8000 scans
D	1.5	***D1***	1.5
DE	18.85148	***DS***	4
Date	19 Apr 2017 22:36:28		
Date Stamp	19 Apr 2017 22:36:28		
File Name	\\169.237.229.248\share$\ACDLabs\Zerbe\Prema\081717-mz191-Best\5\fid		
Frequency (MHz)	201.2200	***GB***	0
INSTRUM	<spect>	***LB***	0.5
NS	8000	***Nucleus***	13C
Number of Transients	8000	***Origin***	spect
Original Points Count	32768	***Owner***	pkarunan
PC	1.4		
PROBHD	<5 mm CPTCI 1H-13C/15N/D Z-GRD Z107231/0001 >		
PULPROG	<zgpg30>	***Points Count***	32768
Pulse Sequence	zgpg30	***Receiver Gain***	2050.00
SF	201.19787753		
SFO1	201.220009296528		
SI	65536	***SSB***	0
SW(cyclical) (Hz)	48076.92	***SWH***	48076.9230769231
Solvent	CHLOROFORM-d		
Spectrum Offset (Hz)	22131.7656	***Spectrum Type***	standard
Sweep Width (Hz)	48075.45	***TD***	65536
TD0	1	***TE***	303
Temperature (degree C)	30.000	***UNC1***	<13C>
WDW	1		

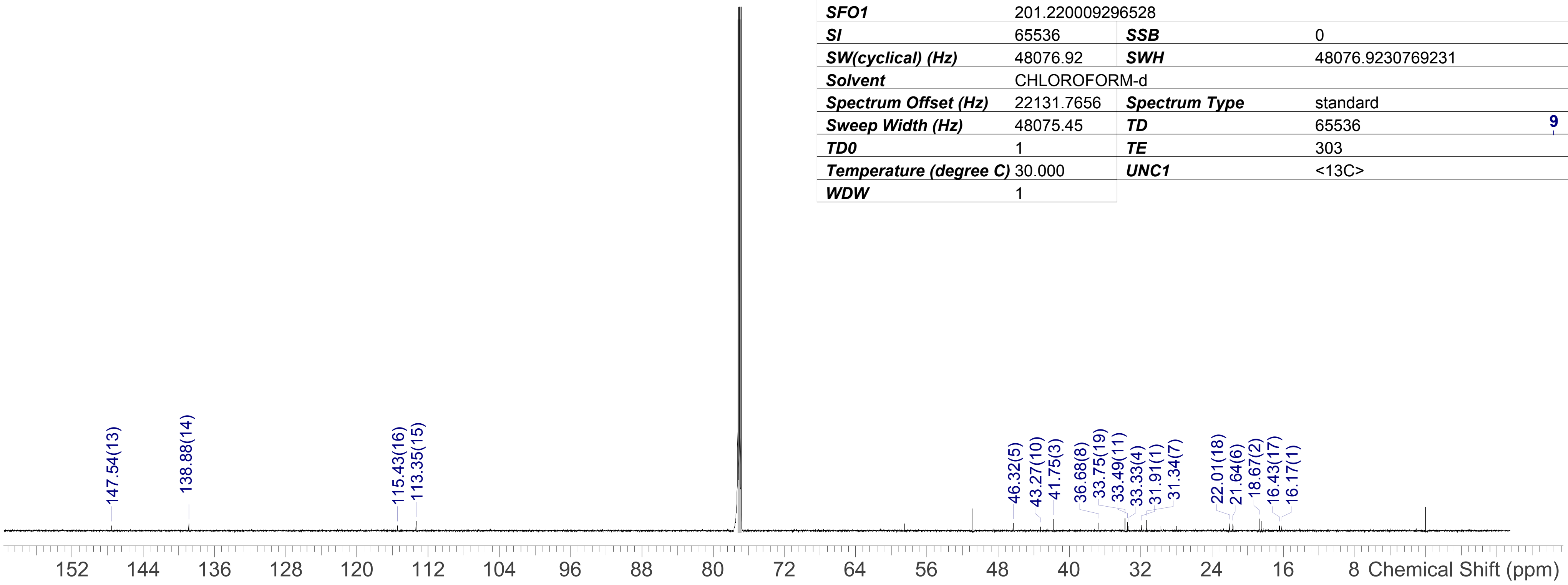

D) HSQC NMR analysis

Acquisition Time (sec)	(0.1278, 0.0119)	***Comment***	HSQC-16 scans
Constant (Hz)	145.0	***Date***	20 Apr 2017 04:22:42
File Name	\\169.237.229.248\share$\ACDLabs\Zerbe\Prema\081717-mz191-Best\7\ser		
Frequency (MHz)	(800.1500, 201.1979)		
Nucleus	(1H, 13C)	***Number of Transients***	16
Origin	spect	***Original Points Count***	(1024, 400)
Owner	pkarunan	***Points Count***	(1024, 1024)
Pulse Sequence	hsqcedetgpsp.3	***Solvent***	CHLOROFORM-d
Spectrum Type	HSQC	***Sweep Width (Hz)***	(8005.00, 33524.28)
Temperature (degree C)	30.000	***Title***	HSQC-16 scans

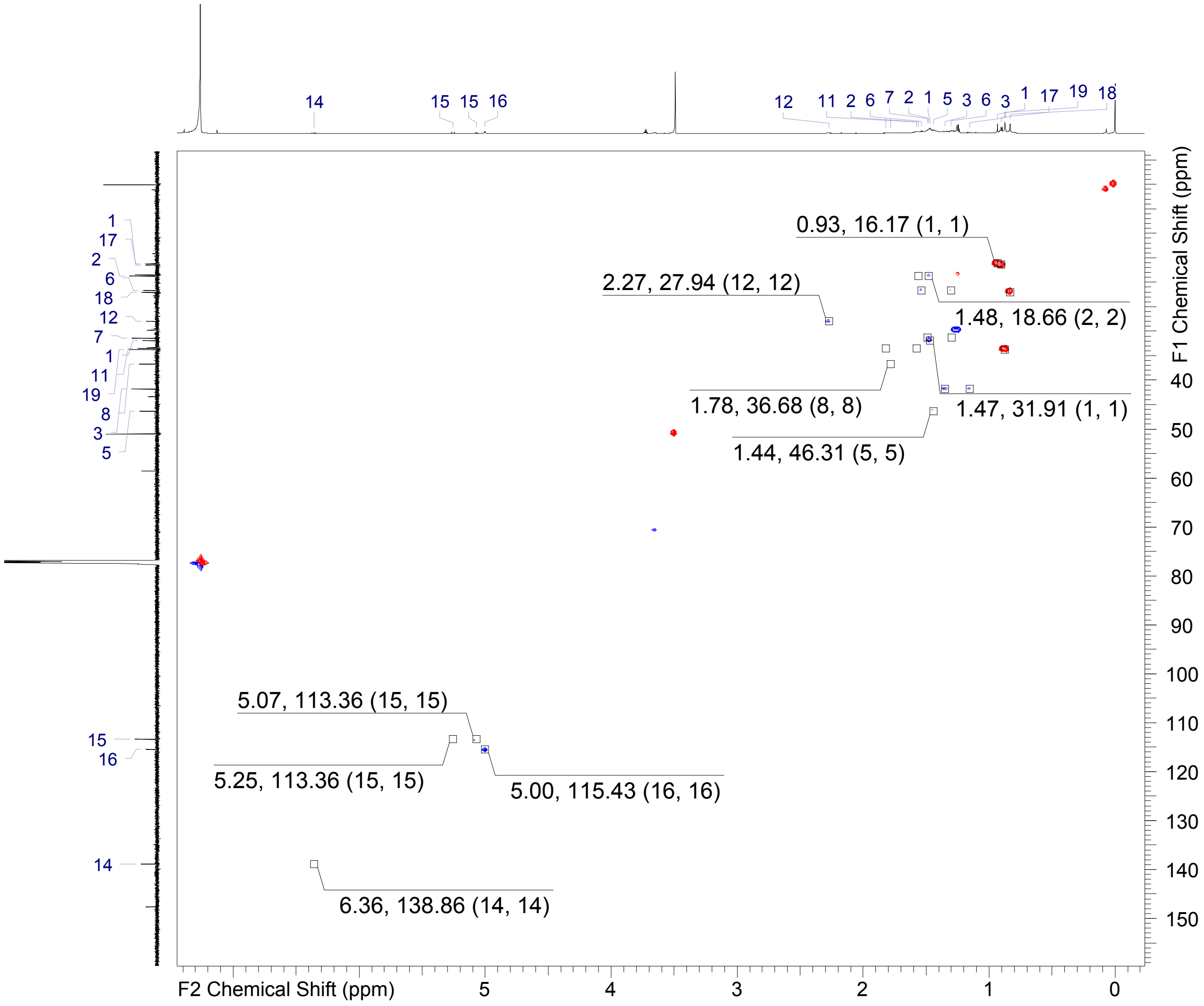

No.	F2 Atom	F1 Atom	F2 (ppm)	F1 (ppm)
1	1	1	0.93	16.17
2	1	1	1.47	31.91
3	2	2	1.48	18.66
4	2	2	1.56	18.66
5	3	3	1.15	41.74
6	3	3	1.35	41.74
7	5	5	1.44	46.31
8	6	6	1.30	21.63
9	6	6	1.54	21.63
10	7	7	1.30	31.33
11	7	7	1.49	31.33
12	8	8	1.78	36.68
13	11	11	1.57	33.49
14	11	11	1.82	33.49
15	12	12	2.27	27.94
16	14	14	6.36	138.86
17	15	15	5.07	113.36
18	15	15	5.25	113.36
19	16	16	5.00	115.43
20	17	17	0.90	16.44
21	18	18	0.83	22.01
22	19	19	0.87	33.74

E) COSY NMR analysis

Acquisition Time (sec)	(0.1278, 0.0319)	Comment	COSY-16 scans
Date	20 Apr 2017 00:32:24		
File Name	\\169.237.229.248\share$\ACDLabs\Zerbe\Prema\081717-mz191-Best\6\ser		
Frequency (MHz)	(800.1500, 800.1500)		
Nucleus	(1H, 1H)	Number of Transients	16
Origin	spect	Original Points Count	(1024, 256)
Owner	pkarunan	Points Count	(1024, 2048)
Pulse Sequence	cosygpmfppqf	Solvent	CHLOROFORM-d
Spectrum Type	COSY	Sweep Width (Hz)	(8005.00, 8008.91)
Temperature (degree C)	30.000	Title	

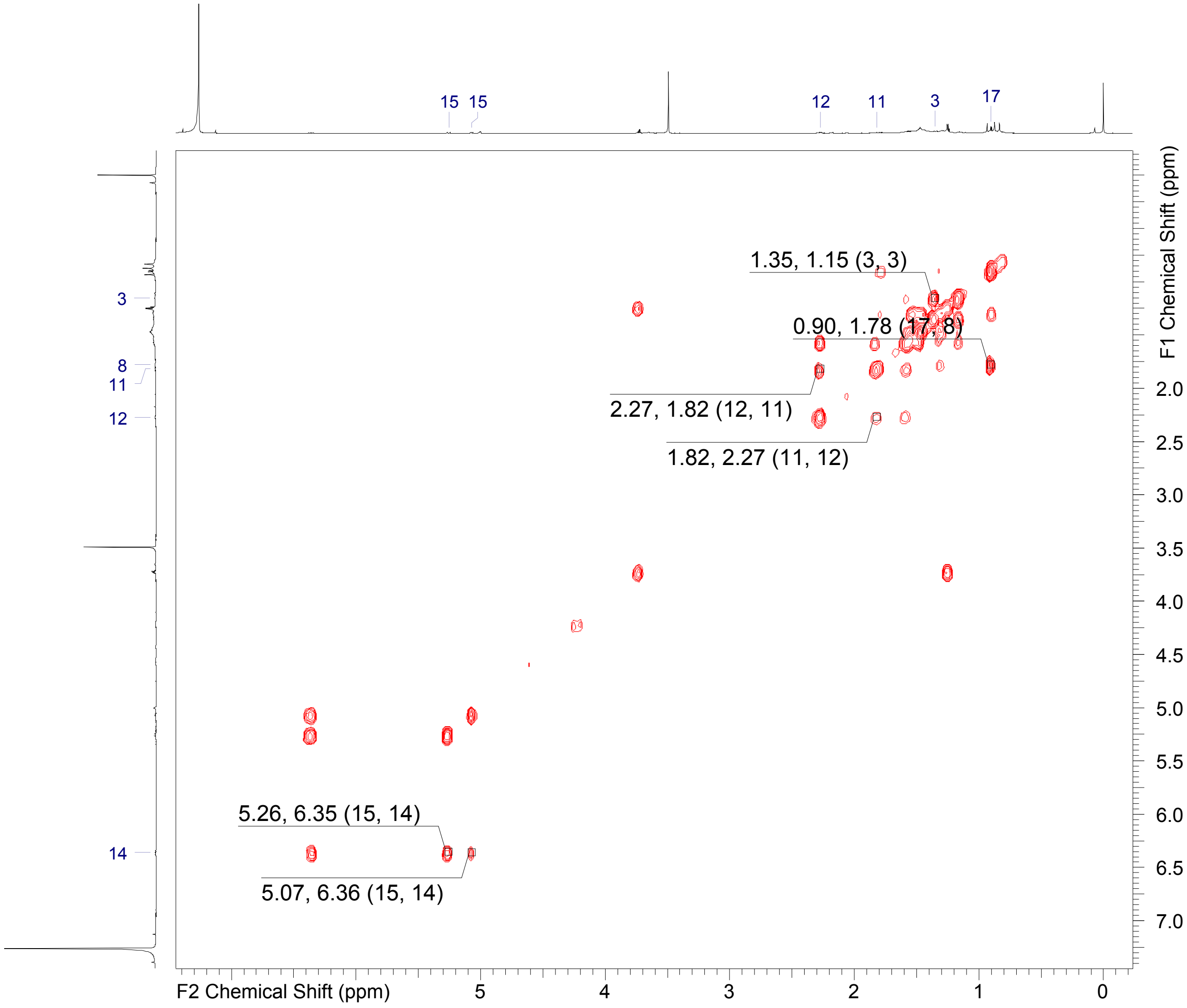

No.	F2 Atom	F1 Atom	F2 (ppm)	F1 (ppm)
1	3	3	1.35	1.15
2	17	8	0.90	1.78
3	12	11	2.27	1.82
4	11	12	1.82	2.27
5	15	14	5.26	6.35
6	15	14	5.07	6.36

F) HMBC NMR analysis

Acquisition Time (sec)	(0.1278, 0.0112)	***Comment***	HMBC-32 scans
Constant (Hz)	8.0	***Date***	20 Apr 2017 10:28:24
File Name	\\169.237.229.248\share$\ACDLabs\Zerbe\Prema\081717-mz191-Best\8\ser		
Frequency (MHz)	(800.1500, 201.1979)		
Nucleus	(1H, 13C)	***Number of Transients***	32
Origin	spect	***Original Points Count***	(1024, 400)
Owner	pkarunan	***Points Count***	(1024, 2048)
Pulse Sequence	hmbcetgpl3nd	***Solvent***	CHLOROFORM-d
Spectrum Type	HMBC	***Sweep Width (Hz)***	(8005.00, 35696.85)
Temperature (degree C)	30.000	***Title***	HMBC-32 scans

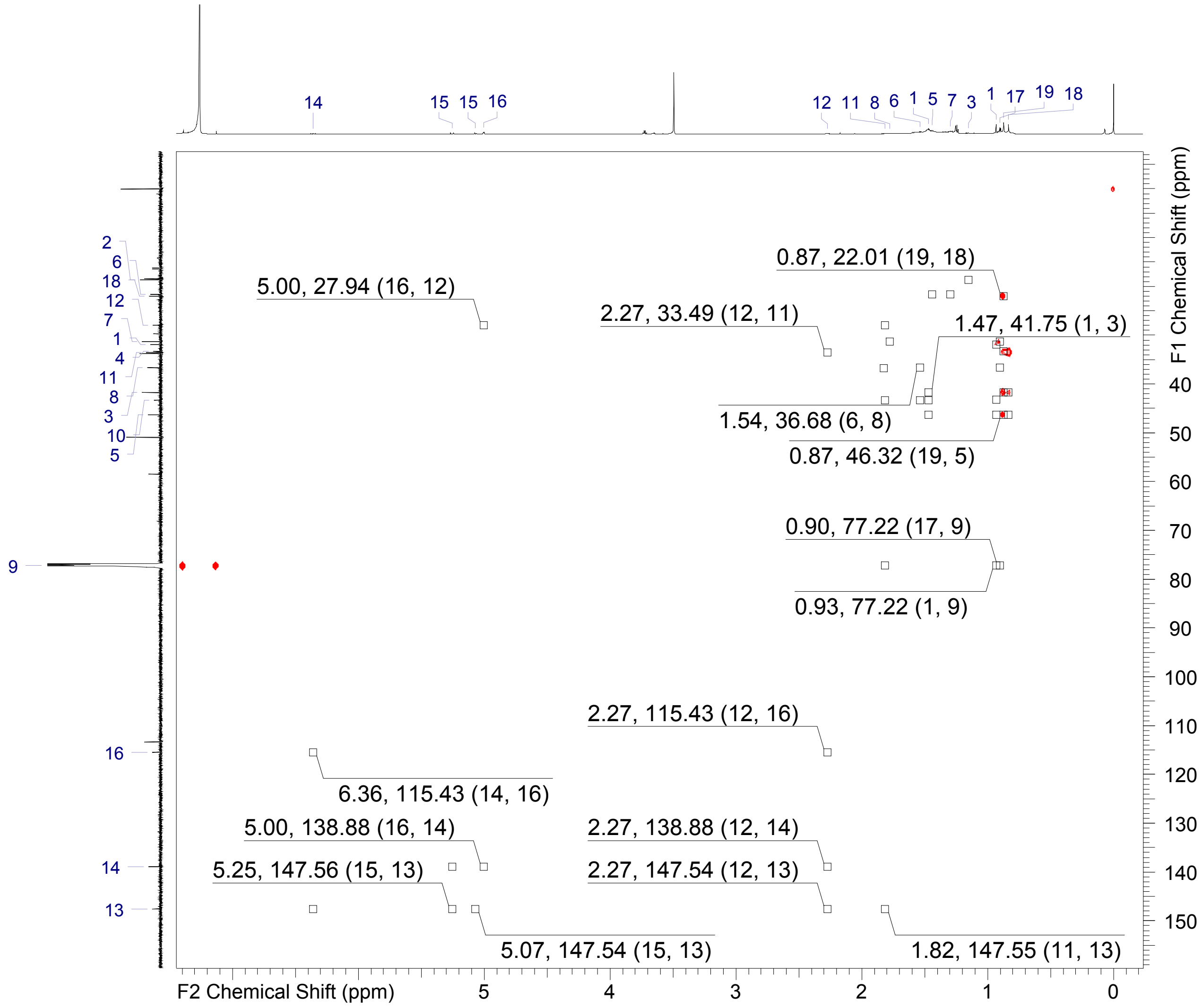

No.	F2 Atom	F1 Atom	F2 (ppm)	F1 (ppm)
1	1	1	0.93	31.91
2	3	2	1.15	18.67
3	1	3	1.47	41.75
4	18	3	0.83	41.75
5	19	3	0.87	41.75
6	19	4	0.87	33.32
7	1	5	0.93	46.32
8	1	5	1.47	46.32
9	18	5	0.83	46.32
10	19	5	0.87	46.32
11	5	6	1.44	21.61
12	7	6	1.30	21.64
13	8	7	1.78	31.34
14	17	7	0.90	31.34
15	6	8	1.54	36.68
16	11	8	1.82	36.74
17	17	8	0.90	36.68
18	1	9	0.93	77.22
19	11	9	1.82	77.22
20	17	9	0.90	77.22
21	1	10	0.93	43.27
22	1	10	1.47	43.27
23	6	10	1.54	43.27
24	11	10	1.82	43.27
25	12	11	2.27	33.49
26	11	12	1.82	27.94
27	16	12	5.00	27.94
28	11	13	1.82	147.55
29	12	13	2.27	147.54
30	14	13	6.36	147.54
31	15	13	5.07	147.54
32	15	13	5.25	147.56
33	12	14	2.27	138.88
34	15	14	5.25	138.88
35	16	14	5.00	138.88
36	12	16	2.27	115.43
37	14	16	6.36	115.43
38	19	18	0.87	22.01

G) H2BC NMR analysis

Acquisition Time (sec)	(0.2556, 0.0080)	***Comment***	H2BC-32 scans
Date	01 Jul 2017 03:23:40		
File Name	\\169.237.229.248\share$\ACDLabs\Zerbe\Prema\081717-mz191-Best\11\ser		
Frequency (MHz)	(800.1500, 201.1979)		
Nucleus	(1H, 13C)	***Number of Transients***	32
Origin	spect	***Original Points Count***	(2048, 256)
Owner	pkarunan	***Points Count***	(2048, 1024)
Pulse Sequence	h2bcetgpl3	***Solvent***	CHLOROFORM-d
Spectrum Type	H2BC	***Sweep Width (Hz)***	(8008.91, 31816.03)
Temperature (degree C)	30.000	***Title***	H2BC-32 scans

No.	F2 Atom	F1 Atom	F2 (ppm)	F1 (ppm)
1	2	1	1.56	31.91
2	2	3	1.56	41.75
3	5	6	1.44	21.64
4	8	7	1.78	31.34
5	17	8	0.90	36.68
6	12	11	2.27	33.49
7	11	12	1.57	27.94
8	11	12	1.82	27.94
9	15	14	5.25	138.87
10	14	15	6.36	113.35
11	8	17	1.78	16.43

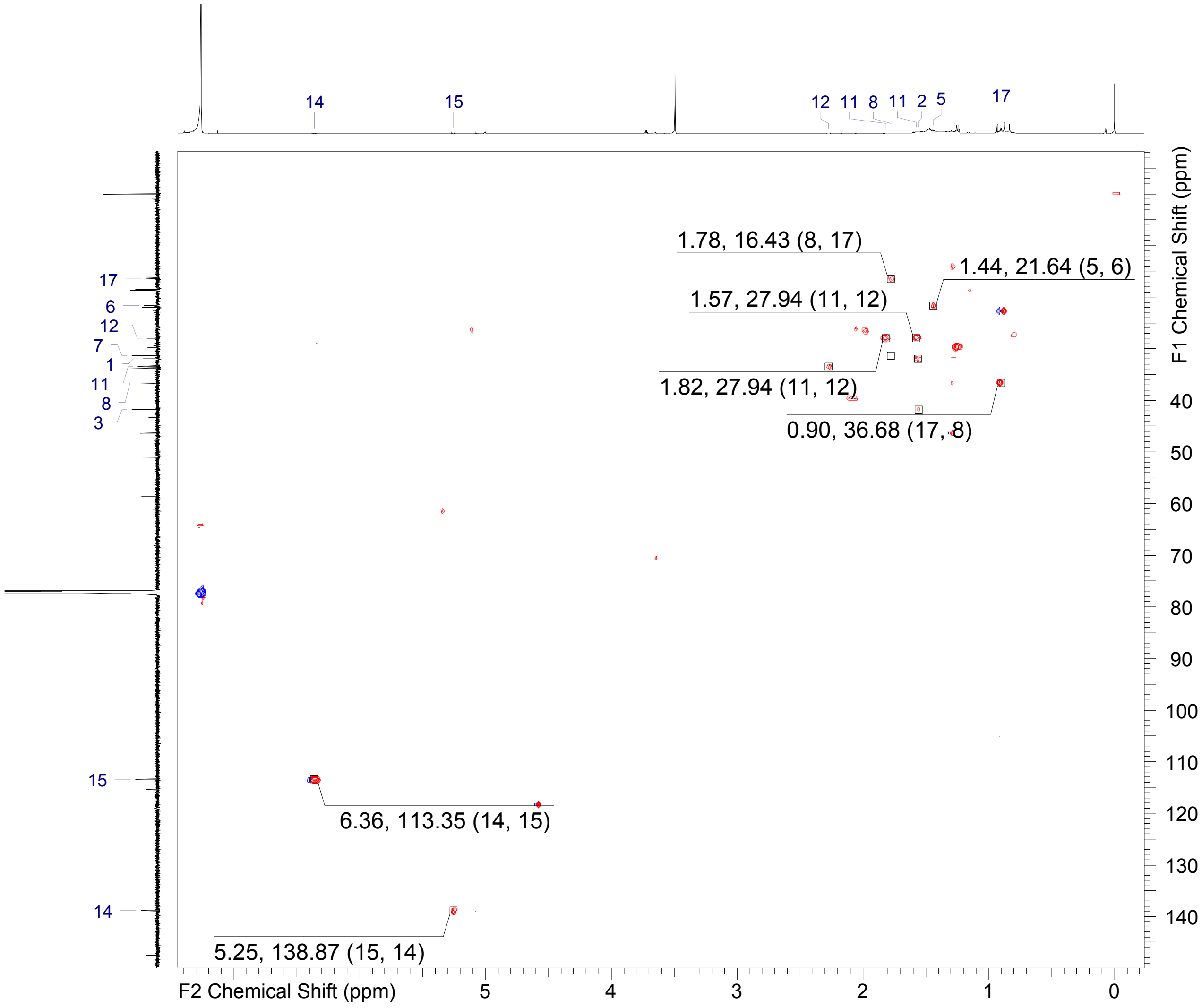

Supplement: Supplementary file 3 — Figure S1. NMR analysis of labda-13(16),14-dien-9-ol (compound 2) formed by the coupled reaction of MvCPS1 and MvELS. (PDF 910 kb) [file 12870_2019_1702_MOESM3_ESM.pdf]
