# Supplementary material for: Functional characterization of the cytochrome P450 monooxygenase CYP71AU87 indicates a role in marrubiin biosynthesis in the medicinal plant Marrubium vulgare
Source: BMC Plant Biol. 2019 Mar 25;19:114. doi: 10.1186/s12870-019-1702-5 (PMC6434833; doi:10.1186/s12870-019-1702-5)
Supplement: Supplementary file 4 — Figure S2. GC-MS analysis of reaction products derived from Nicotiana benthamiana co-expression assays of MvCPS1 and MvELS the P450 candidates Mv1270, Mv1545, Mv4213, Mv6504, and Mv3392, respectively. Shown are extracted ion chromatograms (m/z 151) of the diterpenoid products 9,13-epoxy labd-14-ene 1, labda-13(16),14-dien-9-ol 2, unidentified diterpene 3, 9,13-epoxy labd-14-ene-18-ol 4, and 9,13-epoxy labd-14-ene-19-ol 5. The suppressor of RNA silencing p19 was included in all co-expression assays. (A) Co-expression of MvCPS1, MvELS and MvCYP71AU87, (B) Co-expression of MvCPS1 and MvELS, (C-F) Co-expression of MvCPS1 and MvELS with the P450 Mv1545, Mv4213, Mv6504, and Mv3392, respectively), (G-J) Co-expression of MvCPS1 and MvELS with MvCYP71AU87 and the individual P450 Mv1545, Mv4213, Mv6504, and Mv3392, respectively. (K) Expression of MvCYP71AU87 only. (PDF 264 kb) [file 12870_2019_1702_MOESM4_ESM.pdf]

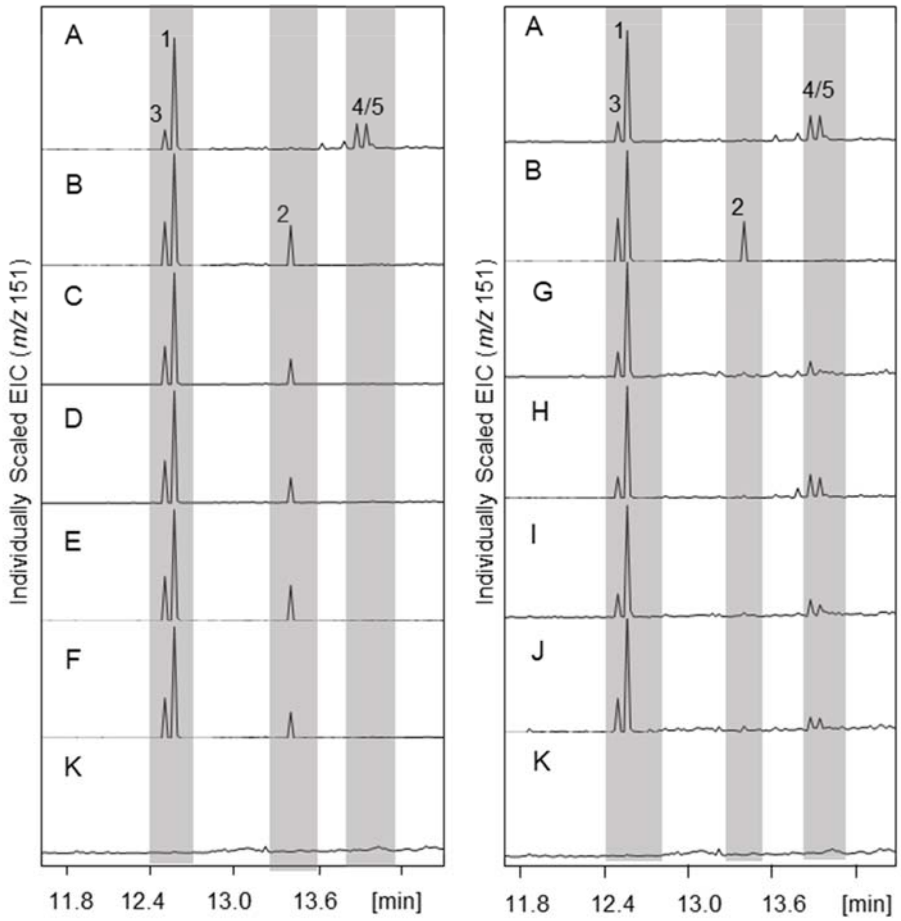


**Additional file 4: Figure S2.** GC-MS analysis of reaction products derived from *Nicotiana benthamiana* co-expression assays of *Mv*CPS1 and *Mv*ELS with the P450 candidates *Mv*1270, *Mv*1545, *Mv*4213, *Mv*6504, and *Mv*3392, respectively. Shown are extracted ion chromatograms (*m*/*z* 151) of the diterpenoid products 9,13-epoxy labd-14-ene **1**, labda-13(16),14-dien-9-ol **2**, unidentified diterpene **3**, 9,13-epoxy labd-14-ene-18-ol **4**, and 9,13-epoxy labd-14-ene-19-ol **5**. The suppressor of RNA silencing p19 was included in all co-expression assays. (**A**) Co-expression of *Mv*CPS1, *Mv*ELS and *Mv*CYP71AU87, (**B**) Co-expression of *Mv*CPS1 and *Mv*ELS, (**C-F**) Co-expression of *Mv*CPS1 and *Mv*ELS with the P450 *Mv*1545, *Mv*4213, *Mv*6504, and *Mv*3392, respectively), (**G-J**) Co-expression of *Mv*CPS1 and *Mv*ELS with *Mv*CYP71AU87 and the individual P450 *Mv*1545, *Mv*4213, *Mv*6504, and *Mv*3392, respectively. (**K**) Expression of *Mv*CYP71AU87 only.
