# Supplementary material for: Functional characterization of the cytochrome P450 monooxygenase CYP71AU87 indicates a role in marrubiin biosynthesis in the medicinal plant Marrubium vulgare
Source: BMC Plant Biol. 2019 Mar 25;19:114. doi: 10.1186/s12870-019-1702-5 (PMC6434833; doi:10.1186/s12870-019-1702-5)
Supplement: Supplementary file 5 — Figure S3. NMR analysis of 9,13-epoxy-labd-14-ene-19-ol (compounds 4/5) formed by the coupled reaction of MvCPS1, MvELS and CYP71AU87. (PDF 1415 kb) [file 12870_2019_1702_MOESM5_ESM.pdf]

Supplemental Fig. S3: NMR analysis of 9,13-epoxy-labd-14-ene-19-ol (compound 4/5) formed by the coupled reaction of MvCPS1, MvELS and MvCYP71AU87.

A) 1D NMR analysis

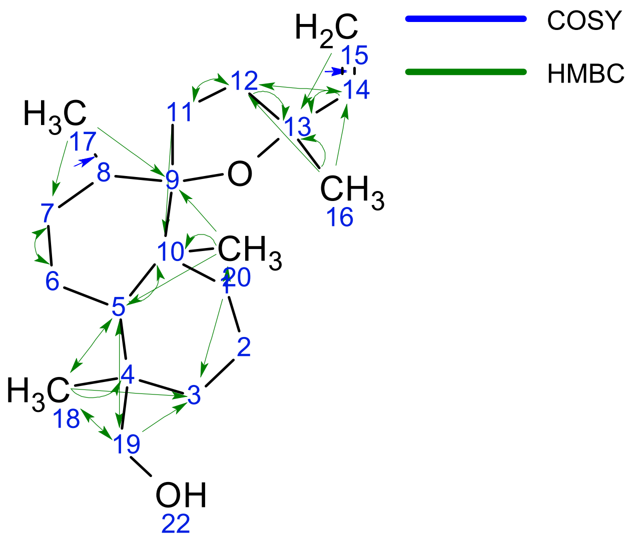

#	Atom#	C Label	C Shift	XHn	H Label	H Shift	C Calc Shift (Neural Net)	H Calc Shift (Neural Net)	H Multiplicity	COSY	H HMBC	C HMBC
1	18	C 1	17.472	CH3	H 1	0.746	23.276	0.930	u		5, 19	3, 4, 5, 19
2	20	C 2	18.056	CH3	H 3	0.877	22.443	0.943	d (4.60)			1, 5, 10, 9
3	17	C 3	18.079	CH3	H 2	0.778	17.553	0.920	d (6.64)	8		7, 8, 9
4	2	C 4	18.316	CH2	H 12	1.556	18.341	1.440	u			
5	6	C 5	21.903	CH2	H 10	1.394	22.007	1.432	m		7	7
6	6	C 5	21.903	CH2	H 7	1.304	22.007	1.432	m		7	
7	16	C 6	28.984	CH3	H 6	1.285	27.235	1.351	m			12, 13, 14
8	11	C 7	29.353	CH2	H 14	1.711	28.627	1.809	m		12	
9	11	C 7	29.353	CH2	H 17	1.933	28.627	1.809	u		12	12, 10
10	7	C 8	31.463	CH2	H 9	1.373	31.590	1.463	m		17, 6	6
11	7	C 8	31.463	CH2	H 8	1.308	31.590	1.463	m		17, 6	
12	1	C 9	33.090	CH2	H 11	1.456	36.163	1.370	m		20	
13	1	C 9	33.090	CH2	H 4	1.174	36.163	1.370	m		20	3
14	3	C 10	35.269	CH2	H 5	1.255	36.661	1.225	m		18, 1, 19	
15	12	C 11	36.406	CH2	H 18	1.966	36.409	1.802	m		16, 11, 14	11, 13, 14
16	12	C 11	36.406	CH2	H 15	1.715	36.409	1.802	m		16, 11, 14	13
17	8	C 12	36.737	CH	H 13	1.669	36.264	1.954	m	17	17	
18	4	C 13	37.797	C			38.980				18	
19	5	C 14	40.384	CH	H 16	1.750	43.035	1.603	dd (12.77, 2.72)		18, 20, 19	18, 10, 19

A) 1D NMR analysis continued

#	Atom#	C Label	C Shift	XHn	H Label	H Shift	C Calc Shift (Neural Net)	H Calc Shift (Neural Net)	H Multiplicity	COSY	H HMBC	C HMBC
20	10	C 15	42.625	C			43.562				20, 5, 11	
21	19	C 16	72.660	CH2	H 19	3.119	70.863	3.417	d (4.43)	19	18, 5	18, 3, 5
22	19	C 16	72.660	CH2	H 20	3.367	70.863	3.417	d (2.04)	19	18, 5	
23	13	C 17	83.731	C			82.297				16, 12, 12, 15, 15, 14	
24	9	C 18	92.856	C			92.773				17, 20	
25	15	C 19	109.838	CH2	H 21	4.910	112.937	4.935	dd (5.19, 1.45)	14		13
26	15	C 19	109.838	CH2	H 22	5.061	112.937	5.066	dd (17.54, 1.19)			13, 14
27	14	C 20	146.053	CH	H 23	6.047	143.390	5.871	d (10.90)	15	16, 12, 15	12, 13

B) ^{1}H NMR analysis

Acquisition Time (sec)	2.9360	***Comment***	1H NMR	***D***	0.0002
D1	2	***DE***	18	***DS***	2
Date	29 Nov 2016 14:44:53			***Date Stamp***	29 Nov 2016 14:44:53
File Name	\\169.237.229.248\share$\jbaddiso\zerbe\Prema\11292016-Tob1270-P1-P7-Trial1\2\PDATA\1\1r				
Frequency (MHz)	800.1500	***GB***	0	***INSTRUM***	<spect>
LB	0	***NS***	16	***Nucleus***	1H
Number of Transients	16	***Origin***	spect	***Original Points Count***	32768
Owner	pkarunan	***PC***	4		
PROBHD	<5 mm CPTCI 1H-13C/15N/D Z-GRD Z107231/0001 >				
PULPROG	<zg30>	***Points Count***	65536	***Pulse Sequence***	zg30
Receiver Gain	40.30	***SF***	800.15	***SFO1***	800.15400075
SI	65536	***SSB***	0	***SW(cyclical) (Hz)***	11160.71
SWH	11160.7142857143			***Solvent***	CHLOROFORM-d
Spectrum Offset (Hz)	3963.7847	***Spectrum Type***	standard	***Sweep Width (Hz)***	11160.54
TD	65536	***TD0***	1	***TE***	303
Temperature (degree C)	30.000	***UNC1***	<1H>	***WDW***	0

^{1}H NMR (800 MHz, CHLOROFORM-*d*) δ ppm 0.78 (d, *J*=6.64 Hz, 4 H) 0.87 (d, *J*=4.60 Hz, 5 H) 1.16 - 1.19 (m, 1 H) 1.28 - 1.29 (m, 5 H) 1.29 - 1.32 (m, 4 H) 1.36 - 1.39 (m, 3 H) 1.44 - 1.47 (m, 2 H) 1.65 - 1.69 (m, 3 H) 1.71 - 1.73 (m, 2 H) 1.75 (dd, *J*=12.77, 2.72 Hz, 1 H) 1.96 - 1.98 (m, 1 H) 3.12 (d, *J*=4.43 Hz, 1 H) 3.37 (d, *J*=2.04 Hz, 1 H) 4.91 (dd, *J*=5.19, 1.45 Hz, 1 H) 5.06 (dd, *J*=17.54, 1.19 Hz, 1 H) 6.05 (d, *J*=10.90 Hz, 1 H)

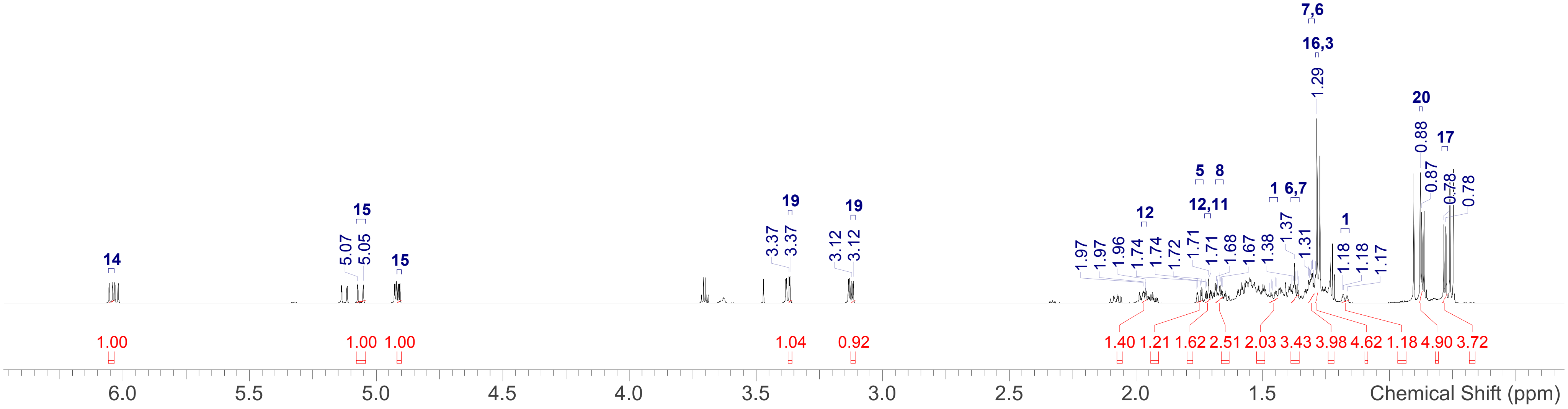

C) ^{13}C NMR analysis

Acquisition Time (sec)	0.6816	***Comment***	13C NMR-test-8000scans
D	1.5	***D1***	1.5
DE	33.15148	***DS***	4
Date	29 Nov 2016 20:29:50		
Date Stamp	29 Nov 2016 20:29:50		
File Name	\\169.237.229.248\share$\jbaddiso\zerbe\Prema\11292016-Tob1270-P1-P7-Trial1\4\fid		
Frequency (MHz)	201.2200	***GB***	0
INSTRUM	<spect>	***LB***	0.3
NS	8192	***Nucleus***	13C
Number of Transients	8192	***Origin***	spect
Original Points Count	32768	***Owner***	pkarunan
PC	1.4		
PROBHD	<5 mm CPTCI 1H-13C/15N/D Z-GRD Z107231/0001 >		
PULPROG	<zgpg30>	***Points Count***	32768
Pulse Sequence	zgpg30	***Receiver Gain***	2050.00
SF	201.19787753		
SFO1	201.220009296528		
SI	65536	***SSB***	0
SW(cyclical) (Hz)	48076.92	***SWH***	48076.9230769231
Solvent	CHLOROFORM-d		
Spectrum Offset (Hz)	22126.3730	***Spectrum Type***	standard
Sweep Width (Hz)	48075.45	***TD***	65536
TD0	1	***TE***	303
Temperature (degree C)	30.000	***UNC1***	<13C>
WDW	1		

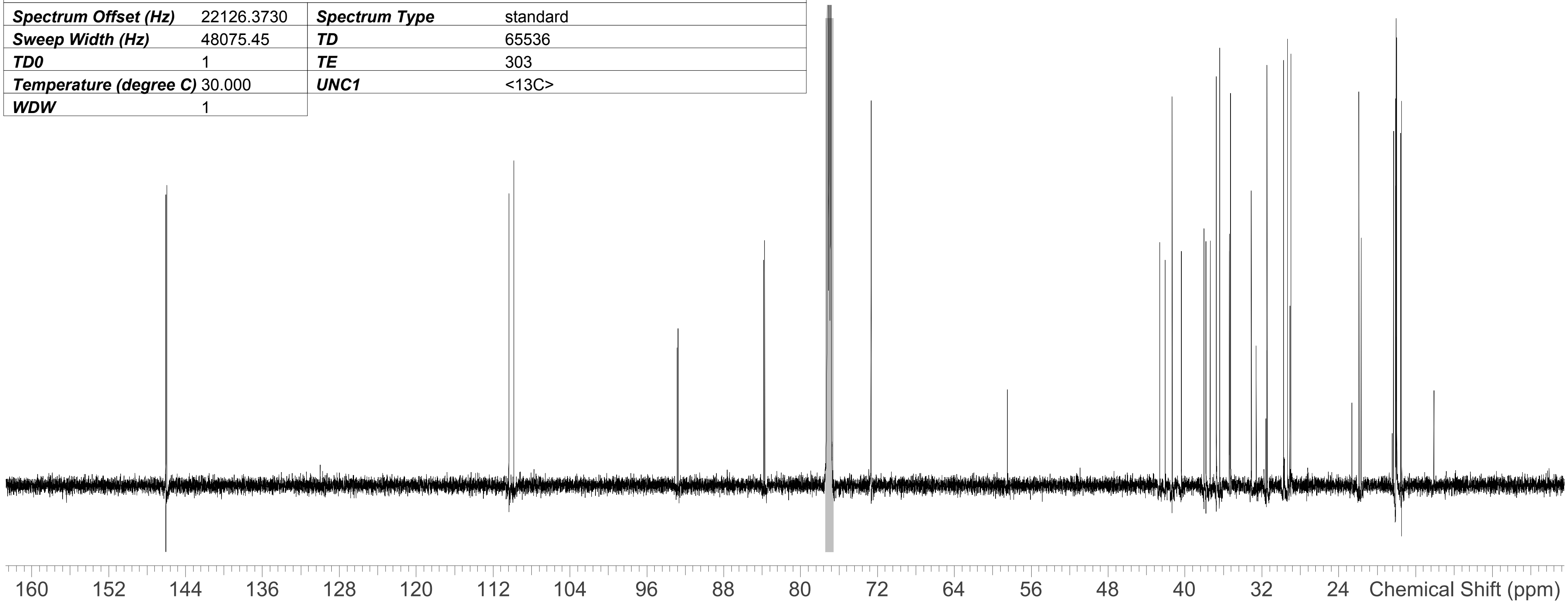

D) HSQC NMR analysis

Acquisition Time (sec)	(0.1348, 0.0068)	***Comment***	HSQC NMR
Constant (Hz)	145.0	***Date***	05 Dec 2016 13:34:40
File Name	\\169.237.229.248\share$\jbaddiso\zerbe\Prema\11292016-Tob1270-P1-P7-Trial1\13\PDATA\1\2rr		
Frequency (MHz)	(800.1500, 201.1979)		
Nucleus	(1H, 13C)	***Number of Transients***	8
Origin	spect	***Original Points Count***	(1024, 256)
Owner	pkarunan	***Points Count***	(4096, 1024)
Pulse Sequence	hsqcedetgpsp.3	***Solvent***	CHLOROFORM-d
Spectrum Type	HSQC-DEPT	***Sweep Width (Hz)***	(7596.93, 37841.80)
Temperature (degree C)	30.000	***Title***	HSQC NMR

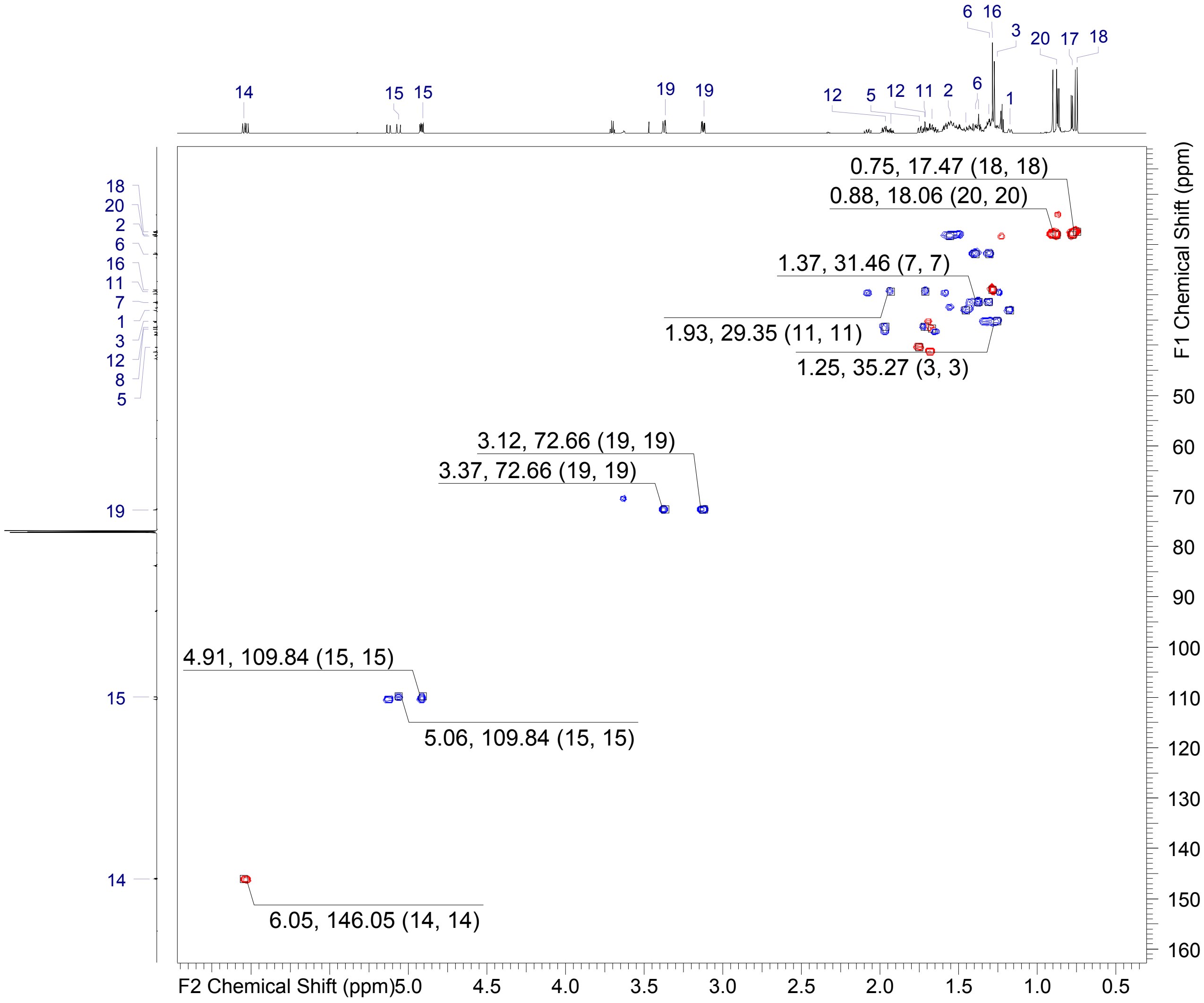

No.	F2 Atom	F1 Atom	F2 (ppm)	F1 (ppm)
1	1	1	1.46	33.09
2	1	1	1.17	33.09
3	2	2	1.56	18.32
4	3	3	1.25	35.27
5	5	5	1.75	40.38
6	6	6	1.30	21.90
7	6	6	1.39	21.90
8	7	7	1.31	31.46
9	7	7	1.37	31.46
10	8	8	1.67	36.74
11	11	11	1.93	29.35
12	11	11	1.71	29.36
13	12	12	1.72	36.41
14	12	12	1.97	36.41
15	14	14	6.05	146.05
16	15	15	4.91	109.84
17	15	15	5.06	109.84
18	16	16	1.29	28.98
19	17	17	0.78	18.08
20	18	18	0.75	17.47
21	19	19	3.12	72.66
22	19	19	3.37	72.66
23	20	20	0.88	18.06

E) SHSQC NMR analysis

Acquisition Time (sec)	(0.1065, 0.0318)	***Comment***	SHSQC-32scans-12-40ppm
Constant (Hz)	125.0	***Date***	14 Dec 2016 01:53:32
File Name	\\169.237.229.248\share$\jbaddiso\zerbe\Prema\12132016-Tob1270-P1-P7-Selective\4\ser		
Frequency (MHz)	(800.1500, 201.1979)	***Nucleus***	(1H, 13C)
Number of Transients	32	***Origin***	spect
Original Points Count	(1024, 256)	***Owner***	pkarunan
Points Count	(1024, 1024)	***Pulse Sequence***	shsqcetgpsisp2.2
Solvent	CHLOROFORM-d	***Spectrum Type***	HSQC
Sweep Width (Hz)	(9606.00, 8043.67)	***Temperature (degree C)***	30.000
Title	SHSQC-32scans-12-40ppm		

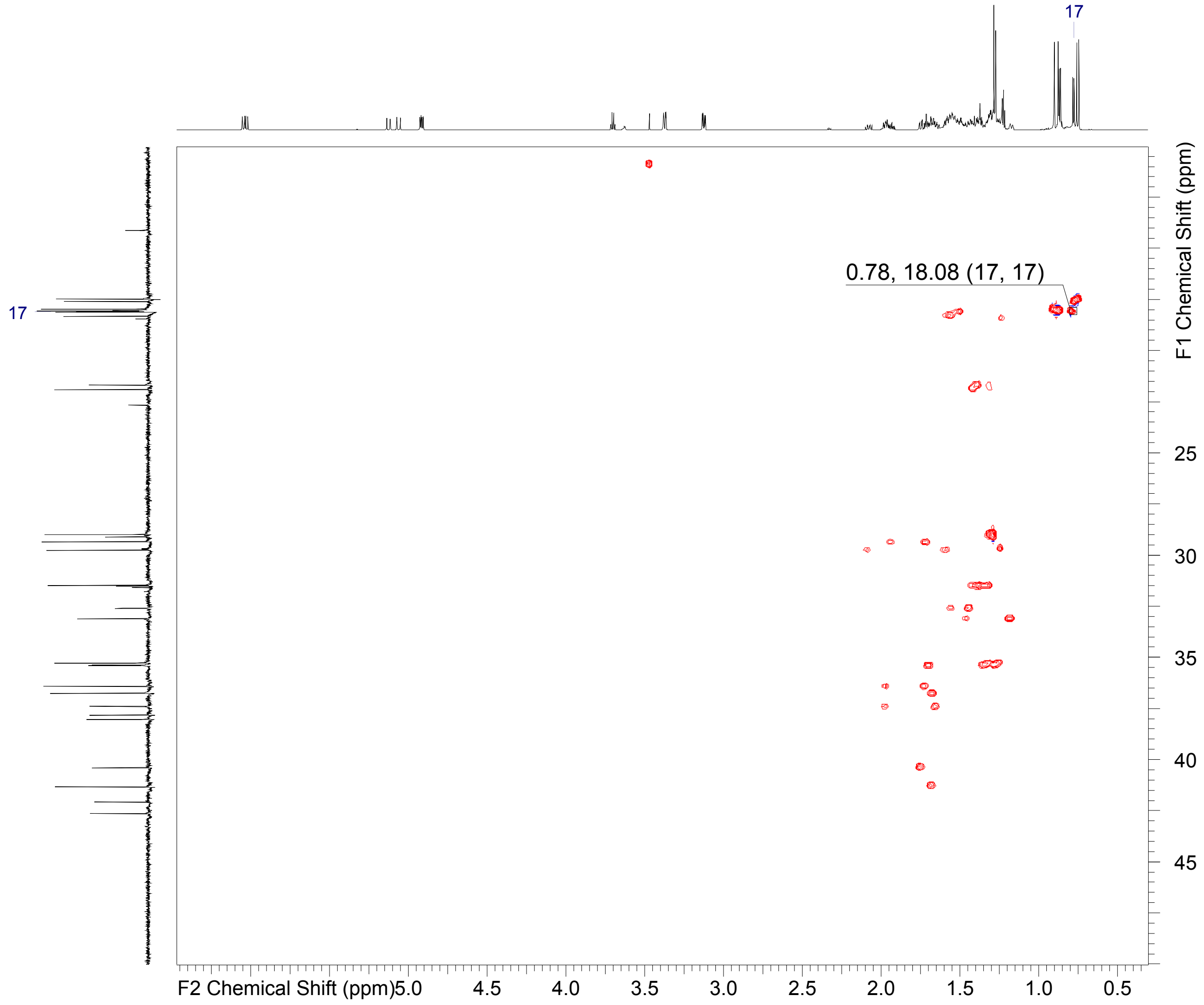

No.	F2 Atom	F1 Atom	F2 (ppm)	F1 (ppm)
1	17	17	0.78	18.08

F) COSY NMR analysis

Acquisition Time (sec)	(0.2763, 0.0346)	***Comment***	COSY
Date	30 Nov 2016 09:51:38		
File Name	\\169.237.229.248\share$\jbaddiso\zerbe\Prema\11292016-Tob1270-P1-P7-Trial1\17\ser		
Frequency (MHz)	(800.1500, 800.1500)		
Nucleus	(1H, 1H)	***Number of Transients***	8
Origin	spect	***Original Points Count***	(2048, 256)
Owner	pkarunan	***Points Count***	(4096, 1024)
Pulse Sequence	cosygpmfppqf	***Solvent***	CHLOROFORM-d
Spectrum Type	COSY	***Sweep Width (Hz)***	(7409.26, 7400.17)
Temperature (degree C)	30.000	***Title***	COSY

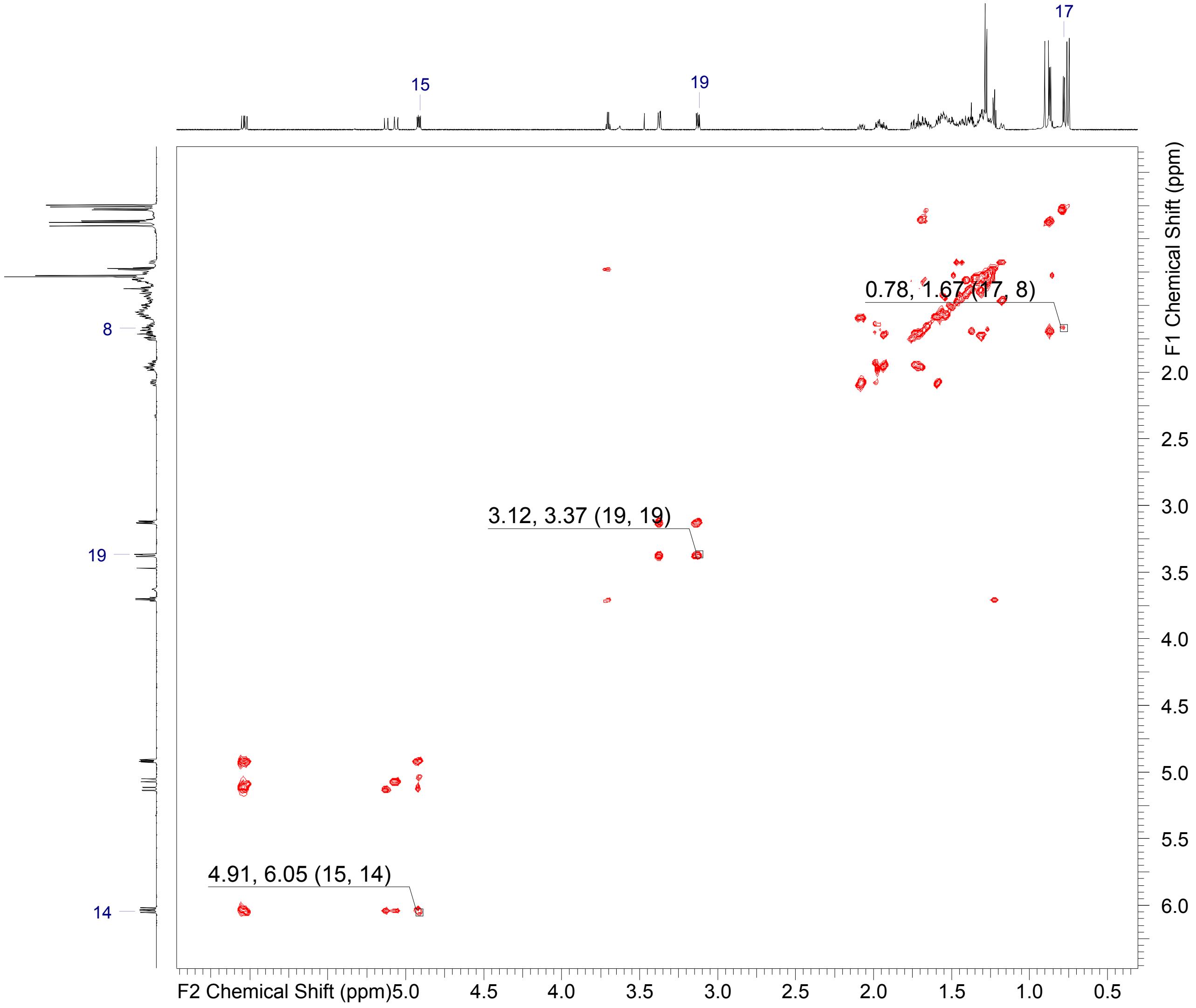

No.	F2 Atom	F1 Atom	F2 (ppm)	F1 (ppm)
1	17	8	0.78	1.67
2	15	14	4.91	6.05
3	19	19	3.12	3.37

G) HMBC NMR analysis

Acquisition Time (sec)	(0.2695, 0.0068)	***Comment***	HMBC-regular
Constant (Hz)	8.0	***Date***	30 Nov 2016 04:40:06
File Name	\\169.237.229.248\share$\jbaddiso\zerbe\Prema\11292016-Tob1270-P1-P7-Trial1\15\ser		
Frequency (MHz)	(800.1500, 201.1979)		
Nucleus	(1H, 13C)	***Number of Transients***	32
Origin	spect	***Original Points Count***	(2048, 256)
Owner	pkarunan	***Points Count***	(4096, 1024)
Pulse Sequence	hmbcetgpl3nd	***Solvent***	CHLOROFORM-d
Spectrum Type	HMBC	***Sweep Width (Hz)***	(7596.93, 37841.80)
Temperature (degree C)	30.000	***Title***	HMBC-regular

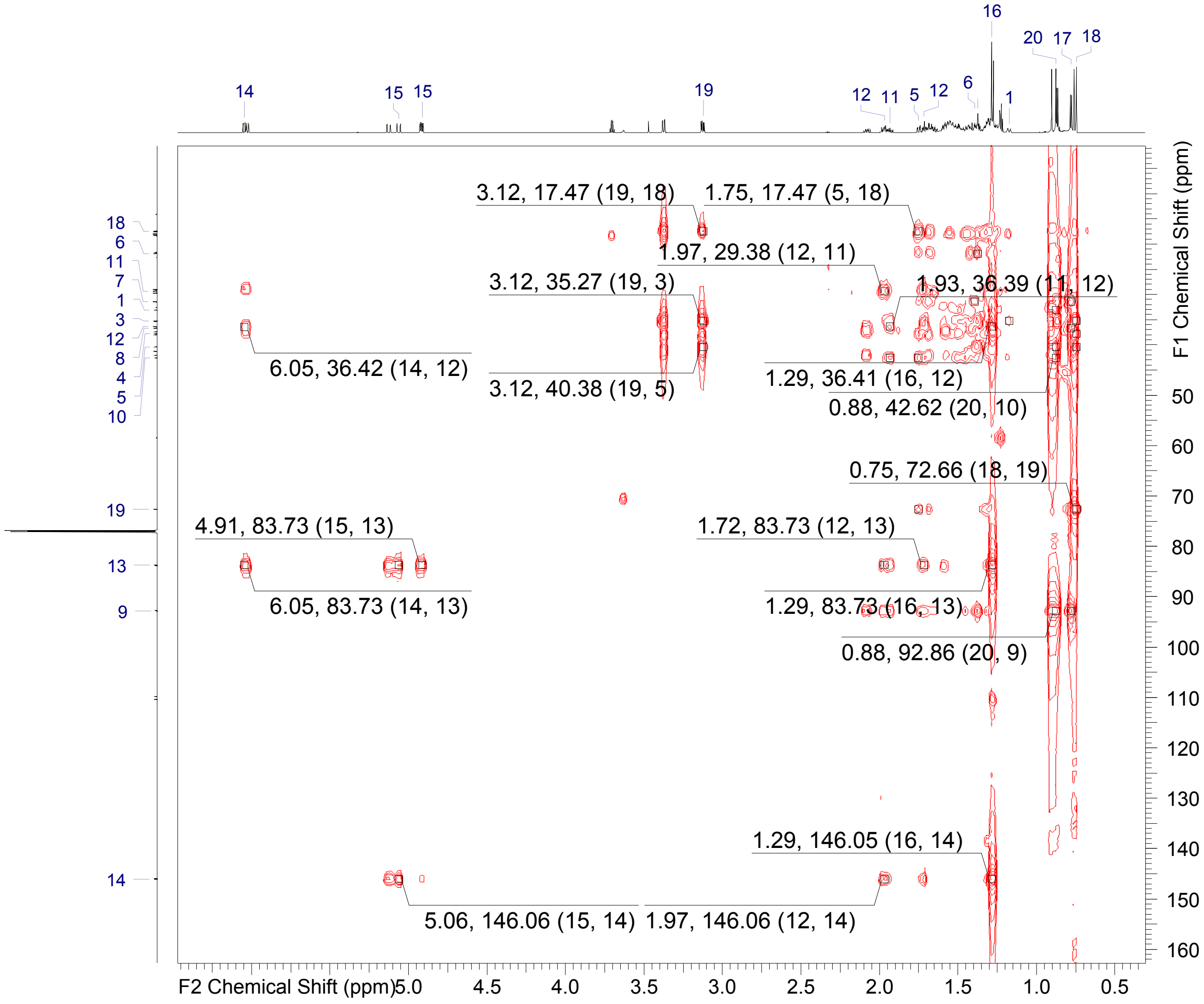

No.	F2 Atom	F1 Atom	F2 (ppm)	F1 (ppm)
1	20	1	0.88	33.09
2	1	3	1.17	35.27
3	18	3	0.75	35.27
4	19	3	3.12	35.27
5	18	4	0.75	37.80
6	18	5	0.75	40.38
7	19	5	3.12	40.38
8	20	5	0.88	40.38
9	7	6	1.37	21.90
10	6	7	1.39	31.46
11	17	7	0.78	31.46
12	17	8	0.78	36.74
13	17	9	0.78	92.86
14	20	9	0.88	92.86
15	5	10	1.75	42.62
16	11	10	1.93	42.63
17	20	10	0.88	42.62
18	12	11	1.97	29.38
19	11	12	1.93	36.39
20	14	12	6.05	36.42
21	16	12	1.29	36.41
22	12	13	1.72	83.73
23	12	13	1.97	83.74
24	14	13	6.05	83.73
25	15	13	5.06	83.73
26	15	13	4.91	83.73
27	16	13	1.29	83.73
28	12	14	1.97	146.06
29	15	14	5.06	146.06
30	16	14	1.29	146.05
31	5	18	1.75	17.47
32	19	18	3.12	17.47
33	5	19	1.75	72.66
34	18	19	0.75	72.66

H) SHMBC NMR analysis

Acquisition Time (sec)	(0.1065, 0.0318)	***Comment***	SHMBC-64scans-12-40ppm
Constant (Hz)	8.0	***Date***	14 Dec 2016 09:38:10
File Name	\\169.237.229.248\share$\jbaddiso\zerbe\Prema\12132016-Tob1270-P1-P7-Selective\5\ser		
Frequency (MHz)	(800.1500, 201.1979)	***Nucleus***	(1H, 13C)
Number of Transients	64	***Origin***	spect
Original Points Count	(1024, 256)	***Owner***	pkarunan
Points Count	(2048, 512)	***Pulse Sequence***	shmbcctetgpl2nd
Solvent	CHLOROFORM-d	***Spectrum Type***	HMBC
Sweep Width (Hz)	(9610.69, 8035.80)	***Temperature (degree C)***	30.000
Title	SHMBC-64scans-12-40ppm		

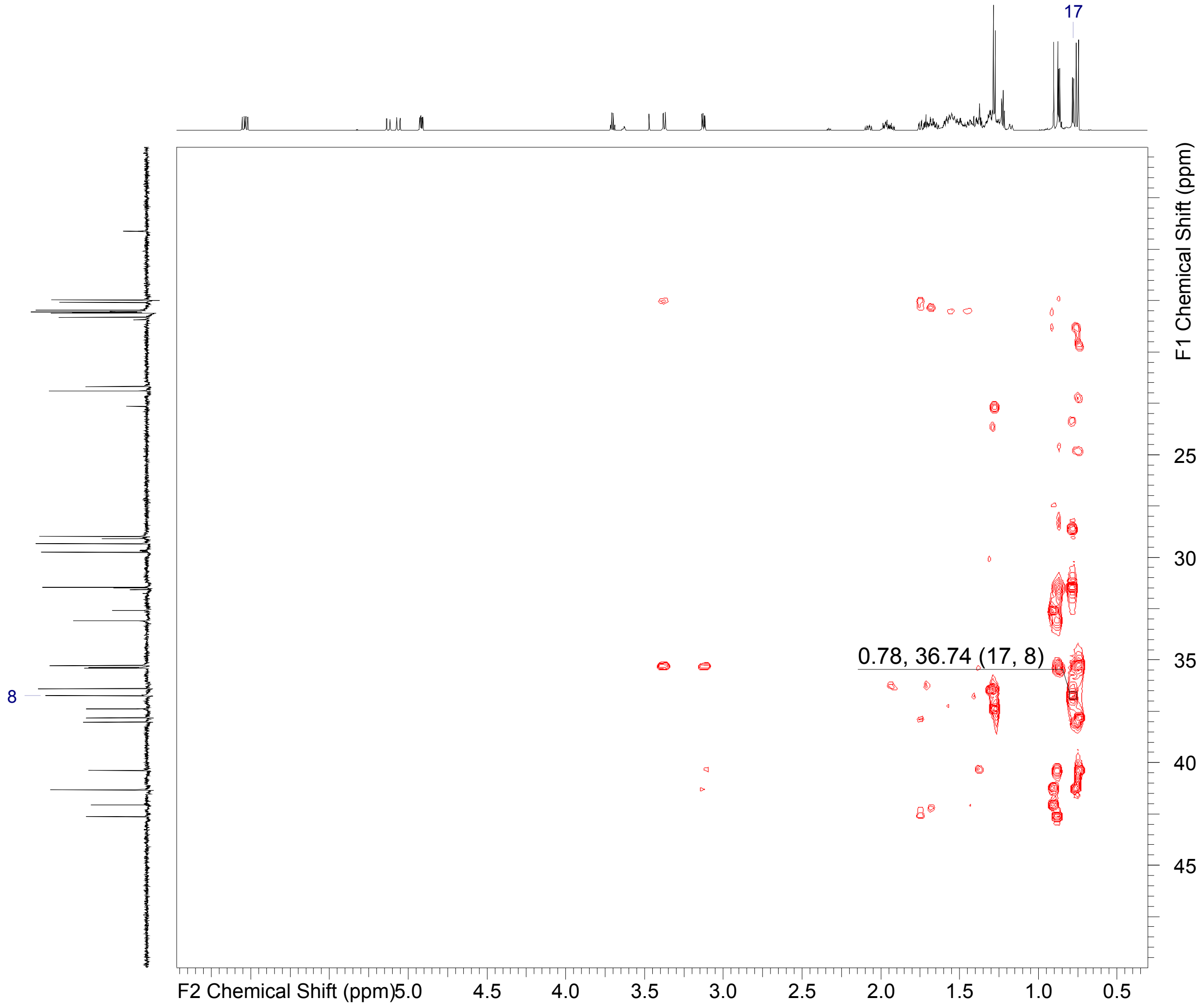

No.	F2 Atom	F1 Atom	F2 (ppm)	F1 (ppm)
1	17	8	0.78	36.74
