# Supplementary material for: Functional characterization of the cytochrome P450 monooxygenase CYP71AU87 indicates a role in marrubiin biosynthesis in the medicinal plant Marrubium vulgare
Source: BMC Plant Biol. 2019 Mar 25;19:114. doi: 10.1186/s12870-019-1702-5 (PMC6434833; doi:10.1186/s12870-019-1702-5)
Supplement: Supplementary file 6 — Figure S4. Co-expression of MvCPS1, MvELS and CYP71AU87 in yeast (Saccharomyces cerevisiae). Shown are extracted ion chromatograms (m/z 151) and mass spectra of the reaction products resulting from co-expression of MvCPS1, MvELS, CYP71AU87, MvCPR and the endogenous yeast GGPP synthase BTS1 in the yeast strain AM94. 9,13-epoxy labd-14-ene 1, labda-13(16),14-dien-9-ol 2, 9,13-epoxy labd-14-ene-18-ol 4, 9,13-epoxy labd-14-ene-19-ol 5, peregrinol (i.e. dephosphorylated peregrinol diphosphate) 6, unidentified compounds 7, geranylgeraniol (i.e. dephosphorylated GGPP) 8. (PDF 451 kb) [file 12870_2019_1702_MOESM6_ESM.pdf]

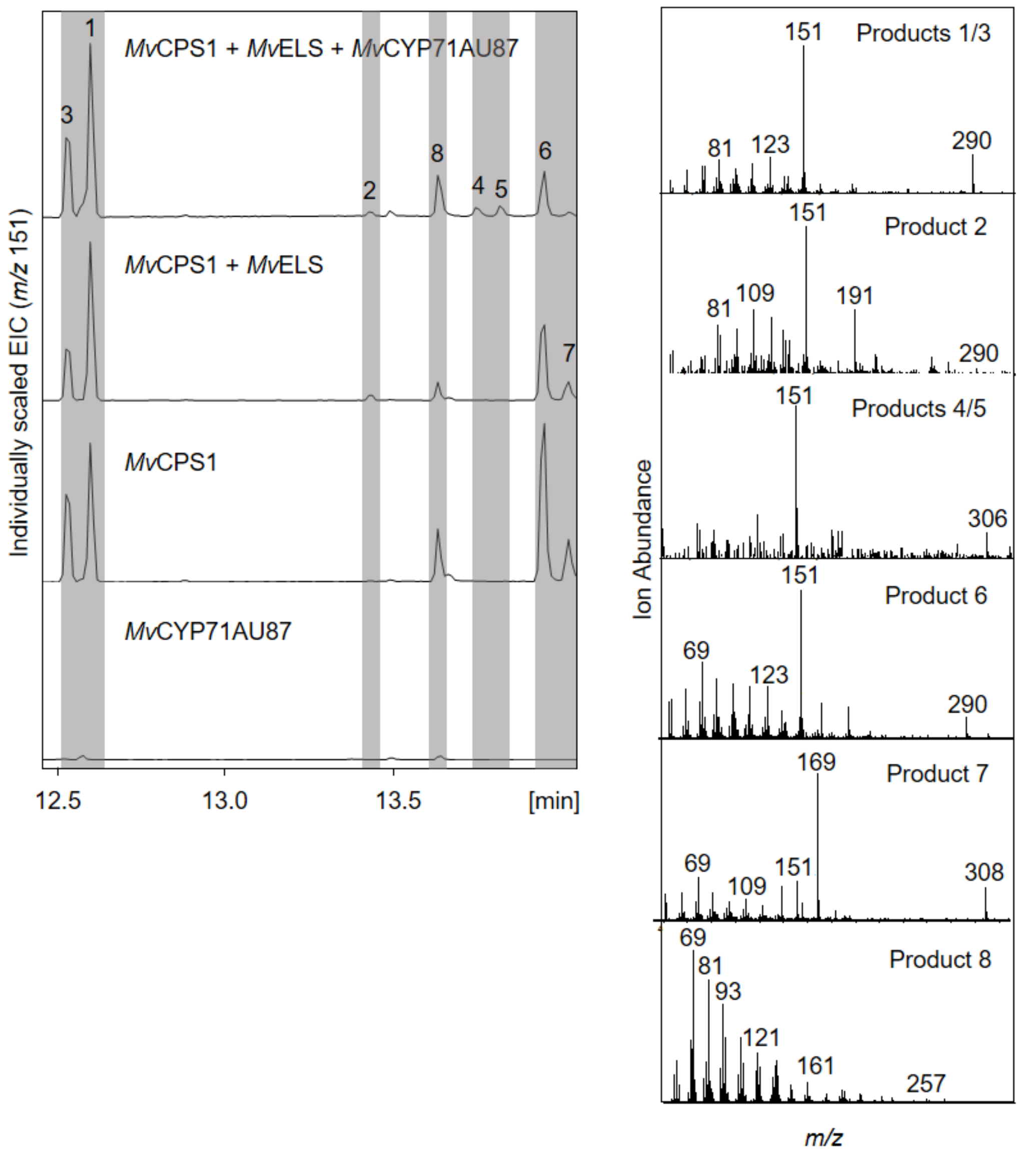

Additional file 6: Figure S4. Co-expression of *Mv*CPS1, *Mv*ELS and CYP71AU87 in yeast (*Saccharomyces cerevisiae*). Shown are extracted ion chromatograms (*m/z* 151) and mass spectra of the reaction products resulting from co-expression of *Mv*CPS1, *Mv*ELS, CYP71AU87, *Mv*CPR and the endogenous yeast GGPP synthase BTS1 in the yeast strain AM94. 9,13-epoxy labd-14-ene **1**, labda-13(16),14-dien-9-ol **2**, 9,13-epoxy labd-14-ene-18-ol **4**, 9,13-epoxy labd-14-ene-19-ol **5**, peregrinol (i.e. dephosphorylated peregrinol diphosphate) **6**, unidentified compounds **7**, geranylgeraniol (i.e. dephosphorylated GGPP) **8**.
