# Supplementary material for: Functional characterization of the cytochrome P450 monooxygenase CYP71AU87 indicates a role in marrubiin biosynthesis in the medicinal plant Marrubium vulgare
Source: BMC Plant Biol. 2019 Mar 25;19:114. doi: 10.1186/s12870-019-1702-5 (PMC6434833; doi:10.1186/s12870-019-1702-5)
Supplement: Supplementary file 7 — Figure S5. Mass spectra and chemical structures for diterpenoids observed in N. benthamiana co-expression assays to test CYP71AU87 substrate promiscuity (see Fig. 6). Transient N. benthamiana co-expression assays were performed by combining MvCYP71AU87 and MvELS with different class II diTPSs, including the maize (Zea mays) ent-CPP synthase ZmAN2 to produce the gibberellin precursor ent-kaurene (compound 9), a LPP synthase from Grindelia robusta (GrLPPS) to produce manoyl oxide (compound 10), and a (+)-CPP synthase from Isodon rubescens (IrTPS3) to form miltiradiene (compound 11). (PDF 322 kb) [file 12870_2019_1702_MOESM7_ESM.pdf]

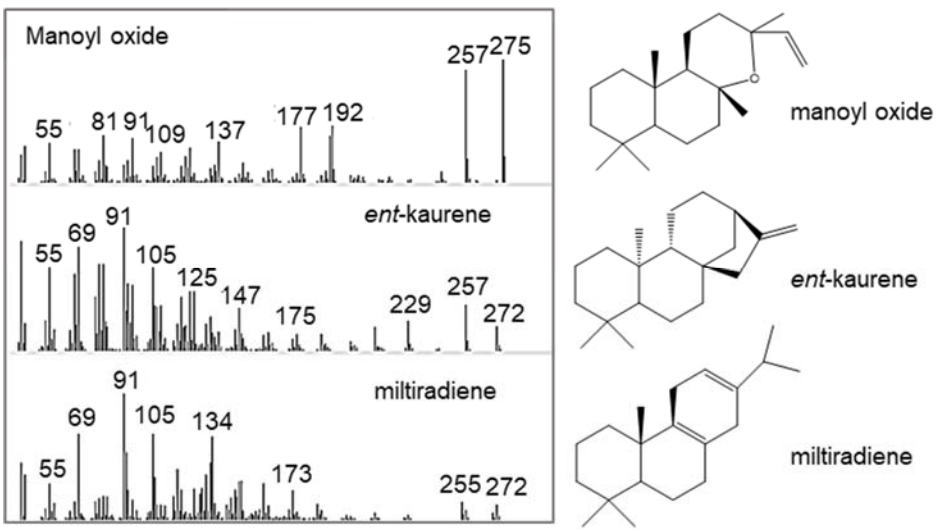

Additional file 7: Figure S5. Mass spectra and chemical structures for diterpenoids observed in *N. benthamiana* co-expression assays to test CYP71AU87 substrate promiscuity (see Figure 6). Transient *N. benthamiana* co-expression assays were performed by combining *Mv*CYP71AU87 and *Mv*ELS with different class II diTPSs, including the maize (*Zea mays*) *ent*-CPP synthase *Zm*AN2 to produce the gibberellin precursor *ent*-kaurene (compound **9**) [Harris et al. (2005) Plant Mol Biol 59:881-94], a LPP synthase from *Grindelia robusta* (*Gr*LPPS) [Zerbe et al. (2013) Plant Physiol 162:1073-91] to produce manoyl oxide (compound **10**), and a (+)-CPP synthase from *Isodon rubescens* (*Ir*TPS3) [Pelot et al. (2017) PLoS One 12:e0176507] to form miltiradiene (compound **11**).
