# Supplementary material for: Functional characterization of the cytochrome P450 monooxygenase CYP71AU87 indicates a role in marrubiin biosynthesis in the medicinal plant Marrubium vulgare
Source: BMC Plant Biol. 2019 Mar 25;19:114. doi: 10.1186/s12870-019-1702-5 (PMC6434833; doi:10.1186/s12870-019-1702-5)
Supplement: Supplementary file 8 — Figure S6. LC-MS analysis of CYP71AU87 products in M. vulgare leaves and flowers. (A) Leaf and flower tissue from 12-week-old M. vulgare plants was extracted with 20% (v/v) ethyl acetate in hexane, and then dried and re-suspended in MeOH. Extracts were analyzed by LC-MS on a Q Exactive HF mass spectrometer coupled with a Vanquish LC system (Thermo Scientific). A volume of 5 μl was separated on a Waters Acquity UPLC CSH C18 column (100 × 2.1 mm; 1.7 μm) coupled to an Acquity UPLC CSH C18 VanGuard precolumn (5 × 2.1 mm; 1.7 μm) at 65 °C and a flow rate of 0.6 ml min− 1. The mobile phases consisted of A: acetonitrile:water (60:40, v/v) with ammonium formate (10 mM) and formic acid (0.1%) and B: 2-propanol:acetonitrile (90:10, v/v) with ammonium formate (10 mM) and formic acid (0.1%). The 15 min separation was conducted under the following gradient: 0 min 15% B; 0–2 min 30% B; 2–2.5 min 48% B;2.5–11 min 82% B; 11–11.5 min 99% B; 11.5–12 min 99% B; 12–12.1 min 15% B; 12.1–15 min 15% B. Orbitrap MS instrument was operated in electrospray ionization (ESI) in positive mode with the following parameters: mass range 60–900 m/z; spray voltage 3.6 kV, sheath gas (nitrogen) flow rate 60 units; auxiliary gas (nitrogen) flow rate 25 units, capillary temperature 320 °C, full scan MS1 mass resolving power 120,000, data-dependent MSMS (dd-MSMS) 4 scans per cycle, dd-MSMS mass resolving power 30,000. Thermo Xcalibur 4.0.27.19 was used for data acquisition and analysis. (B) Structures for CYP71AU87 products. (C) CYP71AU87 products were detectable in ESI(+) MS mode, annotated with accurate mass (< 5 ppm) with a retention time of 2.21 min and a parental mass ion of 324.2893 consistent with the addition of an ammonium ion (monoisotopic mass of the CYP71AU87 products 4/5 = 306.2559). (PDF 205 kb) [file 12870_2019_1702_MOESM8_ESM.pdf]

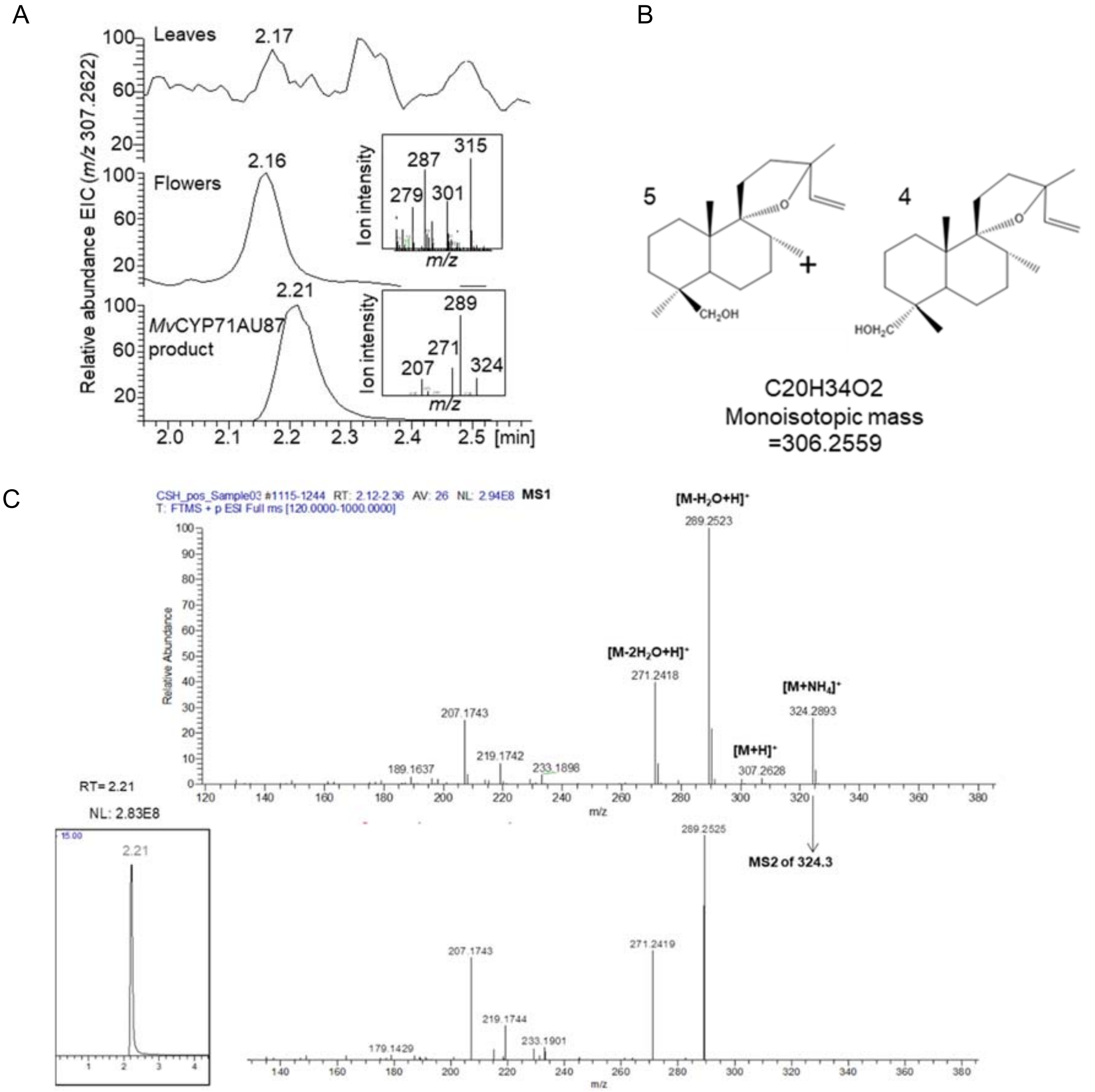

Additional file 8: Figure S6. LC-MS analysis of CYP71AU87 products in *M. vulgare* leaves and flowers. (**A**) Leaf and flower tissue from 12-week-old *M. vulgare* plants was extracted with 20% (v/v) ethyl acetate in hexane, and then dried and re-suspended in MeOH. Extracts were analyzed by LC-MS on a Q Exactive HF mass spectrometer coupled with a Vanquish LC system (Thermo Scientific). A volume of 5 µl was separated on a Waters Acquity UPLC CSH C_{18} column (100 × 2.1 mm; 1.7 µm) coupled to an Acquity UPLC CSH C_{18} VanGuard precolumn (5 × 2.1 mm; 1.7 µm) at 65°C and a flow rate of 0.6 ml min^{-1}. The mobile phases consisted of A: acetonitrile:water (60:40, v/v) with ammonium formate (10 mM) and formic acid (0.1%) and B: 2-propanol:acetonitrile (90:10, v/v) with ammonium formate (10 mM) and formic acid (0.1%). The 15 min separation was conducted under the following gradient: 0 min 15% B; 0−2 min 30% B; 2−2.5 min 48% B;2.5−11 min 82% B; 11−11.5 min 99% B; 11.5−12 min 99% B; 12−12.1 min 15% B; 12.1−15 min 15% B. Orbitrap MS instrument was operated in electrospray ionization (ESI) in positive mode with the following parameters: mass range 60−900 *m*/*z*; spray voltage 3.6 kV, sheath gas (nitrogen) flow rate 60 units; auxiliary gas (nitrogen) flow rate 25 units, capillary temperature 320°C, full scan MS1 mass resolving power 120,000, data-dependent MSMS (dd-MSMS) 4 scans per cycle, dd-MSMS mass resolving power 30,000. Thermo Xcalibur 4.0.27.19 was used for data acquisition and analysis [5]. (**B**) Structures for CYP71AU87 products. (**C**) CYP71AU87 products were detectable in ESI(+) MS mode, annotated with accurate mass (<5ppm) with a retention time of 2.21 min and a parental mass ion of 324.2893 consistent with the addition of an ammonium ion (monoisotopic mass of the CYP71AU87 products **4**/**5** = 306.2559).
