# Supplementary material for: Functional characterization of the cytochrome P450 monooxygenase CYP71AU87 indicates a role in marrubiin biosynthesis in the medicinal plant Marrubium vulgare
Source: BMC Plant Biol. 2019 Mar 25;19:114. doi: 10.1186/s12870-019-1702-5 (PMC6434833; doi:10.1186/s12870-019-1702-5)
Supplement: Supplementary file 9 — Table S2. Oligonucleotides used in this study. (PDF 111 kb) [file 12870_2019_1702_MOESM9_ESM.pdf]

Additional file 9: Table S2: Oligonucleotides used in this study.

<table>
<tr><th>Name of primer</th><th>Sequence (5’ – 3’)</th><th>Primer efficiency</th></tr>
<tr><td>MvCPS1-F</td><td>TGGTCAAATGAATGGCTCTGC</td><td rowspan="2">78%</td></tr>
<tr><td>MvCPS1-R</td><td>CGAGGTAGGCAGGAATACCA</td></tr>
<tr><td>MvELS-F</td><td>TCAACCTCAAAATTGCCCCTT</td><td rowspan="2">104%</td></tr>
<tr><td>MvELS-R</td><td>TCAGCTTTCCCTCCGACCT</td></tr>
<tr><td>CYP71AU87-F</td><td>CGTGGCTTGGGTGGATTAGT</td><td rowspan="2">95%</td></tr>
<tr><td>CYP71AU87-R</td><td>GGCTTTGACGCTGTCCCTAT</td></tr>
<tr><td>Mv-EF1a-F</td><td>ATGCTCCGGTTCTTGACTGT</td><td rowspan="2">84%</td></tr>
<tr><td>Mv-EF1a-R</td><td>GGCTTGGTGGGAACCATCTT</td></tr>
<tr><td>MvCYP71AU87-Full length-F</td><td>TAAGCAGGATCCATGGAGAAAATTCAAATACTTTACATTG</td><td></td></tr>
<tr><td>MvCYP71AU87-Full length-R</td><td>TGCTTACTCGAGTCAATTTTCAAACTGATCTGGG</td><td></td></tr>
<tr><td>MvCPR-Full length-F</td><td>TAAGCAGCGGCCGCATGCAATCCACTTCTGAG</td><td></td></tr>
<tr><td>MvCPR-Full length-R</td><td>TGCTTATCTAGATTACCATACATCACGCAG</td><td></td></tr>
</table>
